# Driving diverse bond functionalisation with *N*-heterocyclic silylene-coinage metal–aryl complexes†

**DOI:** 10.1039/d5sc00879d

**Published:** 2025-06-30

**Authors:** Moushakhi Ghosh, Kumar Gaurav, Prakash Panwaria, Rishukumar Panday, Srinu Tothadi, Shabana Khan

**Affiliations:** a Department of Chemistry, Indian Institute of Science Education and Research Pune Dr Homi Bhabha Road, Pashan Pune-411008 India shabana@iiserpune.ac.in; b Analytical and Environmental Sciences Division and Centralized Instrumentation Facility, CSIR-Central Salt and Marine Chemicals Research Institute Gijubhai Badheka Marg Bhavnagar-364002 India

## Abstract

Anchoring homolytic and heterolytic bond functionalisation at low-coordinate coinage metal centres is important due to their potential use as active catalysts in organic transformations. In the realm of carbene-stabilised coinage metal chemistry, heteroatom functionalised coinage metal precursors synthesised from such bond activations have long been explored. Interestingly, *N*-heterocyclic silylene, being an equally potent neutral donor ligand, has not been used for the same. Of note, carbene-stabilised heteroatom functionalised coinage metal precursors are vastly developed with copper centres only, while silver has been underexplored. This work reports the isolation of a variety of [PhC{N(^*t*^Bu)}_2_SiN(SiMe_3_)_2_] (1) coordinated aryl-copper(i) and aryl-silver(i) complexes (2–8). We have also examined the reactivity pattern of organo-copper with differently substituted silylenes (9–11). These complexes were then utilised to cleave various homolytic and heterolytic bonds to access silylene-coordinated heteroatom functionalised coinage metal complexes (12–24). We have shown the reaction of reactive aryl-coinage metal precursors towards homolytic bonds, having B–B and Se–Se bonds, which led to the formation of an NHSi-supported dimeric μ-boryl bound Cu(i) complex (12) and a new class of unprecedented NHSi-supported coinage metal-selenogenolates (14–16). These aryl-coinage metal precursors also smoothly afforded several elusive NHSi-copper and silver amides (17–22) *via* N–H bond cleavage. A heterolytic cleavage of the P–Si bond resulted in the formation of NHSi stabilised copper and silver phosphide complexes (23 and 24), among which the latter is the first precedent of the dimeric Ag-phosphide complex. Lastly, we have utilised NHSi → copper–aryl complexes as aryl transfer reagents in C–C coupling reactions, which led to the formation of products in excellent yields with a high TON. The analogous silver complex was employed in the three-component α-aminonitrile synthesis efficiently. Our report establishes NHSi coordinated aryl copper and silver complexes as a perfect and robust platform for accessing a diverse array of reactive coinage metal precursors that were hitherto unknown.

## Abstract 2

Harnessing unprecedented *N*-heterocyclic silylene ligated heteroatom functionalized copper and silver complexes.

## Graphical abstract

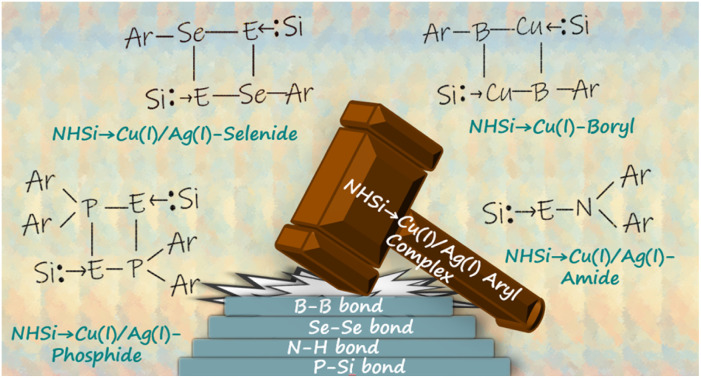

## 1 Introduction

**Chart 1:**
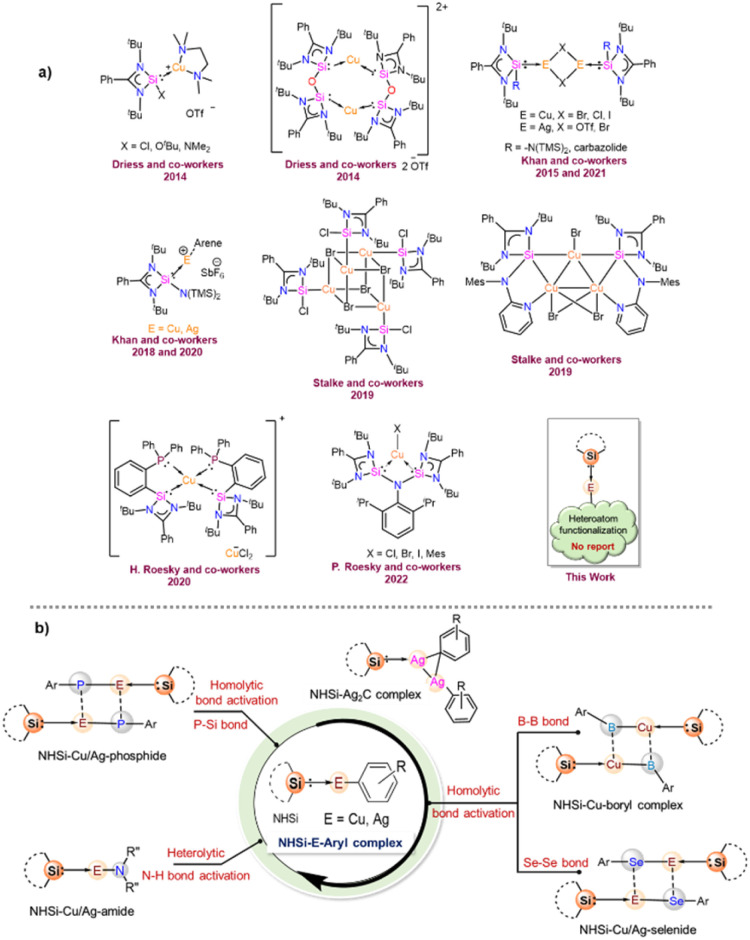
(a) Reported benzamidinato silylene coordinated Cu(i) complexes to date. No NHSi-coordinated Cu(i)/Ag(i)–aryl complex is reported;^7*e*,*g*,*i*,*j*,11^ (b) overview of the present work.

The *N*-heterocyclic silylenes (NHSis), known as heavier congeners of *N*-heterocyclic carbenes (NHCs), are now considered one of the formidable classes of neutral donor ligand systems.^1^ Over the years, several NHSis have been isolated and utilised in stabilising reactive main group species,^2^ coordinating transition metals for homogeneous catalysis,^3^ and activating small molecules.^1*c*,4^ Groundbreaking studies by various pioneering scientists have empowered NHSis as one of the promising ligand motifs for stabilising numerous elusive and reactive species.^1*e*,5^ Despite their superior catalytic activities in several organic transformations, the NHSi stabilised coinage metal complexes are comparatively less studied than the NHC ones.^6^ However, recent years have witnessed a growth in isolating differently functionalised NHSi-coordinated coinage metal complexes and exploring their catalytic activities (Chart 1a).^7^ Given our particular interest in this field,^7*a*–*g*^ we continue to be intrigued by the future possibilities of NHSi-based coinage metal complexes as useful synthons beyond the coordination of coinage metal halides to encompass a broader range of synthetic possibilities. The coordination of NHSis with coinage metals having heteroatom functionalisation remains uncharted territory until now. Many NHC-based copper [Cu(i)] synthons have been isolated and studied for bond activation and homogeneous catalysis.^8^ Interestingly, the analogous situation with silver [Ag(i)] is far more underexplored. This is primarily because of the higher thermal decomposition of Ag(i) complexes under ambient conditions. Also, carbene–Ag(i) complexes are chosen precursors for transmetallation reactions with other transition metals, such as Cu, Au, Fe, Ni, and Co.^9^ The bond strength between carbenes and transition metals is greater than that of carbene–Ag(i) complexes, facilitating a smoother transmetallation process. In this context, aryl–coinage metal precursors(i) are a very reactive class of complexes,^8*c*,10^ which can be explored in bond activations due to the lability of E–aryl bonds (E = Cu and Ag). Also, NHC-based Cu(i) complexes with anionic C-donor atoms have been explored for their catalytic application and functionalisation reactions, such as the formation of Cu–X bonds (X = –SR, –PR_2_, –OR, and –NR_2_), intramolecular hydroalkoxylation of alkynes, the CuAAC reaction, and defluoro-borylation of fluoroalkenes.^8*p*^ However, such utilisation of carbene–Ag(i)–aryl complexes is missing in the literature. Very recently, the NHC-coordinated Ag(i)–mesityl complex has been used as a precursor for crafting graphitic carbon nitride (g-C_3_N_4_),^12^ paving the way for their further exploration in organometallic and material chemistry. All these findings inspired us to isolate the NHSi-based Cu(i) and Ag(i)–aryl complexes (2–8), creating a platform for the further functionalisation of the labile Cu/Ag–aryl bonds (Chart 1b). In this work, we have demonstrated the coordination behaviour of aryl–coinage metal precursors exclusively with benzamidinato silylene, [PhC{N(^*t*^Bu)}_2_SiN(SiMe_3_)_2_] (1), and then showcased the reactivities of these precursors towards various homolytic and heterolytic bond cleavage reactions (12–24) (Chart 1b). We have also briefly checked the reactivity pattern of different NHSis (9–11) with organocopper complexes to establish the breadth of the methodology. While the reactions of NHSi with Cu(i)–aryl precursors afforded di-coordinated NHSi → Cu–aryl complexes (2–5) smoothly, the reaction of Ag(i)–mesityl afforded a unique Ag_2_C ring with argentophilic interaction (6). The stability of the complex is interesting because of the high level of angle strains and torsional strains of the thermodynamically unstable three-membered Ag_2_C ring. Interestingly, there is no precedent of such a complex with NHSis to date. However, the reactions with other Ag(i)–aryl precursors yielded nearly linear coordinated complexes 7 and 8 due to the enhanced steric effects of aryl groups. The isolation of these complexes created a perfect platform for bond functionalisation. Inspired by Sadighi's isolation of NHC-supported Cu(i)–boryl complexes [(IPr)Cu(Bpin), (ICy)Cu(Bpin)], catalytically active for CO_2_ reduction and a few isolated carbene-stabilised copper–boryl complexes,^13^ we isolated NHSi–Cu–boryl complexes *via* diboron bond cleavage, which yielded complex 12 with a dimeric μ-boryl framework. Additionally, given the scarcity of NHC/CAAC-supported chalcogenides^14^ and the absence of NHSi-coordinated coinage metal chalcogenolates, we also reacted NHSi → Cu(i)/Ag(i)–aryl complexes with different diselenides, leading to the isolation of NHSi–Cu/Ag selenogenolate (14–16). Very recently, we have shown that the NHSi → Cu–amides can demonstrate fascinating luminescence properties.^15^ Along with this, N–H bond cleavage is also an important part of cross-coupling reactions; hence, we attempted syntheses of Cu/Ag–amides (17–22).^16^ Lastly, employing the σ-bond metathesis reaction of Cu/Ag–C and P–Si bonds, we were able to isolate rarely occurring Cu(i) and Ag(i) phosphide complexes (23–24). Meanwhile, NHC–Cu–phosphides have been reported only in limited instances.^8*i*^ To the best of our knowledge, the precedence of low-coordinate Ag–phosphide is still elusive. Overall, this vast piece of work intends to highlight the synthetic versatility and facile bond activation potential of NHSi–Cu/Ag–aryl complexes towards various energy demanding bonds such as B–B (∼462.86 kJ mol^−1^), Se–Se (∼239.48 kJ mol^−1^), N–H (∼387.41 kJ mol^−1^), and P–Si (∼263.58 kJ mol^−1^) (Table S4, see the ESI for the details†). To understand the bonding nature of all the newly synthesised complexes, density functional theory (DFT) calculations were performed using the Gaussian 09 program package (see the ESI for details†), and the description of theoretical input is provided at the relevant places.

To establish the significance of such organo-coinage metal precursors in catalytic reactions, we performed a few brief proof-of-concept catalytic model reactions, such as C–C cross-coupling reactions and a three-component α-aminonitrile synthesis, which afforded the desired compounds in excellent yields (*vide infra*).

## 2 Results and discussion

### 2.1 Synthesis of NHSi–E–aryl (E = Cu and Ag) complexes

#### 2.1.1 Synthesis of NHSi-based Cu(i)–aryl complexes (2–5)

**Scheme 1:**
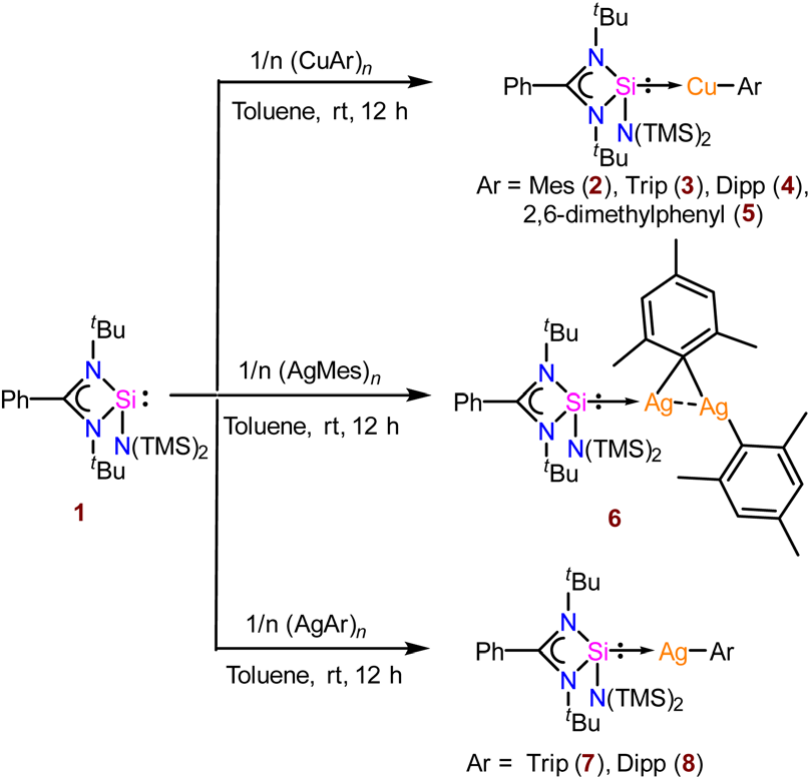
The reaction of compound 1 with various aryl Cu(i) and Ag(i) compounds.

We started our investigation by reacting bis-(trimethylsilyl) amide substituted benzamidinato silylene, [PhC{N(^*t*^Bu)}_2_SiN(SiMe_3_)_2_] (1), with different aryl copper precursors^17^ in toluene at room temperature, which furnished complexes 2–5 (Scheme 1) in good yield.

**Fig. 1:**
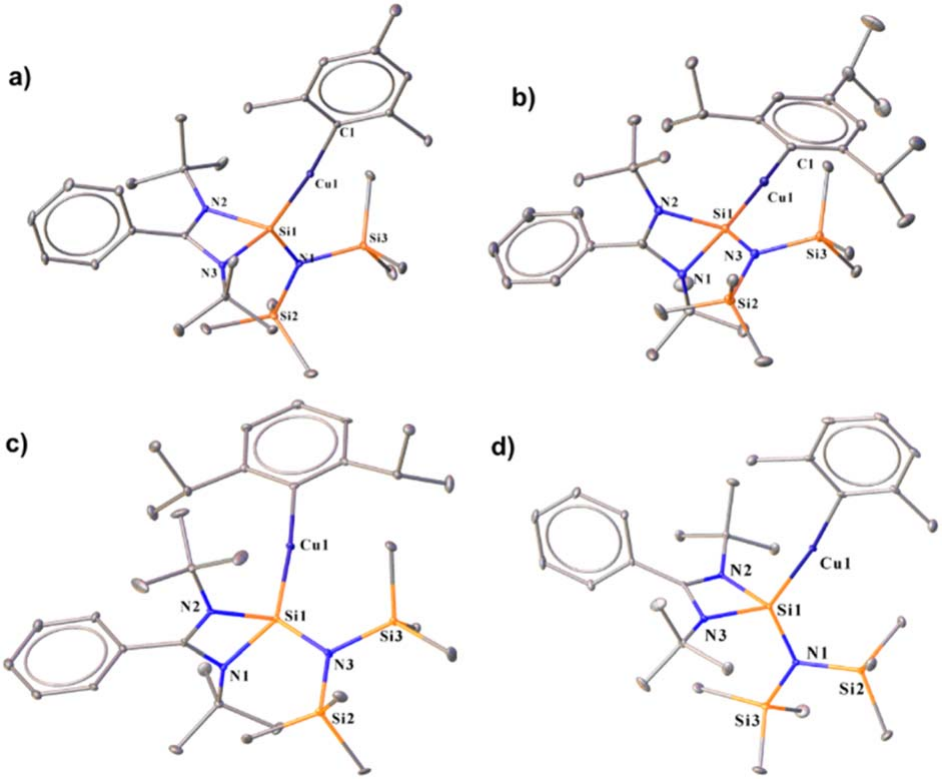
Molecular structures of 2 (a), 3 (b), 4 (c), and 5 (d). The bond distance (Å) and bond angle (°) are discussed in the ESI.†

The ^29^Si{^1^H} spectra display a broad peak at *δ* 6.5 and *δ* 6.6 ppm for the Si(ii) → Cu centre for complexes 2 and 3–5, respectively. The molecular structures of 2–5 demonstrate that the Si(ii) centres adopt a distorted tetrahedral geometry (Fig. 1). The Si–Cu bond lengths are 2.2590(8) Å (complex 2), 2.2407(8) Å (complex 3), 2.246(2) Å (complex 4), and 2.242(1) Å (complex 5), which are shorter than the previously reported Si–Cu bond length in the carbazole-substituted benzamidinato silylene stabilised dimeric Cu_2_Br_2_ complex (2.203(2) and 2.212(2) Å),^7*c*^ and longer than that in [PhC{N(^*t*^Bu)}_2_SiN(SiMe_3_)_2_] stabilised Cu_2_Br_2_ (2.222(2) Å). But they match closely with that in the dimeric Cu_2_I_2_ complex of [PhC{N(^*t*^Bu)}_2_SiN(SiMe_3_)_2_] (2.243(3) and 2.250(3) Å).^6*a*^ They are significantly longer than the predicted bond length for the NHSi → Cu(i) complex (2.061 Å) by Frenking and co-workers.^18^ It was observed that the Si–Cu bond lengths decrease with increasing steric bulk around the aryl group, following the trend 2 > 4 > 5 > 3. They feature almost linear geometry around the Cu(i) centre featuring a bond angle Si1–Cu1–C1 of 173.28(6)° (complex 2), 170.72 (7)° (complex 3), 172.7(1)° (complex 4) and 172.5(1)° (complex 5) which is in accordance with the previously reported IMes (1,3-bis-(2,4,6-trimethylphenyl)imidazole-2-ylidene) stabilised Cu–mesityl complex [173.53(9)°]^19^ but the Si–Cu–C angles of complexes 2–5, are shorter than that of the carbene coordinated copper-mesityl complexes (in the range of [174.53(6)–178.06(12)°]).^19,20^ To understand the implications of these bonding patterns on the electronic structure, the frontier molecular orbital (FMO) analysis was carried out (Fig. S111†). This reveals that the highest occupied molecular orbital (HOMO) is primarily localised over the Cu d orbital and the π orbital of the mesityl group. Conversely, the electron density in HOMO−1 predominantly resides over the Cu(i)–C_ipso_ bond, with a marginal distribution extending over the Si(ii) → Cu bond and the mesityl group. Notably, HOMO−3 is majorly localised over the Si(ii) → Cu bond, exhibiting a minor dispersion over the Cu(i)–C_ipso_ bond and the amidinate fragment. The HOMOs of complexes 3 and 4 (see the ESI for computational details†) show a predominant contribution from the Cu(i)–C_ipso_ bond and a minor involvement from the Si(ii) → Cu bond and the aryl group. It is important to note that the electron density on C_ipso_ is more in complex 3 (35%) and 4 (35%) in the HOMO than in complex 2 (27%), indicating the stronger electron-donating effect of Dipp (2,6-diisopropylphenyl) and Trip(2,4,6-triisopropylphenyl) over the Mes (2,4,6-trimethylphenyl) group. However, the lowest unoccupied molecular orbital (LUMO) for all complexes is predominantly localised over the benzamidinato fragment.

#### 2.1.2 Synthesis of NHSi-based Ag(i)–aryl complexes (6–8)

**Fig. 2:**
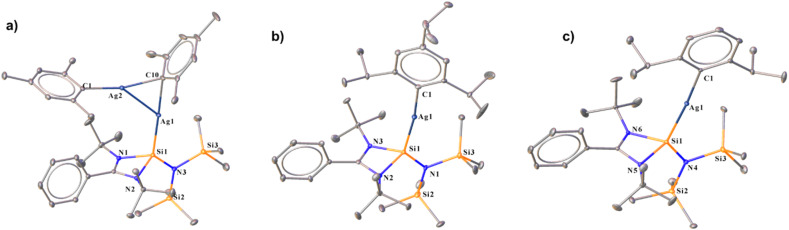
Molecular structures of 6 (a), 7 (b) and 8 (c). The bond distance (Å) and bond angle (°) are discussed in the ESI.†

Unlike organocopper(i) complexes, the chemistry of organosilver(i) complexes has not been explored much, as we already discussed. There are a few examples of ligated silver(i)–mesityl moieties in the literature.^21^ Upon reaction of compound 1 with mesityl silver^17*c*^ in toluene at room temperature, an interesting μ-mesityl bound dimeric silver(i) complex (6) was obtained (Scheme 1). The ^29^Si{^1^H} NMR spectrum of 6 indicated the formation of the desired complex by showing two doublets at *δ* 14.7 and 11.5 ppm due to the presence of two NMR active nuclei of Ag (109 and 107). Complex 6 features a 3c–2e bond involving the C_ipso_ atom of the mesityl group with two silver atoms (Fig. 2), resulting in close proximity of Ag1–Ag2 (2.7453(5) Å). The Ag1–Ag2 bond length of 6 is much shorter than the sum of the van der Waals radii (3.440 Å).^22^ The aryl group bound to coinage metal complexes often shows μ-bonding in the aggregated form to form a polynuclear complex (Fig. 2).^23^ The Si1–Ag1 bond length in 6 is 2.3900(9) Å, which is similar to that of the carbazole-substituted benzamidinato silylene coordinated tetrameric Ag_4_I_4_ complex.^7*c*^ Kays and co-workers reported an *m*-terphenyl group-bound dimeric Ag_2_ core with a Ag⋯Ag distance of 2.6706(3) Å, which is shorter than that of complex 6.^24^ While the Ag1–C1 bond in 6 is shorter (2.106 Å), that of the μ-bridged mesityl-bound Ag atoms are longer (Ag1–C10 2.215(3) and Ag2–C10 2.211(3) Å) and form almost symmetrical bonds with the C10 atom. This is the first NHSi-supported three-membered Ag_2_C ring system. Sadighi and co-workers isolated a triangular [Ag_2_H]^+^ core stabilised by 1,3-bis(2,6-diisopropylphenyl)imidazolin-2-ylidene (SIDipp),^25^ but such a molecular framework with NHSis is not known. Furthermore, we also tried to validate whether such a heteroleptic complex is present in the solution; thus, we performed diffusion ordered NMR spectroscopy (DOSY). In this method, molecules are differentiated according to their diffusion coefficient (*D*), which correlates with their hydrodynamic radius.^26^ We found the hydrodynamic radius of complex 6 to be 6.24 Å in toluene-*d*_8_, which suggests that it stays as a monomeric complex in solution. Our attempt to crystallise them in the monomeric form in other solvents like THF and DCM was unsuccessful. The FMO analysis shows that the HOMO of complex 6 shows the contribution over the delocalised mesityl–Ag–C_ipso_ and d orbital of the Ag atom bonded to NHSi, whereas HOMO−1 shows the contribution over the localised mesityl group and d orbital of the Ag atom (Fig. S112†). HOMO−2 is localised over the mesityl group, whereas HOMO−3 is delocalised over the Si(ii) → Ag_2_C ring connected to the μ-bridged mesityl group (see the ESI for computational details†). Furthermore, natural bond orbital (NBO) analysis was performed to understand the presence of metallophilic interaction in 6, which confirms the presence of argentophilic interaction (Fig. S120†). The second-order perturbative energy [*E*^(2)^] was hence calculated to quantify the bonding–antibonding interactions. The *E*^(2)^ value for the delocalisation of electron density from the Ag(i) centre to the σ* orbital of the Ag(i)–Si bond was 2.10 kcal mol^−1^. However, the *E*^(2)^ value for the Ag(i) centre attached to the NHSi to the σ* orbital of the Ag(i)–C_ipso_ bond was found to be 1.71 kcal mol^−1^. The Wiberg bond index (WBI) of the Ag⋯Ag bond was found to be 0.072, which is in the range (0.022–0.094) of reported literature on argentophilic interaction.^27^ Interestingly, treatment of AgTrip and AgDipp with 1 yielded NHSi-bound linear Ag(i)–aryl complexes (7 and 8) (Scheme 1). The ^29^Si{^1^H} NMR spectra for complexes 7 and 8 show two doublets appearing at *δ* 14.3 and 10.9; 14.1 and 10.8 ppm, respectively, due to the coupling of ^109^Ag and ^107^Ag with the ^29^Si centre. The Si–Ag bond lengths for complexes 7 and 8 are 2.400(1) and 2.387(6) Å, respectively, whereas the Si–Ag–C bond angles for complex 7 [172.21(6)°] deviated more from linearity than that of complex 8 [173.44(5)°] due to more steric bulk for the former than the latter, as also observed in complexes 3–4 (Fig. 1). The HOMO of both these complexes resides on the NHSi → Ag–C_aryl_ fragment (Fig. S113†). The rest of the MOs for complexes 7 and 8 are similar to those of complexes 3 and 4.

#### 2.1.3 Reaction of organocopper towards differently functionalised benzamidinato silylene

**Scheme 2:**
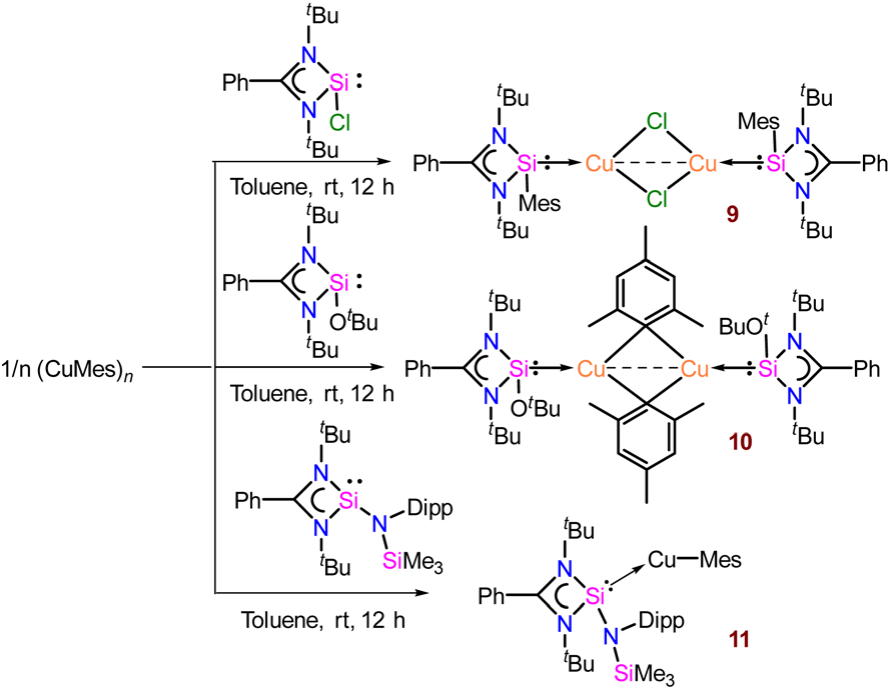
Syntheses of complexes 9–11.

To monitor the reactivity pattern of organo-copper reagents with differently substituted NHSis, we further probed the reactivity pattern of [PhC{N(^*t*^Bu)}_2_SiCl], [PhC{N(^*t*^Bu)}_2_SiO^*t*^Bu], and newly synthesised [PhC{N(^*t*^Bu)}_2_Si{N(Dipp)SiMe_3_}] with CuMes (Scheme 2).^28^ For that, we have treated [PhC{N(^*t*^Bu)}_2_SiCl], [PhC{N(^*t*^Bu)}_2_SiO^*t*^Bu], and [PhC{N(^*t*^Bu)}_2_Si{N(Dipp)SiMe_3_}] with mesityl copper in a 1 : 1 molar ratio, which furnished complexes 9, 10, and 11, respectively.^29^ We selected these variations based on their differences in electronic and steric parameters, anticipating diverse reactivities. The formation of 9 proceeded through σ-bond metathesis at the labile Si–Cl moiety, and the mesityl group migrated to the Si(ii) centre to form a dimeric Cu_2_Cl_2_ core (Fig. S136†). The peak at *δ* 32.7 ppm in the ^29^Si{^1^H} NMR spectrum is more deshielded than the previously reported dimeric copper(i) chloride of compound 1 (*δ* 4.79 ppm),^11*f*^ which might be due to the presence of the σ-donating mesityl group at the silicon(ii) centre. 9 crystallises in the monoclinic *P*2_1_/*n* space group (see Fig. S136†) and is isostructural to the dimeric Cu_2_Cl_2_ complex of 1.^11*f*^

**Fig. 3:**
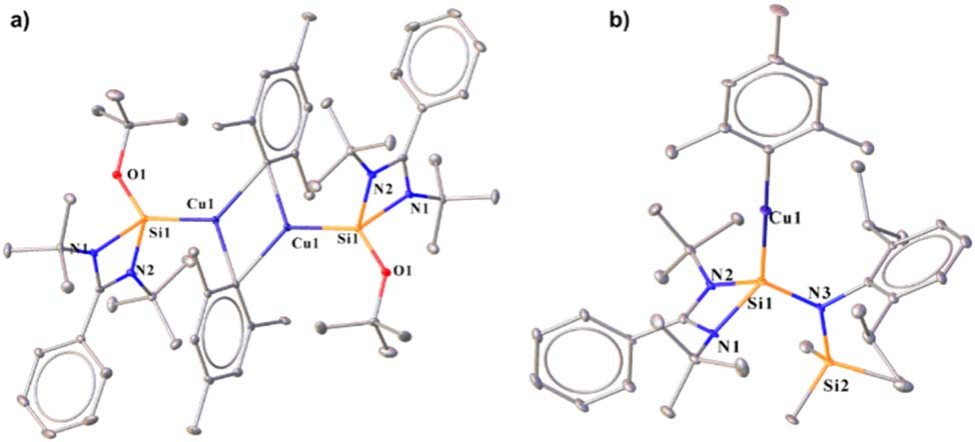
Molecular structures of 10 (a) and 11 (b). The bond distance (Å) and bond angle (°) are discussed in the ESI.†

The silicon(ii) centre of 9 attains a distorted tetrahedral geometry with a Si–Cu bond length of 2.213(1) Å.^11*f*^ On the other hand, [PhC{N(^*t*^Bu)}_2_SiO^*t*^Bu] furnishes μ-dimeric mesityl-bridged Cu(i) complex, 10 (Scheme 2). The ^29^Si{^1^H} NMR spectrum shows a broad peak at *δ* 3.8 ppm, deshielded from [PhC{N(^*t*^Bu)}_2_SiO^*t*^Bu] (*δ*−5.0 ppm),^28*b*^ but comparable to the dimeric Cu_2_Cl_2_ complex of 1 as mentioned above. Complex 10 crystallises in the triclinic *P*1̄ space group with a Si–Cu bond length of 2.249(1) Å, and the Cu–C bond lengths are 2.170(4) and 2.132(4) Å, suggesting the μ-bridging fashion (Fig. 3). The tri-coordinated Cu(i) adopts a Si1–Cu1–C1 bond angle of 119.8(1)°. The Cu⋯Cu distance in 10 is 2.335(6) Å, which is shorter than the Cu⋯Cu distance (2.46 Å) in mesityl copper.^10^ To broaden the scope of the reactivity pattern, we performed a reaction of mesityl copper with newly synthesised [PhC{N(^*t*^Bu)}_2_Si{N(Dipp)SiMe_3_}] and obtained complex 11, similar to complex 2. Formation of complex 11 was confirmed by ^29^Si{^1^H} NMR, which shows a broad peak at *δ* 11.8 ppm, which is further deshielded than [PhC{N(^*t*^Bu)}_2_Si{N(Dipp)SiMe_3_}] (*δ* 2.98 ppm). The Si–Cu distance in complex 11 is observed to be 2.227(2) Å, shorter than that in complex 2 {2.2590(8) Å}, and the Cu–C distance is 1.934(3) Å, with a bond angle of 179.68(9)° around Si–Cu–C centres (Fig. 3). The abovementioned reactivity of different silylenes clearly outlines the influence of steric and electronic properties on the structure of the final product.

### 2.2 Reactivities of NHSi-based E–aryl complexes (2 and 7) towards various bond cleavage reactions

Based on various structural motifs obtained (2–11) through the coordination ability of differently functionalised silylene with organo-coinage metal precursors, complexes 2 and 7 were chosen for further exploration in a range of bond activation reactions.

#### 2.2.1 Synthesis of the NHSi-based Cu(i)–boryl complex (12) *via* B–B bond cleavage

Various NHC and CAAC-coordinated low-valent boron species have found application in optoelectronics.^30^ The coinage metal complexes of diborene and boron–boron triple-bonded systems also showcased interesting photophysical behaviour.^31^ Thus, we were interested in probing the reaction of 2 with the diboron (B–B bond) reagents^32^ in the quest of homolytic bond cleavage.^33^

**Scheme 3:**
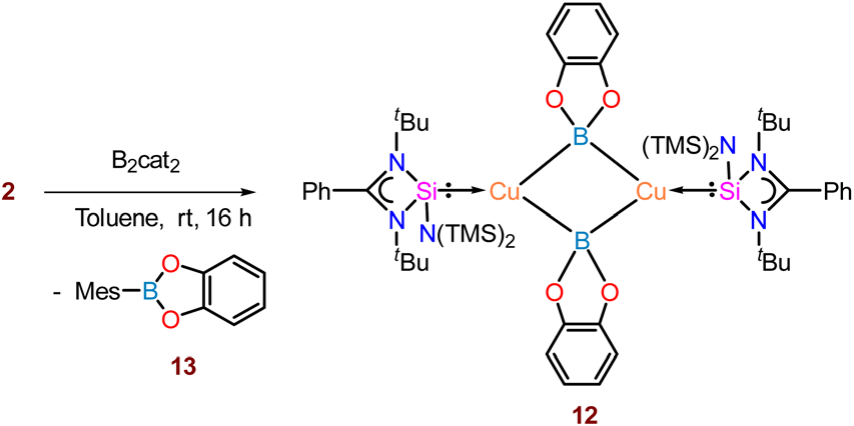
Reaction of complex 2 with a diboron compound (B_2_cat_2_).

**Fig. 4:**
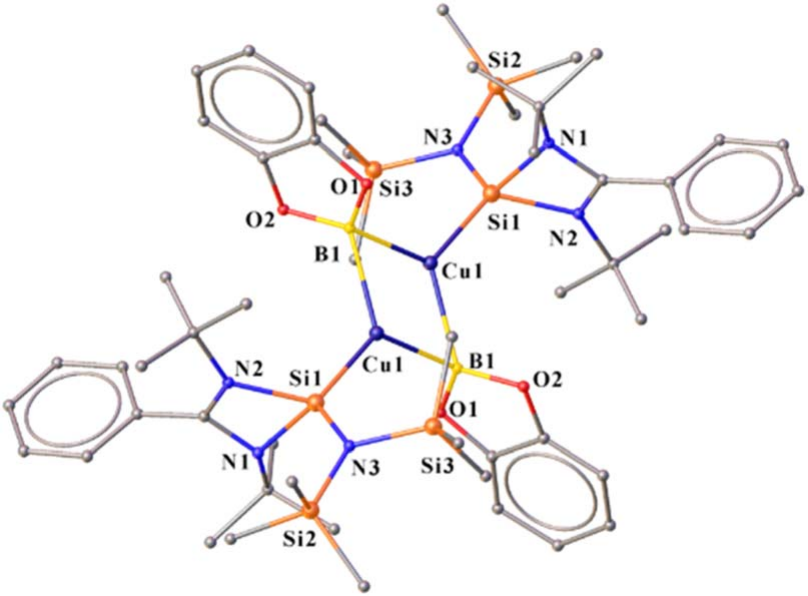
Molecular structure of 12.

**Chart 2:**
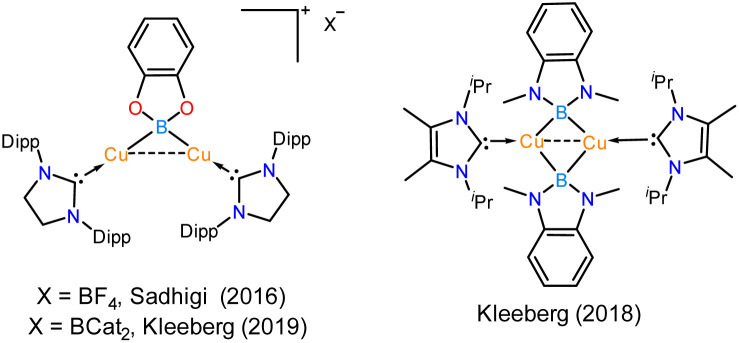
Previously reported examples of NHC-supported μ-boryl Cu(i) complexes.^34,35^

Thus, we performed a reaction of less sterically bulky bis(catecholato)diboron (B_2_cat_2_) with complex 2 (Scheme 3) in toluene. In this case, we could obtain dimeric [PhC{N(^*t*^Bu)}_2_SiN(SiMe_3_)_2_}_2_Cu_2_B_2_Cat_2_] (12) and Mes–Bcat (13) as a mixture of the products. The isolation of complex 12 also indicated the higher reactivity of sterically less hindered B_2_cat_2_, which was previously pointed out.^34^ The molecular structure of complex 12 (Fig. 4) established the μ-coordination mode of the boryl linkage to Cu(i) coordinated with NHSi unambiguously. However, the poor quality of the crystals prevented us from discussing the metrical parameters. Analogous NHC stabilised μ-boryl Cu(i) complexes were reported by Sadhigi, Kleeberg, and co-workers, which show a μ-boryl Cu(i) core (Chart 2).^35^ We could not get a clean ^1^H NMR spectrum of 12 as it was isolated as a mixture with 13 (see the ESI†). However, ^29^Si{^1^H} NMR spectra revealed two sharp singlets at *δ* 6.3 and 7.1 ppm, corresponding to the –SiMe_3_ moiety, and a singlet at *δ* 3.7 ppm, presumably indicating the Si(ii)–Cu(i) bond. Although there are a few Cu–μ-boryl complexes reported in the literature, the understanding of the associated FMOs is largely missing, apart from the work by Tilley and co-workers.^35*e*^ Drawing inspiration from this, we analysed its electronic structure, which reveals that the HOMO of the dimeric Cu(i)–μ^2^–Boryl complex (12) (Fig. S114†) is localised over the Cu_2_B_2_ ring. The orbital composition of the HOMO shows that the localisation of electron densities at Cu centres is 19 and 13%, while at B centres, it is 30 and 22%, respectively. This 3c–2e bonding situation was further confirmed by the natural localised molecular orbital (NLMO) analysis, showing that the contribution of each Cu centre and B orbital in the Cu–B–Cu bond is 13, 6, and 73%, respectively. HOMO−1 also has a contribution over the Cu_2_B_2_ ring along with the Si(ii) → Cu bond, whereas the electron density in the HOMO−2 is predominantly localised over the Si(ii) → Cu bond, along with the contribution from the catechol–boryl moiety.

**Fig. 5:**
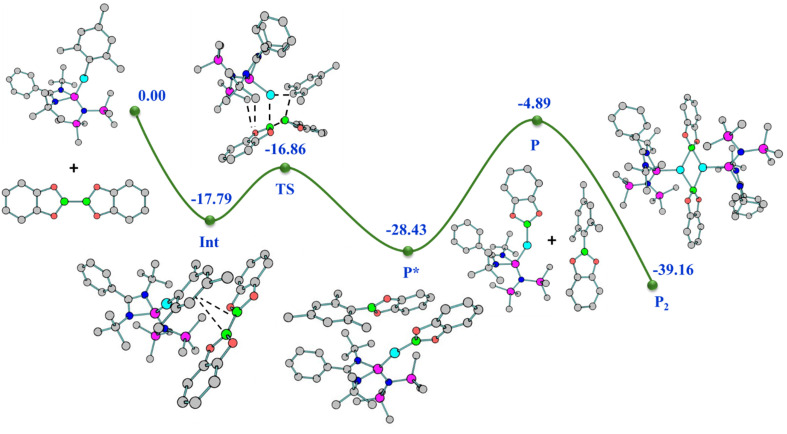
Relative energy (kcal mol^−1^) (not drawn to scale) reaction profile for the formation of complex 12 from complex 2 with B_2_cat_2_.

The reaction pathway shows that complex 2 forms an intermediate (Int) involving non-covalent interaction between boron atoms of B_2_pin_2_ and the mesityl ring (Fig. 5), proceeding *via* an intermediate to give complex 12 (detailed analyses provided in the ESI†). Interestingly, the preparation of aryl boronate esters either involves Suzuki–Miyaura cross-coupling for C–B bond formation^36^ or base-mediated ArB(OH)_2_ formation.^37^ Thus, the formation of 13 as a byproduct of this reaction in the crystalline form gives easy access to mesityl boronic ester (see the ESI†). It is important to note that a similar reaction with complex 7 failed to yield the NHSi → Ag–boryl complex, likely due to poor thermal stability and rapid dissociation of the Ag–B bond. Although we attempted to monitor the fate of the product using ^29^Si{^1^H} NMR spectroscopy, we were able to detect a Si(ii)–bound Ag(i) species. However, the exact nature of the species formed alongside Trip–Bcat remains unclear at this stage.

We also tried the reaction of complex 2 with other diboron agents discussed in the ESI.†

#### 2.2.2 Synthesis of NHSi-based Cu(i) and Ag(i) selenogenolate complexes (14–16) *via* Se–Se bond cleavage

**Scheme 4:**
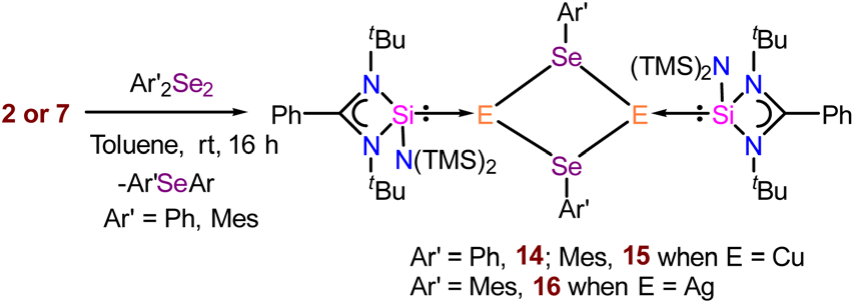
The reaction of complexes 2 and 7 with diaryl diselenide (Ar′_2_Se_2_).

**Fig. 6:**
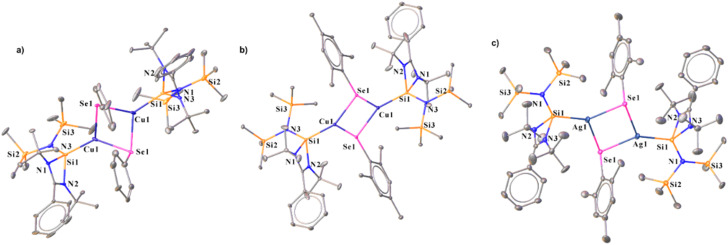
Molecular structures of 14 (a), 15 (b), and 16 (c). The bond distance (Å) and bond angle (°) are discussed in the ESI.†

To access NHSi-supported coinage metal chalcogenolate complexes, we treated complex 2 with diphenyl diselenide (Ph_2_Se_2_) and sterically demanding bis(2,4,6-trimethylphenyl) diselenide (Mes_2_Se_2_) in toluene at room temperature (Scheme 4). This led to the formation of immediate white precipitates, which were characterised by ^1^H and ^77^Se{^1^H} NMR spectra as MesSePh^38^ and Mes_2_Se,^39^ respectively (see the ESI†). The filtrate part of the reaction mixture afforded suitable single crystals of 14 and 15. The reaction of complex 7 with Mes_2_Se_2_ also afforded NHSi stabilised Ag(i) mesitylselenogenolate (16) (Scheme 4). The ^29^Si{^1^H} NMR spectra display two sharp singlets for the –SiMe_3_ groups at *δ* 4.4 and 5.9 ppm for 14, and *δ* 5.3 and 6.7 ppm for 15, respectively. A broad peak for Si(ii)–Cu was observed in ^29^Si{^1^H} NMR spectra at *δ* 5.9 and 3.2 ppm for 14 and 15, respectively. Complex 16 shows a doublet at *δ* 4.12–4.47 and 9.13–9.49 ppm, similar to complex 7, discussed earlier. Complexes 14–16 display a dimeric E_2_Se_2_ (E = Cu and Ag) core with the Si–E bond lengths of 2.248(3), 2.2631(6), and 2.401(1) Å, for 14, 15, and 16, respectively. The E–Se distances in 14–16 are 2.456(1), 2.5076(6), and 2.6245(7) Å, respectively (Fig. 6). They are marginally longer than those in the Ph_3_PCu(μ-SePh)_2_Cu(PPh_3_)_2_·CH_3_CN complex {2.406(1) Å} by Oliver and co-workers and [Ag_4_(μ-SePh)_4_(^i^Pr_2_-bimy)_4_] {2.649(1), 2.748(1) Å} by Corrigan and co-workers.^14*d*,40^ The Cu⋯Cu distances in 14 and 15 are 2.938(2) and 3.4398(6) Å, respectively, which are longer than the Cu⋯Cu distance in the Ph_3_PCu(μ-SePh)_2_Cu(PPh_3_)_2_·CH_3_CN complex {2.738(1) Å},^41^ but shorter than the Cu⋯Cu distance [3.1378(9) and 3.255(1) Å] in [PhC(N^*t*^Bu)_2_N(TMS)_2_SiCu_2_X_2_] (X = Cl and Br).^11*f*^ The Ag⋯Ag distance is 3.8110(7) Å in complex 16, significantly longer than the argentophilic interaction range and complex 6.^22^ Noteworthy to mention that there is no report of such dimeric carbene-coordinated Ag(i) selenogenolates in the literature.^40^ In a similar line, we also tried similar reactions with diphenyl sulfide (Ph_2_S_2_) and diphenyl telluride (Ph_2_Te_2_) for consecutive bond activation, but could not obtain the desired compounds. FMO analysis shows that the HOMO of complexes 14 and 15 (Fig. S115†) is prominently concentrated over the dimeric Cu_2_Se_2_ ring, demonstrating substantial contributions from the Si(ii) centres and the phenyl rings, whereas HOMO−3 is localised over the Si(ii) → Cu bond for complex 15. As we observed the presence of cuprophilic interaction in the molecular structure of 14, we went on to examine its *E*^(2)^ value, which shows 0.62 kcal mol^−1^ strength for Cu⋯Cu interaction (Fig. S121†), less than the reported strength.^42^ It thus denotes the weaker nature of cuprophilic interaction. We have also calculated the change in the Gibbs free energy (Δ*G*) of 14 in the temperature range of 0–450 K to investigate the thermal stability.

**Fig. 7:**
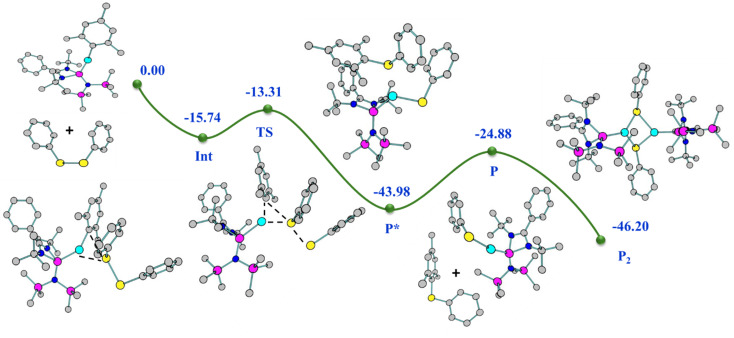
Relative energy (kcal mol^−1^) (not drawn to scale) reaction profile for the formation of complex 14 from complex 2 with Ph_2_Se_2_.

It was found that 14 is stable in the dimeric form up to the temperatures of 380 and 420 K in the solution and gas phase, respectively (see the ESI for computational details†). The Int observed in the reaction pathway for the formation of complex 14 (Fig. 7) is stabilized by the interaction between Cu and Se centers, C–H⋯Se, C–H⋯π, and tetrel bonding. These non-covalent interactions favour the formation of the Cu–Se bond and the breaking of the Cu–C_ipso_ bond, which is visualised in the TS, and confirmed by the relevant geometrical parameters and NBO analysis (see the ESI for details†).

#### 2.2.3 Synthesis of NHSi-based Cu(i)/Ag(i) amide complexes (17–22) *via* N–H bond cleavage

**Scheme 5:**
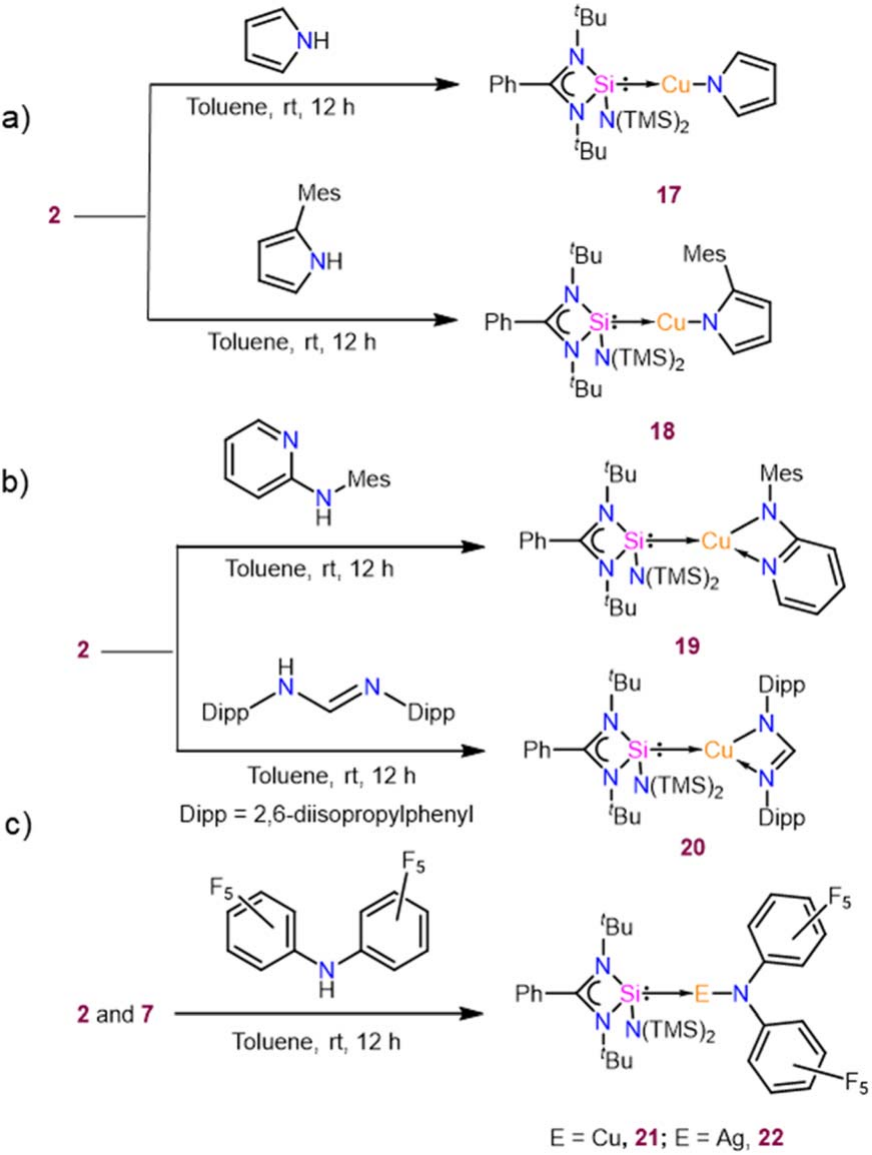
Syntheses of various NHSi-coordinated metal amide complexes [monodentate amides (a), bidentate amides (b), and perfluorinated amides (c)] from 2 and 7.

There are prodigious examples of NHC-coordinated copper alkyl complexes that work as intermediates to different X–H (X = N, O, and C) bond functionalised products, which even led to the isolation of the first NHC-based copper amido and anilido complexes.^8*p*,43^ Recently, carbene metal amide (CMA) complexes have attracted special attention due to their promising photophysical properties.^16,44^ Motivated by the fact that NHSi-metal amide complexes had not been isolated to date until very recently by us,^15^ we delved into the reactivity of the NHSi-ligated Cu(i)/Ag(i) aryl complex towards N–H bond cleavage, which facilitated a straightforward route for NHSi-coordinated metal amide complexes (Scheme 5a). The stoichiometric reaction of complexes 2 and 7, with N–H containing compounds, afforded NHSi-metal amide complexes, which are stable for days under inert conditions at room temperature and months at 0 °C.

**Fig. 8:**
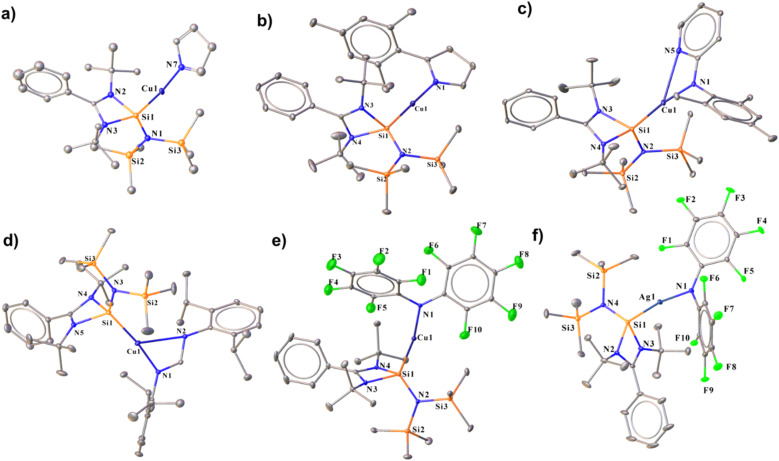
Molecular structures of complexes 17 (a), 18 (b), 19 (c), 20 (d), 21 (e), and 22 (f). The bond distance (Å) and bond angle (°) are discussed in the ESI.†

Complex 17, formed by reacting 2 with pyrrole, shows a broad peak at *δ* 7.8 ppm in the ^29^Si{^1^H} NMR spectrum, and its structure was confirmed by single-crystal X-ray diffraction. The Si1–Cu1 bond length for complex 17 was found to be 2.231(3)Å, and the Cu–N bond length was 1.878(7) Å. Complex 17 features an almost linear Si–Cu–N bond angle of 172.2(5)° (Fig. 8). Similarly, reacting 2 with 2-mesityl-1*H*-pyrrole yielded complex 18, displaying a^29^Si{^1^H} peak at *δ* 7.4 ppm, deshielded relative to 2 due to the π-electron-donating mesityl group. Complex 18 has a Si1–Cu1 bond length of 2.203(1) Å and a Cu1–N1 bond length of 1.874(3) Å (Fig. 8), with a nearly linear Si1–Cu1–N1 angle of 177.5(1)°. The Si–Cu bond distance is comparable to that of the heteroleptic [Cu(tmeda)(PhC(N^*t*^Bu)_2_Si{O^*t*^Bu})][OTf] (tmeda = *N*,*N*,*N*,*N*-tetramethylethylenediamine) complex [2.2003(6) Å]. Furthermore, we have performed FMO visualisation along with NBO analysis for all the isolated NHSi → E–amide complexes (17–22) to elucidate the nature of the bonding. The HOMO of complex 17 is located over the pyrrolato ring whereas HOMO−1 and HOMO−2 have a major contribution from the Si(ii) → Cu(i) bond (Fig. S116†). For 18, the HOMO is localised on the 2-mesitylpyrrolide moiety, HOMO−1 on the Cu(i)–N_amide_ fragment, and HOMO−2 across the Si(ii) → Cu bond (Fig. S116†). After isolating complexes 17 and 18, we were curious to investigate the coordinative variability of N-donor functionalised secondary amines. To explore the coordination ability of N–H systems containing auxiliary N-donor atoms, we obtained a few coordinated Cu(i) complexes (19 and 20) (Scheme 5b). The three-coordinate NHSi–copper(i) complex (19) was isolated by treating 2 with *N*-mesitylpyridin-2-amine, which gives a broad peak at *δ* 6.9 ppm for the Si(ii) centre in the ^29^Si{^1^H} NMR spectrum. Complex 19 features a Si1–Cu1 bond length of 2.1964(8) Å (Fig. 8), which is shorter than the Si(ii) → Cu(i) bond in complex 18. The Cu1–N5 bond length of 19 is 2.654(2) Å, which is significantly longer than the reported IPr coordinated three copper(i) complexes attached to the 2-(2,3,4,5-tetrafluorophenyl) pyridine group (2.185(4) Å).^45^ Thompson and co-workers isolated a few (NHC)Cu(py_2_BMe_2_) {py_2_BMe_2_ = di(2-pyridyl)dimethylborate} complexes, which feature N^N donation to the Cu(i) centre. These complexes possess Cu–N bond lengths ranging from 1.9929(16) to 2.0288(15) Å.^46^ The Si1–Cu1–N1 bond angle in 19 is 172.44(7)°, which deviates from linearity due to the additional N-donation. Stalke and co-workers isolated *N*-mesitylpyridin-2-amine substituted benzamidinato silylene coordinated trimeric Cu(i) halide complexes,^47^ which also feature pyridyl N-donation to the Cu(i) centre with N → Cu bond lengths from 1.995(4) to 2.009(2) Å. These bonds are significantly shorter than the observed N → Cu bond in 19, denoting a greater extent of electron donation in the former one. The amidinate ligands are infinitely versatile in terms of potential structure and substitution patterns.^48^ This prompted us to isolate complex 20 containing the Dipp group substituted formamidine moiety (Scheme 5b) as an example of auxiliary N-donor coordinated secondary amines. This features a broad peak at *δ* 6.6 ppm for Si(ii) attached to three-coordinate Cu(i) centres in the ^29^Si{^1^H} NMR spectrum. Due to an additional N-donor atom, the Si(ii) centre is more shielded than the complexes 17 and 18. Complex 20 is structurally reminiscent of the CAAC stabilised three-coordinate formamidinate copper(i) complex (Fig. 8).^49^ The molecular structure of 20 reveals a Si1–Cu1–N1 angle of 171.24(9)°, whereas, for the CAAC one, the C–Cu–N angle is 175.09(7)°.^49,50^ The Si1–Cu1 and Cu1–N1 bond lengths are 2.194(1) and 1.903(3) Å, respectively. The Cu1–N2 bond length is 2.712(3) Å, shorter than the sum of their van der Waals radii (2.94 Å), suggesting the coordinative nature. This is also shorter than the formamidinate Cu(i) complex of CAAC (Cu–N 2.912(14) Å).^49^ The C–N bond lengths in the formamidinate backbone are different, with long N1–C13 and short N2–C13 distances of 1.337(4) and 1.294(4) Å, respectively. FMO visualisation of 19 and 20 shows that the HOMO of complex 19 shows that there is a major contribution from the 2-mesitylpyridyl moiety and d orbital of the Cu atom (Fig. S117†). The electron density at the HOMO−1 is predominantly localised over the Cu–N_amide_ bond with a minor contribution over the pyridine ring, whereas the electron density is delocalised over the Si(ii) → Cu–N–mesityl moiety in HOMO−2 with a minor contribution from the pyridine ring (Fig. S117†). However, for complex 20 the HOMO is majorly located over the formamidine moiety whereas HOMO−2 and HOMO−4 are spread over the Si(ii) → Cu bond (Fig. S117†). NBO calculation further shows that the N → Cu bond is stabilised by *E*^(2)^ values of 3.80 and 4.54 kcal mol^−1^ for 19 and 20, respectively (Fig. S122a and b†). While there could be possibilities of numerous secondary amide groups in the literature, N–H bond activation of bis-perfluoroamine is of particular interest because of its weak donation ability to the metal centre.^51^ Treatment of complex 2 with bis-pentafluorophenylamine in toluene yielded complex 21 (Scheme 5c), with a Si1–Cu1 bond length of 2.220(1) Å and a Si1–Cu1–N1 angle of 169.3(1)° (Fig. 8), deviating from linearity due to *ortho*-fluorine Cu⋯F contacts (Cu1–F10: 2.791(3) Å and Cu1–F5: 2.727(2) Å). These interactions elongate the corresponding C–F bonds [C–F5: 1.350(6) Å and C–F2: 1.358(3) Å]. Complex 21 shows the peak at *δ* 7.1 ppm for the Si(ii) centre in the ^29^Si{^1^H} NMR spectrum. As a proof of concept, the reaction of complex 7 with bis-perfluoroarylamine produces a structurally identical complex of 22 (Scheme 5c). The Si–Ag bond length in complex 22 is 2.3543(8) Å, longer than the Si–Cu bond length in complex 21. The intermolecular π-stacking between the phenyl group of the amidinate moiety and the perfluoro-substituted benzene ring further stabilises complexes 21 and 22. Also, complex 21 features intermolecular F⋯F interaction (see the ESI†). The MOs of these complexes are reminiscent of complex 18 (Fig. S118†). However, the Cu⋯F interactions in complex 21/22 are supported by the *E*^(2)^ values of 0.70/2.26 and 3.06/1.04 kcal mol^−1^ for F5 and F10, respectively (Fig. S122c and d†).

**Fig. 9:**
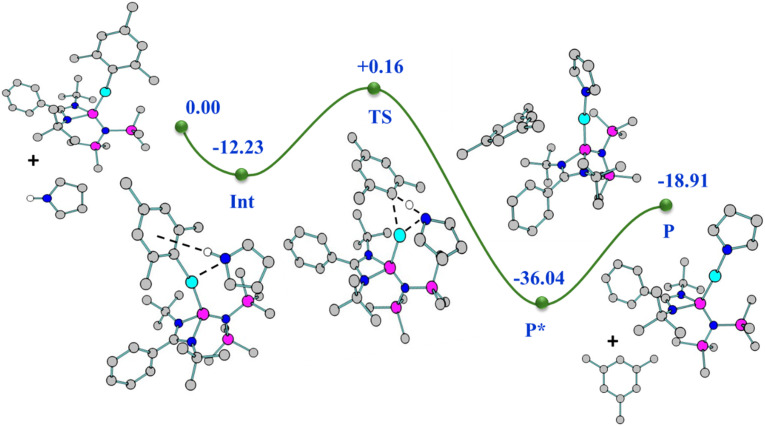
Relative energy (kcal mol^−1^) (not drawn to scale) reaction profile for the formation of complex 17 from complex 2 with pyrrole.

An apparent trend emerges after observing the chemical shifts of ^29^Si{^1^H} NMR and bonding parameters of the Cu–amide complexes (17–21) with 2 (Table 1). We observe a deshielding effect on the Si(ii) centres for complexes 17–21 that can be attributed to the electrophilic nature of the amide group. Furthermore, the Si(ii) → Cu and Cu–N_amide_ bond lengths of complexes 17–21 are shorter than the corresponding Si(ii) → Cu and Cu–C_ipso_ bond lengths in complex 2. This discrepancy suggests an enhanced electropositive character of the Cu(i) centre in complexes 17–21, as also realised from the Mulliken charge analysis (Table 2) on the Si, Cu, C_ipso_, and N_amide_. It was found that the electropositive character of the Cu(i) centre indeed increases in 17–21 with a gradual increase in the electrophilic nature of the amide ligand as compared to the mesityl group of 2. As a proof of concept, we investigated the reaction pathway of complex 17. Interestingly, the reaction profile (Fig. 9) for the formation of complex 17 shows the hydrogen transfer of the pyrrole group to the mesityl ipso carbon in the transition state (TS), which was confirmed by the imaginary frequency at 1373 cm^−1^ along these reaction coordinates. Furthermore, the covalent nature of the C_ipso_–H and N–H bonds is confirmed by the enormously strong C–H⋯N hydrogen bond in the TS with an *E*^(2)^ energy of 134.62 kcal mol^−1^ (details in the ESI†).

**Table 1:** Bond lengths of NHSi-coordinated Cu–mesityl and Cu–amide complexes

Complex	^29^Si{^1^H} chemical shift (*δ*) ppm	Si(ii) → Cu(i) bond length (Å)	Cu–C/N bond length (Å)
2	6.5	2.2509(8)	1.946(2)
17	7.8	2.231(3)	1.878(7)
18	7.4	2.203(1)	1.874(3)
19	6.9	2.1964(8)	1.906(2)
20	6.6	2.194(1)	1.903(3)
21	7.1	2.220(1)	1.929(4)

**Table 2:** Mulliken charges on Si(ii), Cu(i), C_ipso_, and N_amide_ centres

	2	17	18	19	20	21	22
*q*_Si_	0.617	0.830	0.793	0.857	0.837	0.820	0.599
*q*_M_	−0.474	−0.342	−0.193	−0.214	−0.203	−0.227	−0.060
*q*_C/N_	−0.183	−0.115	−0.209	−0.248	−0.240	−0.239	−0.354

#### 2.2.4 Syntheses of NHSi-based Cu(i) and Ag(i) phosphide complexes (23–24) *via* P–Si bond cleavage

**Chart 3:**
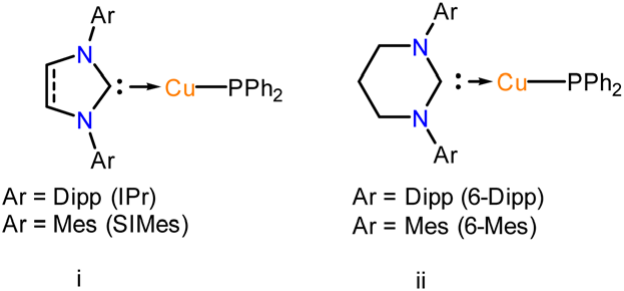
Structurally defined monomeric NHC → Cu–PPh_2_ complexes reported in the literature.

**Scheme 6:**
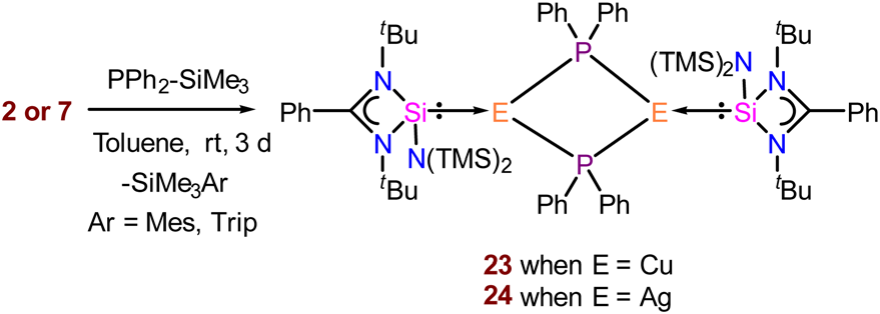
Syntheses of NHSi-coordinated metal phosphide complexes from 2 and 7.

Low-coordinate coinage metal phosphide complexes stabilised by electron-donating neutral ligands are rare due to their propensity to form oligomers.^52^ This is also reflected by the availability of the limited examples of structurally characterised NHC-coordinated Cu(i)–PR_2_ species in the literature (Chart 3).^8*i*^ The metal centre must have at least one vacant frontier orbital with suitable symmetry to accept π-donation from the p-character lone pair of the phosphide ligand to form an E–PR_2_ type bond. However, late transition metals like Cu and Ag, with their higher d-electron counts, are more susceptible to interactions between their filled metal orbitals and the phosphorus lone pair. Such interactions often induce a pyramidal geometry at the phosphorus atom. Thus, understanding their bonding situation is an important aspect. To our knowledge, low-coordinate Ag(i)–PR_2_ complexes are still structurally unknown. However, polynuclear [Ag_12_(PSiMe_3_)_6_(^i^Pr_2_-bimy)_6_] and [Ag_26_P_2_(PSiMe_3_)_10_(^i^Pr_2_-bimy)_8_] were isolated.^53^ Thus, stabilisation of such coinage metal phosphides in low-nuclearity is challenging. This inspired us to proceed with NHSi → Cu/Ag–PR_2_ isolation with complexes 2 and 7 as suitable precursors for the safer handling of Ph_2_P–SiMe_3_. Adding one equivalent of PPh_2_–SiMe_3_ to the pale-yellow solution of complexes 2 and 7 resulted in an instantaneous cloudy solution (Scheme 6). Stirring the resulting solution for 2 days, we could isolate complexes 23 and 24 with ∼30% yield. The two sets of doublets at *δ* 2.80–3.23 and 9.30–8.87 ppm in ^29^Si{^1^H} NMR indicate the Si(ii)–Ag centre in complex 24. ^31^P{^1^H} NMR shows the peak at *δ* −13.3 ppm for complex 24.

**Fig. 10:**
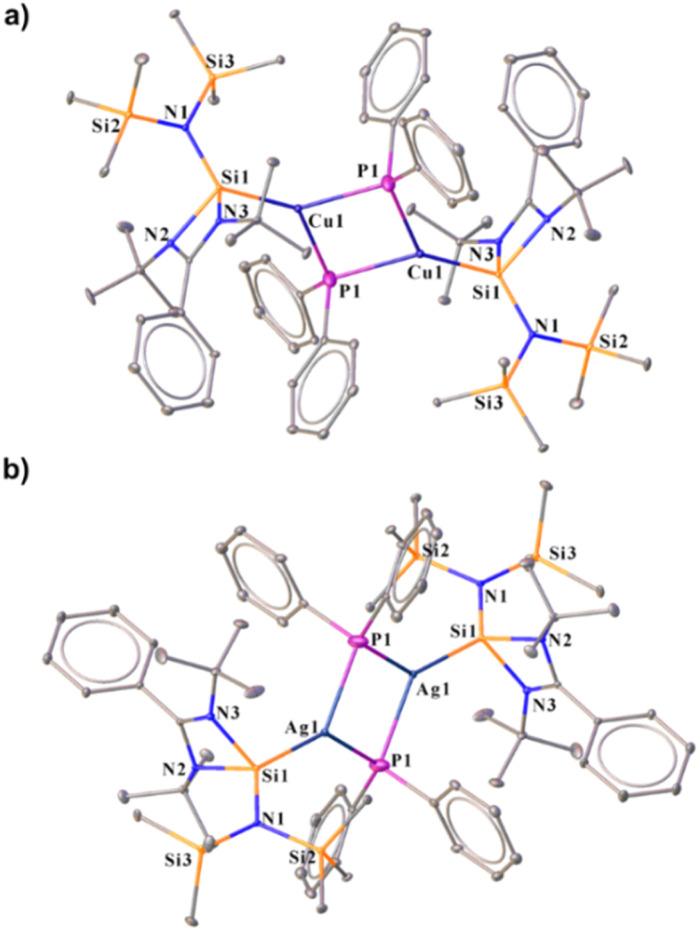
Molecular structures of complexes 23 (a) and 24 (b). The bond distance (Å) and bond angle (°) are discussed in the ESI.†

**Fig. 11:**
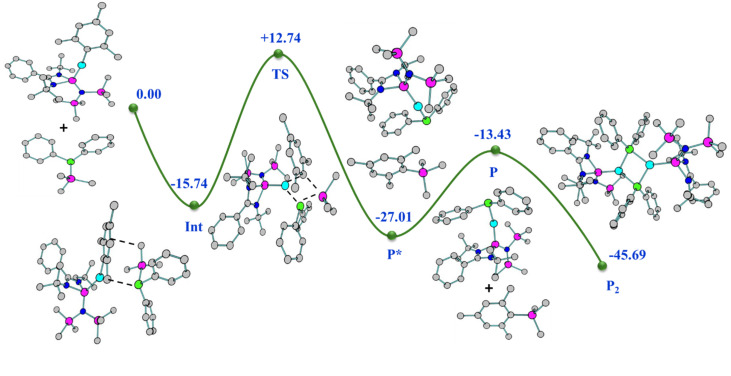
Relative energy (kcal mol^−1^) (not drawn to scale) reaction profile for the formation of complex 23 from complex 2 with PPh_2_SiMe_3_.

The molecular structures of both complexes reveal a dimeric μ-bridged Cu(i)/Ag(i)–PPh_2_ core coordinated with two NHSi moieties from each end (Fig. 10). The formation of 23 and 24 resulted from eliminating Mes–SiMe_3_ (for 23) and Trip–SiMe_3_ (for 24). However, these eliminations are not very facile under ambient conditions, hence dropping the yield of the desired product. The dimeric core represents a near rhomboid geometry with bond angles for 23 [P1–Cu1–P1 86.16° and Si1–Cu1–P1 136.98°] and 24 [P1–Ag1–P1 84.48° and Si1–Ag1–P1 137.49°]. The Si–Cu(i)/Ag(i) bond distances are 2.2758(8) and 2.401(1) Å for 23 and 24, respectively, which are longer than that in the NHC → Cu–PR_2_ complexes [1.9272(18) Å for the monomeric (6-Dipp)CuPPh_2_ complex and 1.9234(15) Å for the dimeric IDippCuPPh_2_ complex] reported in the literature.^8*i*^ The Cu–P distance [2.3799(8) Å] in complex 23, is longer than that in the monomeric (6-Dipp)CuPPh_2_ complex [2.2113(5) Å] and is in line with that of the dimeric IDippCuPPh_2_ complex [2.3298(5) Å].^8*h*,*i*,54^ The Ag–P bond distance [2.537(1) Å] in complex 20 is in line with the Ag–P bond distances reported in [Ag_12_(PSiMe_3_)_6_(^*i*^Pr_2_-bimy)_6_] and [Ag_26_P_2_(PSiMe_3_)_10_(^*i*^Pr_2_-bimy)_8_] [2.579(3) and 2.605(5) Å].^53^ The intermetallic distances (E⋯E) are significantly longer [3.458 Å (complex 23) and 3.791 Å (complex 24)], suggesting the absence of metallophilic interactions in these complexes, unlike those in complex 14. Since such low-coordinate Cu(i)–phosphide complexes are limited only to their structure elucidation, it became customary for us to understand the electronic structures of complexes 23 and 24 using FMO visualisation. The HOMO and HOMO−1 of both complexes were composed of the Cu/Ag–P bonding interaction with d–p orbital overlap, whereas HOMO−4 resides over Si(ii)–Cu/Ag(i) bonds (Fig. S119†). Isolation and characterisation of NHSi → coinage metal–phosphides are the first of their kind, thus indicating future possibilities of fine-tuning and utilisation in homogeneous catalysis. The reaction pathway shows that C–H⋯π and C–H⋯P hydrogen bonding stabilises the Int observed in the reaction profile (Fig. 11) for the formation of complex 23. The strengthening of the interaction between P and Cu centers in the TS suggests the bond formation between P⋯Cu centers. Apart from their brief mention here, a complete discussion of the computed pathways, including energetic profiles and structural details, is provided in the revised ESI (pages S102–S110†). We performed detailed DFT calculations on four representative complexes of each bond type among 12–24, as already mentioned. These complexes were selected to reflect the four distinct types of bond activations investigated: homolytic B–B (12) and Se–Se (14), and heterolytic N–H (17) and P–Si (23) bond activations. We believe the selected cases will provide insight into the general reactivity patterns. All the mechanistic pathways show a similar fashion of nucleophilic attack of the reactant to the Cu(i) centre in the transition states, followed by the mesityl group migration to the electrophilic substituent, affording the desired complexes.

### 2.3 The catalytic utility of NHSi-organo-coinage metal complexes towards different organic transformations

The examples of the NHSi ligated transition metal mediated catalysis are limited to a handful of catalytic reactions.^55^ Recently, our group has demonstrated the scope of NHSi ligated coinage metal complexes in a few different organic reactions, like azide–alkyne cycloaddition,^6*a*,7*e*,47^ glycosidation,^7*f*^ and three-component coupling reactions.^7*d*^ Motivated by our previous results on catalysis, we aimed to probe the catalytic performances of the isolated NHSi–organocoinage metal complexes. Our brief attempt at these complexes towards catalysing organic transformations is equally promising.

#### 2.3.1 Use of NHSi–organocopper complexes as an aryl transfer agent in the C–C coupling reaction

**Chart 4:**
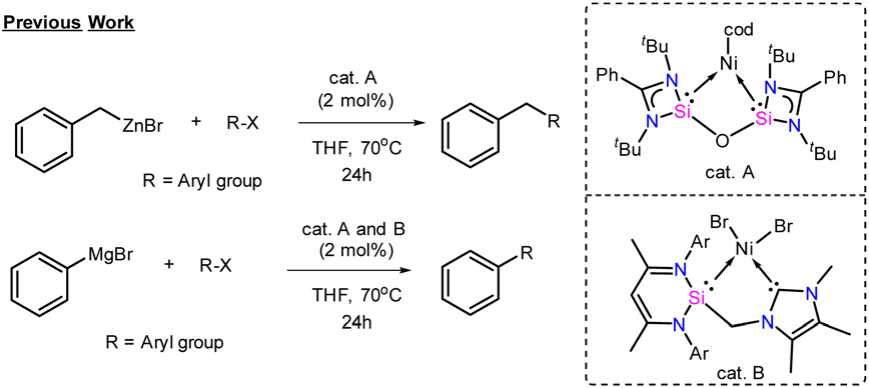
Previous reports on C–C coupling reactions with NHSi as a ligand on the metal centre. (cod = cyclooctadiene).

Over the years, the prodigious use of aryl boronic esters and aryl magnesium precursors has dominated the area of C–C cross-coupling reactions.^56^ A recent report by Uchiyama and co-workers has shown the applicability of aryl copper in Pd-catalysed C–C coupling with sterically demanding substrates.^57^ Moreover, there are only two reports where NHSis is used as a ligand (catalysts A and B, Chart 4) for sp^3^–sp^2^ and sp^2^–sp^2^ C–C coupling reactions.^58^

**Scheme 7:**
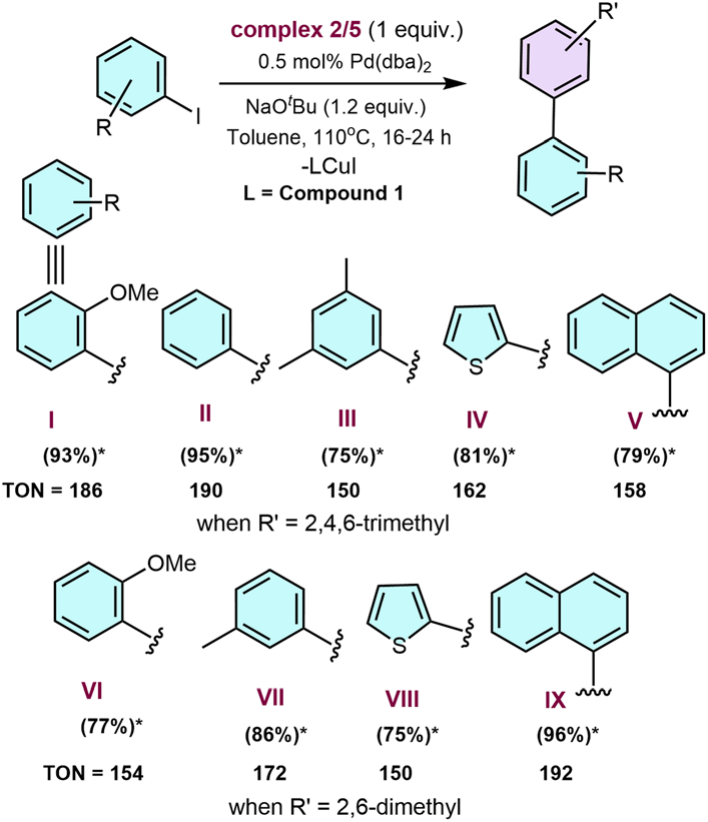
Pd-catalysed sp^2^–sp^2^ C–C coupling reaction using 2 and 5 as an aryl transfer reagent. *Isolated yields.

Since we already had well-characterised NHSi–Cu–aryl systems (2 and 5), we utilised them as an efficient aryl group transfer agent for Pd-catalysed C–C coupling reactions with aryl iodides (Scheme 7).

The C–C coupling reactions with a few aryl iodides were carried out under the optimised conditions with complex 2. We observed good to excellent product yields for the heteroaromatic (IV and VIII) and fused aromatic (V and IX) systems (Scheme 7). Using the NHSi coordinated organo–copper complex as an alternative to conventional aryl boronic esters provides a lower Pd catalyst loading (0.5 mol%) compared to the conventional C–C coupling reactions.^59^ It is important to note that the Pd catalyst loading (5 mol%) for the C–C coupling reaction by Uchiyama and co-workers remains much higher with the use of additional ligand loading (up to 15 mol%) (Table 3).

**Table 3:** Pd-catalysed C–C coupling reaction using mesityl copper

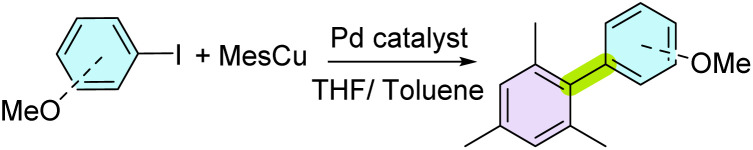				
Serial no.	Pd-based catalyst (mol%)	Temperature (°C)	Yield (%)	TON
1	5	80–140	91 (ref. 57)	18.2
This work	0.5	110	93	186

The TON for the formation of compound I is observed to be 186 with our catalytic method, whereas it is 18.2 by utilising the method of Uchiyama and co-workers (Table 3). Hence, our method utilising 2 and 5 as the aryl transfer reagent works much more efficiently, leading to scope for future exploration of more NHSi-coordinated organocopper compounds in various other organic transformations. This enhanced catalytic performance is presumably due to the strong σ-donor properties of NHSi, which are also observed in other previous reports.^1*c*^

#### 2.3.2 Use of NHSi–organosilver complexes as catalysts for three-component reactions for various α-aminonitrile synthesis

**Scheme 8:**
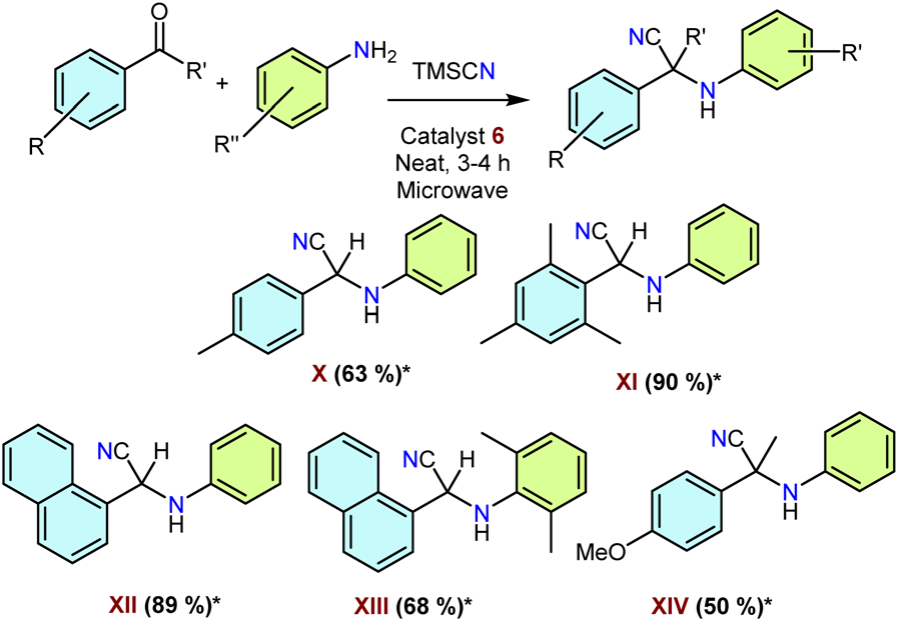
General schematic for NHSi–organosilver catalysed α-aminonitrile synthesis. *Isolated yields are reported after taking an average of three runs. ^#^ Catalyst loading 5 mol%.

Following our success in C–C coupling reactions with the NHSi–organocopper complexes mentioned above, we wanted to explore the catalytic performance of the organosilver analogues as well. We chose the one-pot synthesis of α-aminonitriles as these are an important class of bioactive molecules, and the Strecker reaction is one of the convenient methods to synthesise them.^60^ Until today, these reactions are catalysed by a large excess of Lewis acids as catalysts under vigorous reaction conditions. Thus, we opted for microwave-assisted α-aminonitrile preparation for our purpose. We optimised the best-performing reaction conditions with 5 mol% loading of catalyst 6 at 80 °C for 3 h under solvent-free conditions (Scheme 8). We obtained good to excellent yields as documented in Scheme 8. The future scope can be extended to the number of substrates with specific utility. This short study highlights the potential of these organo–silver complexes for such important catalytic transformations.

## 3 Conclusions

In summary, we have established a versatile route to utilise the NHSi–organocoinage metal complexes as an efficient precursor for heteroatom functionalisation. The derivatisation of NHC or CAAC-based coinage metal complexes has become important lately because of the efficient optoelectronic applications of carbene–metal–amide complexes and several organic transformations. However, the functionalisation of NHSi–Cu(i) and Ag(i) complexes was unknown to date. Our adopted methodology allows facile formation of NHSi-supported Cu–B (12), Cu/Ag–Se (14–16), Cu/Ag–N (17–22), and Cu/Ag–P (23 and 24) bonds. Moreover, utilising N-donor attached secondary amines gives three-coordinate copper(i) complexes (21 and 22). This synthetic protocol helped us to isolate rarely occurring coinage metal phosphides, 23 and 24. We have also demonstrated the potential of these NHSi–organocoinage metal complexes in two important catalytic organic transformations, which led to the isolation of desired products in excellent yields, paving the way for more exploration in this area.

## Author contributions

S. K. and M. G. conceived the idea of the project. M. G. contributed to the design, development, execution, and compilation of the project. M. G. performed all the syntheses and characterisation studies and wrote the original draft with the help of P. P. P. P. performed all the theoretical calculations, prepared the corresponding figures, and provided insightful feedback. K. G. carried out catalytic reactions and curated spectroscopic details in the ESI,† and prepared the figures of characterisation methods along with M. G. S. T. refined the crystallographic data provided by M. G. R. K. P. put significant efforts into refining additional crystal data during the revision. All authors contributed to the discussion. S. K. contributed to fund acquisition, project administration, offered critical insights, coordinated the research, and finalised the manuscript.

## Conflicts of interest

There are no conflicts to declare.

## Supplementary Material

SC-OLF-D5SC00879D-s001

SC-OLF-D5SC00879D-s002

## Data Availability

The data supporting this article have been included as part of the ESI.† The ESI contains experimental data, X-ray data, NMR spectra, computational details, and related references.
