# Supplementary material for: Driving diverse bond functionalisation with N-heterocyclic silylene-coinage metal–aryl complexes
Source: Chem Sci. 2025 Jun 30;16(32):14518–33. doi: 10.1039/d5sc00879d (PMC12264977; doi:10.1039/d5sc00879d)
Supplement: SC-016-D5SC00879D-s001 [file SC-016-D5SC00879D-s001.pdf]

Supplementary Information (SI) for Chemical Science.
This journal is © The Royal Society of Chemistry 2025

Supporting Information

for

Driving Diverse Bond Functionalisation with N-Heterocyclic Silylene-Coinage Metal-Aryl Complexes

Moushakhi Ghosh[a], Kumar Gaurav[a†], Prakash Panwaria[a†], Rishukumar Panday[a], Srinu Tothadi[b] and Shabana Khan[a*]

[a]Department of Chemistry, Indian Institute of Science Education and Research Pune, Dr. Homi Bhabha Road, Pashan, Pune-411008, India.

[b]Analytical and Environmental Sciences Division and Centralized Instrumentation Facility, CSIR-Central Salt and Marine Chemicals Research Institute, Gijubhai Badheka Marg, Bhavnagar-364002, India.

†Both authors contributed equally

*Corresponding author E-mail: shabana@iiserpune.ac.in

Content

S1. Experimental Section

S1.1. Synthesis of Complexes **2-24**

S1.2. C-C Cross-Coupling Reactions

S1.3. *α*-amino-benzyl nitriles synthesis

S2 NMR Spectra

S3. Crystallographic Details

S4. Molecular Structure of Complexes $Ph(N^tBu)_2SiNDippSiMe_3$

S5. Computational Section

S6. Metrical Parameters of Isolated Complexes **2-24**

S7. Reactivity of complex **2** with B_2pin_2

S1. Experimental Section

All experiments were conducted under the inert gas atmosphere of dinitrogen (N_2) or argon (Ar) in oven-dried glassware using standard Schlenk techniques and in a dinitrogen-filled MBRAUN MB 150-G1 glove box. The solvents used were purified by an MBRAUN solvent purification system, MB SPS-800. All chemicals purchased from Sigma-Aldrich and Alfa-Aesar were used without further purification. Some reagents were purchased from other commercial sources and used without further purification unless otherwise stated.

^{1}H, ^{13}C, ^{29}Si, and ^{19}F NMR spectra were measured in $CDCl_3$, C_6D_6 using a 400 MHz NMR spectrometer, "Bruker Avance DRX500"; chemical shifts (δ) are expressed in ppm referenced to external $SiMe_4$ (tetramethylsilane, TMS), ^{77}Se was referenced to external Me_2Se, using the residual solvent as internal standard ($CDCl_3$: ^{1}H, 7.26 ppm and ^{13}C, 77.16 ppm; C_6D_6: ^{1}H, 7.16 ppm and ^{13}C, 128.06 ppm). Coupling constants are expressed in hertz. Individual peaks are reported as multiplicities (s = singlet, d = doublet, dd = doublet of doublet, t = triplet, q = quartet, m = multiplet, integration coupling constants are given in Hz. The spectra were processed and analysed using *MestReNova* software. Melting points were measured in a sealed glass tube and were not corrected. Compound **1** was prepared *via* previously reported procedures.[1] According to the literature procedure, mesityl copper, 2,4,6-triisopropylphenyl copper, 2,6-diisopropylphenyl copper, mesityl silver, 2,4,6-triisopropylphenyl silver, and 2,6-diisopropylphenyl silver were also prepared.[2] Diphenyl(trimethylsilyl)phosphine [PPh_2SiMe_3] was also prepared *via* the literature procedure.[3] LSiCl and LSiO^{t}Bu were also prepared *via* the literature procedure.[1]

S1.1. Synthesis of Complexes 2-24

❖ Preparation of **2**: A Schlenk flask charged with compound **1** (210.42 mg, 0.5 mmol) and mesityl copper (91.37 mg, 0.5 mmol), followed by the addition of toluene (20 ml) and kept in stirring overnight under an inert atmosphere of Argon (Ar). The greenish-yellow colour solution was filtered and evaporated under reduced pressure. Colourless single crystals were grown in toluene at 0 °C after keeping it overnight. Yield: 250 mg (83%). ^{1}H NMR (400 MHz, C_6D_6, 298 K) δ 0.32 (s, 9H, Si-CH_3), 0.70 (s, 9H, Si-CH_3), 1.17 (s, 18H, {N-C(CH_3)$_3$}, 2.44 (s, 3H, *p*-CH_3), 3.06 (s, 6H, *o*-CH_3), 6.85-7.03 (m, 5H, arom. H), 7.14 (d, 2H, J = 8.2 Hz, arom. H) ppm; $^{13}C\{^{1}H\}$ NMR (100.613 MHz, C_6D_6, 298 K): δ 4.82 (Si-*C*H$_3$), 5.98 (Si-*C*H$_3$), 21.78 (mesityl *C*H$_3$), 29.86 (mesityl *C*H$_3$), 31.80 {N-C(*C*H$_3$)$_3$}, 54.36 {N-*C*(CH$_3$)$_3$}, 125.16, 130.29, 132.03, 133.40, 146.66 (arom. C), 165.93, 167.16 (N*C*N)

ppm; $^{29}Si\{^1H\}$ NMR (79.49 MHz, C_6D_6, 298 K): δ 5.3 [-N(SiMe$_3$)$_2$], 5.3 [-N(SiMe$_3$)$_2$], 6.5 (Si-Cu) ppm. HRMS (ESI, CH_3CN) (m/z) for [$C_{30}H_{52}CuN_3Si_3$] calc. 601.2765; found 601.2748 [M^+].

- <u>Preparation of **3**:</u> A Schlenk flask charged with compound **1** (210.42 mg, 0.5 mmol) and 2,4,6-triisopropylphenyl copper (133.45 mg, 0.5 mmol), followed by the addition of toluene (20 ml) and kept in stirring overnight under an inert atmosphere of Argon (Ar). The greenish-yellow colour solution was filtered and evaporated under reduced pressure. Colourless single crystals were grown in toluene at room temperature after being kept overnight. Yield: 300 mg (87.2 %). ^1H NMR (400 MHz, C_6D_6, 298 K) δ 0.24 (s, 9H, Si-CH_3), 0.60 (s, 9H, Si-CH_3), 1.10 (s, 18H, {N-C(CH_3)$_3$}), 1.38 (d, 6H, J = 6.9 Hz, p-CHCH_3), 1.57 (d, 12H, J = 6.9 Hz, o-CHCH_3), 2.97 (sept, 1H, J = 6.9 Hz, p-CHCH$_3$), 3.74 (sept, 2H, J = 6.9 Hz, o-CHCH$_3$), 6.80 (m, 3H, Ar-H), 6.93 (m, 1H, Ar-H), 7.06 (m, 3H, Ar-H) ppm; $^{13}C\{^1H\}$ NMR (100.613 MHz, C_6D_6, 298 K): δ 4.93 (Si-CH$_3$), 5.96 (Si-CH$_3$), 24.46 {-CH(CH$_3$)$_2$}, 26.42 {-CH(CH$_3$)$_2$}, 31.81 {N-C(CH$_3$)$_3$}, 35.37 {N-C(CH$_3$)$_3$}, 42.23 {C-(CH$_3$)$_2$}, 54.41 {N-C(CH$_3$)$_3$}, 118.71, 122.43, 125.70, 129.34, 130.38, 132.10, 145.40, 158.52, (arom. C), 165.65, 166.84 (NCN) ppm; $^{29}Si\{^1H\}$ NMR (79.49 MHz, C_6D_6, 298 K): δ 4.9 [-N(SiMe$_3$)$_2$], 5.4 [-N(SiMe$_3$)$_2$], 6.6 (Si-Cu) ppm. HRMS (ESI, CH_3CN) (m/z) for [$C_{36}H_{64}CuN_3Si_3$] calc. 685.3704; found 708.1825 $[M+Na]^+$. M.p. 178.0 °C.
- <u>Preparation of **4**:</u> A Schlenk flask charged with compound **1** (210.42 mg, 0.5 mmol) and 2, 6-diisopropylphenyl copper (112 mg, 0.5 mmol), followed by the addition of toluene (20 ml) and kept in stirring overnight under an inert atmosphere of Argon (Ar). The greenish-yellow colour solution was filtered and evaporated under reduced pressure. Colourless single crystals were grown in toluene at room temperature after being kept overnight. Yield: 200 mg (62 %). ^1H NMR (400 MHz, C_6D_6, 298 K) δ 0.33 (s, 9H, Si-CH_3), 0.69 (s, 9H, Si-CH_3), 1.19 (s, 18H, {N-C(CH_3)$_3$}), 1.63-1.65 (d, 12H, J = 6.9 Hz, ^{i}Pr-CH_3), 3.82 (sept, 2H, J = 6.9 Hz, CHCH$_3$), 6.89-6.92 (m, 3H, Ar-H), 7.10-7.12 (m, 2H, Ar-H), 7.36-7.38 (m, 2H, Ar-H), 7.46-7.48 (m, 1H, Ar-H) ppm; $^{13}C\{^1H\}$ NMR (100.613 MHz, C_6D_6, 298 K): δ 4.91 (Si-CH$_3$), 5.96 (Si-CH$_3$), 24.32 {-CH(CH$_3$)$_2$}, 26.31 {-CH(CH$_3$)$_2$}, 29.20 {N-C(CH$_3$)$_3$}, 31.80 {N-C(CH$_3$)$_3$}, 33.01 {C-(CH$_3$)$_2$}, 34.65 {C-(CH$_3$)$_2$}, 54.41 {N-C(CH$_3$)$_3$}, 120.60, 124.18, 127.94, 128.17, 128.30, 128.57, 128.82, 129.33, 130.37, 149.03 (Ar-C), 158.45 (NCN) ppm; $^{29}Si\{^1H\}$ NMR (79.49 MHz, C_6D_6, 298 K): δ 4.9 [-N(SiMe$_3$)$_2$], 5.4 [-N(SiMe$_3$)$_2$], 6.6 (Si-Cu) ppm. HRMS (ESI, CH_3CN) (m/z) for [$C_{33}H_{58}CuN_3Si_3$] calc. 643.3235; found 644.3824 $[M+H]^+$. M.p. 166.0 °C.

❖ Preparation of **5**: A Schlenk flask charged with compound **1** (210.42 mg, 0.5 mmol) and 2, 6-dimethylphenyl copper (84 mg, 0.5 mmol), followed by the addition of toluene (20 ml) and kept in stirring overnight under an inert atmosphere of Argon (Ar). The greenish-yellow colour solution was filtered and evaporated under reduced pressure. Colourless single crystals were grown in toluene at room temperature after being kept overnight. Yield: 200 (68 %). ^{1}H NMR (400 MHz, C_6D_6, 298 K) δ 7.36 – 7.29 (m, 3H, Ar-H), 7.13 (dd, J = 8.7, 3.0 Hz, 2H, Ar-H), 7.03 (dd, J = 16.7, 7.9 Hz, 1H, Ar-H), 6.94 (dd, J = 6.6, 5.3 Hz, 2H, Ar-H), 3.06 (s, 6H, *o*-C*H*$_3$), 1.16 (s, 18H, {N-C(C*H*$_3$)$_3$}), 0.69 (s, 9H, Si-C*H*$_3$), 0.32 (s, 9H, Si-C*H*$_3$) ppm; ^{13}C NMR (101 MHz, C_6D_6) δ 170.11 (N*C*N), 167.21 (arom. C), 146.61 (arom. C), 131.99 (arom. C), 130.28 (arom. C), 129.33 (arom. C), 126.45 (arom. C), 125.70 (arom. C), 125.27 (arom. C), 124.04 (arom. C), 54.36 ({-N*C*(CH$_3$}$_3$), 33.00 (*o*-*C*H$_3$), 31.79 (*o*-*C*H$_3$), 30.01 ({-NC(*C*H$_3$}$_3$), 5.97 (Si-*C*H$_3$), 4.81 (Si-*C*H$_3$) ppm; ^{29}Si{^{1}H} NMR (79.49 MHz, C_6D_6, 298 K): δ 5.4 [-N(*Si*Me$_3$)$_2$], 6.6 (*Si*-Cu) ppm. HRMS (ESI, CH_3CN) (*m/z*) for [$C_{29}H_{50}CuN_3Si_3$] calc. 587.2609; found 586.2797 [M-H]$^+$. M.p. 77.6 °C.

❖ Preparation of **6**: A Schlenk flask charged with compound **1** (211 mg, 0.5 mmol) and mesityl silver (114 mg, 0.5 mmol), followed by the addition of toluene (20 ml) and kept in stirring overnight under an inert atmosphere of Argon (Ar). The resultant solution was filtered and evaporated under reduced pressure, which afforded colourless single crystals at room temperature. Yield: 350 mg (80 %). ^{1}H NMR (400 MHz, C_6D_6, 298 K) δ 0.31 (s, 9H, Si-C*H*$_3$), 0.66 (s, 9H, Si-C*H*$_3$), 1.16 (s, 18H, {N-C(C*H*$_3$)$_3$}), 2.45 (s, 3H, *o*-C*H*$_3$), 3.02 (s, 6H, *p*-C*H*$_3$), 6.88 (m, 3H, arom. H), 7.00 (d, 1H, *J* = 7.4 Hz, arom. H), 7.12 (d, 1H, *J* = 7.6 Hz, arom. H), 7.21 (s, 2H, arom. H) ppm; ^{13}C{^{1}H} NMR (100.613 MHz, C_6D_6, 298 K): δ 4.89 (Si-*C*H$_3$), 5.92 (Si-*C*H$_3$), 14.35 (Mes-*C*H$_3$), 21.75 (mesityl *C*H$_3$), 23.06 (mesityl *C*H$_3$), 31.86 (N-C*C*H$_3$), 54.48 {N-*C*(CH$_3$)$_3$}, 125.11, 130.35, 131.98, 133.40, 146.16 (arom. C), 166.18 (NCN) ppm; ^{29}Si{^{1}H} NMR (79.49 MHz, C_6D_6, 298 K): δ 6.1-6.2 (d, [-N(*Si*Me$_3$)$_2$] *J*= 5.9 Hz), 6.9[-N(*Si*Me$_3$)$_2$], 11.2-11.5, 14.5-14.7 (two sets of d, *Si*-Ag, ^{1}J ^{29}Si to ^{109}Ag and ^{107}Ag = 257.65, 255.267 Hz) ppm. HRMS (ESI, CH_3CN) (*m/z*) for [$C_{39}H_{63}Ag_2N_3Si_3$] calc. 871.2432; found 872.2076 [M+H]$^+$. M.p. 212.5 °C.

❖ Preparation of **7**: A Schlenk flask charged with compound **1** (210.42 mg, 0.5 mmol) and 2, 4, 6-triisopropylphenyl silver (155 mg, 0.5 mmol), followed by the addition of toluene (20 ml) and kept in stirring overnight under an inert atmosphere of Argon (Ar). The greenish-yellow colour solution was filtered and evaporated under reduced pressure. Colourless single crystals were grown in toluene at room temperature after being kept overnight. Yield:

250 mg (68 %). ^{1}H NMR (400 MHz, C_6D_6, 298 K) δ 0.19 (s, 9H, Si-C*H$_3$*), 0.54 (s, 9H, Si-C*H$_3$*), 1.04 (s, 18H, {N-C(C*H$_3$*)$_3$}), 1.34-1.36 (d, 6H, J = 6.9 Hz, *p*-CHC*H$_3$*), 1.52-1.54 (d, 12H, J = 7.0 Hz, *o*-CHC*H$_3$*), 2.96 (sept, 1H, J = 6.9 Hz, *p*-C*H*CH$_3$), 3.58 (sept, 2H, J = 6.9 Hz, *o*-C*H*CH$_3$), 6.78 (m, 3H, Ar-H), 6.99 (m, 1H, Ar-H), 7.02 (m, 3H, Ar-H) ppm; ^{13}C{^{1}H} NMR (100.613 MHz, C_6D_6, 298 K): δ 4.94 (Si-*C*H$_3$), 5.87 (Si-*C*H$_3$), 25.19 {-CH(*C*H$_3$)$_2$}, 26.13 {-CH(*C*H$_3$)$_2$}, 31.88 {N-C(*C*H$_3$)$_3$}, 35.39 {N-C(*C*H$_3$)$_3$}, 43.08 {*C*-(CH$_3$)$_2$}, 54.55 {N-*C*(CH$_3$)$_3$}, 118.78, 122.43, 127.82, 128.30, 130.45, 132.01, 145.39, 157.39, (Ar-*C*), 165.90 (N*C*N) ppm; ^{29}Si{^{1}H} NMR (79.49 MHz, C_6D_6, 298 K): δ 6.2-6.3(d, [-N(*Si*Me$_3$)$_2$], *J*= 5.9 Hz), 6.9 [-N(*Si*Me$_3$)$_2$], 11.0-10.8, 14.0-14.2 (two set of d, *Si*-Ag, ^{1}J ^{29}Si to ^{109}Ag and ^{107}Ag = 257.65, 255.267 Hz) ppm. HRMS (ESI, CH_3CN) (*m/z*) for [$C_{36}H_{64}AgN_3Si_3$] calc. 729.3459; found 729.3184 [M]$^+$. M.p. 196.7 °C.

- <u>Preparation of **8**:</u> A Schlenk flask charged with compound **1** (210.42 mg, 0.5 mmol) and 2, 6-diisopropylphenyl silver (134 mg, 0.5 mmol), followed by the addition of toluene (20 ml) and kept in stirring overnight under an inert atmosphere of Argon (Ar). The greenish-yellow colour solution was filtered and evaporated under reduced pressure. The concentrated solution afforded colourless single crystals at room temperature. Yield: 210 mg (61 %). ^{1}H NMR (400 MHz, C_6D_6, 298 K) δ 0.32 (s, 9H, Si-C*H$_3$*), 0.67 (s, 9H, Si-C*H$_3$*), 1.17 (s, 18H, {N-C(C*H$_3$*)$_3$}), 1.63-1.65 (d, 12H, J = 6.9 Hz, ^{i}Pr-C*H$_3$*), 3.70 (sept, 2H, J = 6.9 Hz, C*H*CH$_3$), 6.88-6.97 (m, 3H, Ar-H), 7.13-7.15 (m, 2H, Ar-H), 7.40-7.42 (m, 2H, Ar-*H*), 7.47-7.50 (m, 1H, Ar-H) ppm; ^{13}C{^{1}H} NMR (100.613 MHz, C_6D_6, 298 K): δ 4.93 (Si-*C*H$_3$), 5.89 (Si-*C*H$_3$), 26.04 {-CH(*C*H$_3$)$_2$}, 31.87 {-CH(*C*H$_3$), 43.16 {*C*-(CH$_3$)$_2$}, 54.56 {N-*C*(CH$_3$)$_3$}, 120.65, 125.75, 127.82, 128.30, 130.46, 131.93, 157.35 (Ar-*C*), 166.04 (N*C*N) ppm; ^{29}Si{^{1}H} NMR (79.49 MHz, C_6D_6, 298 K): δ 6.2-6.3 (d, [-N(*Si*Me$_3$)$_2$], *J*= 6.3 Hz), 6.9 [-N(*Si*Me$_3$)$_2$], 10.75, 13.91-14.11 (d, *Si*-Ag, J = 16.4 Hz) ppm. HRMS (ESI, CH_3CN) (*m/z*) for [$C_{33}H_{58}AgN_3Si_3$] calc. 687.2989; found 726.1973 [M+K]$^+$. M.p. 195.6 °C.
- <u>Preparation of **9**</u>: A Schlenk flask charged with LSiCl (189 mg, 0.5 mmol) and mesityl copper (91.37 mg, 0.5 mmol) was taken in 20 mL toluene and kept in stirring overnight under an inert atmosphere of Argon (Ar). The greenish-yellow solution was evaporated under reduced pressure, extracted in 20 mL DCM, and filtered off. Concentrating DCM to 2 mL and adding 4 mL *n*-Pentane produced colourless single crystals at room temperature. Yield: 410 mg (86 %). ^{1}H NMR (400 MHz, C_6D_6, 298 K) δ 1.16 (s, 18H, {N-C(C*H$_3$*)$_3$}), 2.23 (s, 3H, mesityl-C*H$_3$*), 2.54 (s, 3H, mesityl-C*H$_3$*), 3.13 (s, 3H, mesityl-C*H$_3$*), 6.78-6.82 (m, 2H, Ar-H), 7.38-7.42 (m, 2H, Ar-H), 7.51 (m, 3H, Ar-H) ppm; ^{13}C{^{1}H} NMR (100.613

MHz, C_6D_6, 298 K): δ 20.36 (N-C*C*H_3), 21.17 (mesityl *C*H_3), 27.34 (mesityl *C*H_3), 31.26 (N-C*C*H_3), 54.41 {N-*C*(CH_3)$_3$}, 127.64, 127.94,128.27, 129.61, 130.07, 130.87, 131.69, 141.08, 145.15, 147.52, 167.07 (N*C*N) ppm; ^{29}Si{^{1}H} NMR (79.49 MHz, C_6D_6, 298 K): δ 32.7 (*Si*-Cu) ppm. HRMS (ESI, CH_3CN) (*m/z*) for [$C_{48}H_{68}C_{12}Cu_2N_4Si_2$] calc. 952.2952; found 953.1852 $[M+H]^+$. M.p. 279.6 °C.

- Preparation of **10**: A Schlenk flask charged with LSiO^{t}Bu (167 mg, 0.5 mmol) and mesityl copper (91.37 mg, 0.5 mmol) was taken in 20 mL toluene and kept in stirring overnight under an inert atmosphere of Argon (Ar). The bright-yellow solution was filtered and evaporated under reduced pressure. Concentrating toluene to 2 mL and adding 4 mL *n*-Pentane produced yellow-coloured single crystals at room temperature. Yield: 350 mg (68 %). ^{1}H NMR (400 MHz, C_6D_6, 298 K) δ 6.91 – 6.84 (m, 3H, Ar-H), 6.82 – 6.76 (m, 2H, Ar-H), 3.08 (s, 6H, *o*-C*H*$_3$), 2.45 (s, 3H, *p*-C*H*$_3$), 1.53 (s, 9H, -OC(C*H*$_3$)$_3$), 1.10 (s, 18H, {N-C(C*H*$_3$)$_3$}) ppm; ^{13}C NMR (101 MHz, C_6D_6) δ 171.0 (N*C*N), 146.91 (arom. C), 130.15 (arom. C), 128.89 (arom. C), 127.82 (arom. C), 125.15 (arom. C), 74.14 {OC(C*H*$_3$)$_3$}, 53.78 (({N-*C*(CH_3)$_3$}), 32.89 {-OC(*C*H_3)$_3$}, 31.59 (mesityl C), 29.61 (mesityl C), 21.82 (({N-C(*C*H_3)$_3$}) ppm; ^{29}Si{^{1}H} NMR (79.49 MHz, C_6D_6, 298 K): δ 3.8 (*Si*-Cu) ppm. HRMS (ESI, CH_3CN) (*m/z*) for [$C_{56}H_{86}Cu_2N_4O_2Si_2$] calc. 1028.4881; found 1028.4915 $[M]^+$.
- Preparation of **[PhC{N(^{t}Bu)}$_2$Si{N(Dipp)SiMe$_3$}]**: A suspension of [Li{N(Dipp)(SiMe$_3$)}]$_2$ (1.71 g, 3.35 mmol)[4] and LSiCl (1 g, 3.35 mmol) was taken in a Schlenk flask and stirred in 60 mL diethyl ether. The resultant dark brown solution was filtered and reduced to ~5 mL. A binary mixture of diethyl ether and toluene (1:1) solution afforded light yellow-coloured single crystals at room temperature overnight. Yield: 1.2 g (71 %). ^{1}H NMR (400 MHz, C_6D_6, 298 K) δ 7.35 – 7.33 (m, 1H, Ar-H), 7.26 – 7.121 (m, 3H, Ar-H), 7.08 – 7.06 (m, 2H, Ar-H), 6.99 – 6.96 (m, 2H, Ar-H), 6.91 (dd, J = 7.5, 1.8 Hz, 1H, Ar-H), 4.03 – 4.00 (sept, J = 12 Hz, 2H, C*H*CH_3), 1.42 (d, J = 6.9 Hz, 6H, ^{i}Pr-C*H*$_3$), 1.36 (d, J = 6.8 Hz, 6H, ^{i}Pr-C*H*$_3$), 1.29 (s, 18H, {N-C(C*H*$_3$)$_3$}), 0.32 (s, 9H, -Si*Me*$_3$) ppm; ^{13}C NMR (101 MHz, C_6D_6) δ 164.26 (N*C*N), 148.14 (arom. C), 145.04 (arom. C), 135.59 (arom. C), 131.55 (arom. C), 129.41 (arom. C), 127.32 (arom. C), 124.99 (arom. C), 124.43 (arom. C), 54.27 (({N-*C*(CH_3)$_3$}), 32.99 ({-*C*H(CH_3)$_2$}), 27.81 ({-CH(*C*H_3)$_2$}), 27.79 ({-CH(*C*H_3)$_2$}), 24.40 ({N-C(*C*H_3)$_3$}), 4.04 (Si-{*C*H_3}$_3$) ppm; ^{29}Si{^{1}H} NMR (79.49 MHz, C_6D_6, 298 K): δ 3.0 (-DippN*Si*SiMe$_3$,) 0.6 [-(*Si*Me$_3$)$_2$] ppm. HRMS (ESI, CH_3CN) (*m/z*) for [$C_{30}H_{49}N_3Si_2$] calc. 507.3465; found 508.3596 $[M+H]^+$. M.p. 138.0 °C.

- Preparation of **11**: A Schlenk flask charged with LSi{N(Dipp)($SiMe_3$)}]$_2$ (254 mg, 0.5 mmol) and mesityl copper (91.37 mg, 0.5 mmol) was taken in 20 mL toluene and kept in stirring overnight under an inert atmosphere of Argon (Ar). The brown solution was filtered off and reduced to ~5 mL to obtain a colourless single crystal at ambient conditions. Yield: 250 mg (72 %). ^{1}H NMR (400 MHz, C_6D_6, 298 K) δ 7.19 – 7.17 (m, 3H, Ar-H), 7.03 (s, 2H, Ar-H), 6.98 – 6.88 (m, 5H, Ar-H), 3.76 (sept, 2H, J = 24 Hz, C*H*CH_3), 2.61 (s, 6H, *o*-C*H*$_3$), 2.38 (s, 3H, *p*-C*H*$_3$), 1.47 (d, J = 6.7 Hz, 6H, ^{i}Pr-C*H*$_3$), 1.27 (s, 6H, J =15.9 Hz ^{i}Pr-C*H*$_3$), 1.23 (s, 18H, {N-C(C*H*$_3$)$_3$}), 0.20 (s, 9H, -Si*Me*$_3$) ppm; ^{13}C NMR (101 MHz, C_6D_6) δ 173.3 (N*C*N), 147.5 (arom. C), 133.3 (arom. C), 130.26 (arom. C), 124.93 (arom. C), 55.25 ({N-*C*(CH_3)$_3$}, 32.63 ({-*C*H(CH_3)$_2$}), 28.0 {-CH(*C*H$_3$), 27.85 (mesityl C), 24.47 (mesityl C), 21.75 ({N-C(*C*H$_3$)$_3$}), 3.36 (Si-{*C*H$_3$}$_3$) ppm; ^{29}Si{^{1}H} NMR (79.49 MHz, C_6D_6, 298 K): δ 6.7 [-(*Si*Me$_3$)$_2$], 11.8 (*Si*-Cu) ppm. HRMS (ESI, CH_3CN) (*m/z*) for [$C_{39}H_{60}CuN_3Si_3$] calc. 689.3622; found 689.4143 [M]$^+$. M.p. 132.3 °C.
- Preparation of **12** and **13**: A Schlenk flask charged with compound 1 (210.42 mg, 0.5 mmol) and mesityl copper (91.37 mg, 0.5 mmol) followed by the addition of toluene (20 ml) and kept in stirring for 4 h. B_2Cat_2 (127mg, 0.5 mmol) was added to the solution and kept in stirring overnight under an inert atmosphere of Argon (Ar). The dark red-coloured solution was filtered off and concentrated to 2 mL to obtain colourless single crystals at room temperature. This afforded the desired complex. The spectroscopic data are in accordance with the previously reported literature.[5] For **12**; ^{1}H NMR (400 MHz, C_6D_6, 298 K) δ 0.34 (s, 9H, Si-C*H*$_3$), 0.74 (s, 9H, Si-C*H*$_3$), 1.11 (s, 18H, {N-C(C*H*$_3$)$_3$}), 6.92 (m, 4H, Ar-H), 7.02 (m, 3H, Ar-H), 7.33 (m, 2H, Ar-H) ppm; ^{13}C{^{1}H} NMR (100.613 MHz, C_6D_6, 298 K): δ 5.04 (Si-*C*H$_3$), 6.73 (Si-*C*H$_3$), 31.93 {N-C(*C*H$_3$)$_3$}, 54.93 {N-*C*(CH_3)$_3$}, 122.95, 125.70, 128.57, 129.34, 131.72 (Ar-*C*), 153.15 (N*C*N) ppm; ^{29}Si{^{1}H} NMR (79.49 MHz, C_6D_6, 298 K): δ 3.7 [-N(*Si*Me$_3$)$_2$], 6.3 [-N(*Si*Me$_3$)$_2$], 7.1 (*Si*-Cu) ppm. HRMS (ESI, CH_3CN) (*m/z*) for [$C_{27}H_{45}N_3CuBO_2Si_3$] calc. 601.2214; found 601.2728 [M]$^+$.
- For **13**: A very few light-yellow coloured crystals were obtained together with Mes-BCat at 0 °C, which decomposes rapidly at ambient conditions. ^{1}H NMR (400 MHz, C_6D_6, 298 K): δ ^{1}H NMR (400 MHz, C_6D_6, 298 K) δ 2.11 (s, 3H, Mes-C*H*$_3$), 2.55 (s, 6H, Mes-C*H*$_3$), 6.78 (s, 2H, Ar-H), 6.84 (dd, 2H, Ar-H), 7.12 (dd, 2H, Ar-H) ppm; ^{13}C{^{1}H} NMR (100.613 MHz, C_6D_6, 298 K): δ 21.35 (Mes-*C*H$_3$), 23.26 (Mes-*C*H$_3$), 109.01, 112.77, 118.23, 140.99, 145.10, 148.72 (Ar-*C*) ppm; ^{11}B {^{1}H} NMR (C_6D_6, 298 K, 128 MHz): δ 33.52 (Mes-*B*Cat) ppm. HRMS (ESI, CH_3CN) (*m/z*) for [$C_{15}H_{15}BO_2$] calc. 238.1165; found 238.0554 [M]$^+$.

- Preparation of **14**: A Schlenk flask charged with compound **1** (210.42 mg, 0.5 mmol) and mesityl copper (91.37 mg, 0.5 mmol) followed by addition of toluene (20 ml) and kept in stirring for 4 h. Diphenyl diselenide (Ph_2Se_2) (156.06 mg, 0.5 mmol) was added to the solution and kept in stirring overnight under inert atmosphere of Argon (Ar). The greenish-yellow colour solution was filtered off and concentrated to 2 ml, and the addition of 4 ml n-pentane produced yellow-coloured single crystals at room temperature. Yield: 255 mg (40 %). ^{1}H NMR (400 MHz, C_6D_6, 298 K) δ 0.38 (s, 9H, Si-C*H₃*), 0.72 (s, 9H, Si-C*H₃*), 1.23 (s, 18H, {N-C(CH_3)$_3$}), 6.93 (m, 3H, Ar-H), 7.03 (m, 1H, Ar-H), 7.09 (s, 2H, Ar-H), 7.20 (m, 1H, Ar-H), 7.35 (d, 1H, *J* = 5.9 Hz, Ar-H), 8.21 (d, 2H, *J* = 7.0 Hz, Ar-H) ppm; ^{13}C{^{1}H} NMR (100.613 MHz, C_6D_6, 298 K): δ 5.29 (Si-*C*H₃), 6.29 (Si-*C*H₃), 31.72 {N-C(*C*H₃)₃}, 54.42 {N-*C*(CH₃)₃}, 123.40, 127.53, 129.64, 129.86, 132.59, 136.76 (Ar-*C*), 166.12 (N*C*N) ppm; ^{29}Si{^{1}H} NMR (79.49 MHz, C_6D_6, 298 K): δ 4.4 [-N(*Si*Me₃)₂], 5.9 [-N(*Si*Me₃)₂], 5.9 (*Si*-Cu); ^{77}Se NMR (76.49 MHz, $CDCl_3$, 298 K): δ 37.1 ppm. HRMS (ESI, CH_3CN) (*m/z*) for [$C_{54}H_{92}Cu_2N_6Se_2Si_6$] calc. 1278.2921; found 1279.2452 $[M+H]^+$. M.p. 152.2 °C.
- NMR details of **mesityl(phenyl)selane**: The white precipitate formed during the reaction, collected and dried. All analytical data were performed without further purification. ^{1}H NMR (400 MHz, $CDCl_3$, 298 K): δ 2.33 (s, 3H, *p*-C*H₃*), 2.46 (s, 6H, o-C*H₃*), 7.02 (m, 2H), 7.14 (m, 5H); ^{13}C{^{1}H} NMR (100.613 MHz, C_6D_6, 298 K): δ 21.20, 24.40, 125.48, 126.89, 128.56, 128.99, 129.25, 133.60, 139.23, 143.78 ppm; ^{77}Se NMR (76.49 MHz, $CDCl_3$, 298 K): δ 290.4 ppm. HRMS (ESI, CH_3CN) (*m/z*) for [$C_{15}H_{16}Se$] calc. 276.0417; found 276.0455 $[M]^+$.
- Preparation of **15**: A Schlenk flask charged with compound **1** (210.42 mg, 0.5 mmol) and mesityl copper (91.37 mg, 0.5 mmol) followed by addition of toluene (20 ml) and kept in stirring for 4 h. Bis(2,4,6-trimethylphenyl) diselenide (Mes_2Se_2) (198 mg, 0.5 mmol) was added to the solution and kept in stirring overnight under inert atmosphere of Argon (Ar). The greenish-yellow colour solution was filtered off and concentrated to 2 ml, which afforded yellow-coloured single crystals at room temperature. Yield: 400 mg (69 %). ^{1}H NMR (400 MHz, C_6D_6, 298 K) δ 0.25 (s, 9H, Si-C*H₃*), 0.55 (s, 9H, Si-C*H₃*), 1.07 (s, 18H, {N-C(CH_3)$_3$}), 2.24 (s, 3H, Mes-C*H₃*), 3.10 (s, 6H, Mes-C*H₃*), 6.86 (m, 1H, Ar-H), 6.95 (m, 2H, Ar-H), 7.02 (s, 3H, Ar-H), 7.07 (s, 1H, Ar-H) ppm; ^{13}C{1H} NMR (100.613 MHz, C_6D_6, 298 K): δ 4.85 (Si-*C*H3), 5.85 (Si-*C*H₃), 21.14 (Mes-*C*H₃), 28.85 (Mes-*C*H₃), 31.74 {N-C(*C*H₃)₃}, 54.47 {N-*C*(CH₃)₃}, 129.46, 130.24, 131.43, 142.23, 155.37 (Ar-*C*), 167.02

(N*C*N) ppm; ^{29}Si{^{1}H} NMR (79.49 MHz, C_6D_6, 298 K): δ 6.7 [-N(*Si*Me$_3$)$_2$], 5.3 [-N(*Si*Me$_3$)$_2$], 3.2 (*Si*-Cu); ^{77}Se NMR (76.49 MHz, $CDCl_3$, 298 K): δ 230.4 ppm. M.p. 178°C.

- NMR details of **dimesityl selane**: The white precipitate formed during the reaction, collected and dried. All analytical data were performed without further purification. ^{1}H NMR (400 MHz, $CDCl_3$, 298 K): δ: 2.23 (6H, s, CH_3), 2.25 (12H, s, CH_3), 6.84 (4H, s, ArH) ppm; ^{13}C NMR (100 MHz, $CDCl_3$) δ: 20.96, 23.64, 125.46, 128.39, 129.20,141.50 ppm; ^{77}Se NMR (76.49 MHz, $CDCl_3$, 298 K): δ 334.3 ppm. HRMS (ESI, CH_3CN) (*m/z*) for [$C_{18}H_{22}Se$] calc. 318.0887; found 319.0959 $[M+H]^+$. M.p. <300°C.
- Preparation of **16**: A Schlenk flask charged with compound **7** (365 mg, 0.5 mmol) and bis(2,4,6-trimethylphenyl) diselenide (Mes_2Se_2) (198 mg, 0.5 mmol) was taken to the toluene solution and kept in stirring overnight under an inert atmosphere of Argon (Ar). The greenish-yellow colour solution was filtered off and concentrated to 2 ml, which afforded colourless single crystals at room temperature. Yield: 300 mg (72 %). ^{1}H NMR (400 MHz, $CDCl_3$, 298 K) δ 0.27 (s, 9H, Si-C*H$_3$*), 0.39 (s, 9H, Si-C*H$_3$*), 1.16 (s, 18H, {N-C(C*H$_3$*)$_3$}), 2.19 (s, 3H, Mes- C*H$_3$*), 2.57 (s, 6H, Mes-C*H$_3$*), 6.80 (s, 2H, Ar-H), 6.97 (m, 1H, Ar-H), 7.33-7.35 (m, 1H, Ar-H), 7.42-7.46 (m, 2H, Ar-H), 7.49-7.53 (m, 1H, Ar-H) ppm; ^{13}C{^{1}H} NMR (100.613 MHz, $CDCl_3$, 298 K): δ 4.79 (Si-*C*H$_3$), 5.79 (Si- *C*H$_3$), 20.91 (Mes-*C*H$_3$), 27.84 (Mes-*C*H$_3$), 31.93 {N-C(*C*H$_3$)$_3$}, 54.47 {N-*C*(CH$_3$)$_3$}, 127.43, 128.23, 130.76, 131.13, 141.69 (Ar-*C*), 166.84 (N*C*N) ppm; ^{29}Si{^{1}H} NMR (79.49 MHz, $CDCl_3$, 298 K): δ 7.28, 7.40 [-N(*Si*Me$_3$)$_2$], 4.12-4.47, 9.13-9.49 [two set of d, *Si*-Ag, ^{1}J ^{29}Si to ^{109}Ag and ^{107}Ag = 400.4, 399.6 Hz]; ^{77}Se NMR (76.49 MHz, $CDCl_3$, 298 K): δ 198.21 ppm. HRMS (ESI, CH_3CN) (*m/z*) for [$C_{33}H_{41}CuF_{10}N_4Si_3$] calc. 830.1775; found 829.1780 $[M-H]^-$. M.p. 193.3 °C.
- Preparation of **17**: A Schlenk flask charged with compound **2** (210.42 mg, 0.5 mmol) and mesityl copper (91.37 mg, 0.5 mmol) followed by addition of toluene (20 ml) and kept in stirring for 4 h. Pyrrole (33.55 mg, 0.5 mmol) was added to the solution and kept in stirring overnight under an inert atmosphere of Argon (Ar). The resultant solution was filtered and evaporated under reduced pressure, which afforded colourless single crystals at room temperature. Yield: 190 mg (69 %). ^{1}H NMR (400 MHz, C_6D_6, 298 K) δ 0.25 (s, 9H, Si-C*H$_3$*), 0.61 (s, 9H, Si-C*H$_3$*), 1.04 (s, 18H, {N-C(C*H$_3$*)$_3$}), 6.72 (m, 1H, Ar-H), 6.84 (m, 2H, Ar-H), 6.92 (m, 5H, Ar-H), 7.58 (m, 1H, Ar-H) ppm; ^{13}C{^{1}H} NMR (100.613 MHz, C_6D_6, 298 K): δ 4.65 (Si-*C*H$_3$), 6.06 (Si-*C*H$_3$), 31.78 (N-C*C*H$_3$), 54.63 {N-*C*(CH$_3$)$_3$}, 107.19, 125.69, 126.82, 129.33, 130.58, 131.31, 137.89, (Ar-*C*), 168.25 (N*C*N) ppm; ^{29}Si{^{1}H}

NMR (79.49 MHz, C_6D_6, 298 K): δ 5.9 [-N(*Si*Me$_3$)$_2$], 6.7 [-N(*Si*Me$_3$)$_2$], 7.8 (*Si*-Cu) ppm. HRMS (ESI, CH_3CN) (*m/z*) for [$C_{25}H_{45}CuN_4Si_3$] calc. 548.225; found 549.2099 $[M+H]^+$. M.p. 149.6 °C.

❖ Preparation of **18**: A Schlenk flask charged with compound **2** (210.42 mg, 0.5 mmol) and mesityl copper (91.37 mg, 0.5 mmol) followed by the addition of toluene (20 ml) and kept in stirring for 4 h. 2-mesityl-1H-pyrrole[6] (92.56 mg, 0.5 mmol) was added to the solution and kept in stirring overnight under an inert atmosphere of Argon (Ar). The resultant solution was filtered and evaporated under reduced pressure, which afforded colourless single crystals at room temperature. Yield: 233 mg (70 %). ^{1}H NMR (400 MHz, C_6D_6, 298 K) δ 0.50 (s, 9H, Si-C*H$_3$*), 0.81 (s, 9H, Si-C*H$_3$*), 1.24 (s, 18H, {N-C(C*H$_3$*)$_3$}), 2.54 (s, 3H, Mes-C*H$_3$*), 2.79 (s, 6H, Mes-C*H$_3$*), 6.84 (m, 1H, Ar-H), 7.07 (d, 1H, Ar-H), 7.21(m, 2H, Ar-H), 7.27-7.42 (m, 5H, Ar-H), 7.75 (m, 1H, Ar-H) ppm; ^{13}C{^{1}H} NMR (100.613 MHz, C_6D_6, 298 K): δ 4.68 (Si-*C*H$_3$), 5.95 (Si-*C*H$_3$), 21.51 (Mes-*C*H$_3$), 22.00 (Mes-*C*H$_3$), 31.72 ({N-C(*C*H$_3$)$_3$}), 54.47 {N-*C*(CH$_3$)$_3$}, 106.49, 107.94, 125.69, 127.15, 129.33, 130.63, 131.41, 134.51, 137.49, 139.19 (Ar-*C*), 167.89 (N*C*N) ppm; ^{29}Si{^{1}H} NMR (79.49 MHz, C_6D_6, 298 K): δ 5.9 [-N(*Si*Me$_3$)$_2$], 6.6 [-N(*Si*Me$_3$)$_2$], 7.4 (*Si*-Cu) ppm. HRMS (ESI, CH_3CN) (*m/z*) for [$C_{34}H_{54}CuN_4Si_3$] calc. 666.3031; found 665.1862 $[M-H]^-$. M.p. 193.1 °C.

❖ Preparation of **19**: A Schlenk flask was charged with compound **2** (210.42 mg, 0.5 mmol) and mesityl copper (91.37 mg, 0.5 mmol) followed by the addition of toluene (20 ml) and kept stirring for 4 h. *N*-mesitylpyridin-2-amine[7] (106 mg, 0.5 mmol) was added to the solution and kept in stirring overnight under an inert atmosphere of Argon (Ar). The resultant solution was filtered and evaporated under reduced pressure, which afforded colourless single crystals at room temperature. Yield: 190 mg (55 %). ^{1}H NMR (400 MHz, C_6D_6, 298 K) δ 0.40 (s, 9H, Si-C*H$_3$*), 0.72 (s, 9H, Si-C*H$_3$*), 1.27 (s, 18H, {N-C(C*H$_3$*)$_3$}), 2.40 (s, 3H, *p*-C*H$_3$*), 2.69 (s, 6H, *o*-C*H$_3$*), 5.96 (d, 1H, J = 8.5 Hz, Ar-H), 6.31 (m, 1H, Ar-H) 7.02-7.13 (m, 5H, Ar-H), 7.23-7.32 (m, 2H, Ar-H), 8.22 (d, 1H, J = 3.7 Hz, Ar-H) ppm; ^{13}C{^{1}H} NMR (100.613 MHz, C_6D_6, 298 K): δ 4.80 (Si-*C*H$_3$), 5.82 (Si-*C*H$_3$), 19.49 (mesityl-*C*H$_3$), 21.23 (mesityl-*C*H$_3$), 31.74 {N-C(*C*H$_3$)$_3$}, 54.57 {N-*C*(CH$_3$)$_3$}, 104.81, 106.69, 129.34, 130.33, 130.64, 132.11, 133.80, 137.11, 146.47, 148.00 (Ar-*C*), 167.13, 168.70 (N*C*N) ppm; ^{29}Si{^{1}H} NMR (79.49 MHz, C_6D_6, 298 K): δ 5.3 [-N(*Si*Me$_3$)$_2$], 6.4 [-N(*Si*Me$_3$)$_2$], 6.9 (*Si*-Cu) ppm. HRMS (ESI, CH_3CN) (*m/z*) for [$C_{35}H_{56}CuN_5Si_3$] calc. 693.3139; found 692.1946 $[M-H]^-$. M.p. 216.1 °C.

- Preparation of **20**: A Schlenk flask charged with compound **2** (210.42 mg, 0.5 mmol) and mesityl copper (91.37 mg, 0.5 mmol) followed by the addition of toluene (20 ml) and kept in stirring for 4 h. *N, N'*-bis(2,6-diisopropylphenyl)formimidamide[8] (182.29 mg, 0.5 mmol) was added to the solution and kept in stirring overnight under an inert atmosphere of Argon (Ar). The resultant solution was filtered and evaporated under reduced pressure, which afforded colourless single crystals at room temperature. Yield: 253 mg (60 %) ^{1}H NMR (400 MHz, C_6D_6, 298 K) δ 0.28 (s, 9H, Si-C*H₃*), 0.50 (s, 9H, Si-CH_3), 1.19 (s, 18H, {N-C(CH_3)$_3$}), 1.43 (d, 24H, J = 6.9 Hz, ^{i}Pr-CH_3), 4.05 (sept, 4H, J = 6.9 Hz, CH-CH$_3$), 6.76 (m, 1H, Ar-H), 6.86 (m, 2H, Ar-H), 7.04(m, 1H, Ar-H), 7.19 (s, 1H, Ar-H), 7.28 (d, 4H, J = 7.6 Hz, Ar-H), 7.59 (d, 1H, Ar-H), 7.63 (s, 1H, Ar-H) ppm; ^{13}C{^{1}H} NMR (100.613 MHz, C_6D_6, 298 K): δ 4.94 (Si-*C*H$_3$), 5.97 (Si-*C*H$_3$), 24.47 {CH-(*C*H$_3$)$_2$}, 26.41 {CH-(*C*H$_3$)$_2$}, 31.82 {N-C(*C*H$_3$)$_3$}, 42.21 {*C*H-(CH$_3$)$_2$}, 54.42 {N-*C*(CH$_3$)$_3$}, 118.68, 120.57, 122.42, 125.70, 129.33, 130.41, 132.09, 135.45, 137.88, 145.35, 149.13, 158.51, (Ar-*C*), 165.67, 166.87 (N*C*N) ppm; ^{29}Si{^{1}H} NMR (79.49 MHz, C_6D_6, 298 K): δ 4.9 [-N(*Si*Me$_3$)$_2$], 5.4 [-N(*Si*Me$_3$)$_2$], 6.6 (*Si*-Cu) ppm. HRMS (ESI, CH$_3$CN) (*m/z*) for [$C_{46}H_{76}CuN_5Si_3$] calc. 845.4705; found 868.4382 [M+Na]$^+$. M.p. 215.2 °C.
- Preparation of **21**: A Schlenk flask charged with compound **2** (210.42 mg, 0.5 mmol) and mesityl copper (91.37 mg, 0.5 mmol), followed by the addition of toluene (20 ml) and kept in stirring for 4 h. Bis-perfluoroarylamine[9] (174.56 mg, 0.5 mmol) was added to the solution and kept in stirring overnight under an inert atmosphere of Argon (Ar). The resultant solution was filtered and evaporated under reduced pressure, which afforded colourless single crystals at room temperature. Yield: 269 mg (65 %). ^{1}H NMR (400 MHz, C_6D_6, 298 K) δ 0.23 (s, 9H, Si-CH_3), 0.47 (s, 9H, Si-CH_3), 1.05 (s, 18H, {N-C(CH_3)$_3$}), 6.91 (m, 3H, Ar-H), 7.06(m, 2H, Ar-H) ppm; ^{13}C{^{1}H} NMR (100.613 MHz, C_6D_6, 298 K): δ 4.74 (Si-*C*H$_3$), 5.53 (Si-*C*H$_3$), 31.51 {N-C(*C*H$_3$)$_3$}, 54.66 {N-*C*(CH$_3$)$_3$}, 127.34, 130.70, 131.17, 168.59 (N*C*N) ppm; ^{19}F {^{1}H} (376 MHz, C_6D_6, 298 K): δ -174.3, -167.1, -154.7 ppm; ^{29}Si{^{1}H} NMR (79.49 MHz, C_6D_6, 298 K): δ 6.2 [-N(*Si*Me$_3$)$_2$], 7.1 (*Si*-Cu) ppm. HRMS (ESI, CH$_3$CN) (*m/z*) for [$C_{33}H_{41}CuF_{10}N_4Si_3$] calc. 830.1775; found 829.1780 [M-H]$^-$. M.p. 211.7 °C.
- Preparation of **22**: A Schlenk flask charged with compound **7** (365 mg, 0.5 mmol) and bis-perfluoroarylamine[9] (174.56 mg, 0.5 mmol) was taken in toluene and stirred overnight under an inert atmosphere of Argon (Ar). The resultant solution was filtered and evaporated under reduced pressure, affording colourless single crystals at room temperature. Yield:

200 mg (45 %). ^{1}H NMR (400 MHz, C_6D_6, 298 K) δ 0.30 (s, 9H, Si-CH_3), 0.49 (s, 9H, Si-CH_3), 1.01 (s, 18H, {N-C(CH_3)$_3$}), 6.84-6.87 (m, 1H, Ar-H), 6.94-6.97(m, 1H, Ar-H), 7.00-7.04 (m, 3H, Ar-H) ppm; $^{13}C\{^{1}H\}$ NMR (100.613 MHz, C_6D_6, 298 K): δ 4.78 (Si-CH$_3$), 5.74 (Si-CH$_3$), 24.45, 31.65 {N-C(CH$_3$)$_3$},34.82, 54.80 {N-C(CH$_3$)$_3$}, 122.43, 127.34, 128.57, 130.72, 149.15 (NCN) ppm; HRMS (ESI, CH_3CN) (*m/z*) for [$C_{33}H_{41}AgF_{10}N_4Si_3$] calc. 874.1530; found 874.3388 $[M]^+$. M.p. 214.6 °C.

- Preparation of **23**: A Schlenk flask charged with compound **2** (300 mg, 0.5 mmol) and PPh_2SiMe_3 (129 mg, 0.5 mmol) was taken to the toluene solution and kept in stirring under an inert atmosphere of Argon (Ar) for 3 days. The resultant yellow solution was filtered and concentrated to obtain yellow-coloured crystals at room temperature. Yield: 217 mg (32 %). Poor solubility hinders the NMR characterisation. HRMS (ESI, CH_3CN) (*m/z*) for [$C_{66}H_{102}Cu_2N_6P_2Si_6$] calc. 1334.4849; found 677.3398 $[\frac{1}{2}M]^+$. M.p. 145.4 °C.
- Preparation of **24**: A Schlenk flask charged with compound **7** (365 mg, 0.5 mmol) and PPh_2SiMe_3 (129 mg, 0.5 mmol) was taken to the toluene solution and kept in stirring under an inert atmosphere of Argon (Ar) for 3 days. The resultant yellow solution was filtered and concentrated to obtain yellow-coloured crystals at room temperature. Yield: 214 mg (30 %) ^{1}H NMR (400 MHz, $CDCl_3$) δ 7.39 (dt, J = 15.9, 7.0 Hz, 5H, Ar-H), 7.30 – 7.22 (m, 6H, Ar-H), 7.13 (dd, J = 12.2, 5.2 Hz, 4H, Ar-H), 7.05 (t, J = 7.3 Hz, 4H, Ar-H), 1.12 (s, 18H, {N-C(CH_3)$_3$}), 0.37 (s, 9H, Si-CH_3), 0.19 (s, 9H, Si-CH_3); $^{13}C\{^{1}H\}$ NMR (100.613 MHz, $CDCl_3$) δ 168.0 (NCN), 148.8 (arom. C), 134.6 (arom. C), 134.5 (arom. C), 134.4 (arom. C), 133.2 (arom. C), 131.0 (arom. C), 130.8 (arom. C), 129.1 (arom. C), 128.8 (arom. C), 128.7 (arom. C), 128.5 (arom. C), 128.4 (arom. C), 128.4 (arom. C), 128.3 (arom. C), 127.2 (arom. C), 122.2 (arom. C), 55.0 {N-C(CH$_3$)$_3$}, 32.0 {N-C(CH$_3$)$_3$}, 6.1 (Si-CH$_3$), 4.9 (Si-CH$_3$); $^{29}Si\{^{1}H\}$ NMR (79.49 MHz, $CDCl_3$, 298 K): δ 7.67, 7.49 [-N(*Si*Me$_3$)$_2$], 2.80-3.23, 9.30-8.87 (two set of d, *Si*-Ag ^{1}J ^{29}Si to ^{109}Ag and ^{107}Ag = 484.2, 484.2 Hz; $^{31}P\{^{1}H\}$ NMR (162 MHz, $CDCl_3$) δ -13.26 (s). HRMS (ESI, CH_3CN) (*m/z*) for [$C_{66}H_{102}Ag_2N_6P_2Si_6$] calc. 1422.4359; found 712.3854 $[\frac{1}{2}M+H]^+$. M.p. 124.9 °C
- **S1.2. C–C Cross-Coupling Reactions**

➢ General procedure for C–C cross-coupling reactions with the conventional method:

A 100 mL Schlenk tube was charged with complex **1** (0.50 mmol) and organo-copper (0.50 mmol), followed by the addition of toluene (8 mL) and stirring for 30 minutes at room

temperature. 0.33 mmol of aryl halide was added to the solution, followed by the addition of 0.50 mmol of NaO^{t}Bu and 0.50 mol% Pd(dba)$_2$. The reaction mixture was then stirred for 16 h at 110 °C under an Argon (Ar) atmosphere. After 16 h, the reaction mixture was cooled to room temperature and extracted with *n*-hexane. The resulting solution was concentrated on a rota-evaporator and purified by column chromatography (*n*-hexane) to afford the expected product. All the C–C coupled products were characterised by NMR spectroscopy and mass spectrometry.

➢ Optimisation of the C–C coupling reaction

Table S1. Optimisation of reaction conditions for the C–C coupling reaction

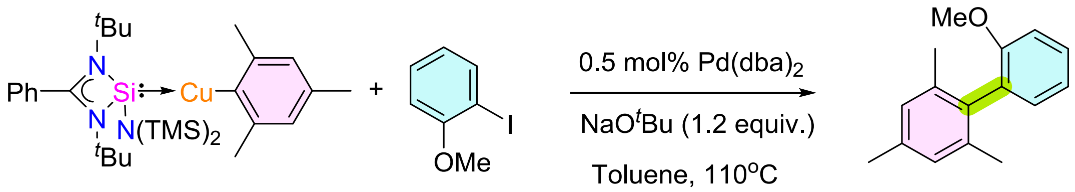

Entry	Substrate	Catalyst	Base	Mol %	Time (h)	Conversion Yield# (%)
1	2	-	NaO^{t}Bu	-	24	trace
2	2	Pd(dba)$_2$	NaO^{t}Bu	0.1	24	60
3	2	Pd(dba)$_2$	NaO^{t}Bu	0.5	24	<99
**4**	**2**	**Pd(dba)$_2$**	NaO^{t}Bu	**0.5**	**16**	**<99**
5	Mesityl Copper	Pd(dba)$_2$	NaO^{t}Bu	0.5	24	40
6	2	Pd(dba)$_2$	-	0.5	16	62
7	2	-	-	-	16	22

^{1}H NMR spectroscopy was used to determine the conversion yield of the products.

❖ **<u>2'-Methoxy-2,4,6-trimethyl-1,1'-biphenyl (I):</u>** Compound **I** was prepared according to the general procedure; 1-iodo-2-methoxybenzene (44 mg, 21.67 µL, 0.33 mmol) was used as the aryl halide. The product was obtained as a colourless oil. Yield: 74 mg (99 %). ^{1}H NMR (400 MHz, CDCl$_3$): δ 1.99 (s, 6H, *o*-CH_3), 2.34 (s, 3H, *p*-CH_3), 3.75 (s, 3H, -OCH_3),

6.95 (s, 2H, aromatic C-*H* of mesityl), 7.03- 6.98 (m, 3H), 7.34 (m, 1H); ^{13}C NMR (100.613 MHz, $CDCl_3$): δ 156.84, 136.62, 135.41, 131.10, 129.62, 128.41, 128.05, 120.76, 55.55 21.28, 20.49. HRMS (ESI, CH_3CN) (*m/z*) for [$C_{16}H_{18}O$] calc. 226.1358; found 227.1447 $[M+H]^+$. All spectral data are consistent with those previously reported.[10]

- **2,3',4,5',6-pentamethyl-1,1'-biphenyl (II)**: Compound **II** was prepared according to the general procedure; 1-iodo-2-methoxybenzene (77.35 mg, 48 µL, 0.33 mmol) was used as the aryl halide. The product was obtained as a colourless oil. Yield: 54 mg (72.3%). ^1H NMR (400 MHz, $CDCl_3$): δ 2.07 (s, 6H, -CH_3), 2.38 (s, 3H, *p*-CH_3), 2.40 (s, 6H, *o*-CH_3), 6.81 (s, 2H, aromatic C-*H* of mesityl), 6.98 (m, 2H), 7.02 (s, 1H); ^{13}C NMR (100.613 MHz, $CDCl_3$): δ 141.12, 139.44, 137.79, 136.37, 136.04, 128.08, 127.13, 125.25, 21.51, 21.14, 20.89. HRMS (ESI, CH_3CN) (*m/z*) for [$C_{17}H_{20}$] calc. 224.1565; found 225.1642 $[M+H]^+$. All spectral data are consistent with those previously reported.[11]
- **2,4,6-trimethyl-1,1'-biphenyl (III)**: Compound **III** was prepared according to the general procedure; iodobenzene (68 mg, 37.30 µL, 0.33 mmol) was used as the aryl halide. The product was obtained as a colourless oil. Yield: 61.8 mg (94.45 %). ^1H NMR (400 MHz, $CDCl_3$): δ 2.39 (s, 3H, o-CH_3), 2.06 (s, 6H, *p*-CH_3), 7.00 (m, 2H), 7.21 (m, 2H), 7.37(m, 1H), 7.46 (m, 2H); ^{13}C NMR (100.613 MHz, $CDCl_3$): δ 141.23, 139.20, 136.68, 136.10, 129.43, 128.49, 128.18, 126.63, 21.16, 20.87. HRMS (ESI, CH_3CN) (*m/z*) for [$C_{15}H_{17}$] calc. 196.1252; found 219.1714 $[M+Na]^+$. All spectral data are consistent with those previously reported.[12]
- **1-mesityl naphthalene (IV)**: Compound **IV** was prepared according to the general procedure; 1-iodo-2-methoxybenzene (84.7 mg, 48.6 µL, 0.33 mmol) was used as the aryl halide. The product was obtained as a colourless oil. Yield: 64.6 mg (79 %). ^1H NMR (400 MHz, $CDCl_3$): δ 1.95 (s, 6H, *o*-CH_3), 2.46 (s, 3H, *p*-CH_3), 7.08 (s, 2H, aromatic C-*H* of mesityl), 7.33 (d, 1H), 7.43 (m, 2H), 7.52 (t, 1H), 7.59 (t, 1H), 7.97-7.90 (dd, 2H); ^{13}C NMR (100.613 MHz, $CDCl_3$): δ 138.97, 136.99, 136.93, 136.88, 133.89, 132.13, 128.41, 128.20, 127.24, 126.84,126.12 125.85, 125.62, 21.27, 20.43. HRMS (ESI, CH_3CN) (*m/z*) for [$C_{19}H_{19}$] calc. 246.1409; found 247.1592 $[M+H]^+$. All spectral data are consistent with those previously reported.[13]
- **2-mesityl thiophene (V)**: Compound **V** was prepared according to the general procedure; 2-iodothiophene (70.01 mg, 36.81 µL, 0.33 mmol) was used as the aryl halide. The product was obtained as a colourless oil. Yield: 54.6 mg (80.96 %). ^1H NMR (400 MHz, $CDCl_3$): δ 2.16 (s, 6H, *o*-CH_3), 2.37 (s, 3H, *p*-CH_3), 6.84 (d, 1H), 6.98 (s, 2H, aromatic C-*H* of

mesityl), 7.14 (s, 1H), 7.39 (d, 1H); ^{13}C NMR (100.613 MHz, $CDCl_3$): δ 141.63, 138.41, 137.92, 131.22, 128.22, 127.11, 126.54, 125.37, 21.20, 20.81. HRMS (ESI, CH_3CN) (*m/z*) for [$C_{13}H_{15}S$] calc. 202.0816; found 203.0887 $[M+H]^+$. All spectral data are consistent with those previously reported.[14]

- **2'-methoxy-2,6-dimethyl-1,1'-biphenyl (VI):** Compound **VI** was prepared according to the general procedure. 1-iodo-2-methoxybenzene (44 mg, 21.67 µL, 0.33 mmol) was used as an aryl halide. The product was obtained as a colourless oil. Yield: 54.0 mg (76.4 %). ^1H NMR (400 MHz, $CDCl_3$): δ 7.30-7.25 (m, 2H), 7.20- 7.18 (m, 1H), 7.13-7.09 (m, 1 H), 7.06-7.04 (m, 2H), 6.97-6.91 (m, 3H), 3.67 (s, 3H), 1.96 (s, 6H); ^{13}C NMR (100.613 MHz, $CDCl_3$) δ: 141.94, 137.32, 135.61, 128.60, 128.51, 128.26, 128.01, 127.59, 126.95, 126.46, 126.06, 125.87, 38.08, 20.35, 19.94. HRMS (ESI, CH_3CN) (*m/z*) for [$C_{15}H_{16}O$] calc. 212.1201; found 213.1447 $[M+H]^+$. All spectral data are consistent with those previously reported.
- **2,3',5',6-tetramethyl-1,1'-biphenyl (VII):** Compound **VII** was prepared according to the general procedure. 1-iodo-2-methoxybenzene (77.35 mg, 48 µL, 0.33 mmol) was used as an aryl halide. The product was obtained as a colourless oil. Yield: 59.8 mg (85.4 %). ^1H NMR (400 MHz, $CDCl_3$): δ 7.20-7.14 (m, 3H), 7.03 (s, 1H), 6.82 (s, 1H), 2.40 (s, 6H), 2.10 (s, 6H); ^{13}C NMR (100.613 MHz, $CDCl_3$) δ:142.26, 141.12, 137.86, 136.13, 128.25, 127.30, 126.90, 126.84, 125.25, 21.50, 20.99. HRMS (ESI, CH_3CN) (*m/z*) for [$C_{16}H_{18}$] calc. 210.1409; found 211.1493 $[M+H]^+$. All spectral data are consistent with those previously reported.
- **2-(2,6-dimethylphenyl)thiophene (VIII):** Compound **VIII** was prepared according to the general procedure. 2-Iodothiophene (70.01 mg, 36.81 µL, 0.33 mmol) was used as an aryl halide. The product was obtained as a colourless oil. Yield: 46.7 mg (74.5 %). ^1H NMR (400 MHz, $CDCl_3$): δ 7.40-7.39 (m, 1H),7.22-7.18 (m, 1H), 7.13-7.12 (m, 3H), 6.84 (m, 1H), 2.17 (s, 6H); ^{13}C NMR (100.613 MHz, $CDCl_3$) δ: 141.48, 138.59, 134.21, 128.21, 127.39, 127.19, 126.40, 125.42, 20.93. HRMS (ESI, CH_3CN) (*m/z*) for [$C_{12}H_{12}S$] calc. 188.0660; found 189.1441 $[M+H]^+$. All spectral data are consistent with those previously reported.
- **1-(2,6-dimethylphenyl)naphthalene (IX):** Compound **IX** was prepared according to the general procedure. 1-iodo-2-methoxybenzene (84.7 mg, 48.6 µL, 0.33 mmol) was used as an aryl halide. The product was obtained as a colourless solid. Yield: 73.6 mg (95.2 %). ^1H NMR (400 MHz, $CDCl_3$): δ 7.83-7.76 (m, 2H), 7.46 (m, 1H), 7.38 (m, 1H), 7.26 (s, 2H),

7.20-7.16 (m, 2H), 7.11-7.09 (m, 2H), 1.83 (s, 6H); ^{13}C NMR (100.613 MHz, $CDCl_3$) δ: 139.78, 138.89, 137.13, 133.89, 131.88, 128.43, 128.28, 127.96, 126.69,126.20, 125.91, 125.84, 125.51, 20.53. HRMS (ESI, CH_3CN) (*m/z*) for [$C_{18}H_{16}$] calc. 232.1252; found 252.1392 $[M+H]^+$. All spectral data are consistent with those previously reported.

➢ S1.3. *α*-amino-benzyl nitriles synthesis

- **General procedure for three-component coupling reactions with microwave heating:**

An oven-dried microwave tube (10 mL) was charged with aldehyde (0.5 mmol) and aniline (0.5 mmol), along with the catalyst (5.0 mol%), and trimethylsilyl cyanide (50 mg, 0.5 mmol) was added to it. The suspension was heated under microwave irradiation at 80/120 °C for 3-4 h. After cooling to room temperature, the mixture was purified by column chromatography over silica gel using an ethyl acetate-hexane mixture as eluent.

Table S2. Optimisation of reaction conditions

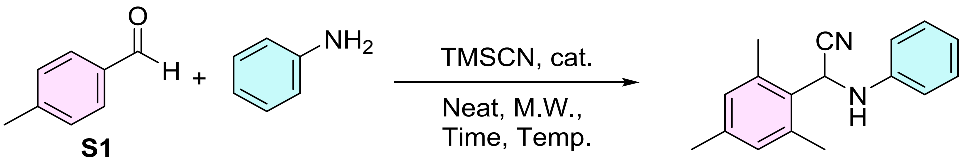

Entry	Substrate	Catalyst	Mol %	Time (h)	Temp. (°C)	Yield (%)
1	**S1**	**6**	**1**	**2**	**80**	**22**
2	**S1**	**7**	**1**	**2**	**80**	**trace**
3	**S1**	8	1	2	80	trace
4	**S1**	**6**	5	2	80	63
5	**S1**	**7**	5	2	80	trace
6	**S1**	**8**	5	2	80	40
7	**S1**	6	5	1	80	48
8	**S1**	6	5	2	60	30
9	**S1**	6	5	3	60	45

- **2-(phenylamino)-2-(*p*-tolyl) acetonitrile (X)**: It was synthesised following the general procedure by reacting 4-methylbenzaldehyde (60 mg, 0.5 mmol), aniline (46.5 mg, 0.5 mmol), and trimethylsilyl cyanide (50 mg, 0.5 mmol) for 2 h at 80 °C in a microwave. Yield: 70.8 mg (63 %). ^{1}H NMR (400 MHz, $CDCl_3$) δ 7.45-7.43 (d, J = 7.7 Hz, 2H), 7.26-7.22 (m, 4H), 6.88-6.85 (m, 1H), 6.75-6.73 (d, J = 7.9 Hz, 2H), 5.35 (s, 1H), 4.04 (s, 1H), 2.36 (s, 3H); ^{13}C {^{1}H} NMR (101 MHz, $CDCl_3$) δ 144.88, 139.72, 131.15, 130.11, 129.69, 127.30, 120.31, 118.48, 114.23, 50.09, 21.31 ppm. HRMS (ESI, CH_3CN) (*m/z*) for $C_{15}H_{14}N_2$ calc. 222.1157; found 223.1249 $[M+H]^+$. All spectral data are consistent with those previously reported.[15]
- **2-mesityl-2-(phenylamino) acetonitrile (XI)**: It was synthesised following the general procedure by reacting 2,4,6-trimethylbenzaldehyde (222.3 mg, 1.5 mmol), aniline (139.5 mg, 1.5 mmol), and trimethylsilyl cyanide (180 mg, 1.8 mmol) for 2 h at 80 °C in a microwave. Yield: 339.4 mg (90 %). ^{1}H NMR (400 MHz, $CDCl_3$): δ 7.36-7.32 (m, 2H), 7.00 (s, 2H), 6.96-6.92 (m, 1H), 6.87-6.85 (d, J = 7.55 Hz, 2H), 5.65-5.64 (d, J = 5.25 Hz, 1H), 3.93 (s, 1H), 2.55 (s, 6H), 2.36 (s, 3H); ^{13}C NMR (125 MHz, $CDCl_3$): δ 145.25, 139.33, 136.71, 130.48, 129.61, 127.96, 119.75, 118.62, 113.53, 45.04, 20.93, 20.32. HRMS (ESI, CH_3CN) (*m/z*) for $C_{17}H_{18}N_2$ calc. 250.1470; found 251.1565 $[M+H]^+$. All spectral data are consistent with those previously reported.[16]
- **2-(naphthalen-1-yl)-2-(phenylamino) acetonitrile (XII)**: It was synthesised following the general procedure by reacting 1-napthaldehyde (78 mg, 0.5 mmol), aniline (46.5 mg, 0.5 mmol), and trimethylsilyl cyanide (50 mg, 0.5 mmol) for 2 h at 80 °C in a microwave. Yield: 115.6 mg (89 %). ^{1}H NMR (400 MHz, $CDCl_3$) δ 7.98-7.92 (m, 4H), 7.57-7.53 (m, 3H), 7.36-7.32 (t, J = 7.6 Hz, 2H), 6.98-6.94 (t, J = 7.3 Hz, 1H), 6.87-6.85 (d, J = 8.2 Hz, 2H), 6.03-6.02 (d, J = 8.0 Hz, 1H), 4.07 (s, 1H); ^{13}C {^{1}H} NMR (101 MHz, $CDCl_3$) δ 144.92, 134.16, 130.83, 130.27, 129.82, 129.23, 129.14, 127.59, 126.71, 126.41, 125.40, 122.89, 120.31, 118.42, 113.88, 48.52 ppm. HRMS (ESI, CH_3CN) (*m/z*) for $C_{18}H_{14}N_2$ calc. 258.3240; found 259.1248 $[M+H]^+$. All spectral data are consistent with those previously reported.[15]
- **2-((2,6-dimethylphenyl)amino)-2-(naphthalen-1-yl) acetonitrile (XIII)**: It was synthesised following the general procedure by reacting 1-napthaldehyde (78 mg, 0.5 mmol), 2,6-dimethylaniline (60.6 mg, 0.5 mmol), and trimethylsilyl cyanide (50 mg, 0.5 mmol) for 2 h at 80 °C in a microwave. Yield: 96.2 mg (67 %). ^{1}H NMR (400 MHz, $CDCl_3$) δ 8.21-8.19 (d, J = 8.2 Hz, 1H), 7.98-7.595 (m, 3H), 7.63-7.56 (m, 2H), 7.13-7.11 (m, 2H),

7.03-6.99 (m, 1H), 5.80 (s, 1H), 3.70 (s, 1H), 2.40 (s, 6H); ^{13}C {^{1}H} NMR (101 MHz, $CDCl_3$) δ 142.65, 134.19, 131.17, 130.89, 130.47, 130.30, 129.41, 129.23, 127.35, 126.60, 125.86, 125.58, 124,36 122.98, 119.09, 51.47, 18.83 ppm. HRMS (ESI, CH_3CN) (*m/z*) for $C_{20}H_{18}N_2$ calc. 286.1470; found 287.1559 $[M+H]^+$.

- **<u>2-(4-methoxyphenyl)-2-(phenylamino) propanenitrile (XIV):</u>** It was synthesised following the general procedure by reacting 4-Methoxyacetophenone (226 mg, 1.5 mmol), aniline (139.5 mg, 1.5 mmol), and trimethylsilyl cyanide (180 mg, 1.8 mmol) for 2 h at 80 °C in a microwave. Yield: 189 mg (50 %). ^{1}H NMR (400 MHz, $CDCl_3$): δ 7.54-7.52 (d, J = 10 Hz, 2H), 7.14-7.10 (m, 2H), 6.93-6.90 (d, J = 10 Hz, 2H), 6.82-6.80 (m, 1H), 6.57-6.55 (d, J = 10 Hz, 2H), 4.23 (s, 1H, N*H*), 3.82 (s, 3H, OCH_3), 1.92 (s, 3H, CH_3). ^{13}C NMR (125 MHz, $CDCl_3$): δ 159.82, 143.73, 131.96, 129.16, 126.33, 120.99, 120.13, 116.04, 114.65, 56.81, 55.49, 33.60. HRMS (ESI, CH_3CN) (*m/z*) for $C_{16}H_{16}N_2O$ calc. 252.1263; found 253.1354 $[M+H]^+$. All spectral data are consistent with those previously reported.[17]

S2.1. NMR Spectra of Complexes 2-20

(*: residual solvent peak = Toluene)

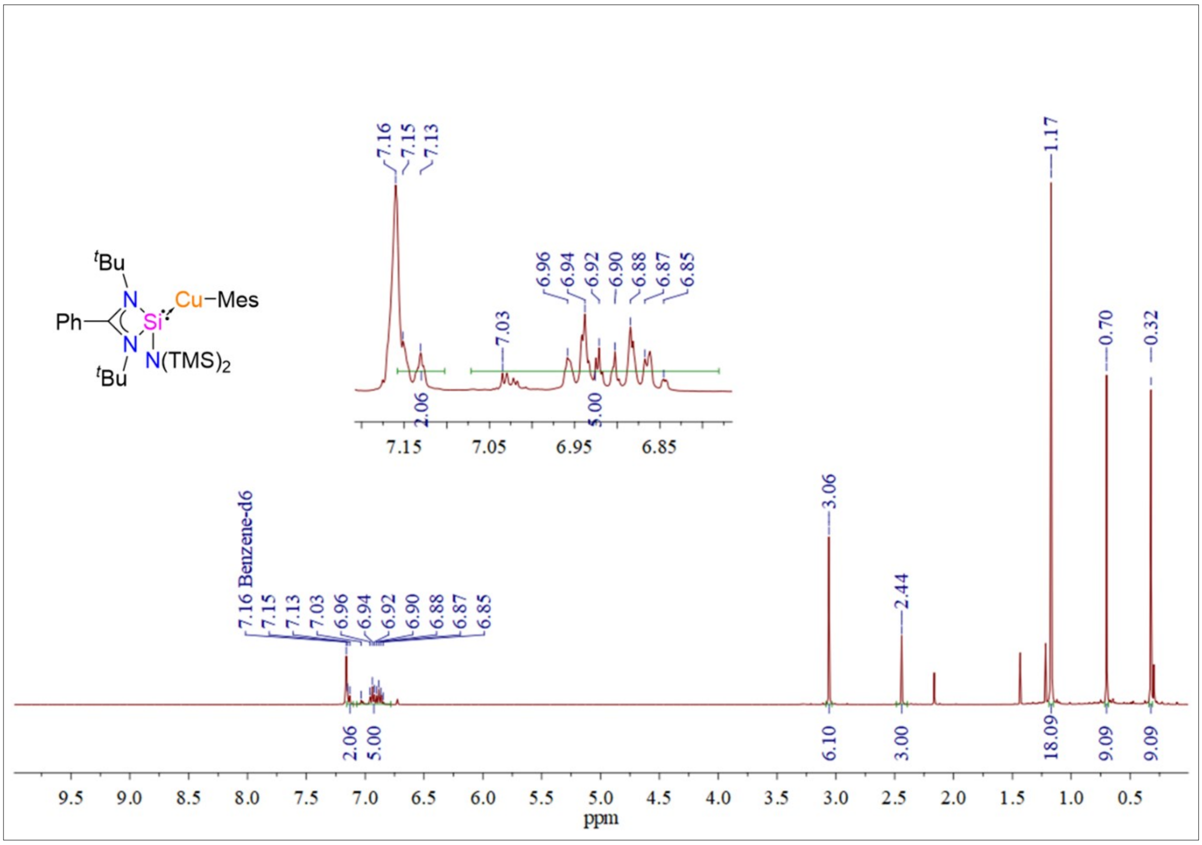

Figure S1. ^{1}H NMR spectrum of complex **2**.

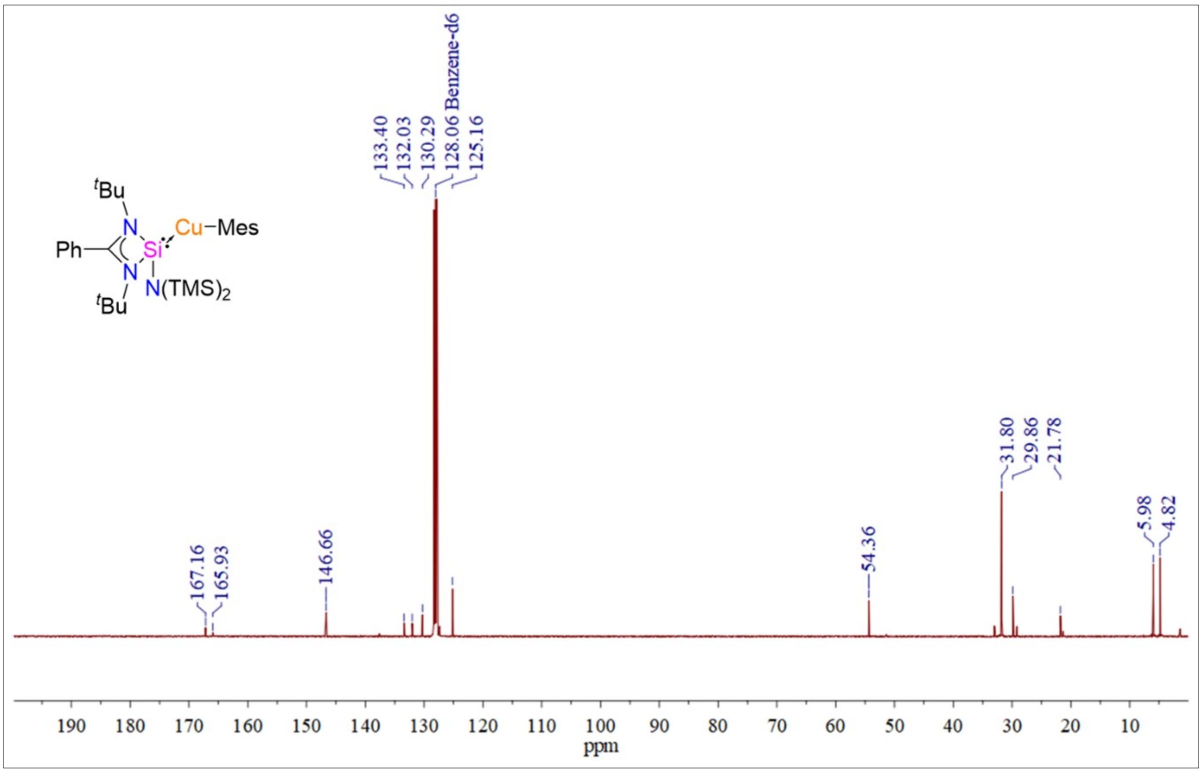

Figure S2. $^{13}C\{^{1}H\}$ NMR spectrum of complex **2**.

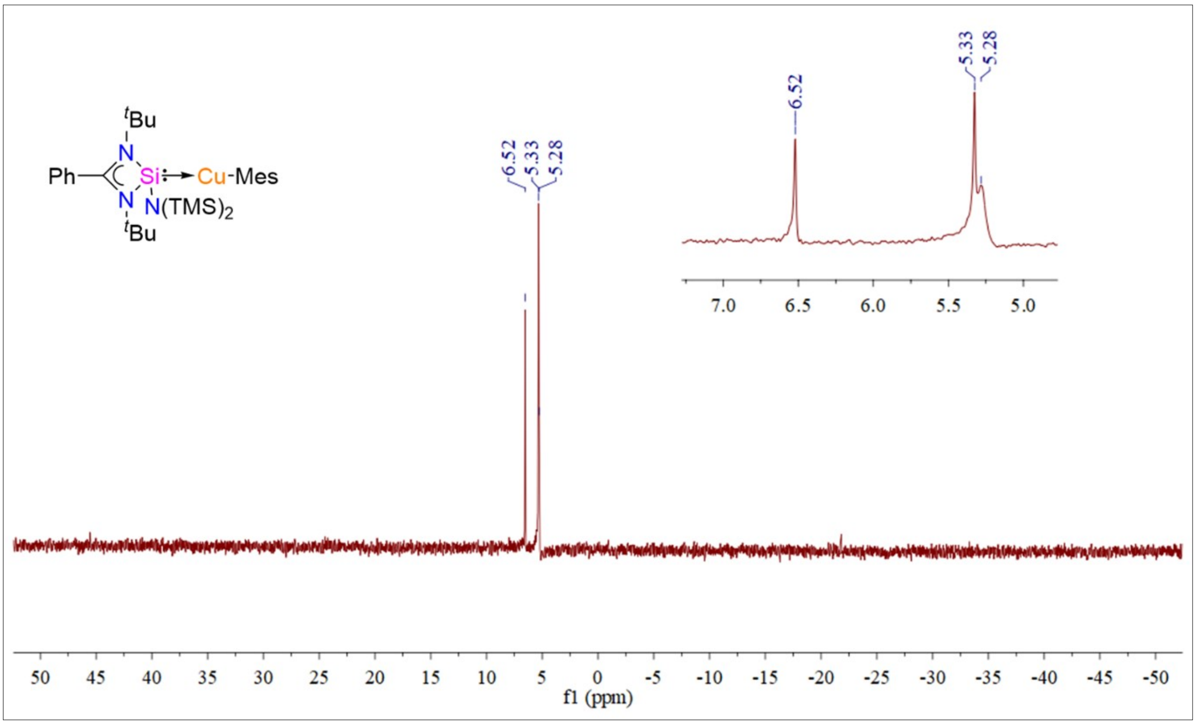

Figure S3. $^{29}Si\{^{1}H\}$ NMR spectrum of complex **2**.

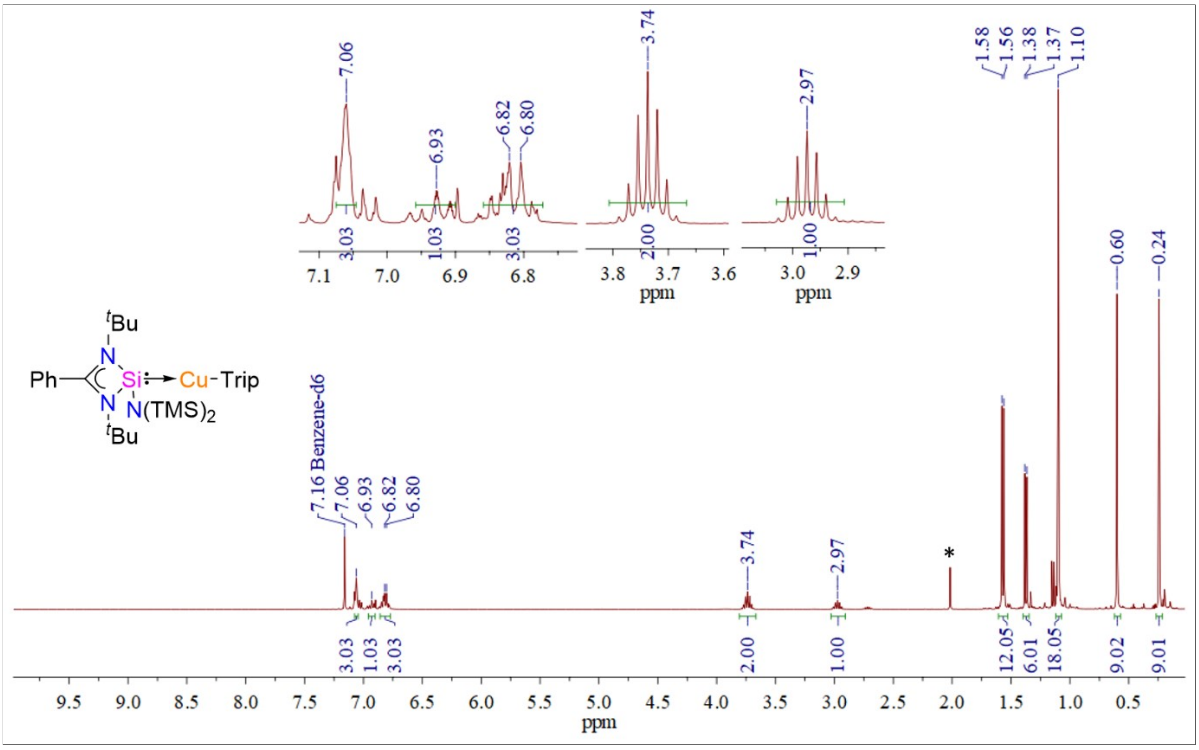

Figure S4. ^{1}H NMR spectrum of complex **3**.

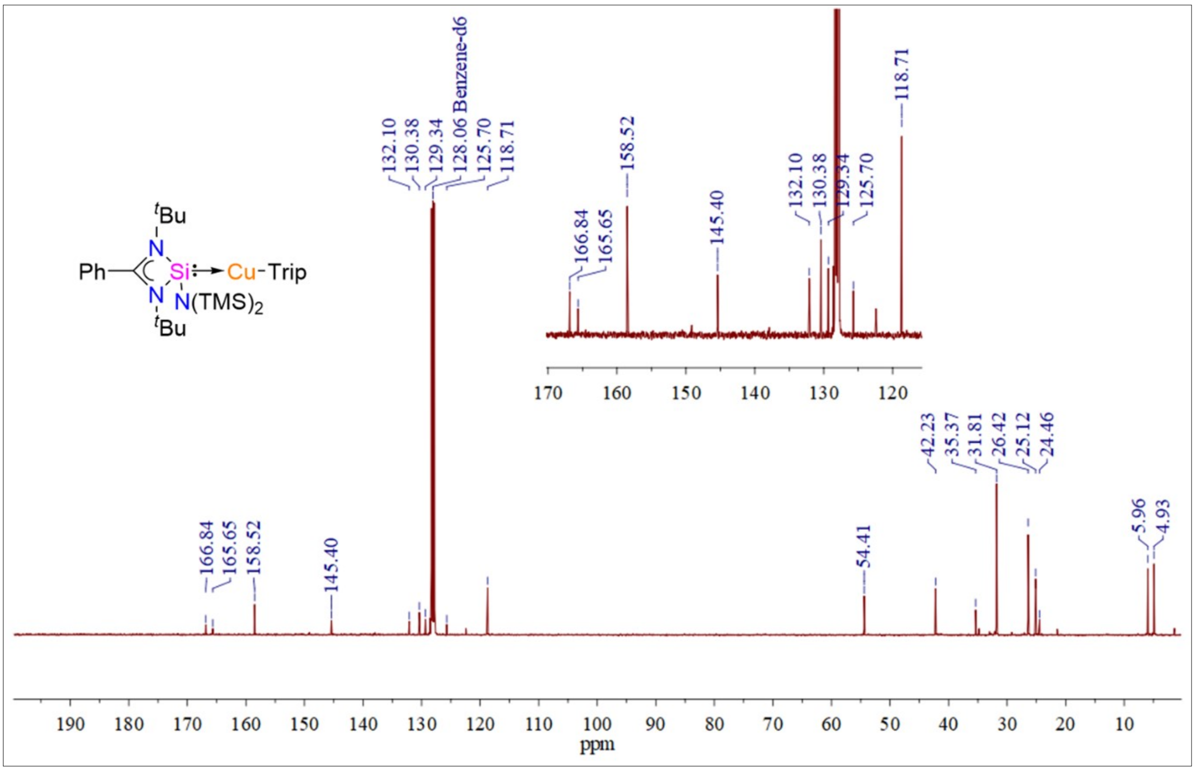

Figure S5. $^{13}C\{^{1}H\}$ NMR spectrum of complex **3**.

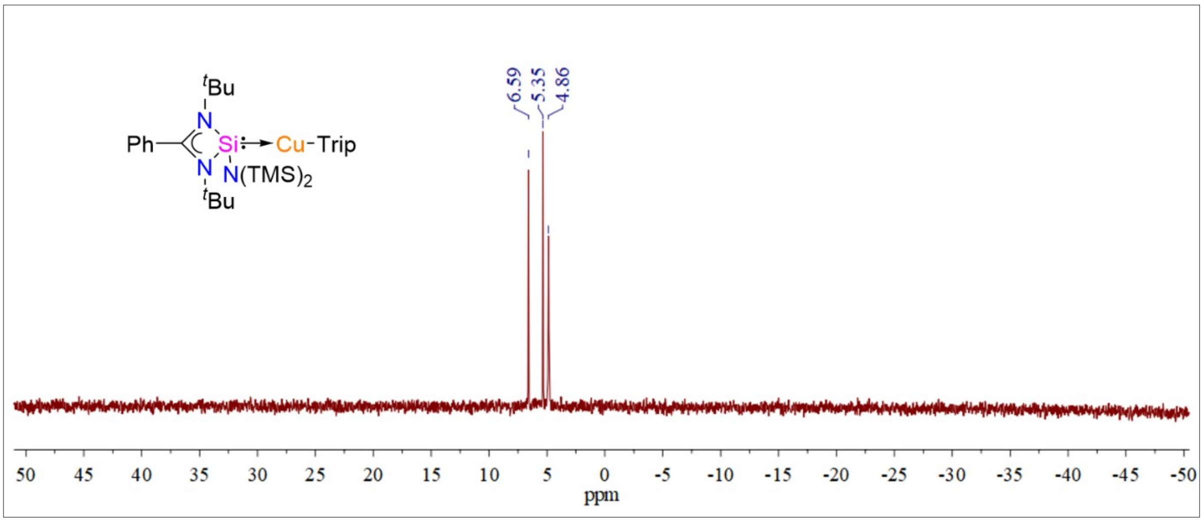

Figure S6. $^{29}Si\{^1H\}$ NMR spectrum of complex **3**.

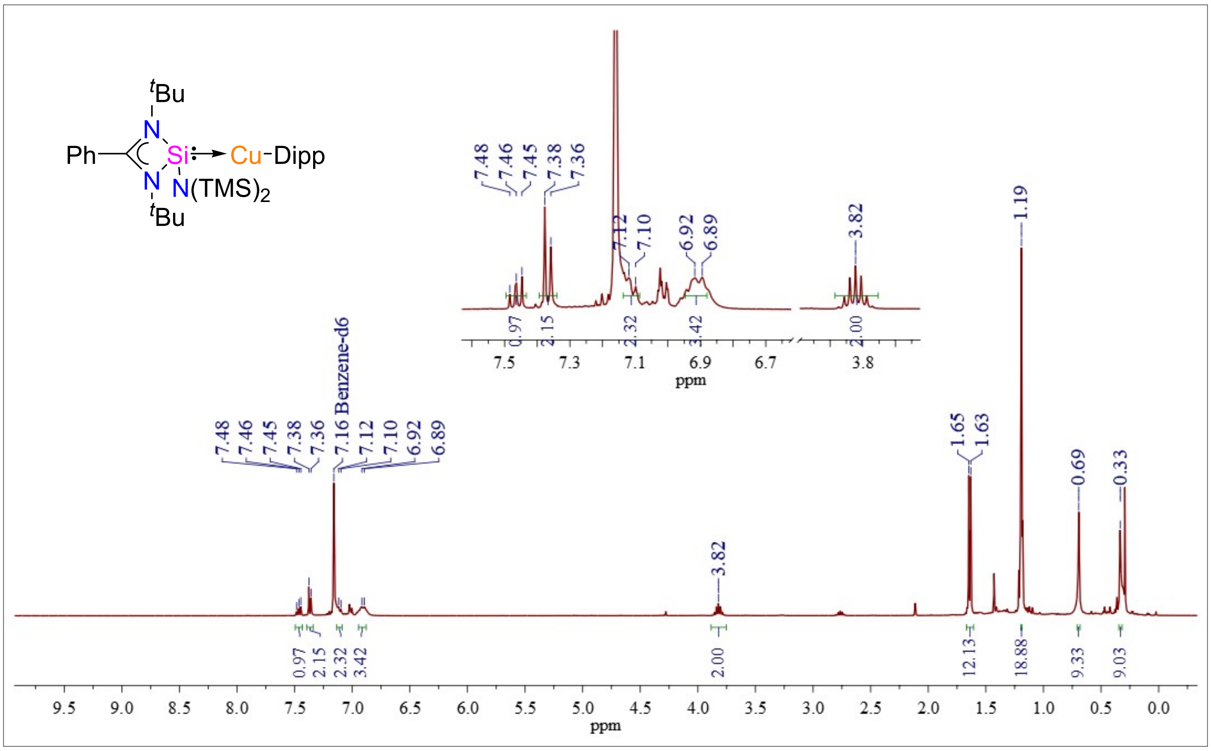

Figure S7. ^1H NMR spectrum of complex **4**.

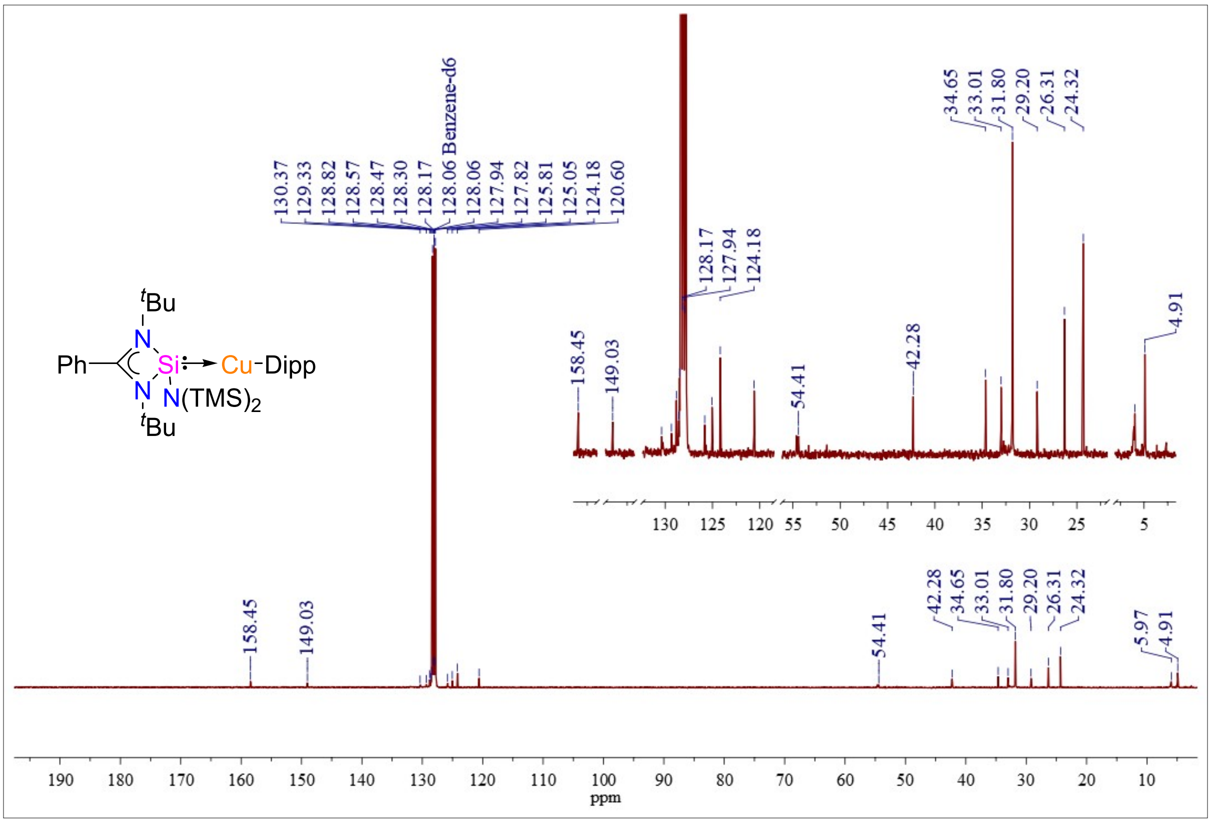

Figure S8. $^{13}C\{^{1}H\}$ NMR spectrum of complex **4**.

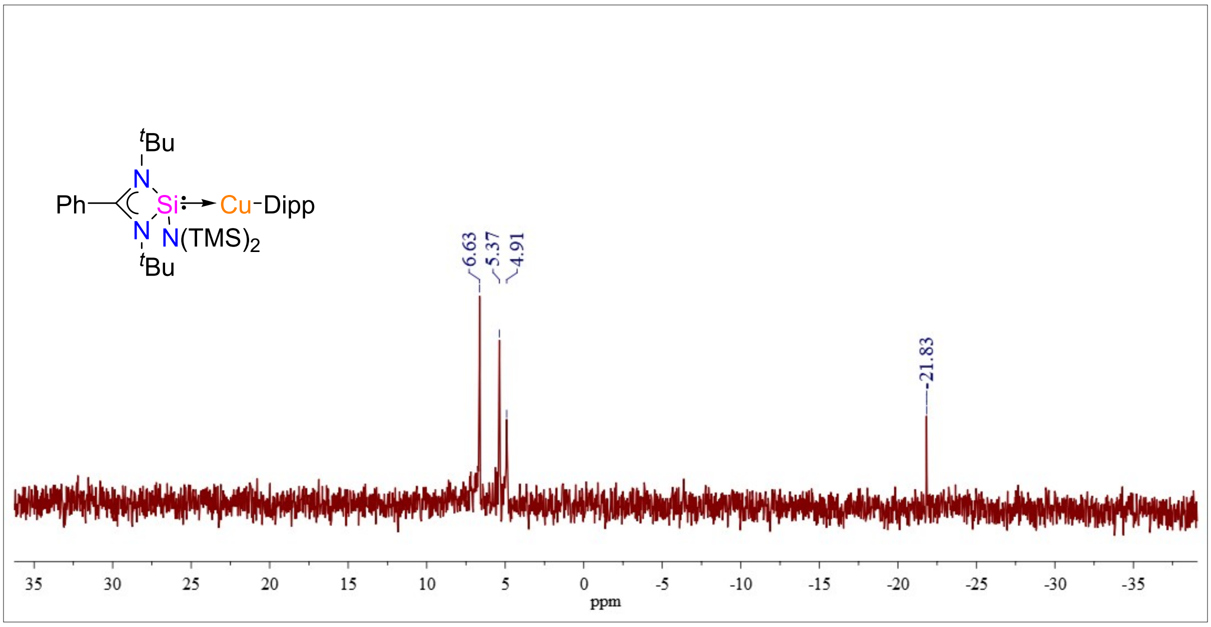

Figure S9. $^{29}Si\{^{1}H\}$ NMR spectrum of complex **4**.

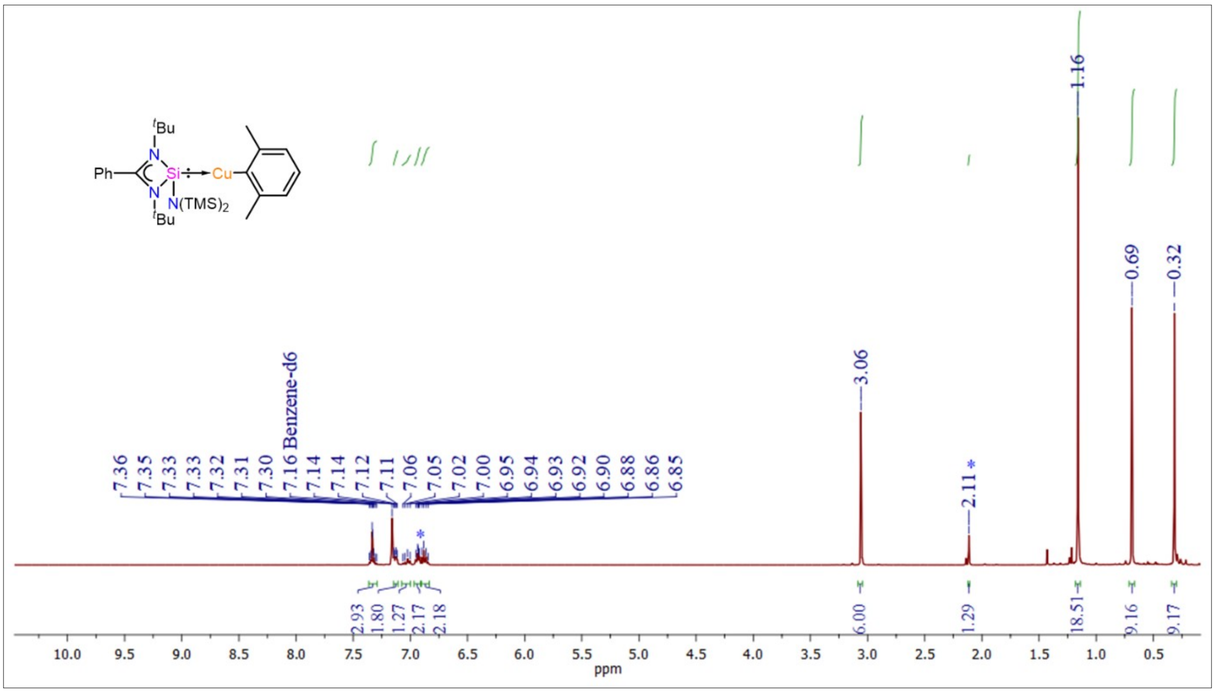

Figure S10. ^{1}H NMR spectrum of complex **5**. * residual toluene.

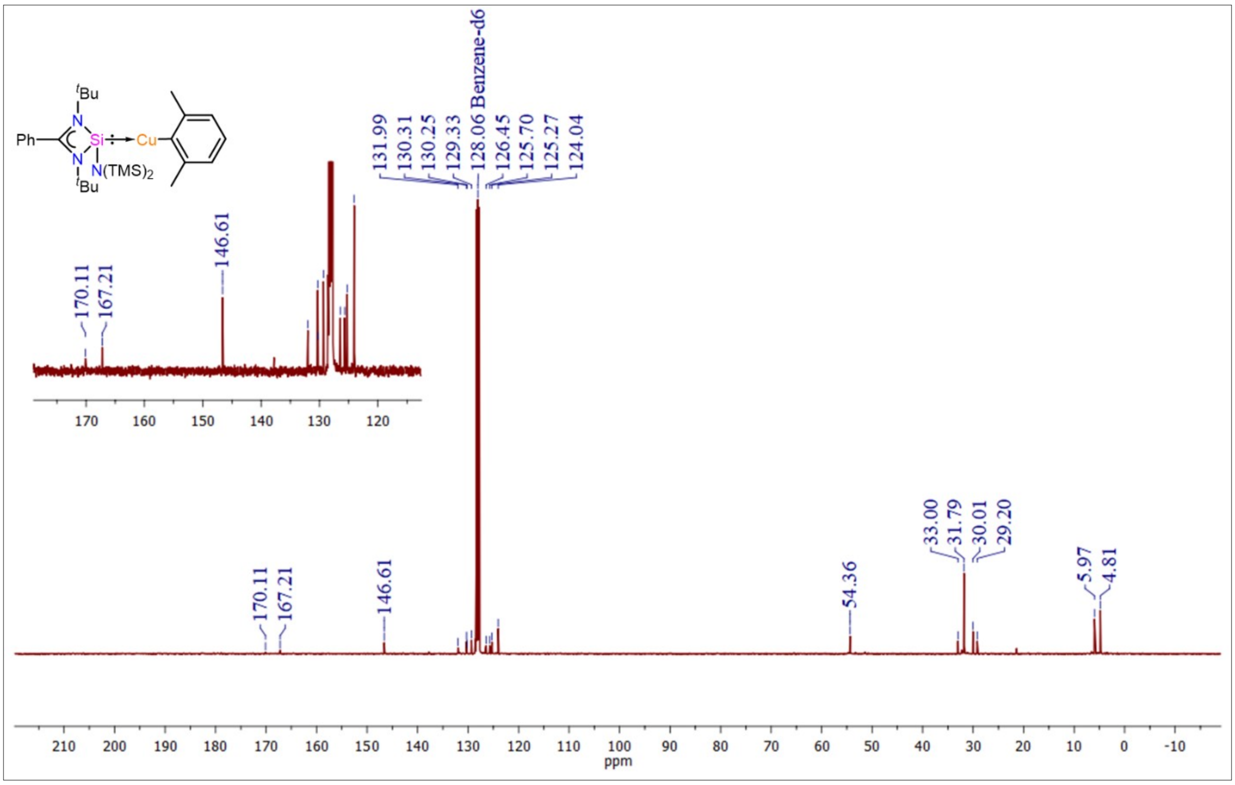

Figure S11. $^{13}C\{^1H\}$ NMR spectrum of complex **5**.

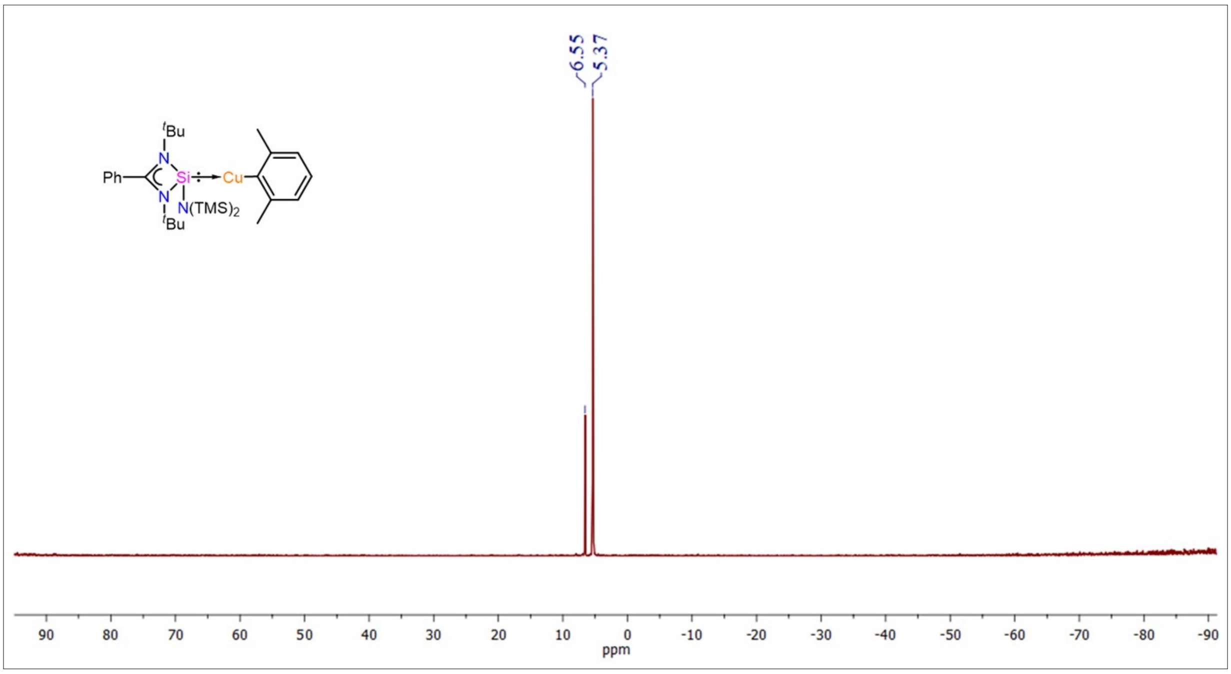

Figure S12. $^{29}Si\{^{1}H\}$ NMR spectrum of complex **5**.

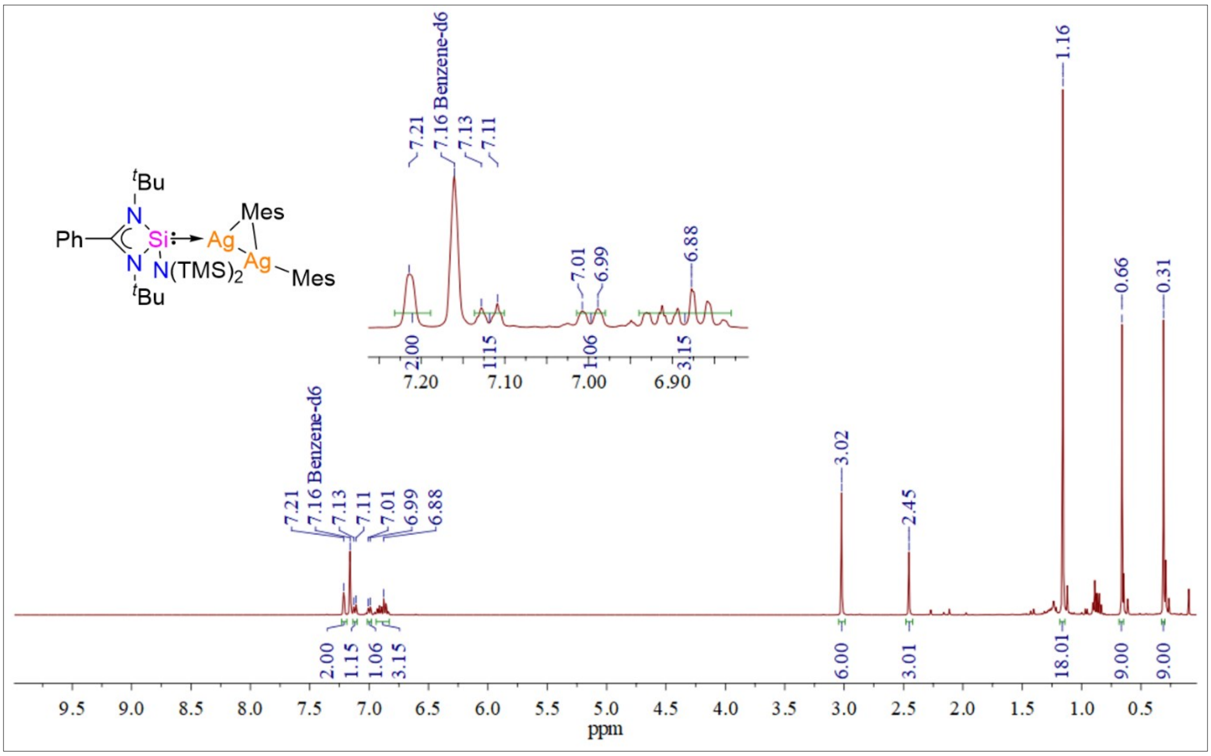

Figure S13. ^{1}H NMR spectrum of complex **6**.

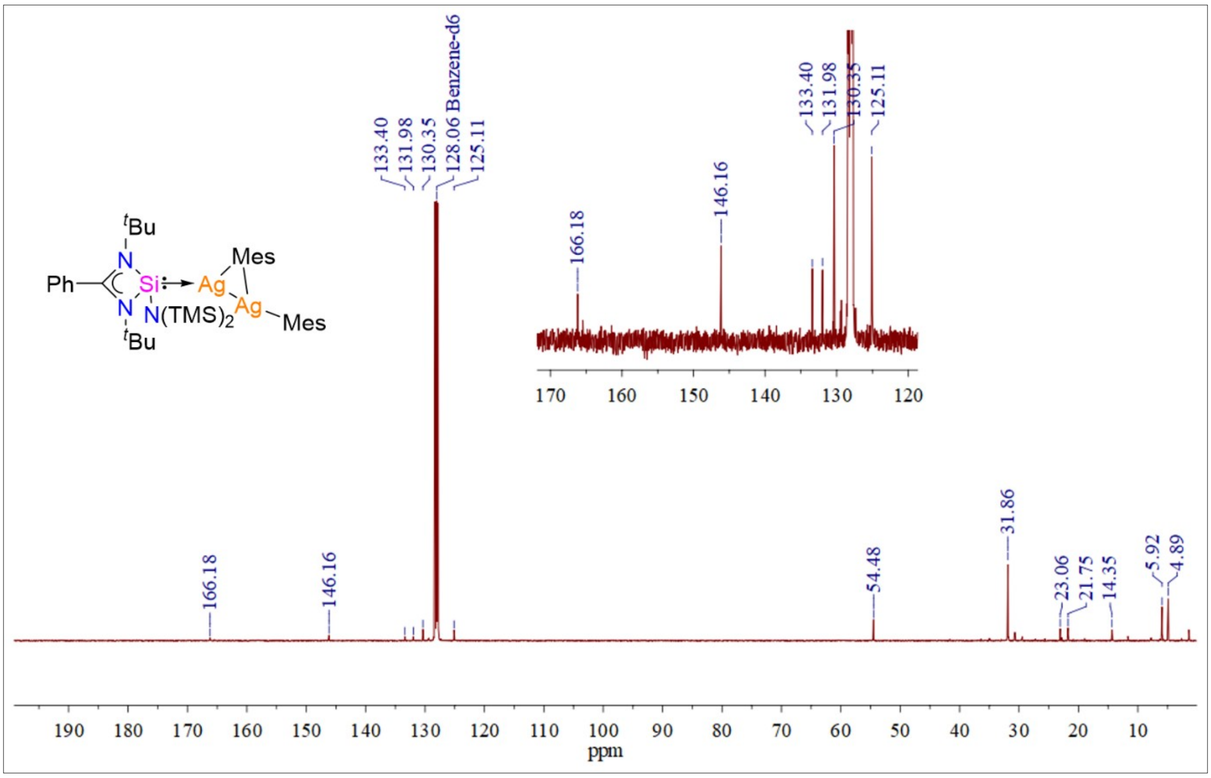

Figure S14. $^{13}C\{^1H\}$ NMR spectrum of complex **6**.

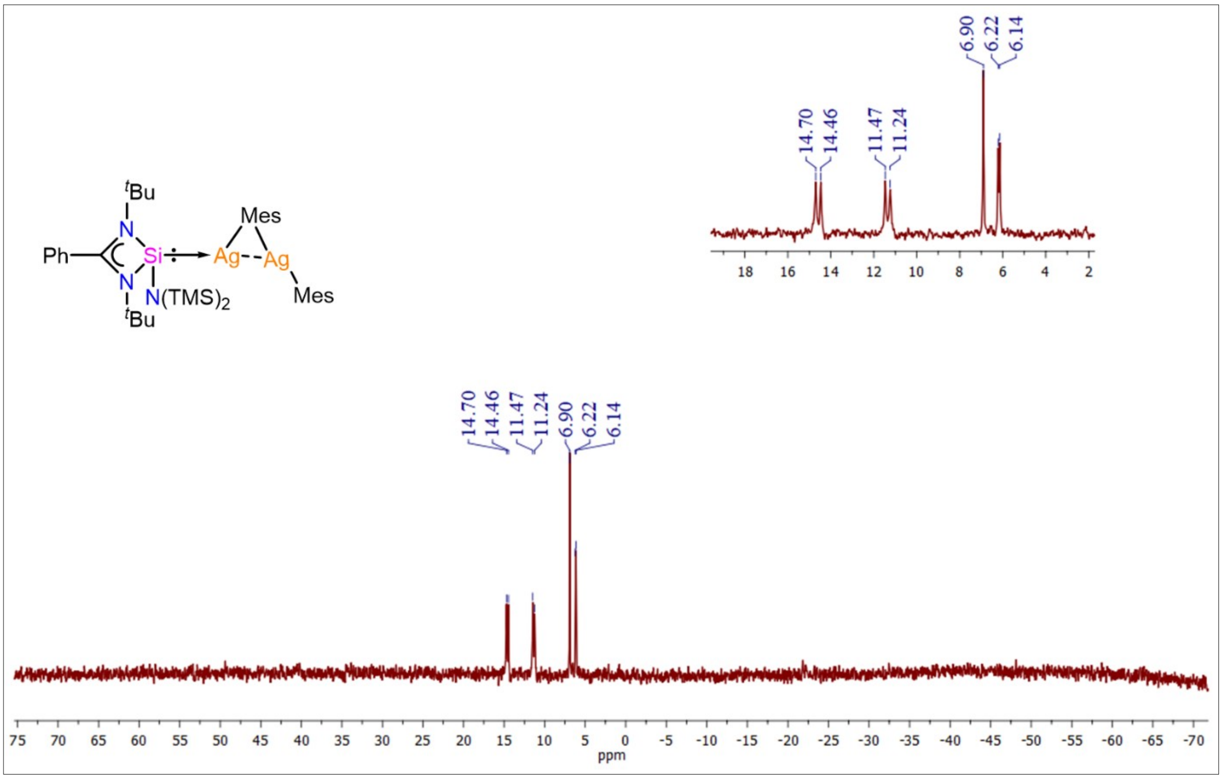

Figure S15. $^{29}Si\{^1H\}$ NMR spectrum of complex **6**.

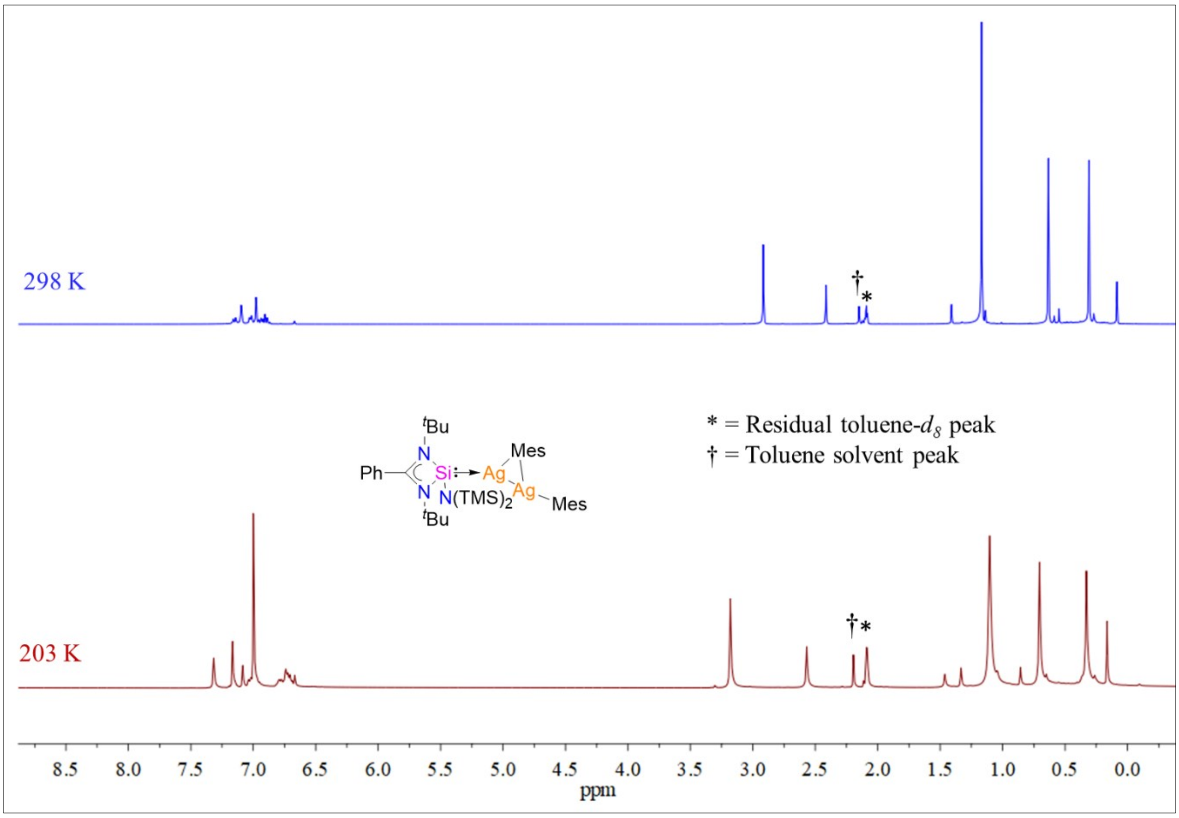

Figure S16. Variable temperature ^{1}H NMR spectra of complex **6** in toluene-d_8.

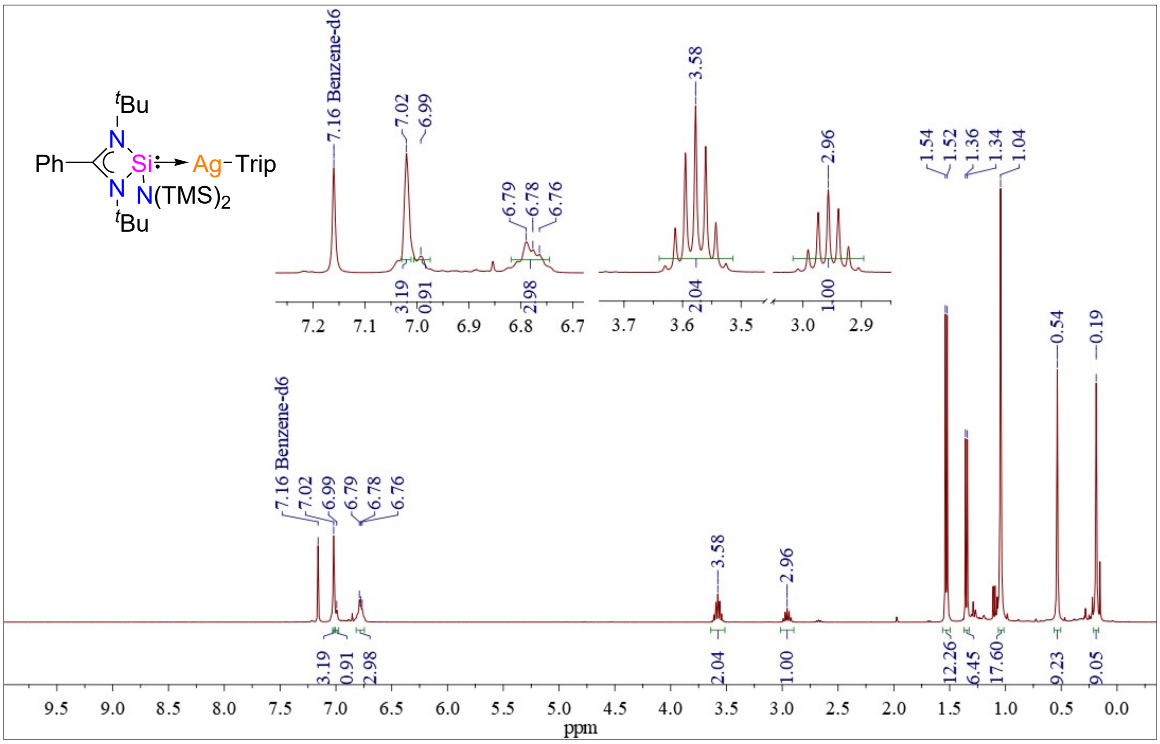

Figure S17. ^{1}H NMR spectrum of complex **7**.

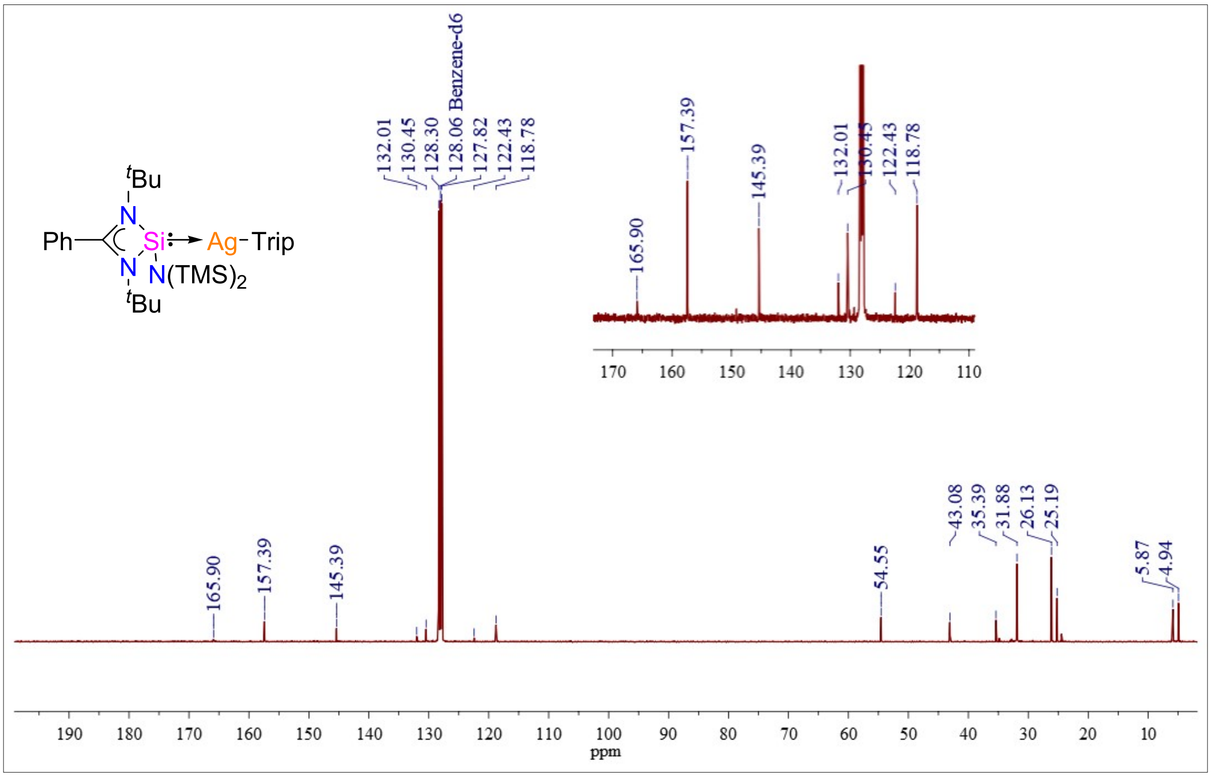

Figure S18. $^{13}C\{^1H\}$ NMR spectrum of complex **7**.

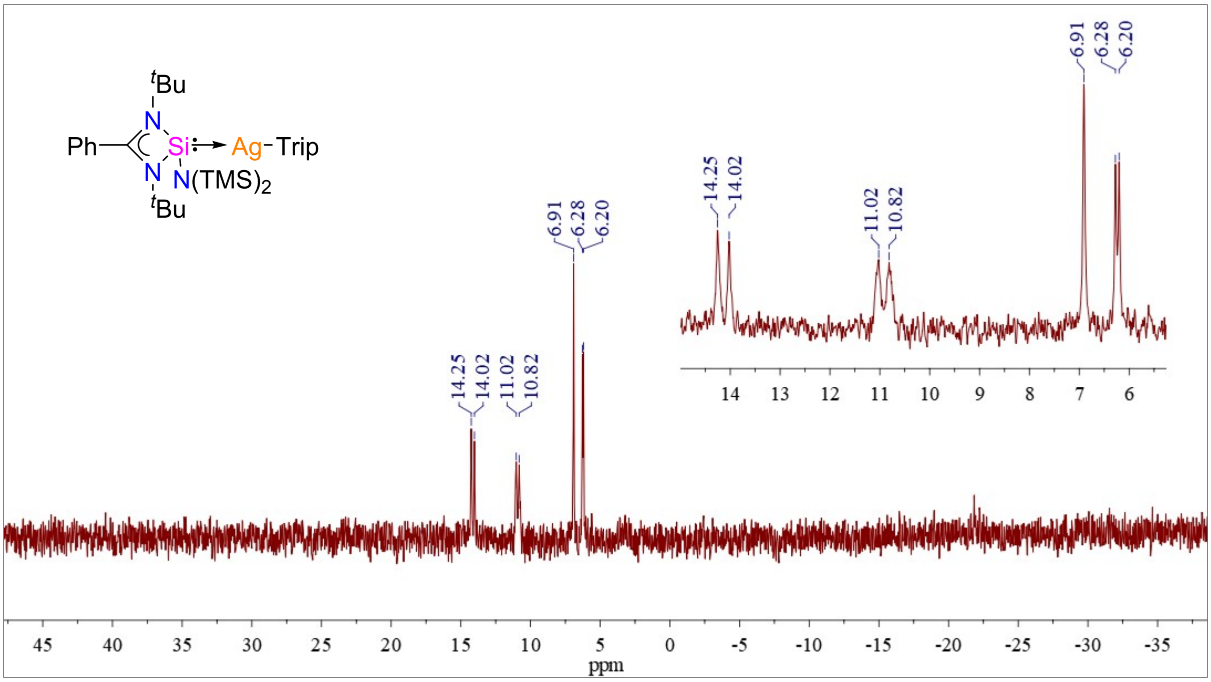

Figure S19. $^{29}Si\{^1H\}$ NMR spectrum of complex **7**.

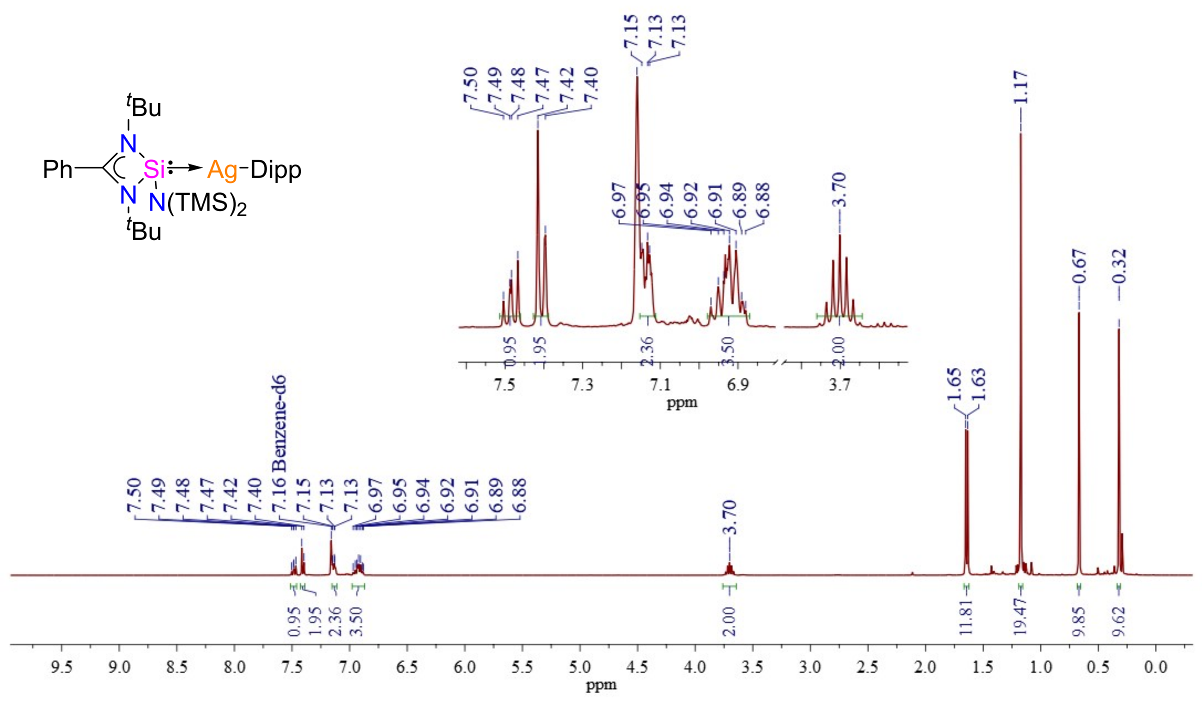

Figure S20. ^{1}H NMR spectrum of complex **8**.

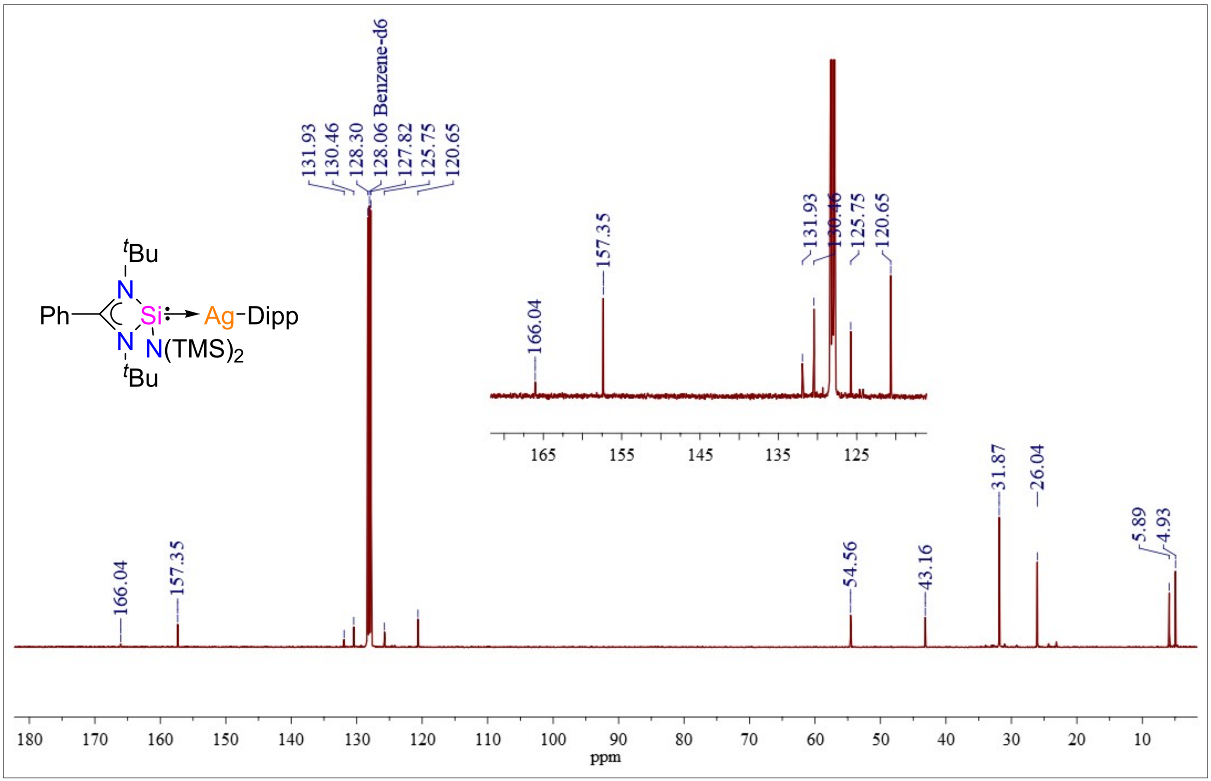

Figure S21. ^{13}C{^{1}H} NMR spectrum of complex **8**.

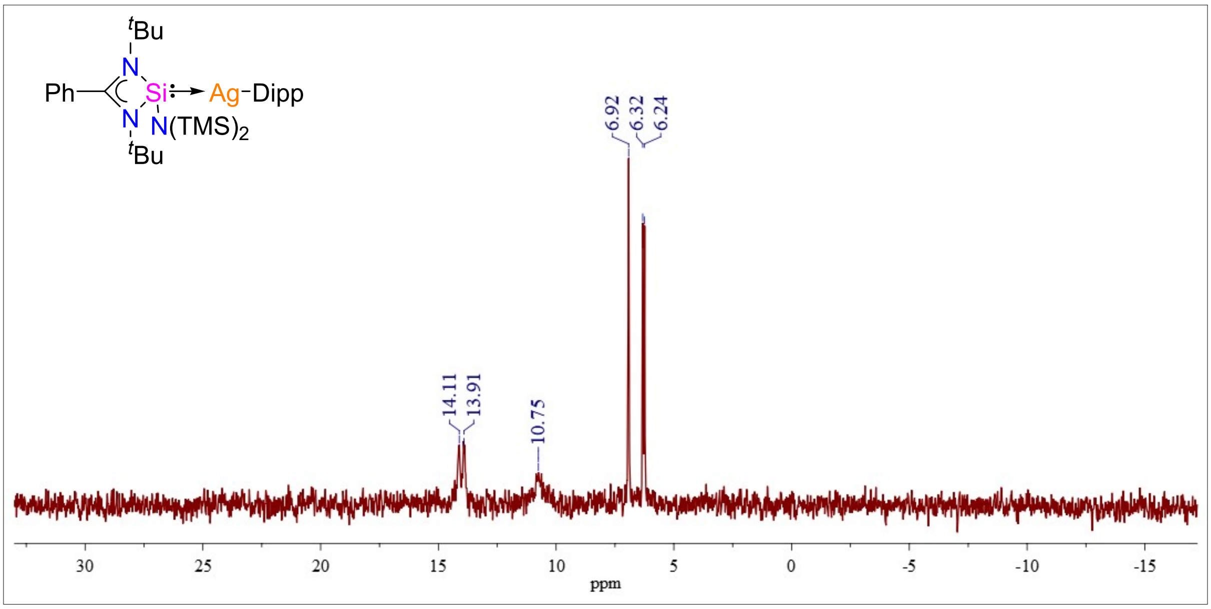

Figure S22. $^{29}Si\{^{1}H\}$ NMR spectrum of complex **8**.

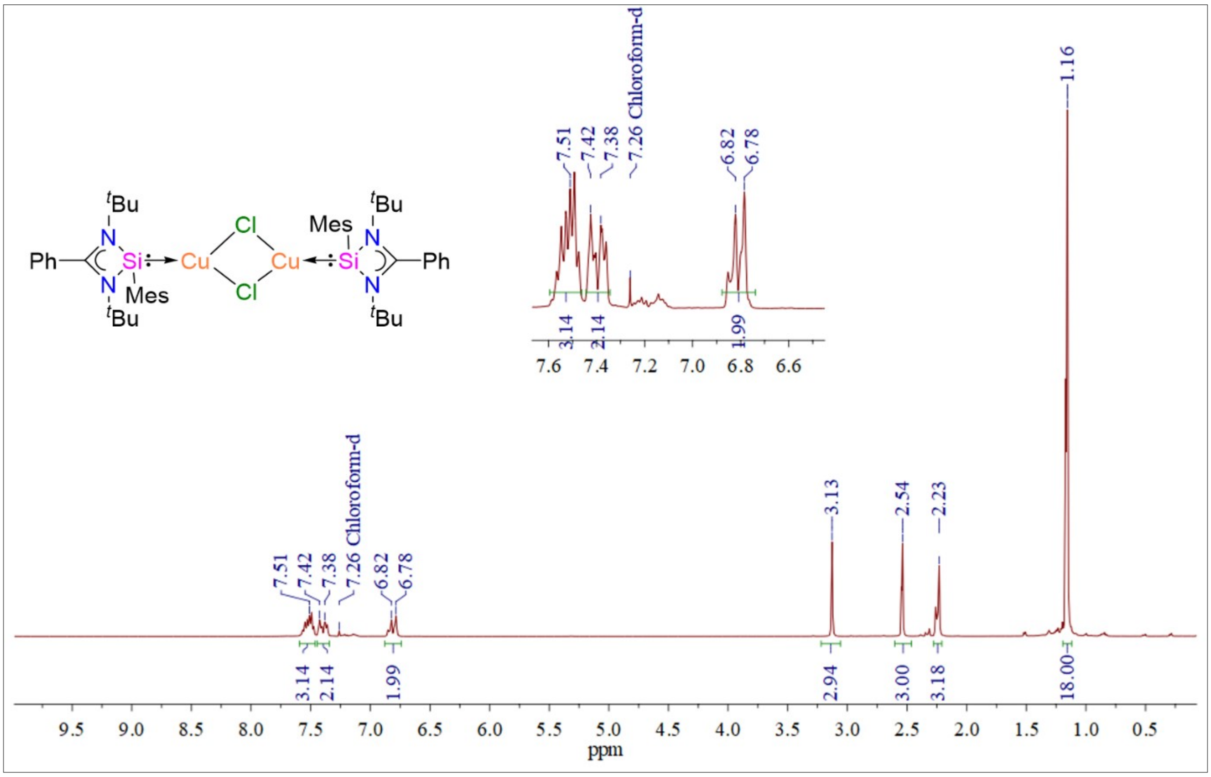

Figure S23. ^{1}H NMR spectrum of complex **9**.

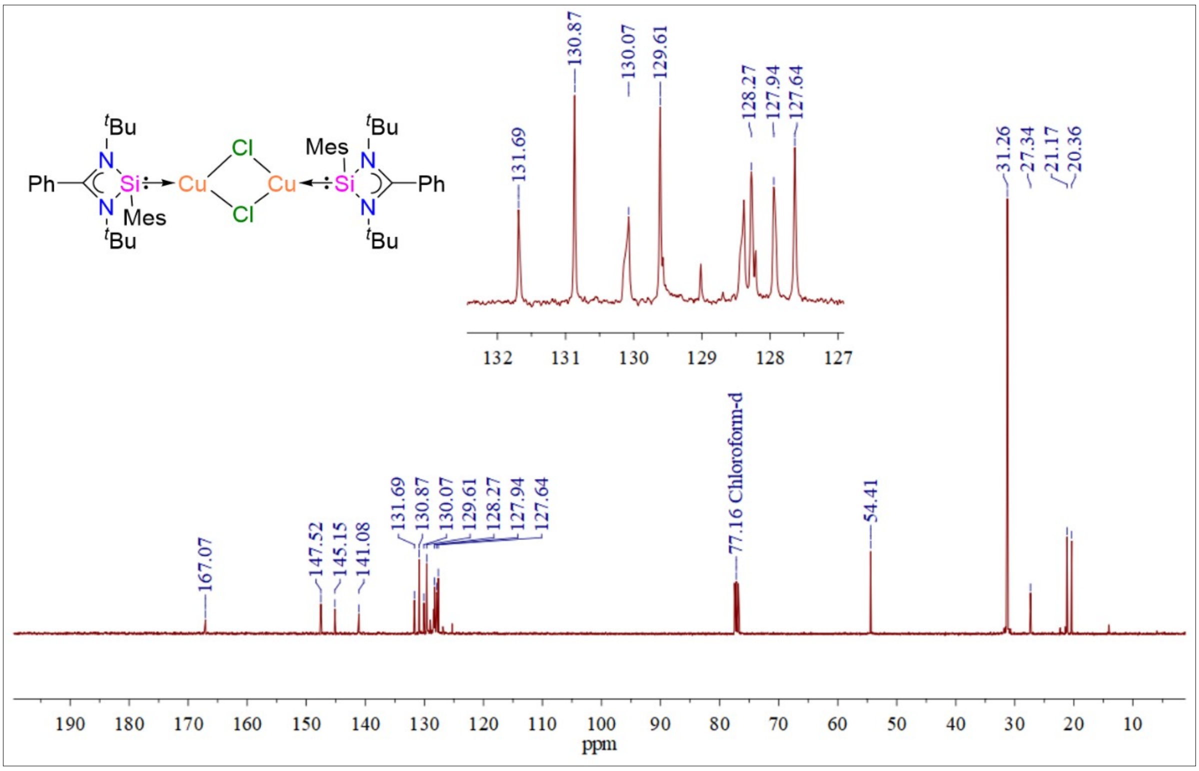

Figure S24. $^{13}C\{^1H\}$ NMR spectrum of complex **9**.

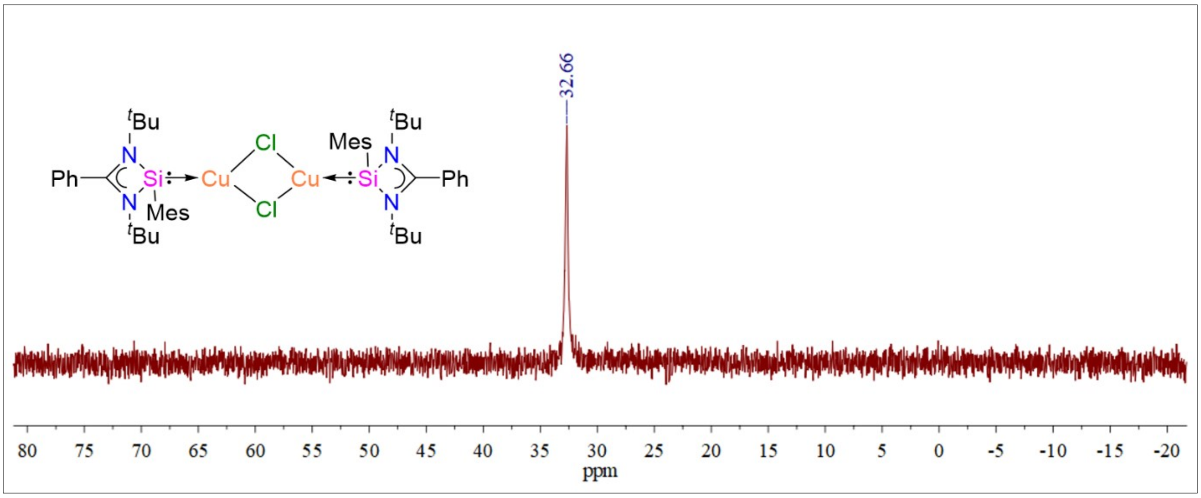

Figure S25. $^{29}Si\{^1H\}$ NMR spectrum of complex **9**.

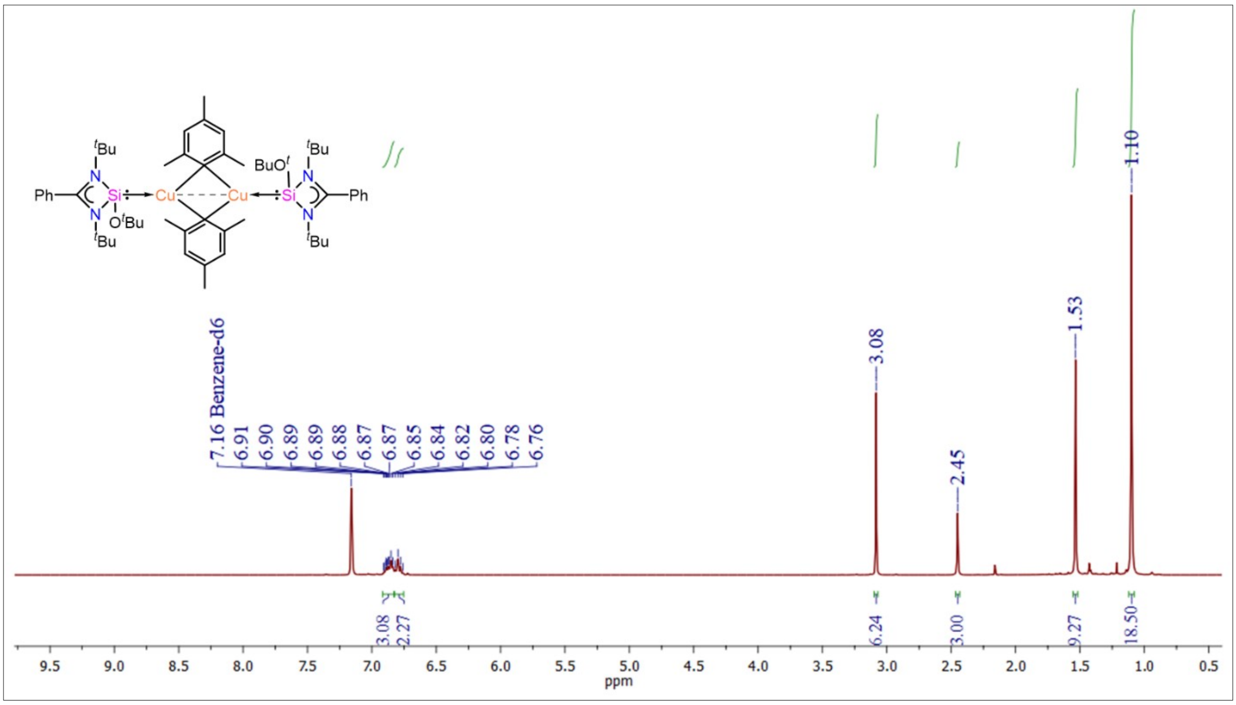

Figure S26. ^{1}H NMR spectrum of complex **10**.

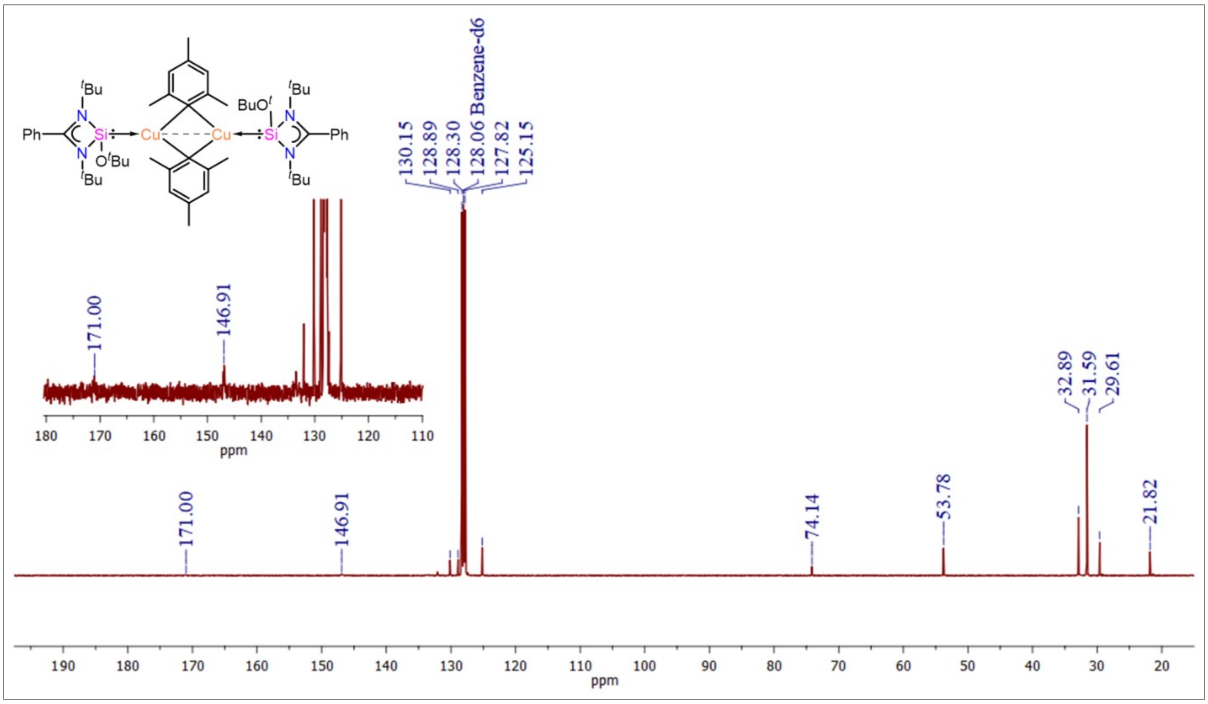

Figure S27. $^{13}C\{^{1}H\}$ NMR spectrum of complex **10**.

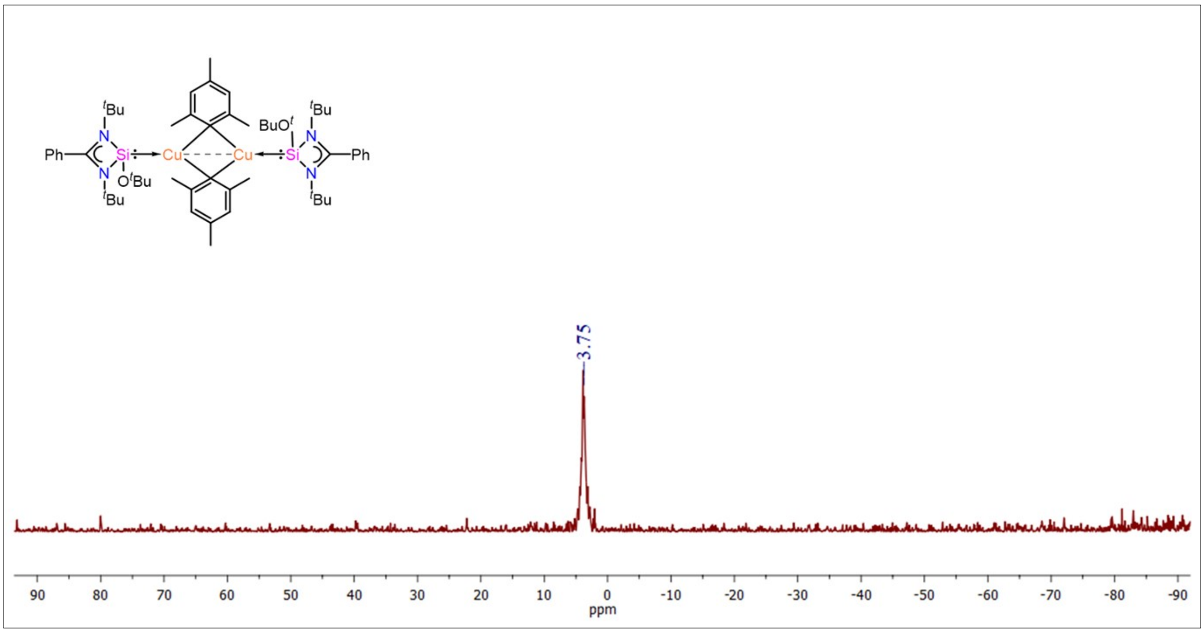

Figure S28. $^{29}Si\{^{1}H\}$ NMR spectrum of complex **10**.

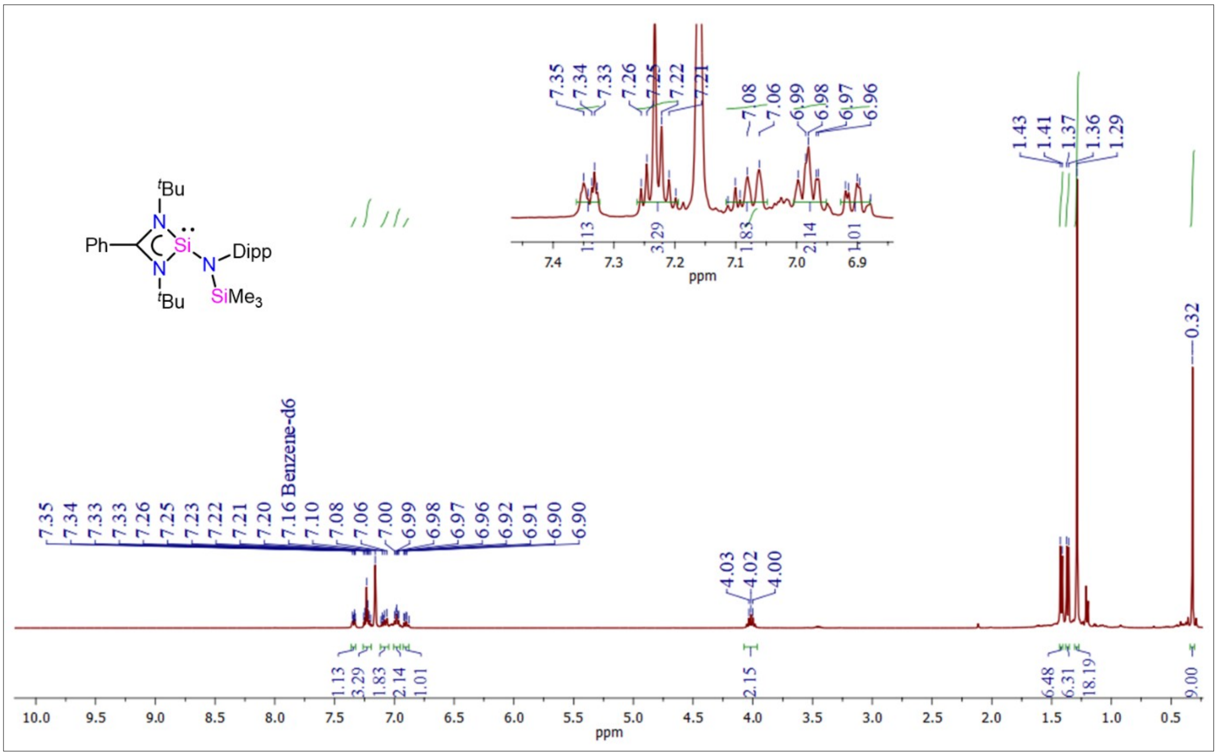

Figure S29. ^{1}H NMR spectrum of **[PhC{N(^{t}Bu)}$_2$Si{N(Dipp)SiMe$_3$}]**.

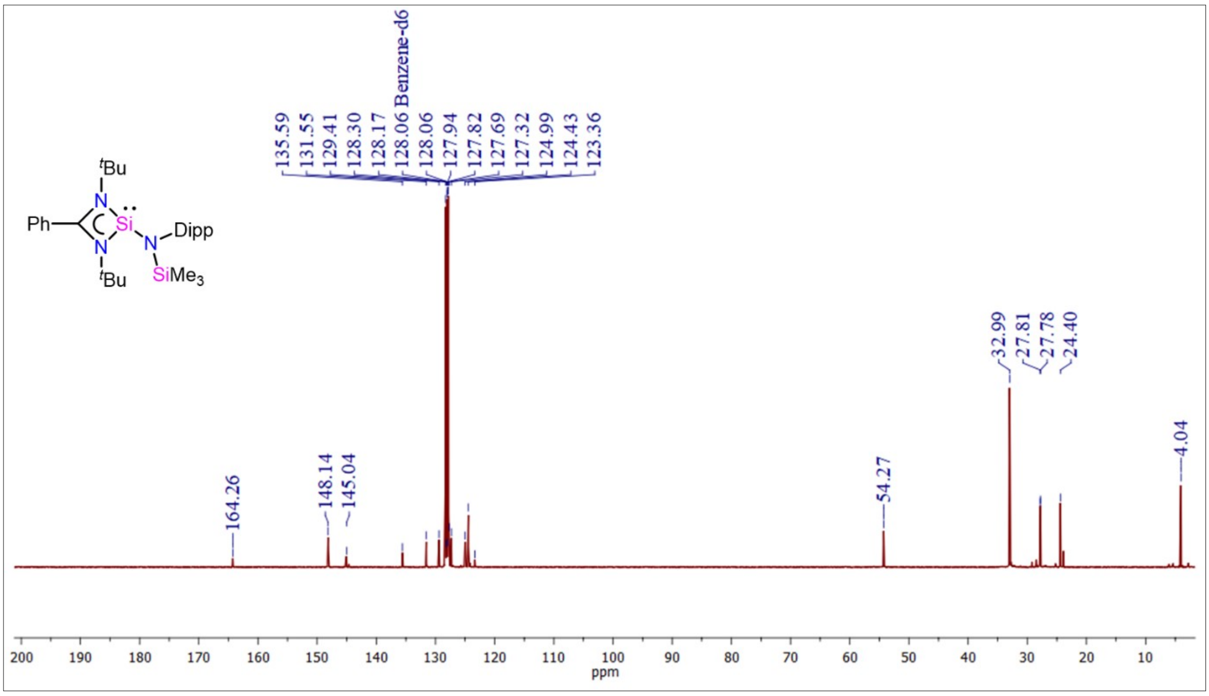

Figure S30. $^{13}C\{^{1}H\}$ NMR spectrum of complex **[PhC{N(^{t}Bu)}$_2$Si{N(Dipp)SiMe$_3$}]**.

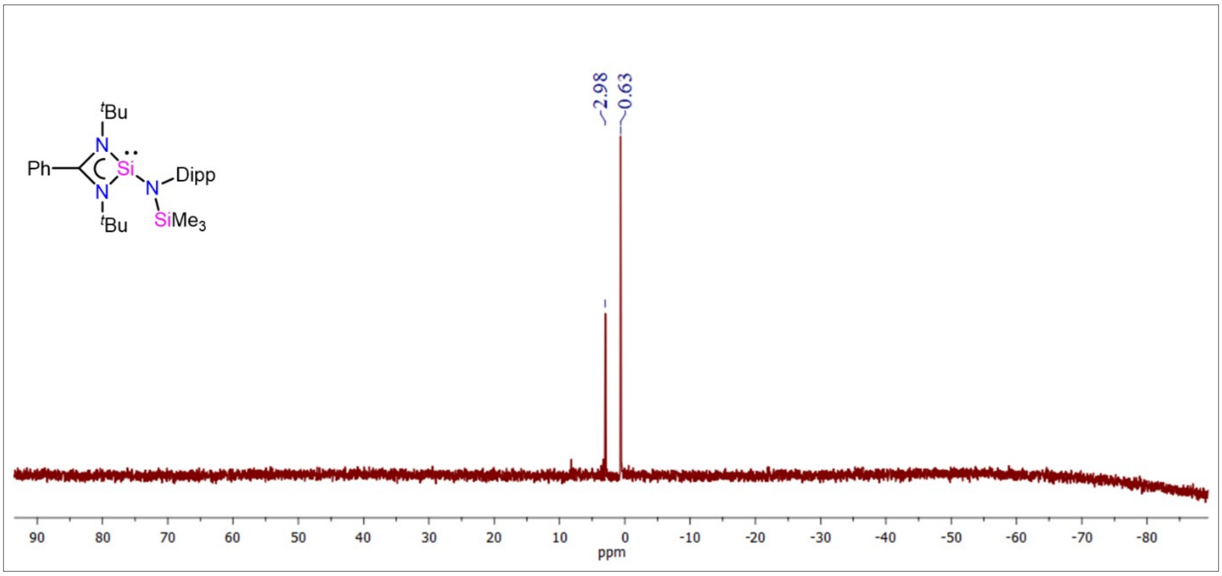

Figure S31. $^{29}Si\{^{1}H\}$ NMR spectrum of complex **[PhC{N(^{t}Bu)}$_2$Si{N(Dipp)SiMe$_3$}]**.

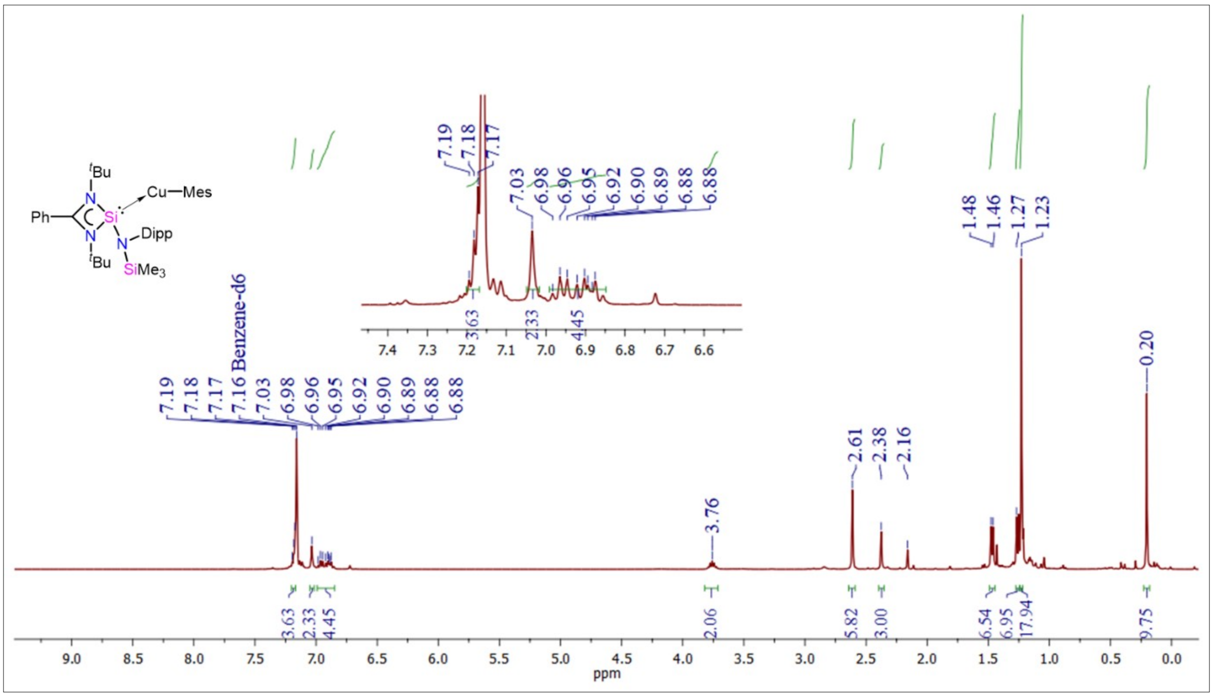

Figure S32. ^{1}H NMR spectrum of complex **11**.

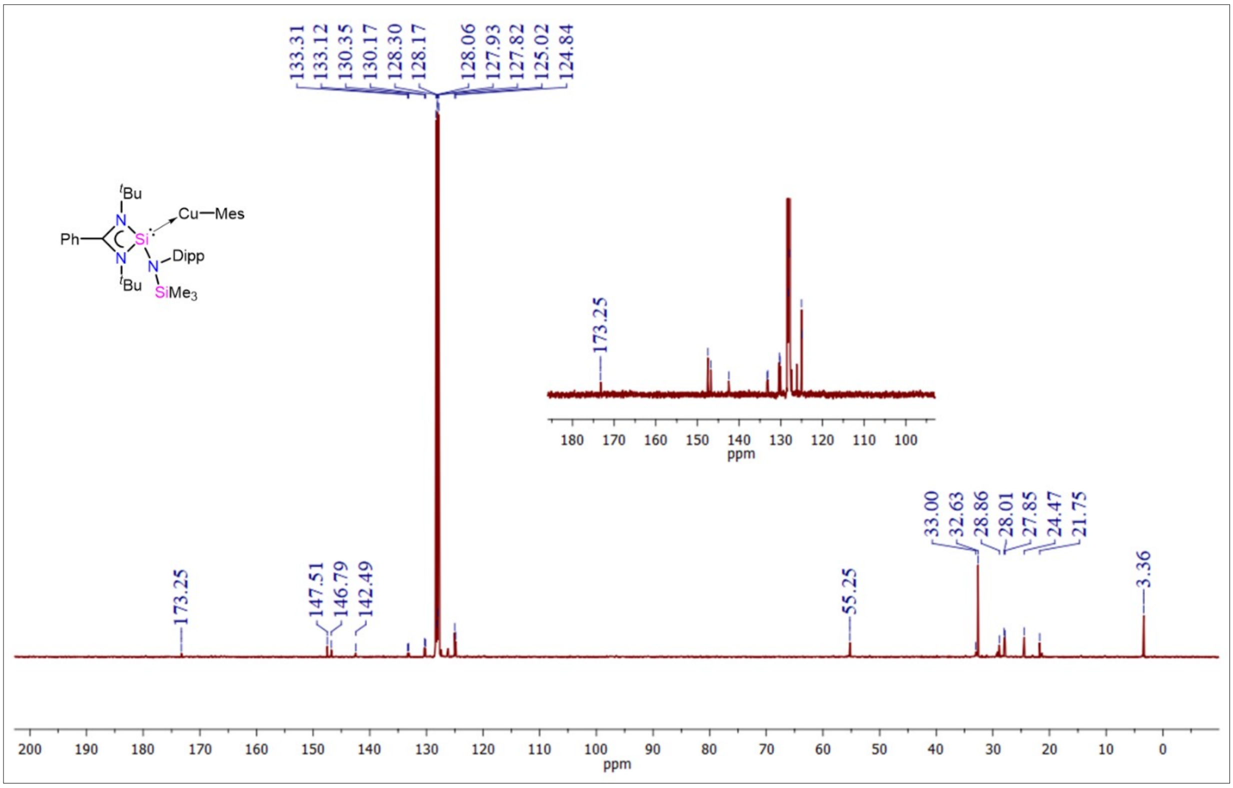

Figure S33. $^{13}C\{^{1}H\}$ NMR spectrum of complex **11**.

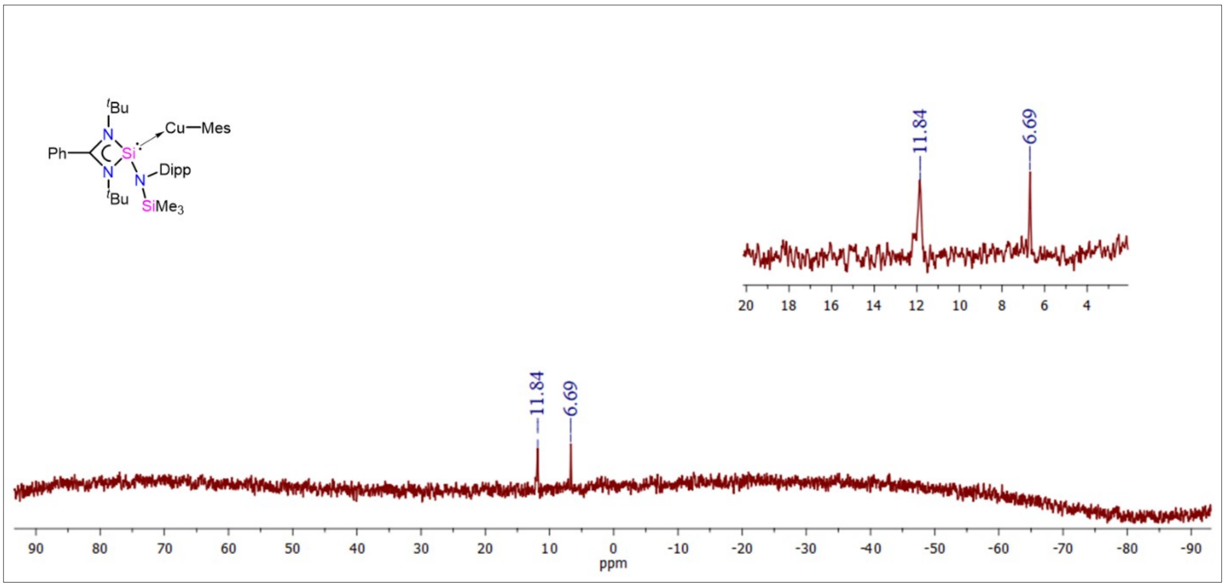

Figure S34. $^{29}Si\{^1H\}$ NMR spectrum of complex **11**.

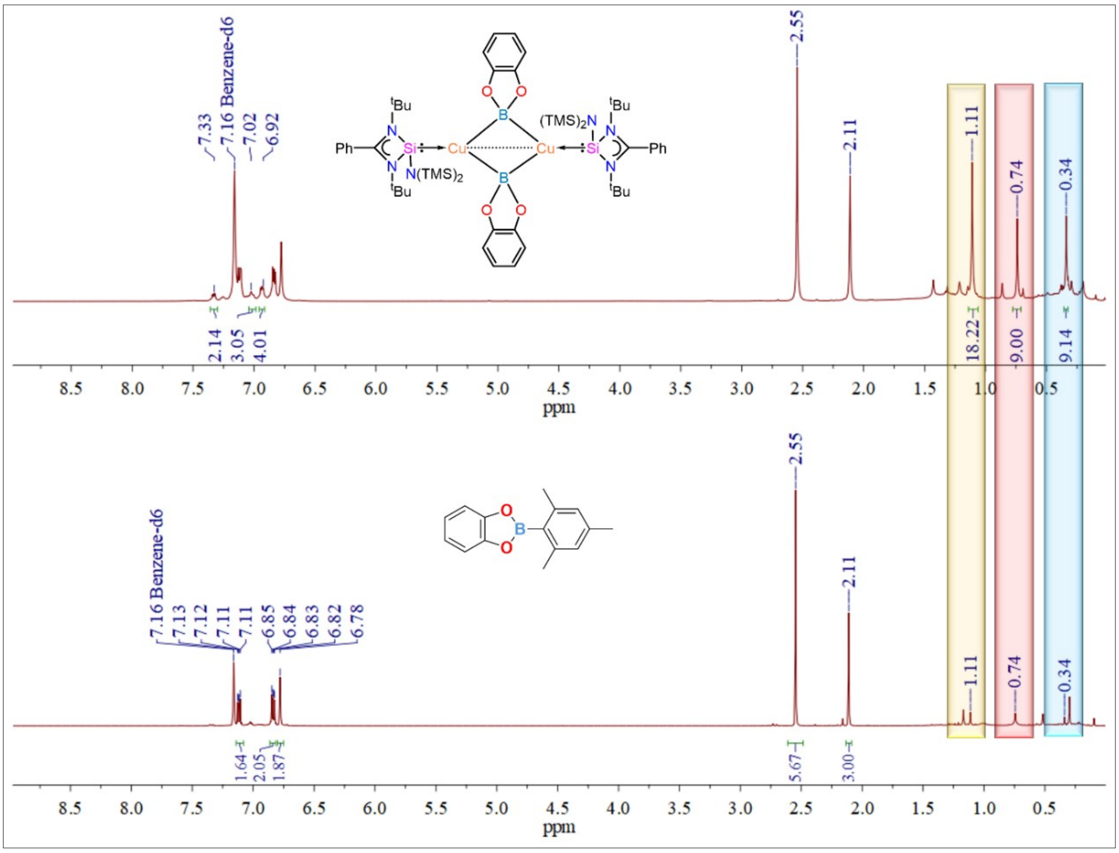

Figure S35. ^1H NMR spectrum of complexes **12** and **13**.

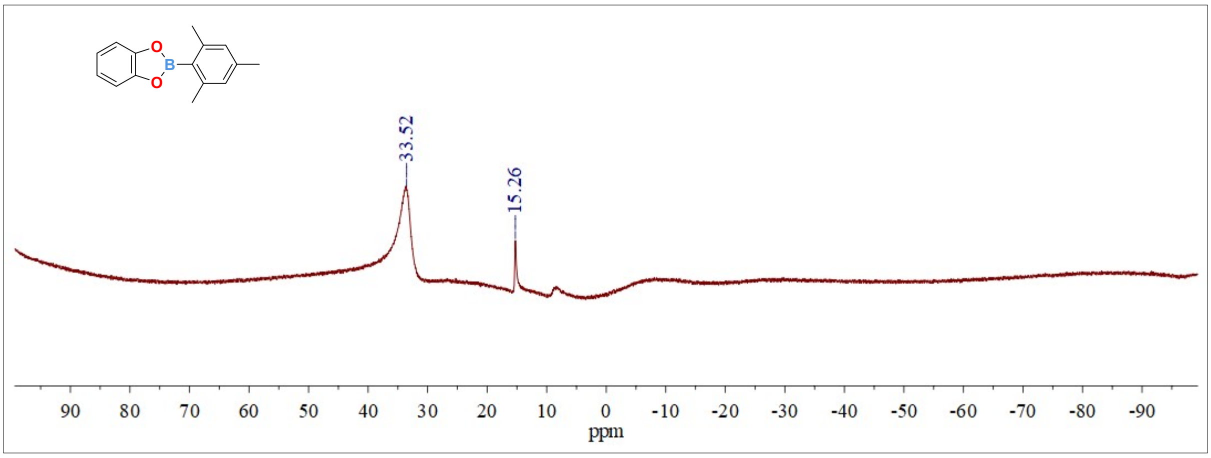

Figure S36. $^{11}B\{^1H\}$ NMR spectrum of complexes **12** and **13**.

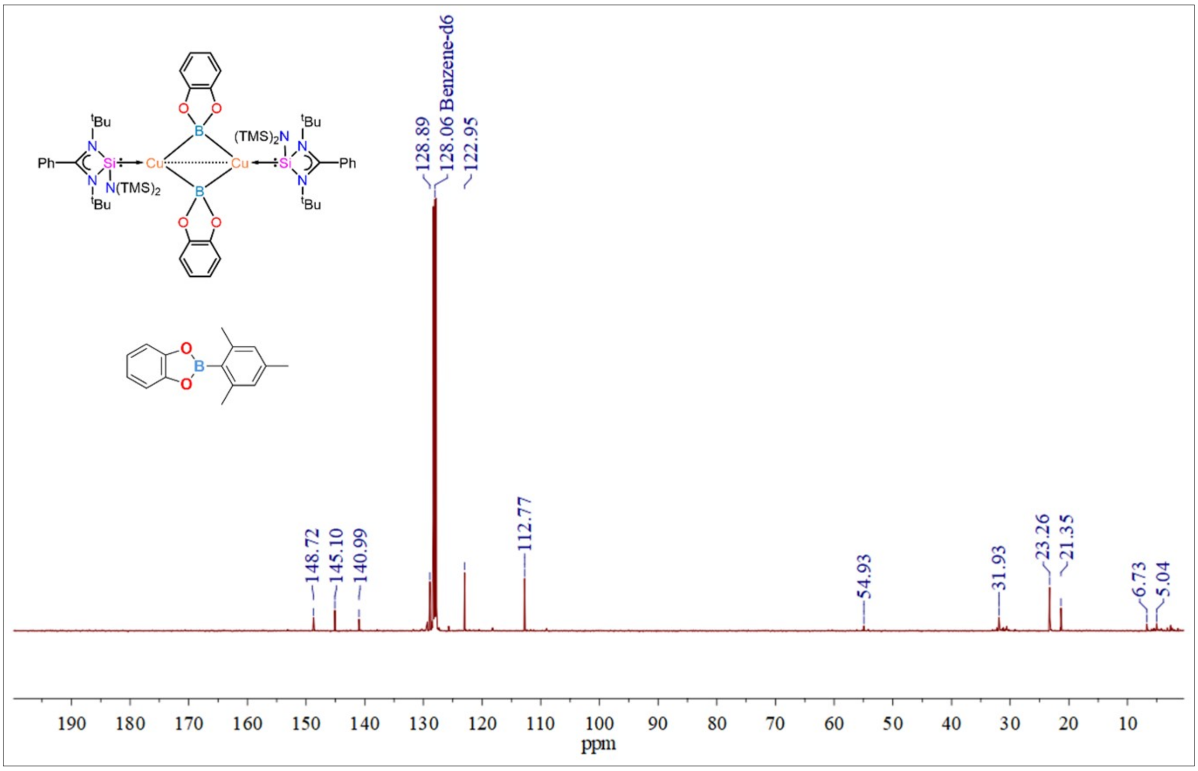

Figure S37. $^{13}C\{^1H\}$ NMR spectrum of complexes **12** and **13**.

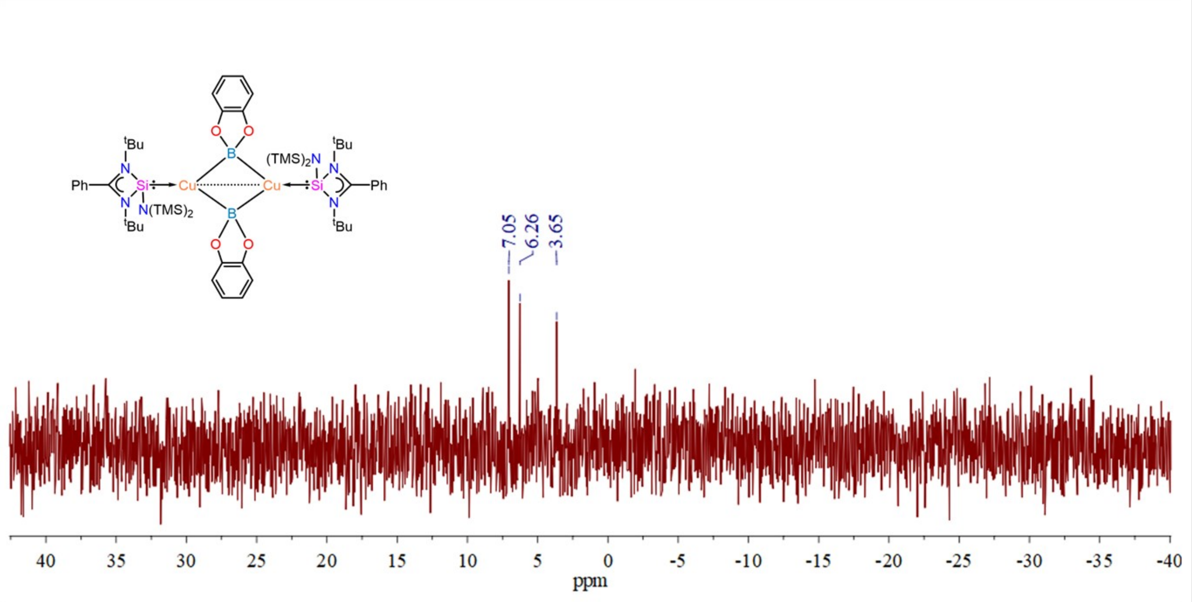

Figure S38. $^{29}Si\{^{1}H\}$ NMR spectrum of complex **12**.

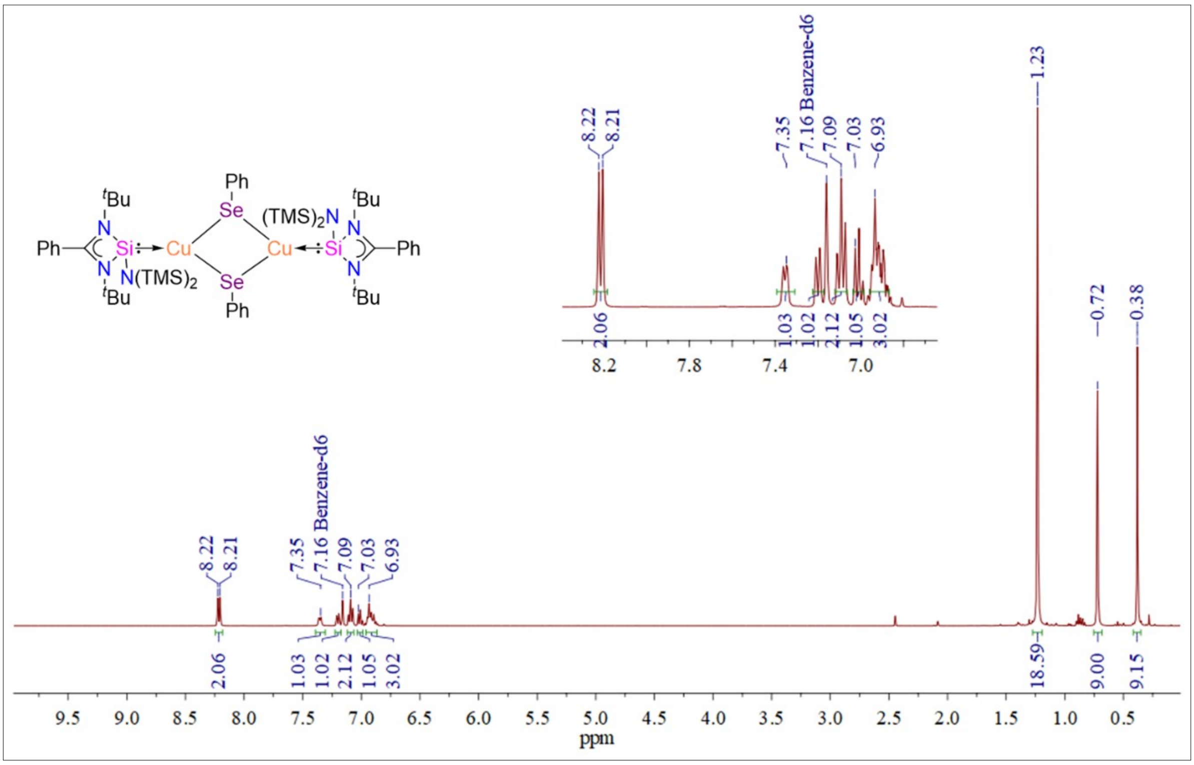

Figure S39. ^{1}H NMR spectrum of complex **14**.

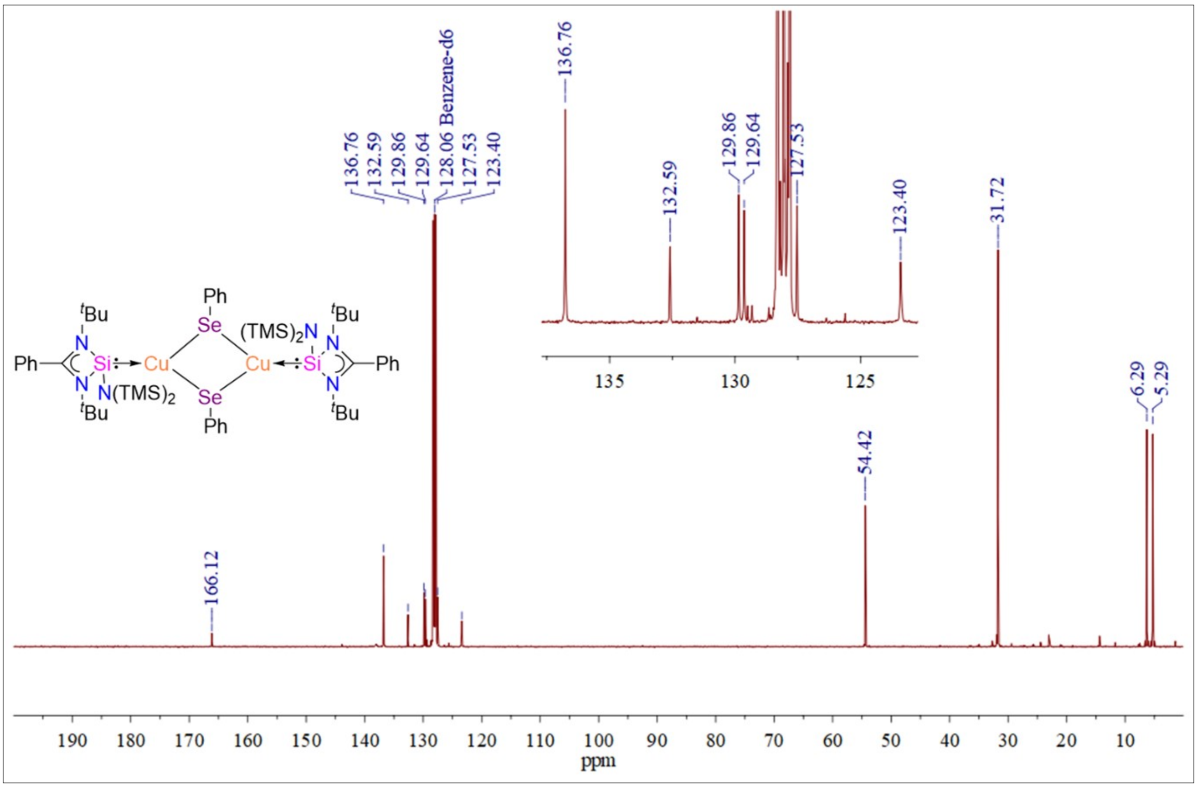

Figure S40. $^{13}C\{^1H\}$ NMR spectrum of complex **14**.

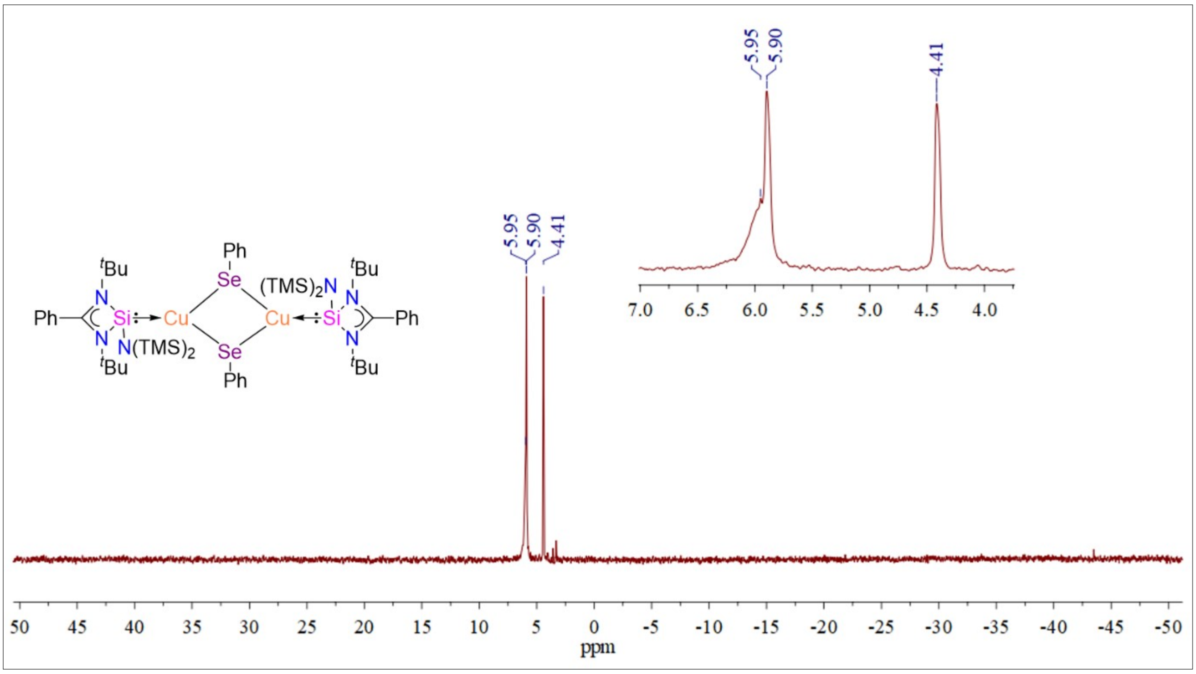

Figure S41. $^{29}Si\{^1H\}$ NMR spectrum of complex **14**.

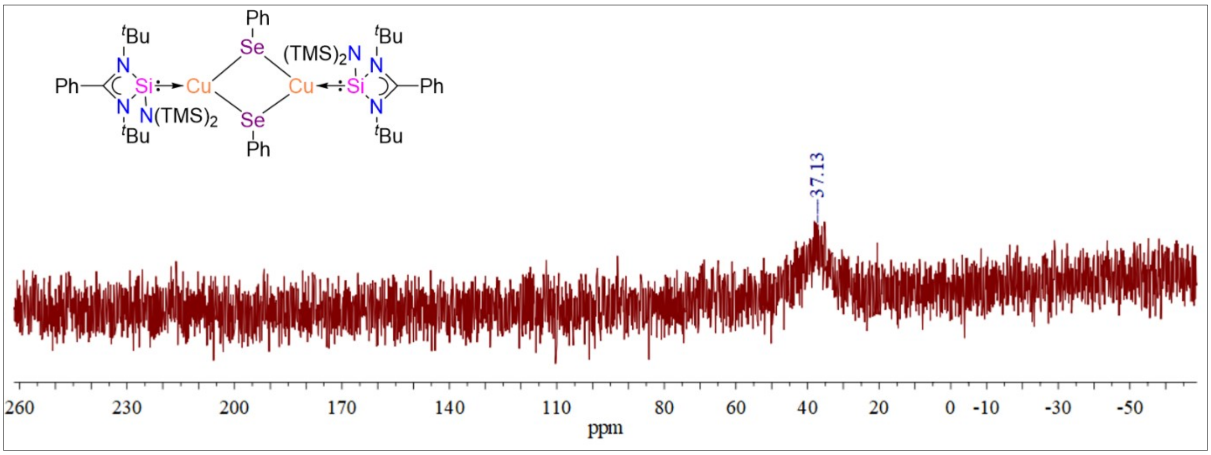

Figure S42. ^{77}Se{^{1}H} NMR spectrum of complex **14**.

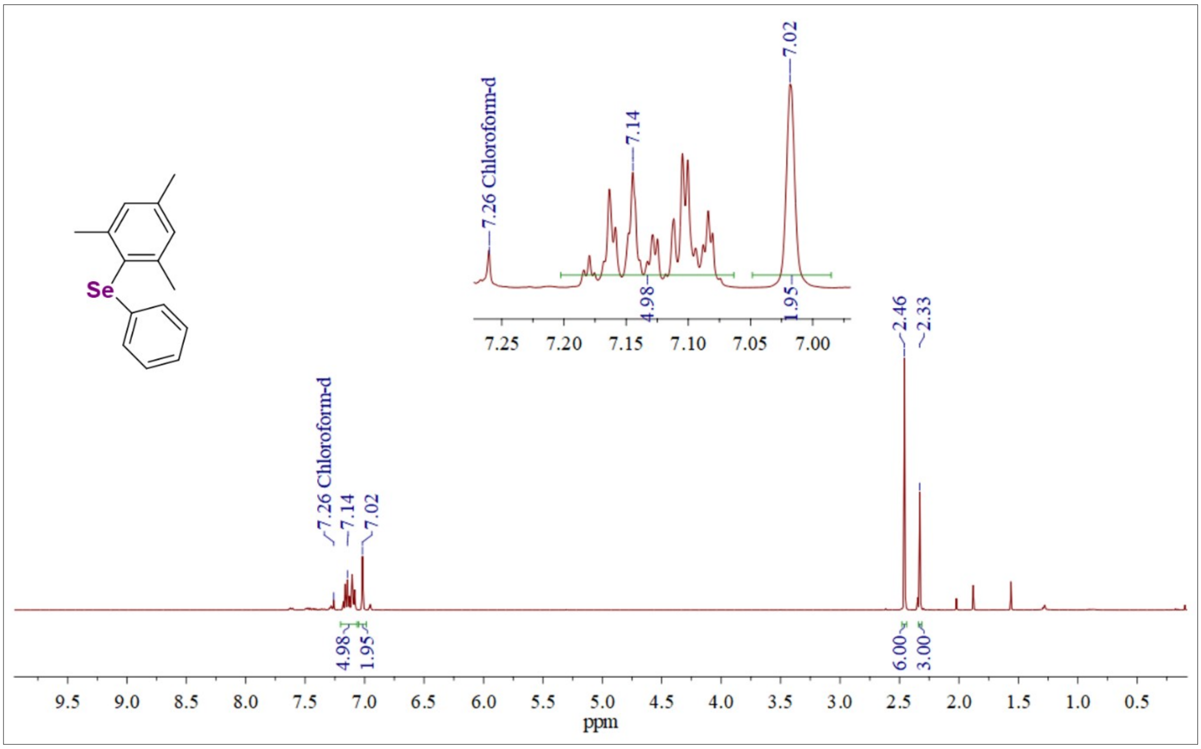

Figure S43. ^{1}H NMR spectrum of **Mesityl(phenyl)selane**.

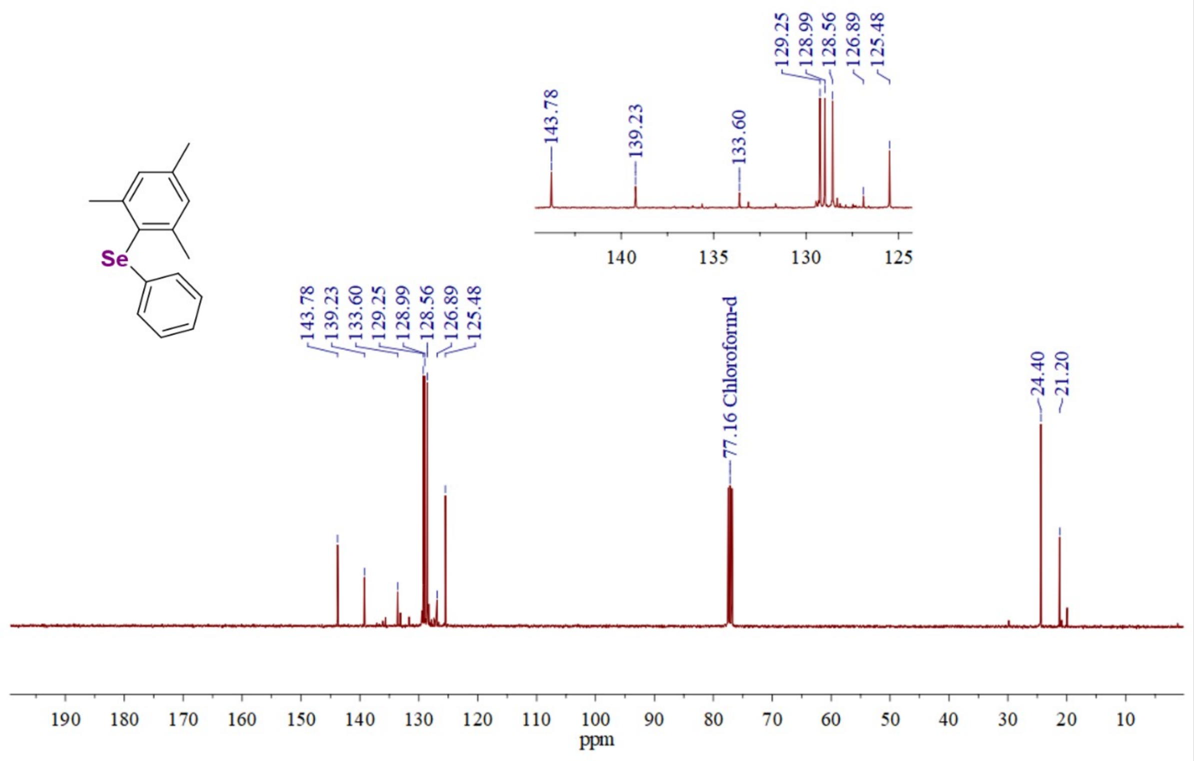

Figure S44. $^{13}C\{^{1}H\}$ NMR spectrum of **Mesityl(phenyl)selane**.

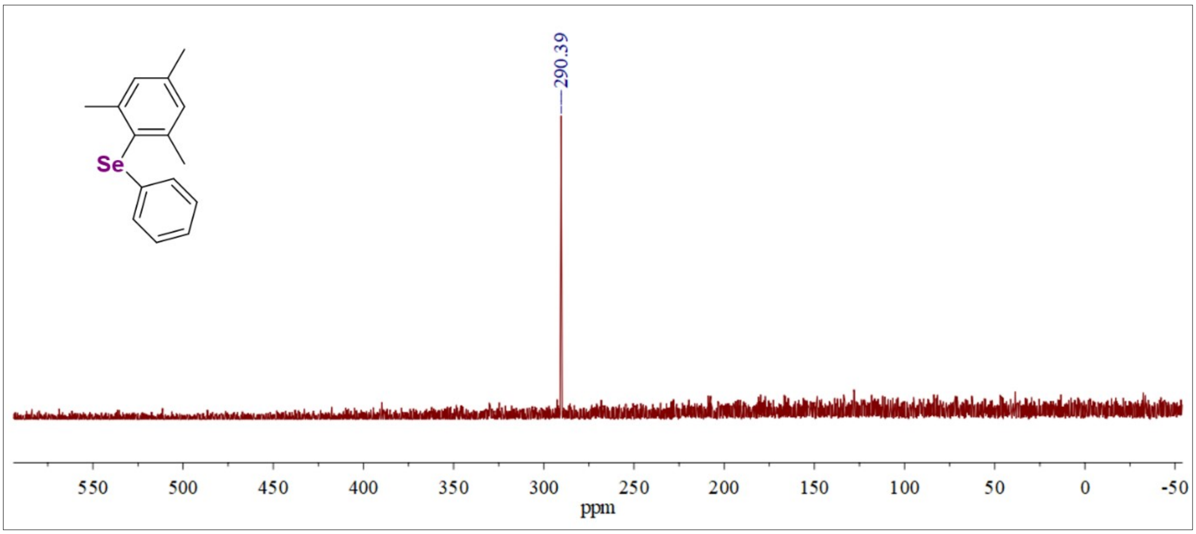

Figure S45. $^{77}Se\{^{1}H\}$ NMR spectrum of **Mesityl(phenyl)selane**.

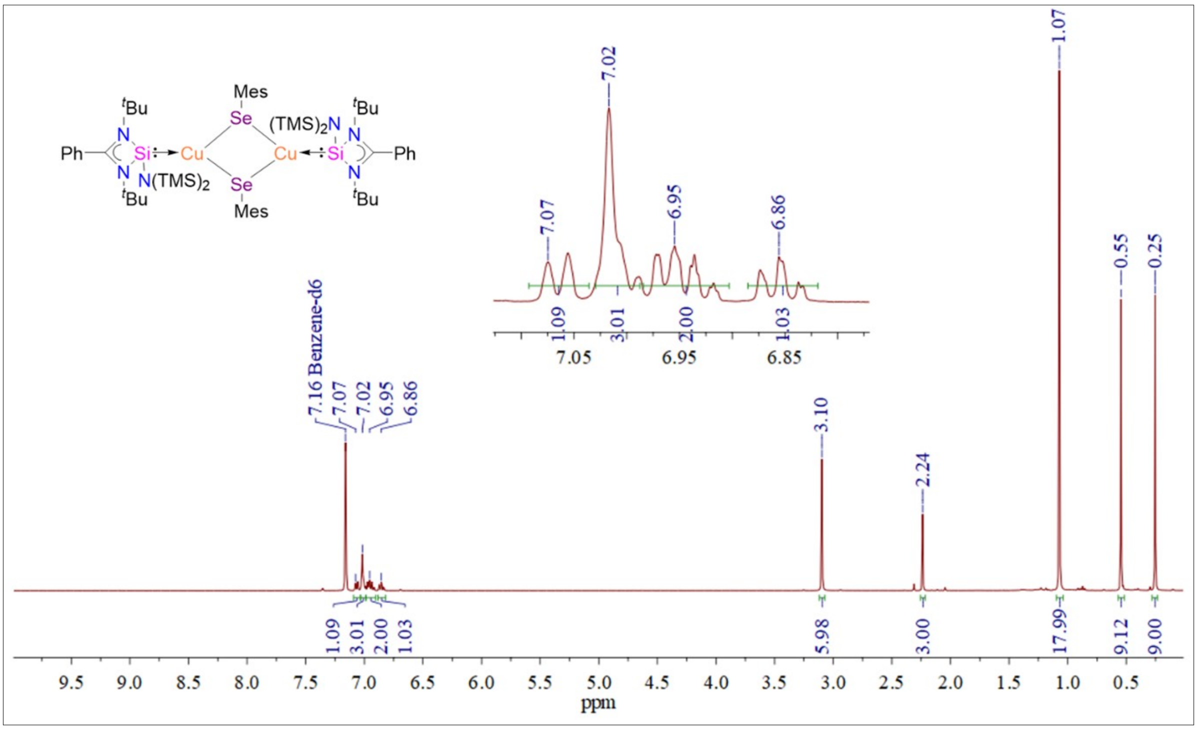

Figure S46. ^{1}H NMR spectrum of complex **15**.

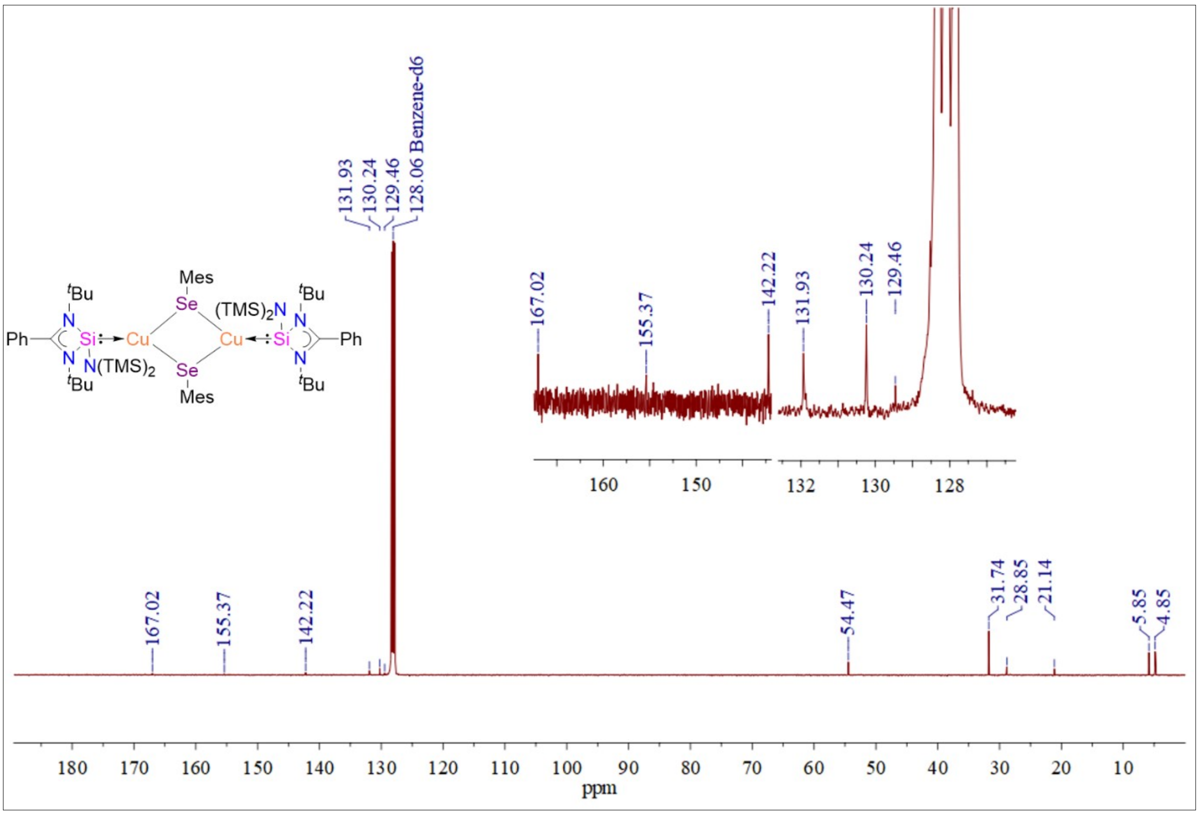

Figure S47. $^{13}C\{^{1}H\}$ NMR spectrum of complex **15**.

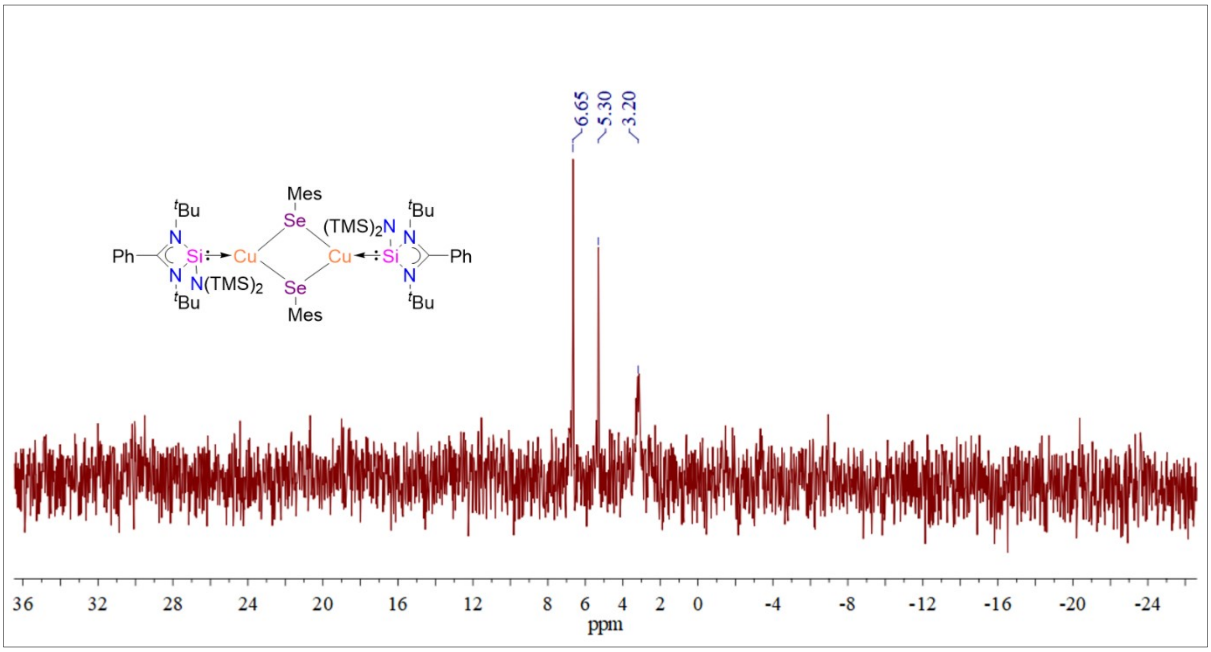

Figure S48. $^{29}Si\{^{1}H\}$ NMR spectrum of complex **15**.

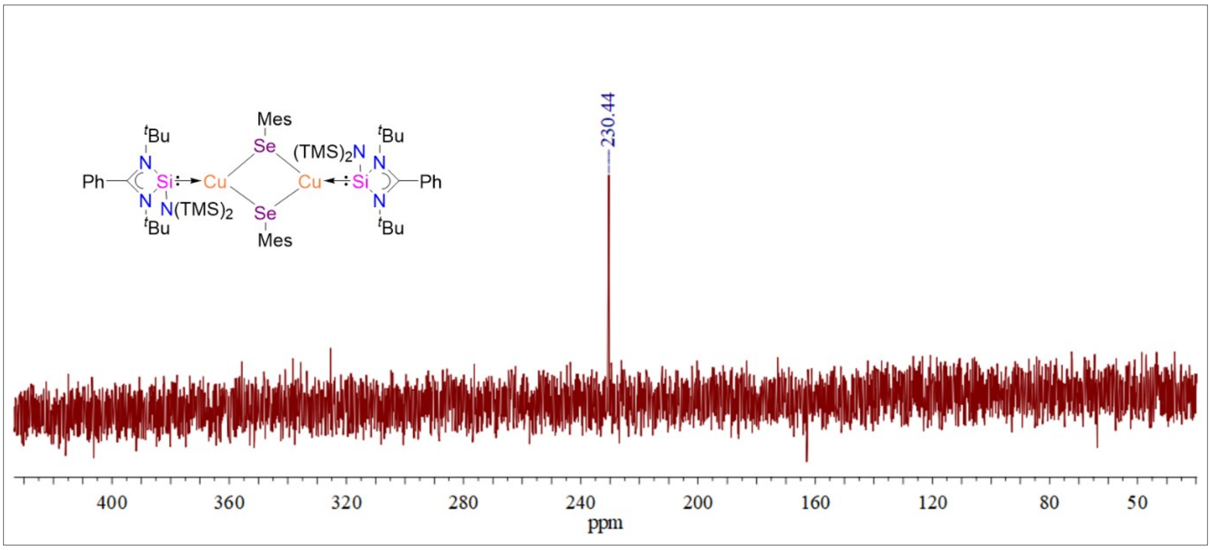

Figure S49. $^{77}Se\{^{1}H\}$ NMR spectrum of complex **15**.

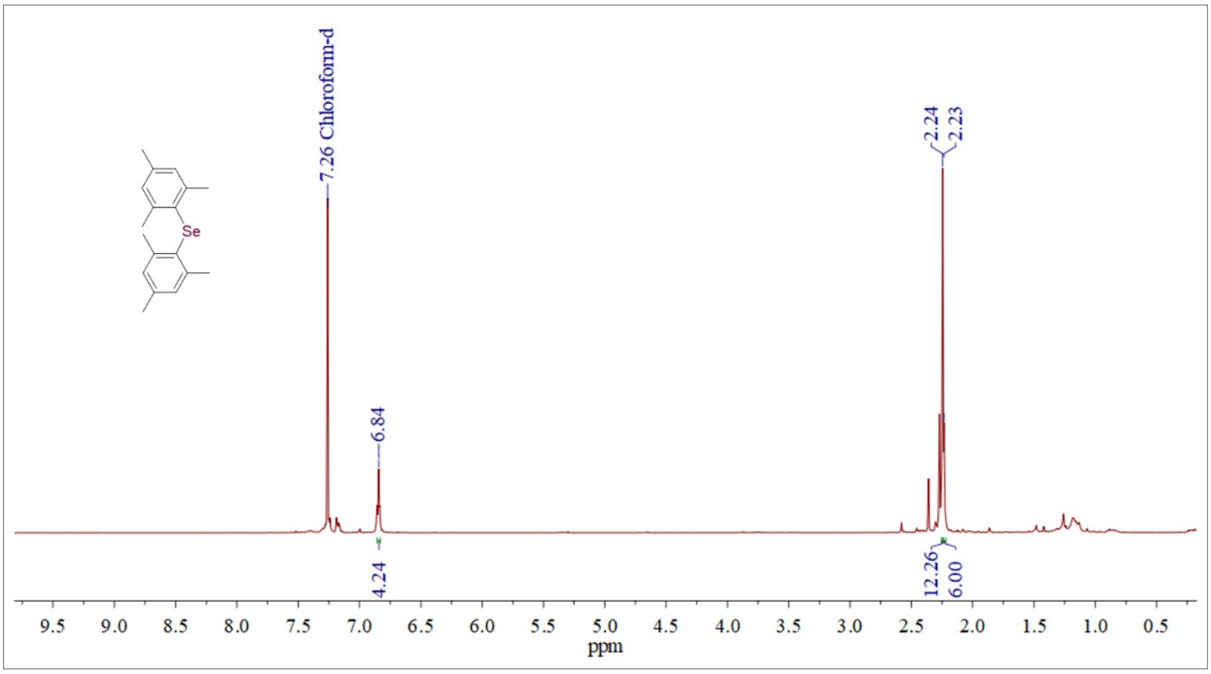

Figure S50. ^{1}H NMR spectrum of **Dimesityl selane**.

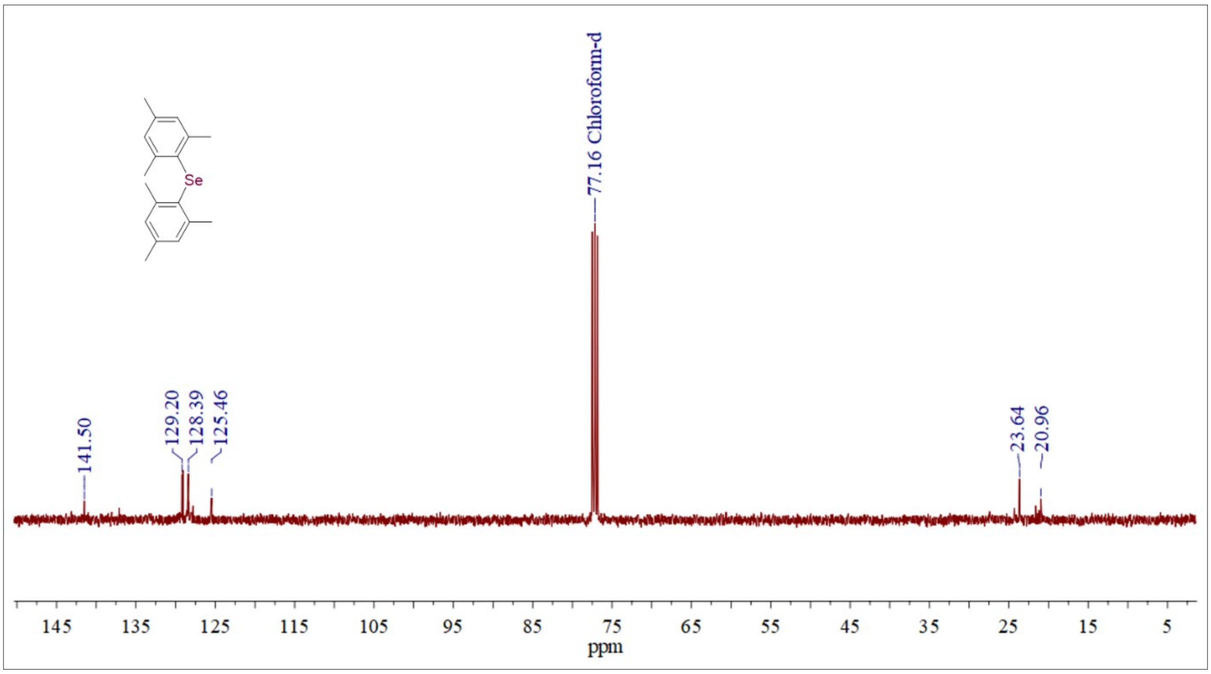

Figure S51. ^{13}C{^{1}H} NMR spectrum of **Dimesityl selane**.

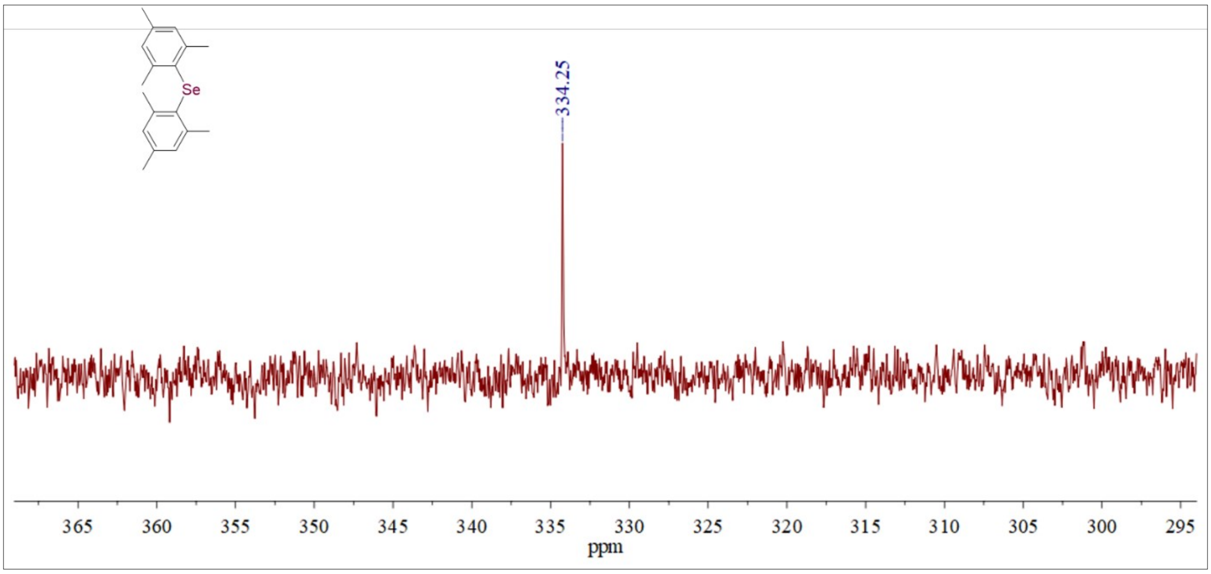

Figure S52. $^{77}Se\{^{1}H\}$ NMR spectrum of **Dimesityl selane**.

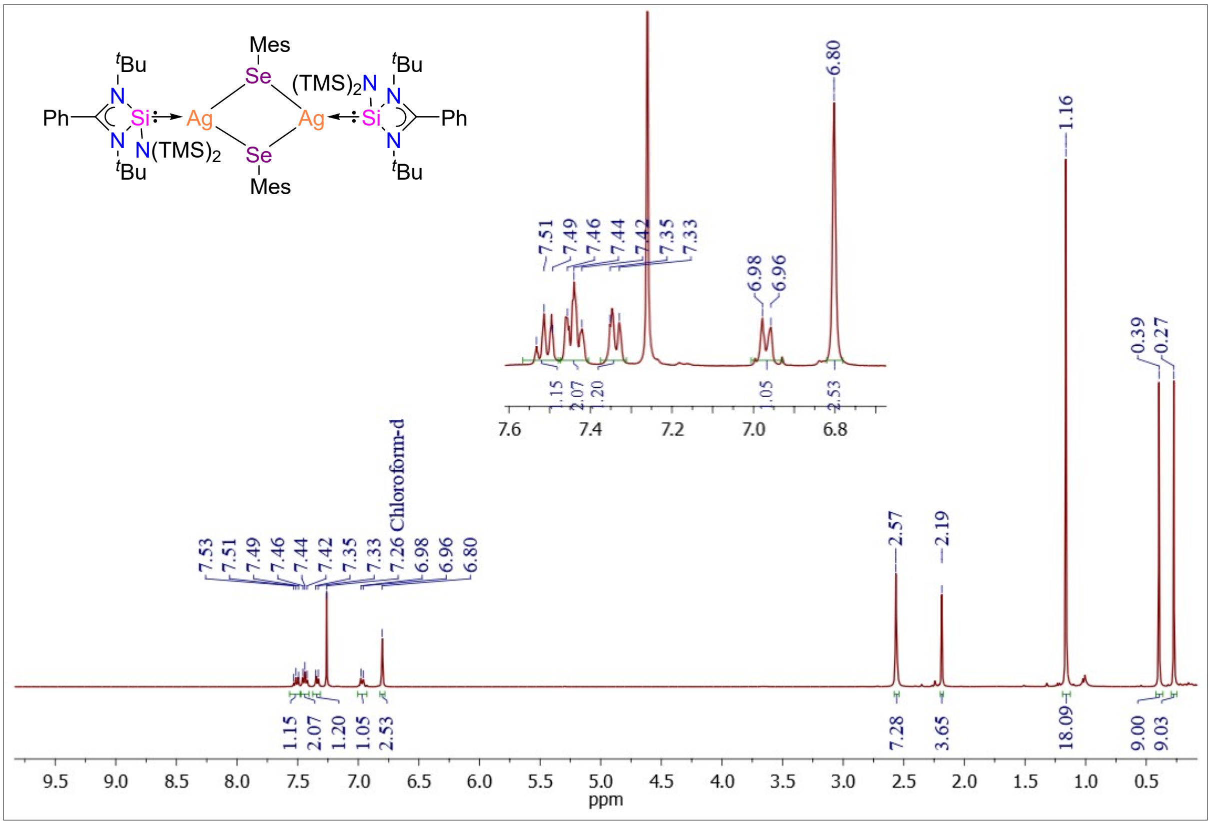

Figure S53. ^{1}H NMR spectrum of complex **16**.

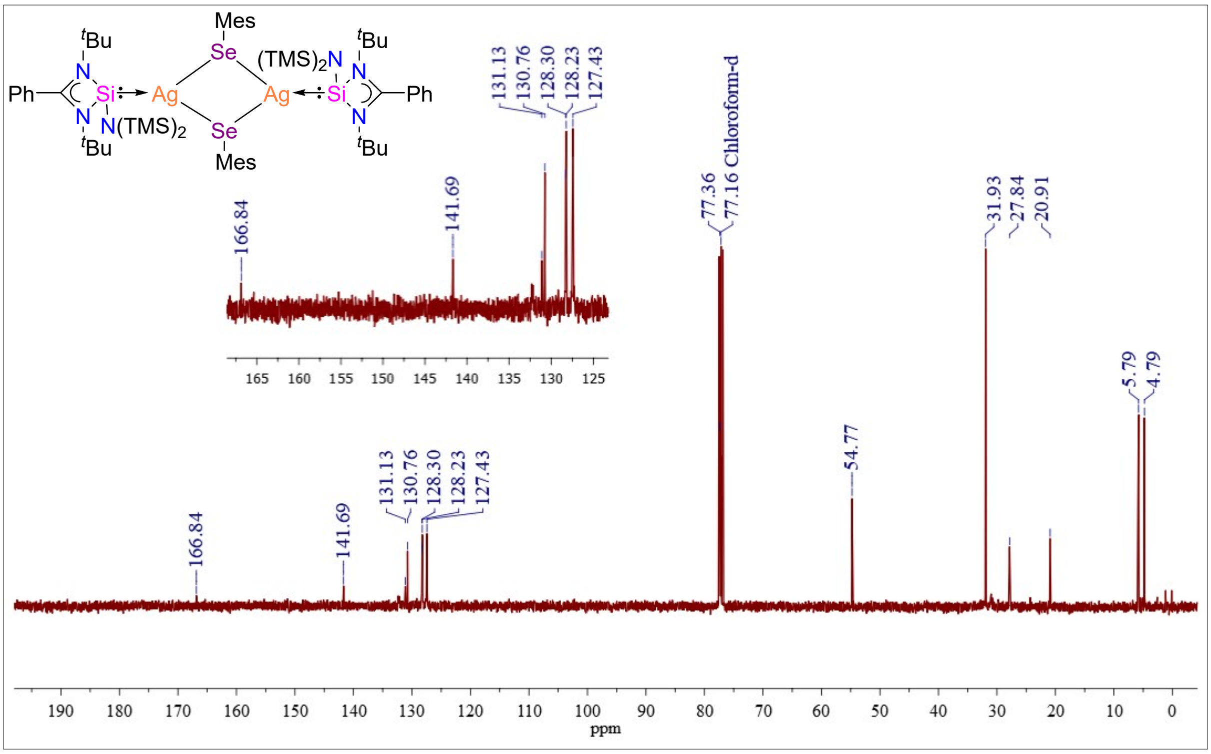

Figure S54. $^{13}C\{^{1}H\}$ NMR spectrum of complex **16**.

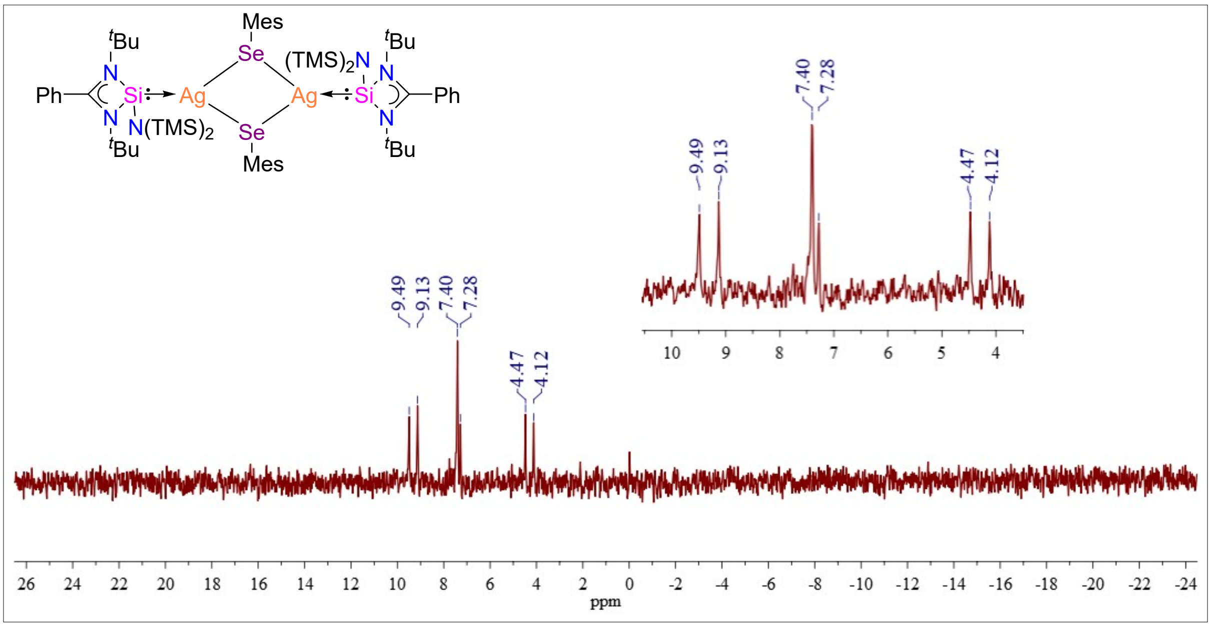

Figure S55. $^{29}Si\{^{1}H\}$ NMR spectrum of complex **16**.

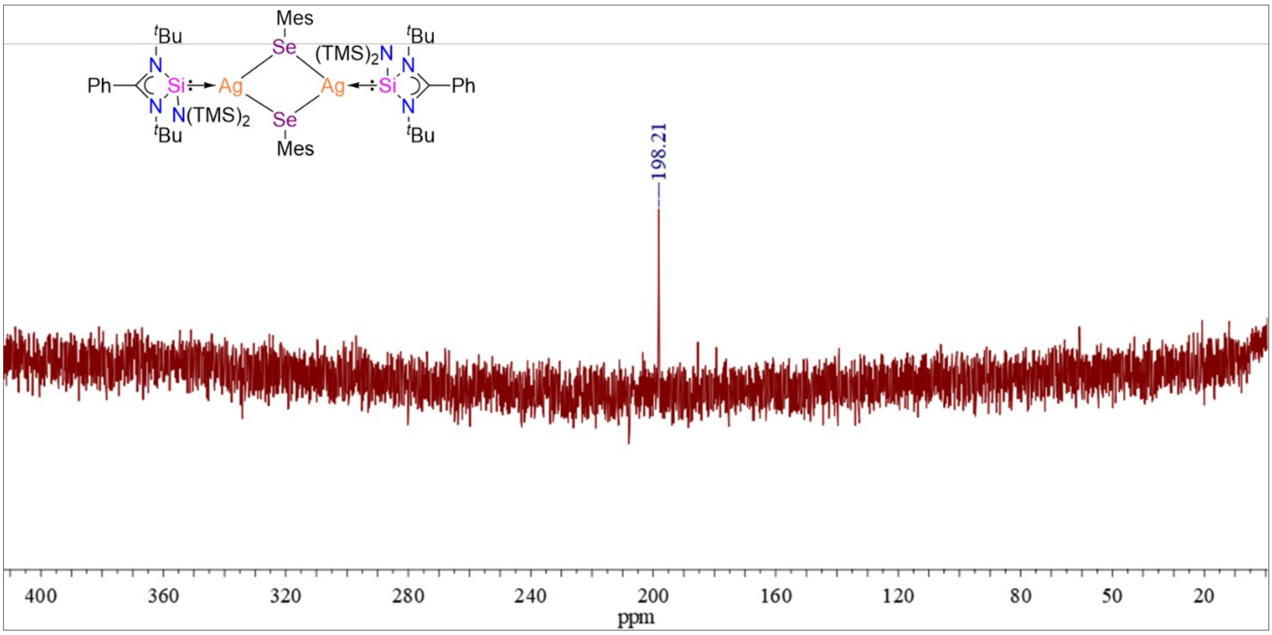

Figure S56. ^{77}Se{^{1}H} NMR spectrum of complex **16**.

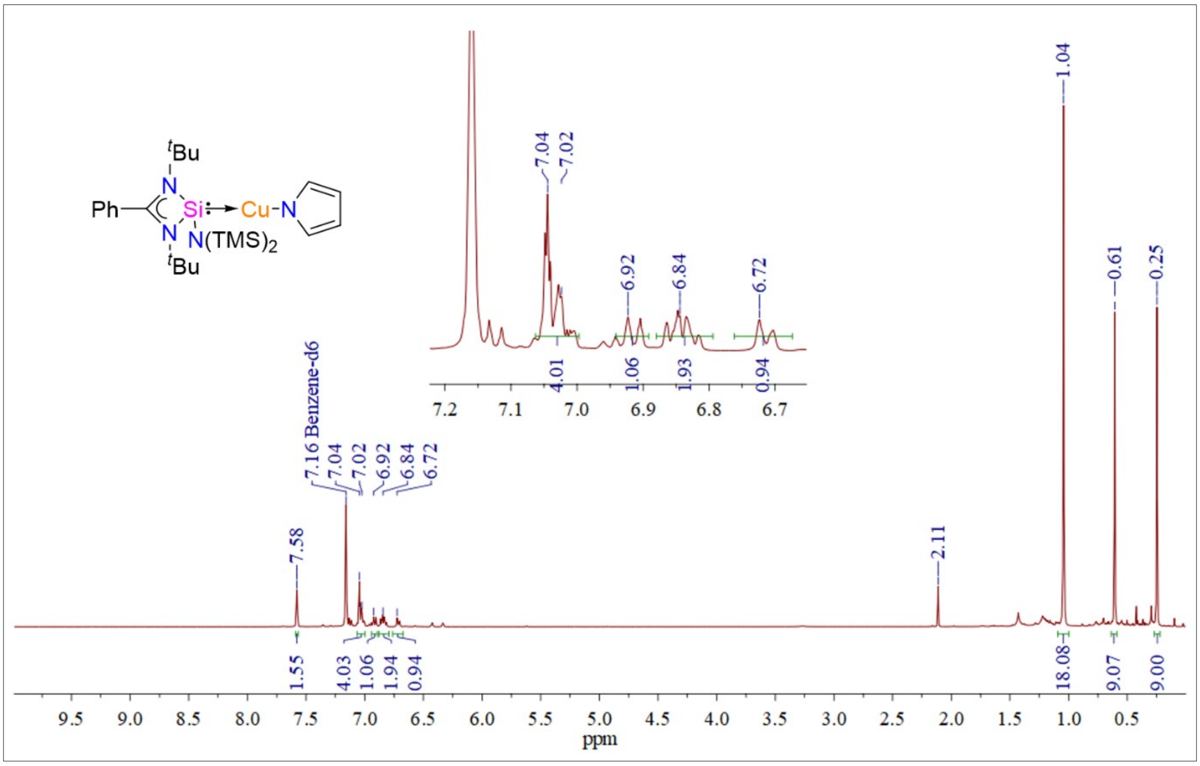

Figure S57. ^{1}H NMR spectrum of complex **17**.

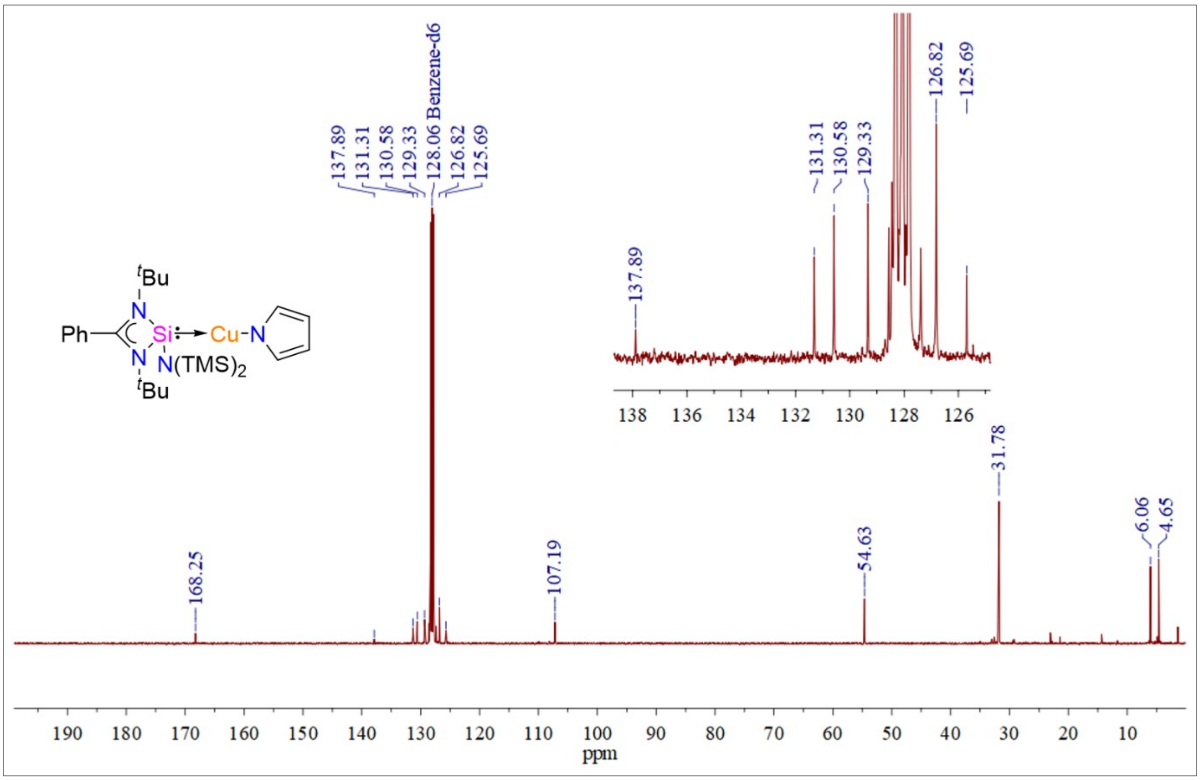

Figure S58. $^{13}C\{^{1}H\}$ NMR spectrum of complex **17**.

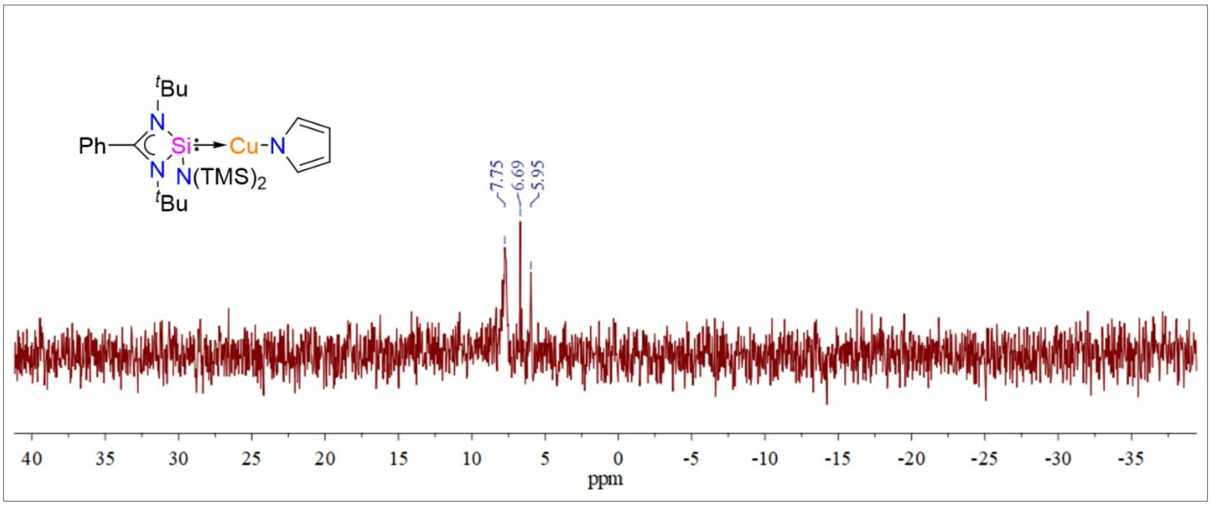

Figure S59. $^{29}Si\{^{1}H\}$ NMR spectrum of complex **17**.

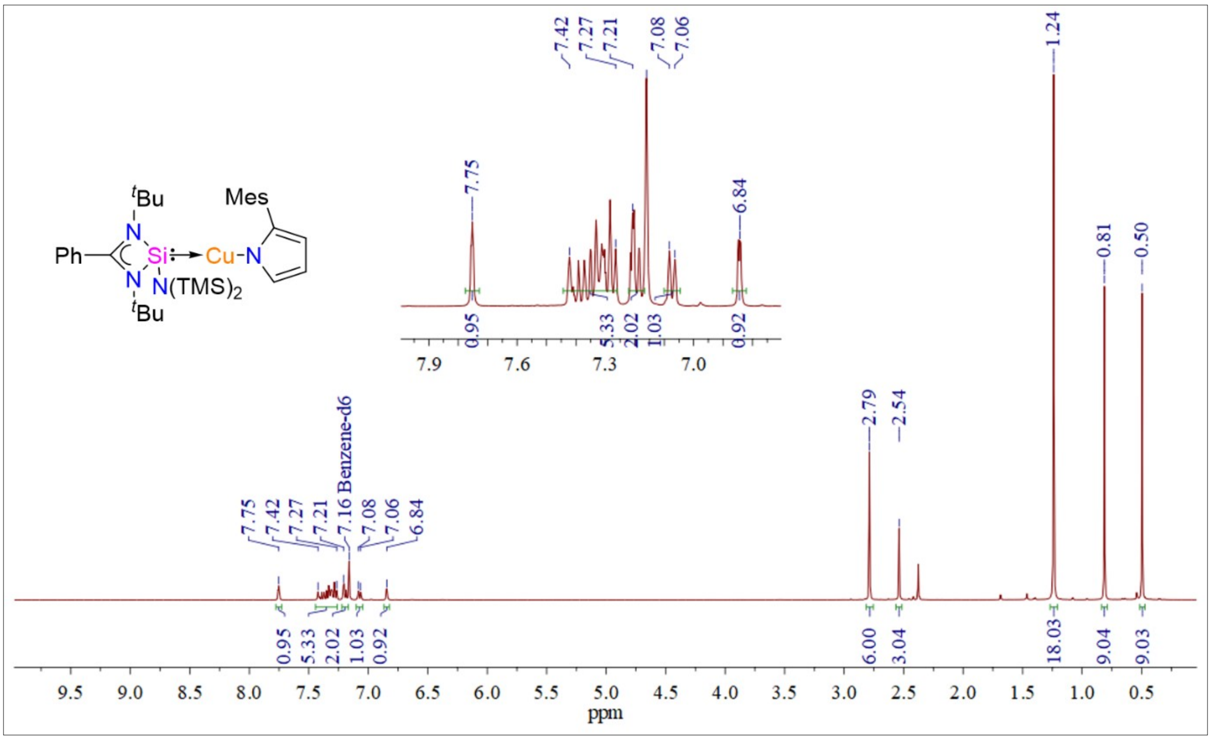

Figure S60. ^{1}H NMR spectrum of complex **18**.

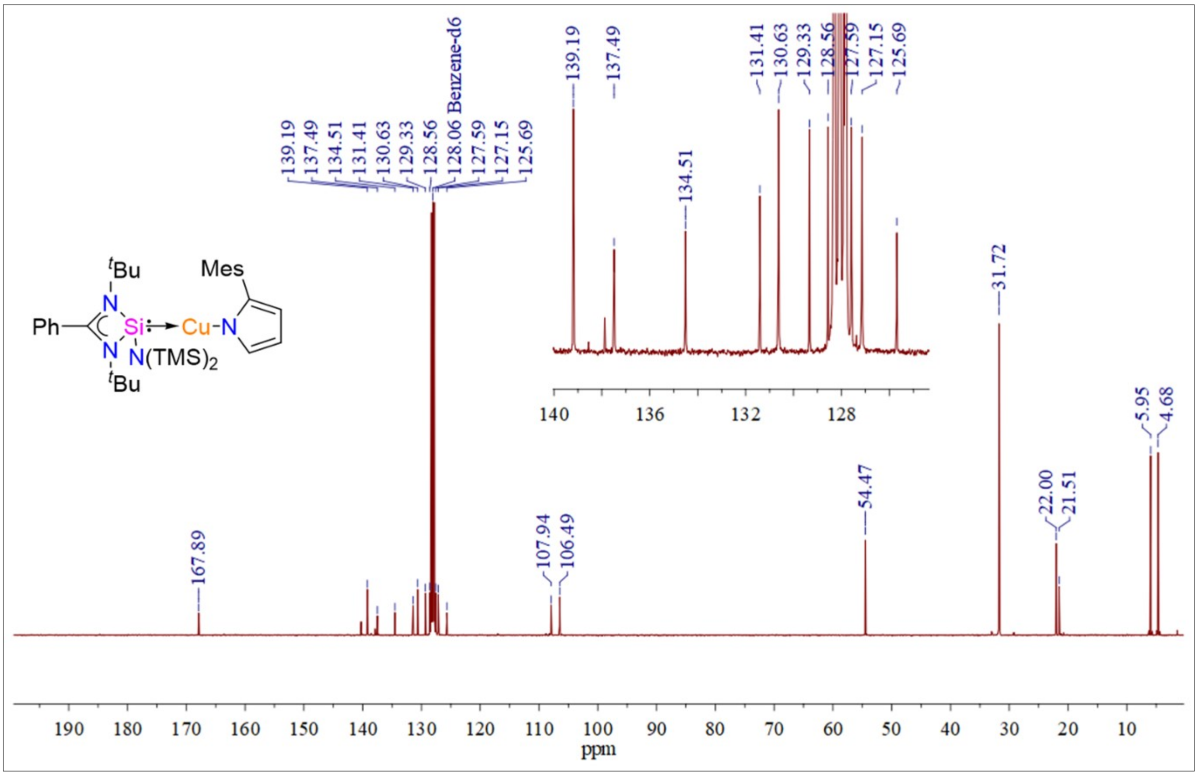

Figure S61. $^{13}C\{^{1}H\}$ NMR spectrum of complex **18**.

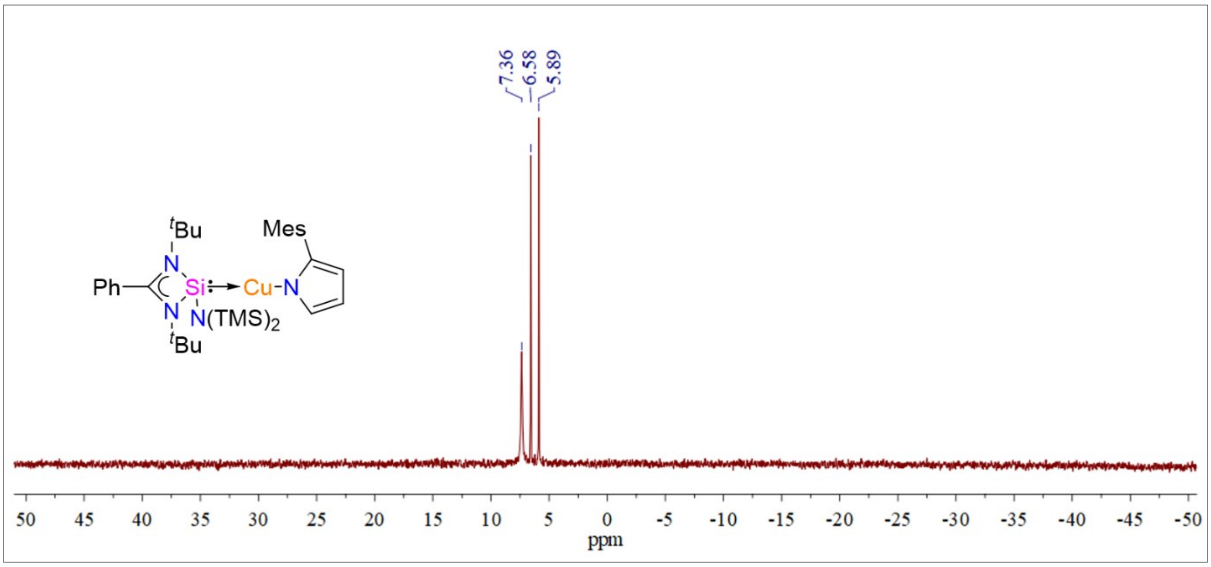

Figure S62. $^{29}Si\{^{1}H\}$ NMR spectrum of complex **18**.

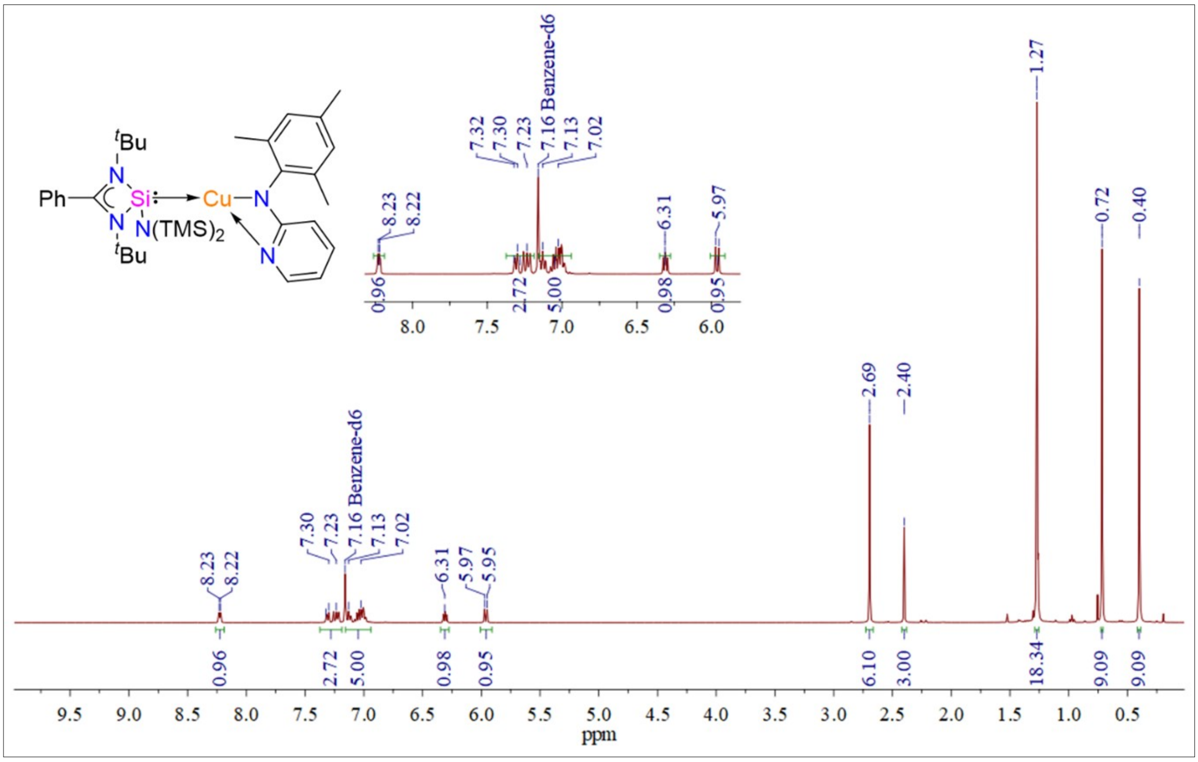

Figure S63. ^{1}H NMR spectrum of complex **19**.

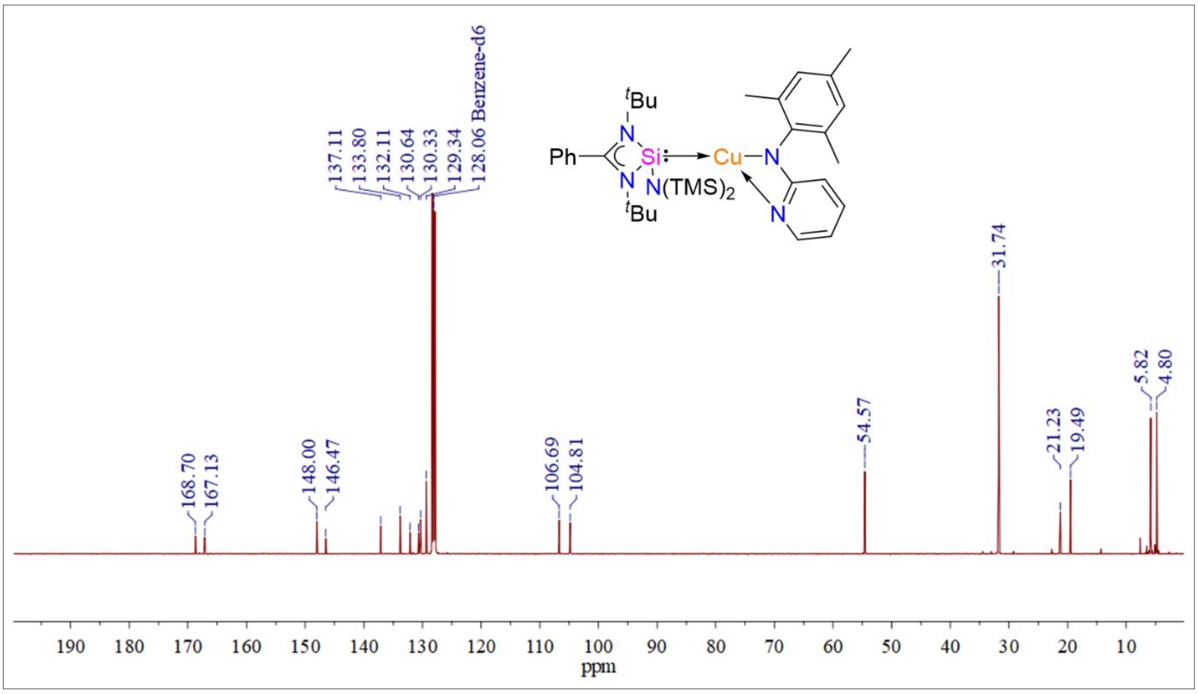

Figure S64. $^{13}C\{^1H\}$ NMR spectrum of complex **19**.

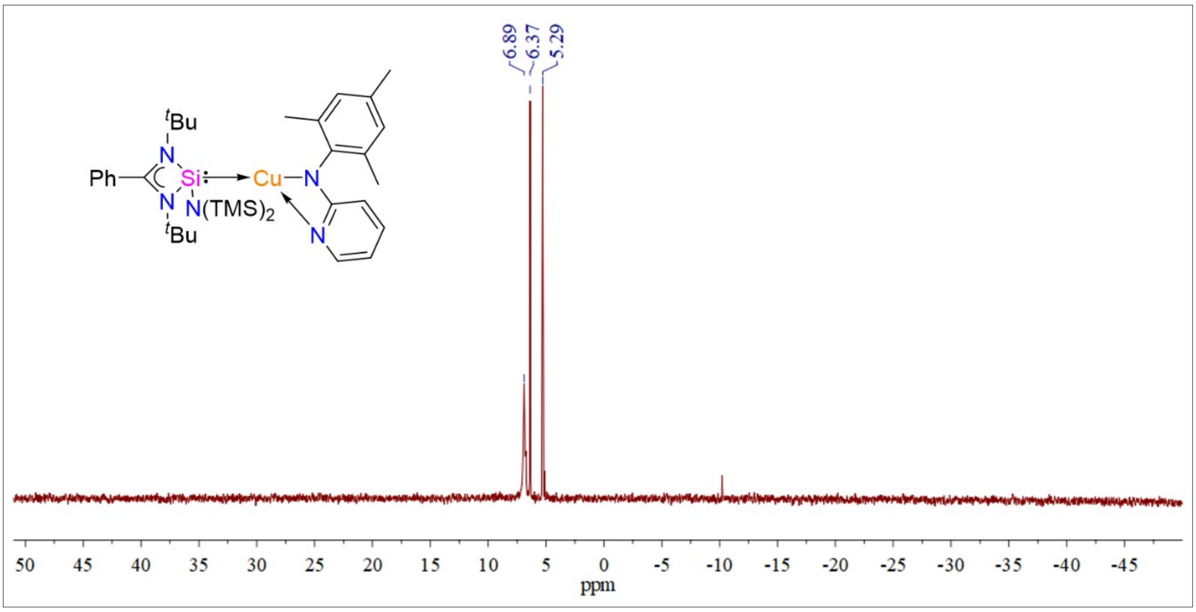

Figure S65. $^{29}Si\{^1H\}$ NMR spectrum of complex **19**.

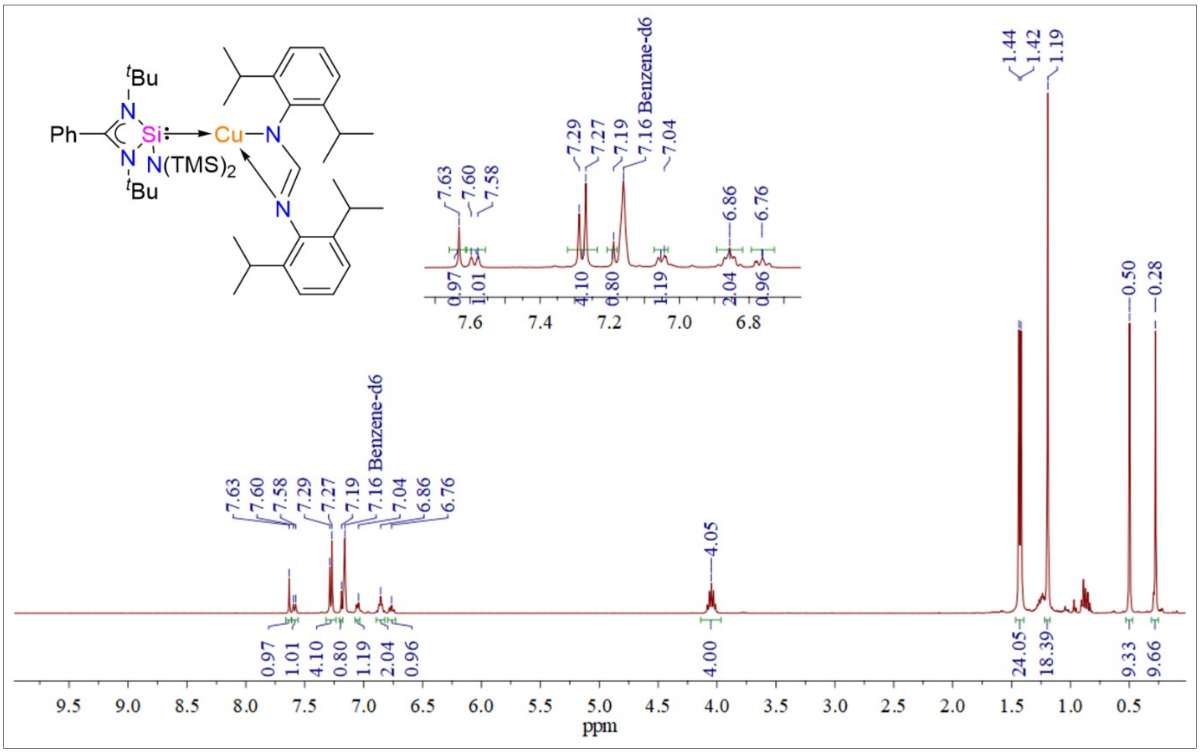

Figure S66. ^{1}H NMR spectrum of complex **20**.

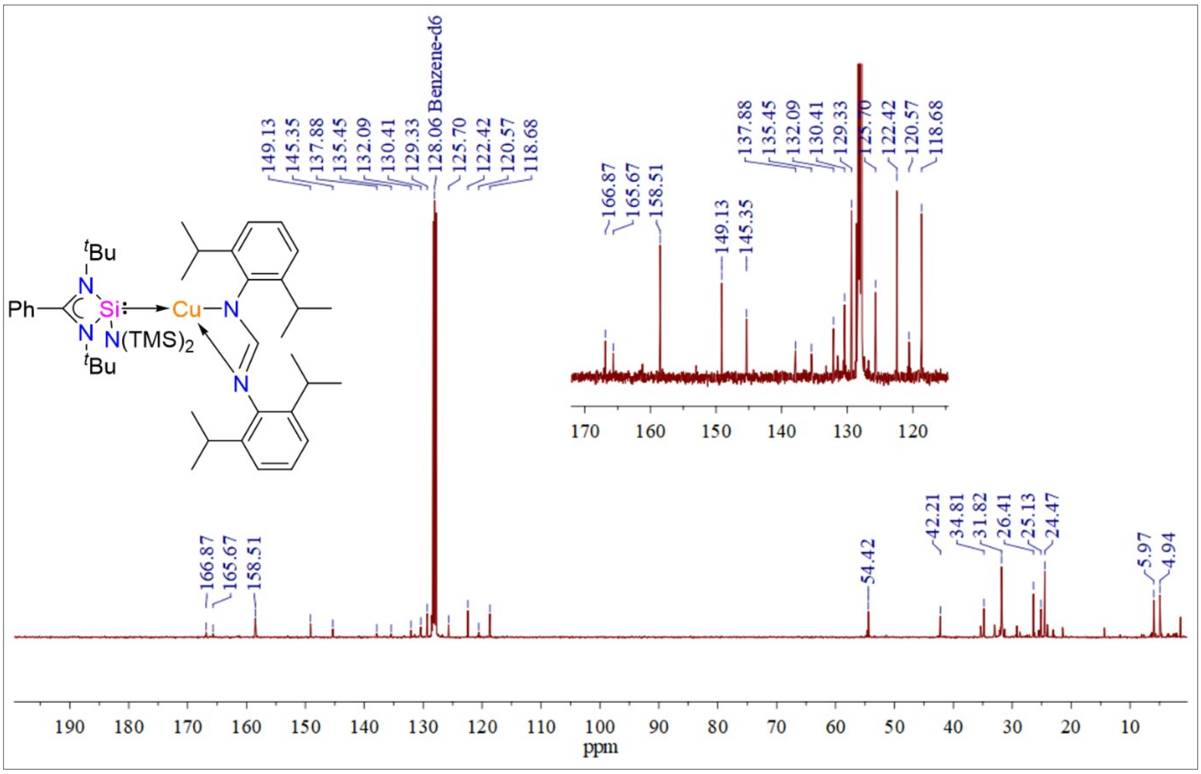

Figure S67. $^{13}C\{^{1}H\}$ NMR spectrum of complex **20**.

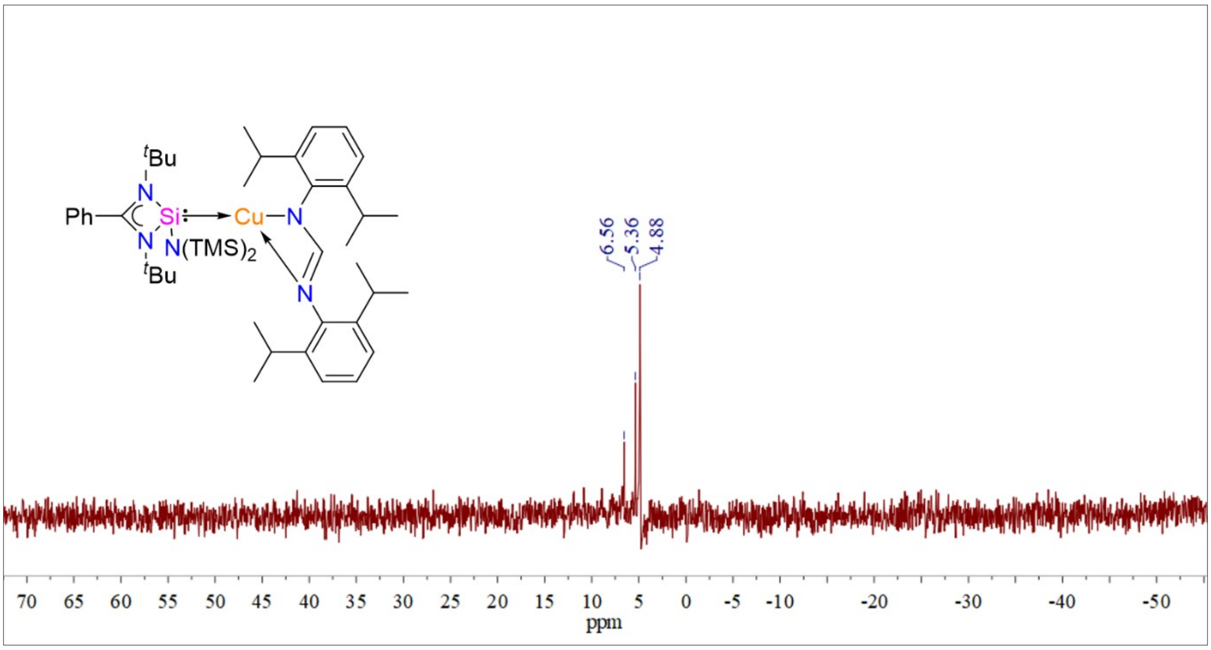

Figure S68. $^{29}Si\{^1H\}$ NMR spectrum of complex **20**.

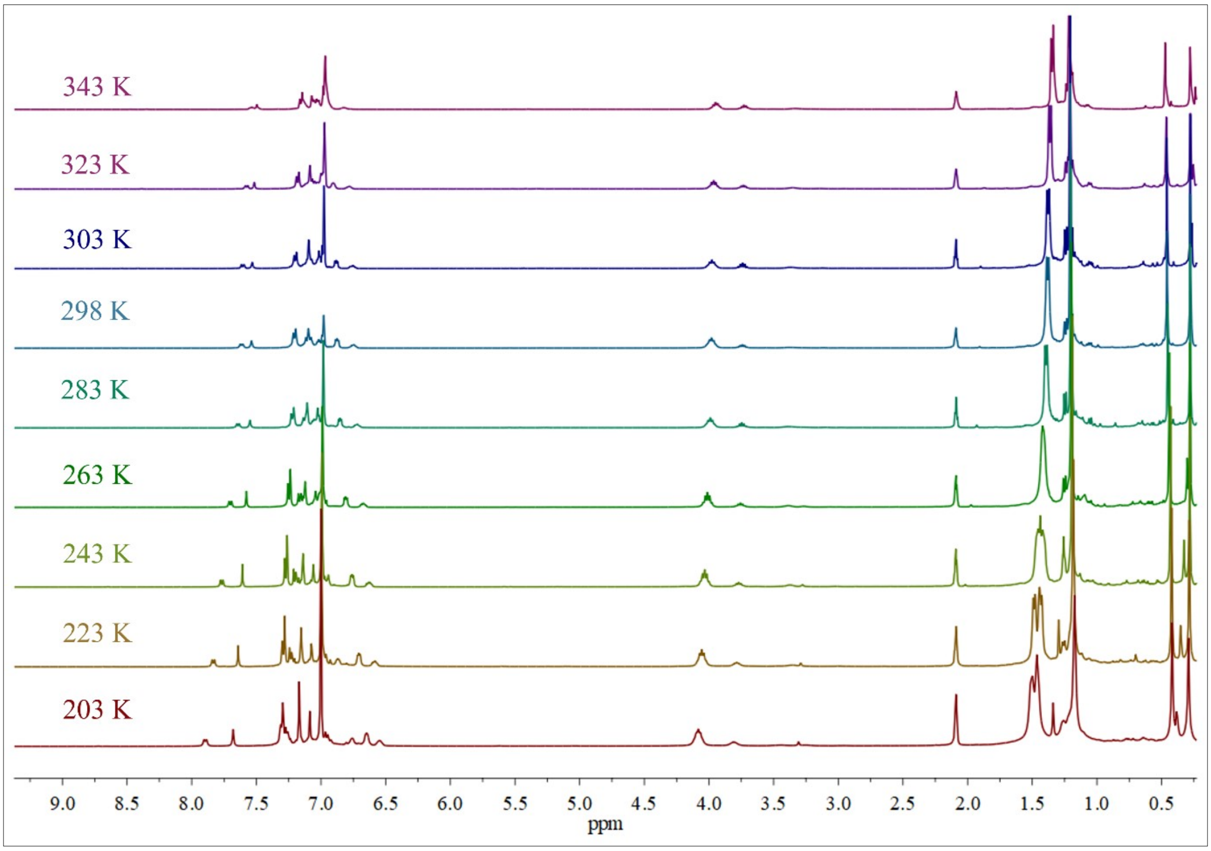

Figure S69. Variable temperature ^1H NMR spectra of complex **20** in toluene-d_8.

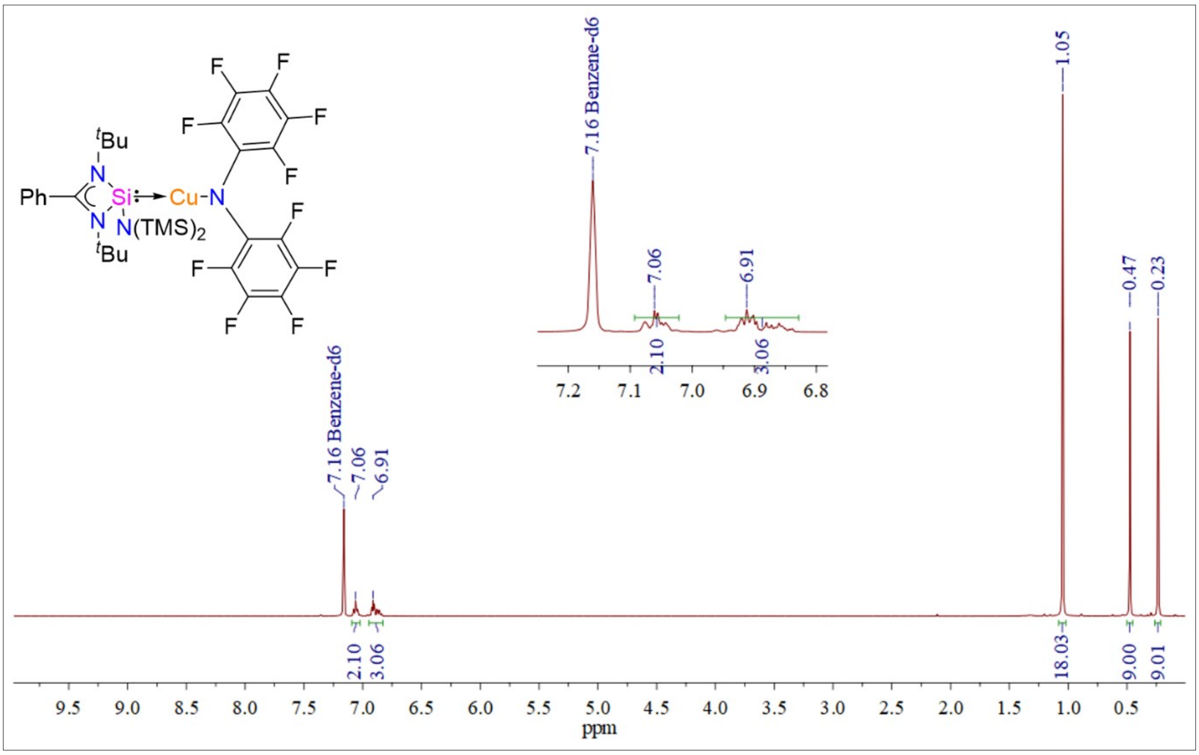

Figure S70. ^{1}H NMR spectrum of complex **21**.

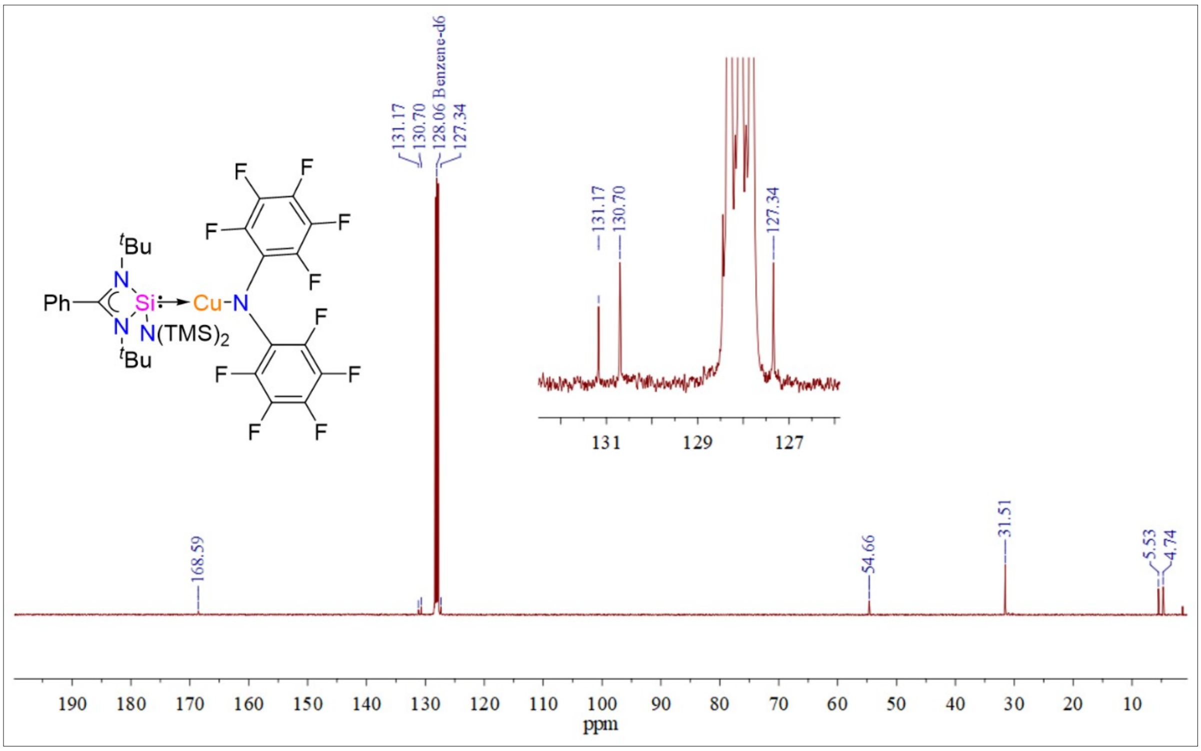

Figure S71. $^{13}C\{^{1}H\}$ NMR spectrum of complex **21**.

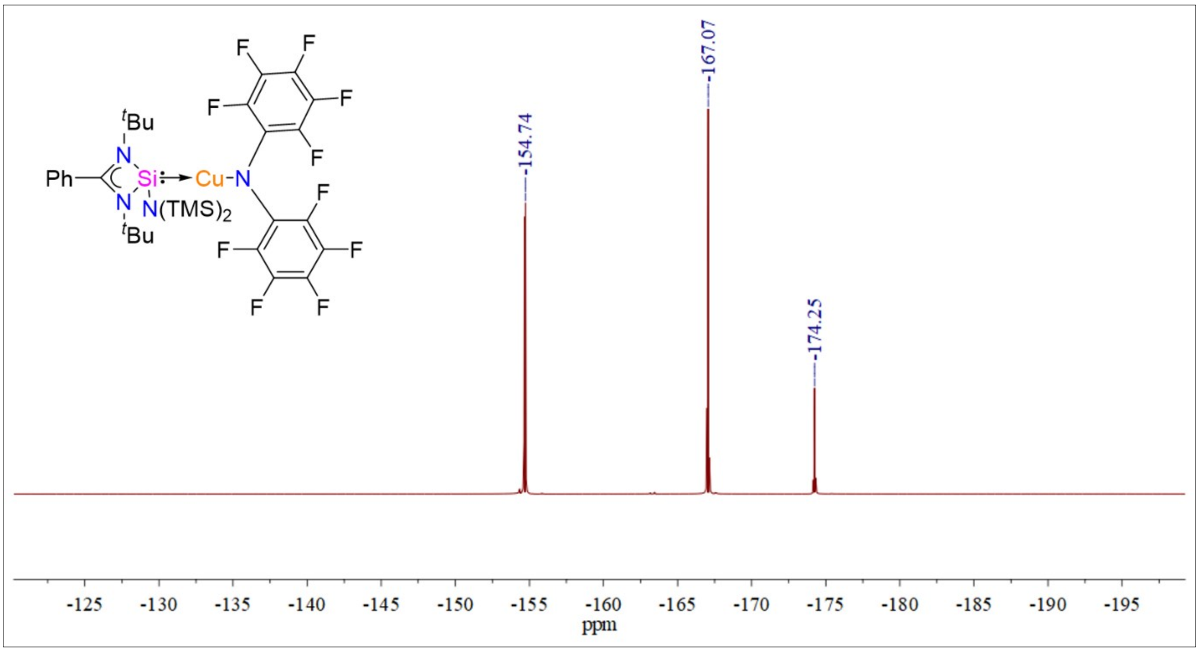

Figure S72. ^{19}F NMR spectrum of complex **21**.

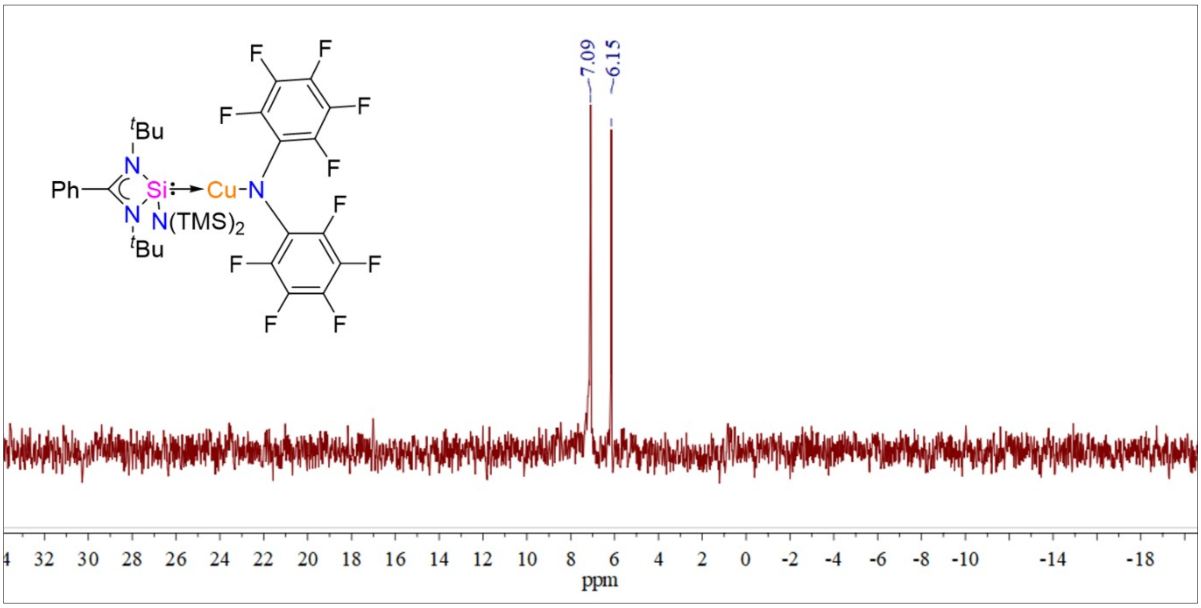

Figure S73. $^{29}Si\{^1H\}$ NMR spectrum of complex **21**.

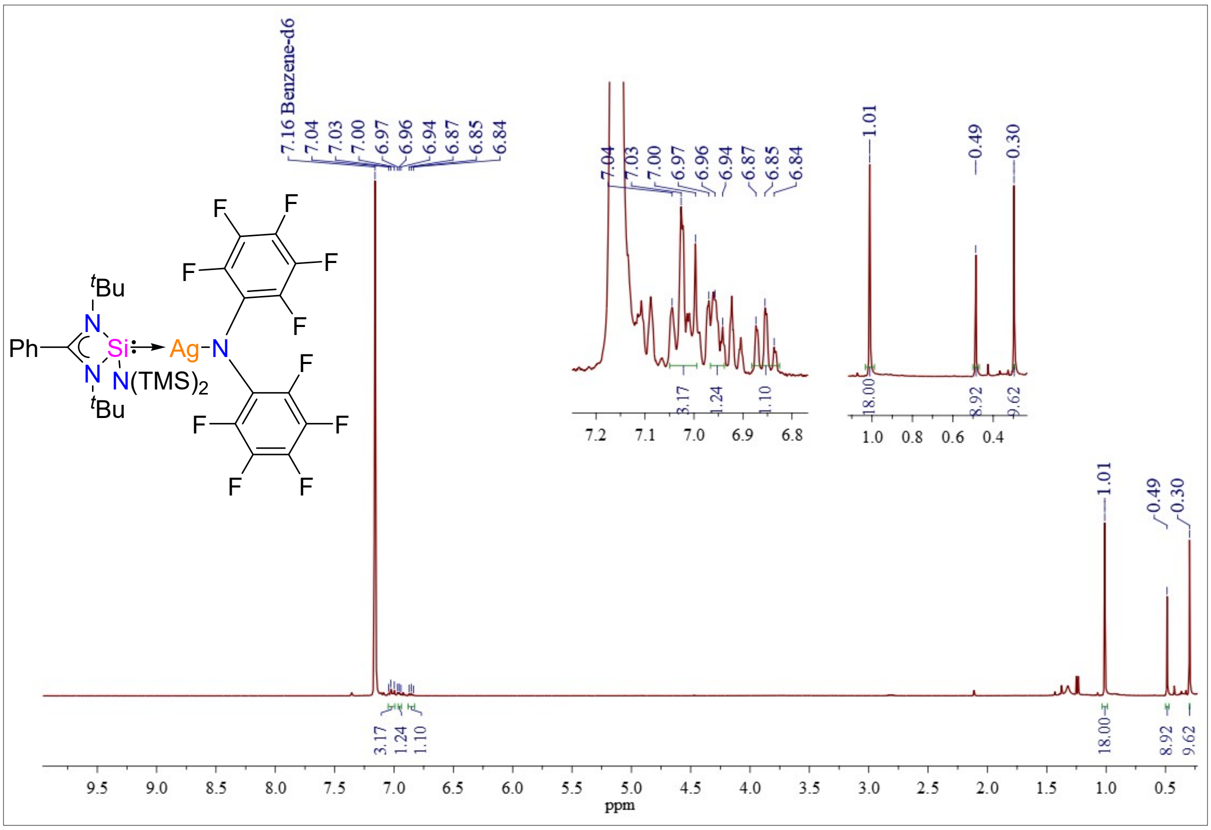

Figure S74. ^{1}H NMR spectrum of complex **22**.

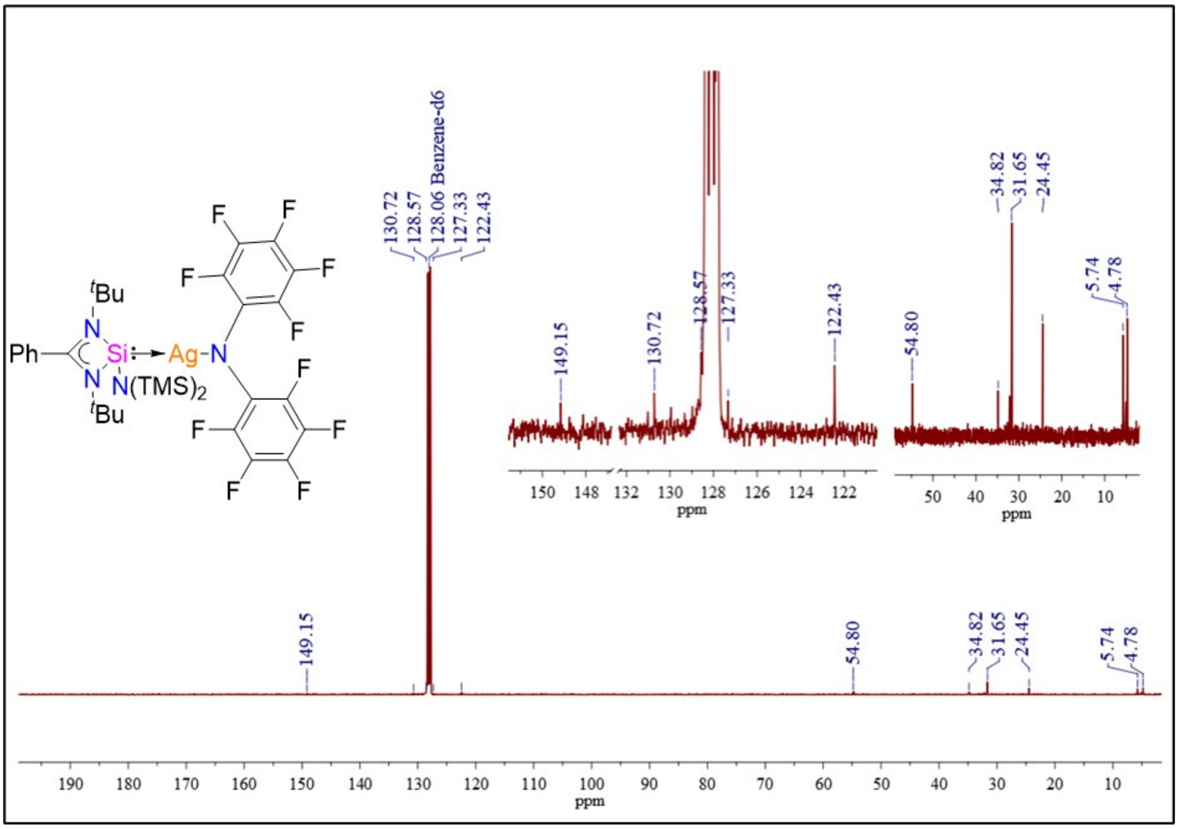

Figure S75. $^{13}C\{^{1}H\}$ NMR spectrum of complex **22**.

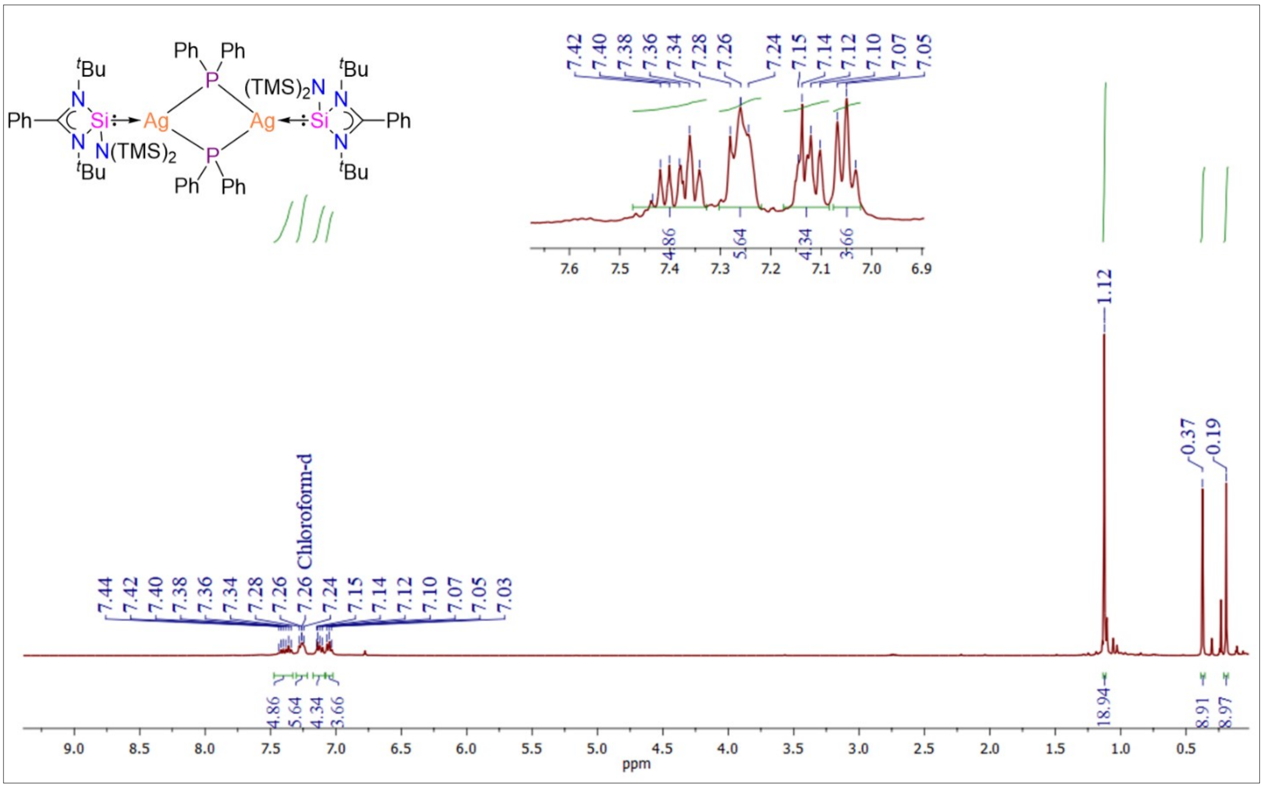

Figure S76. ^{1}H NMR spectrum of complex **24**.

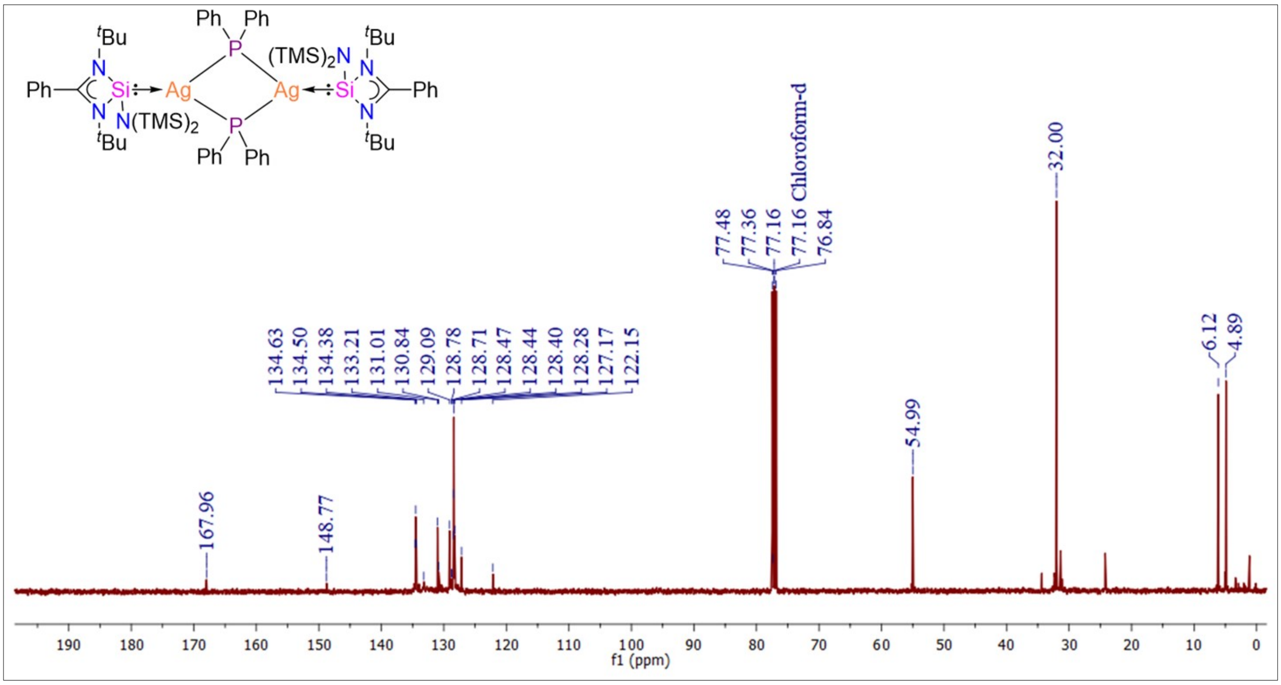

Figure S77. $^{13}C\{^{1}H\}$ NMR spectrum of complex **24**.

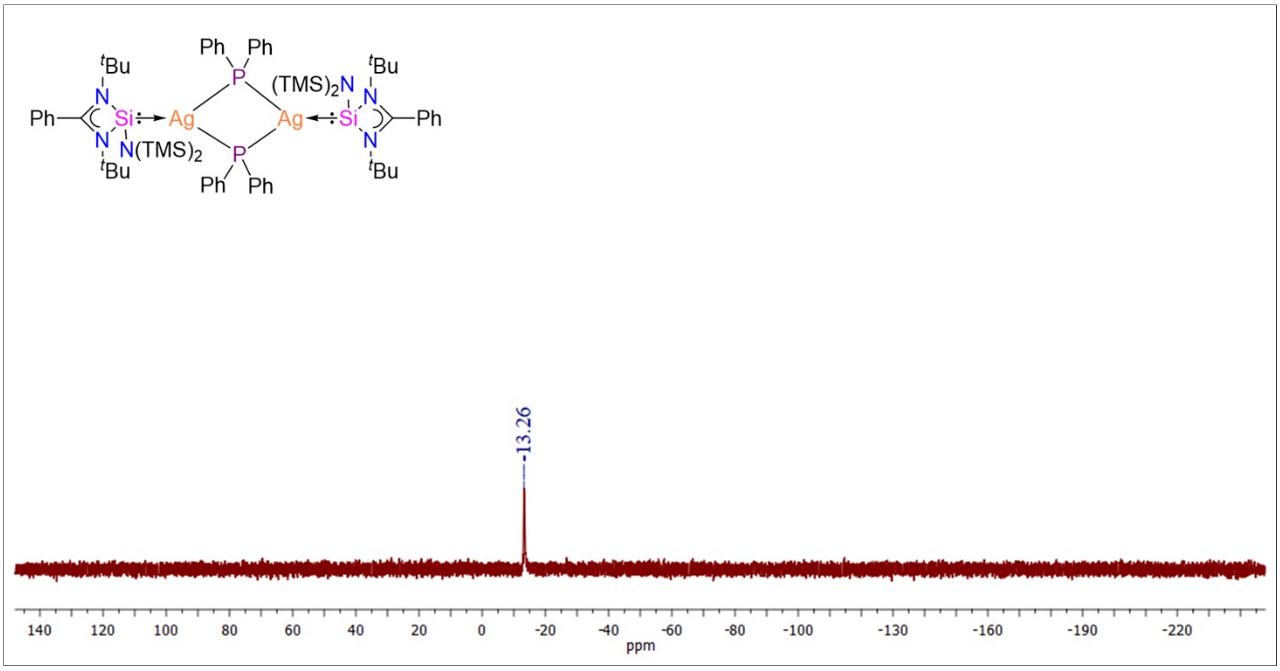

Figure S78. $^{31}P\{^{1}H\}$ NMR spectrum of complex **24**.

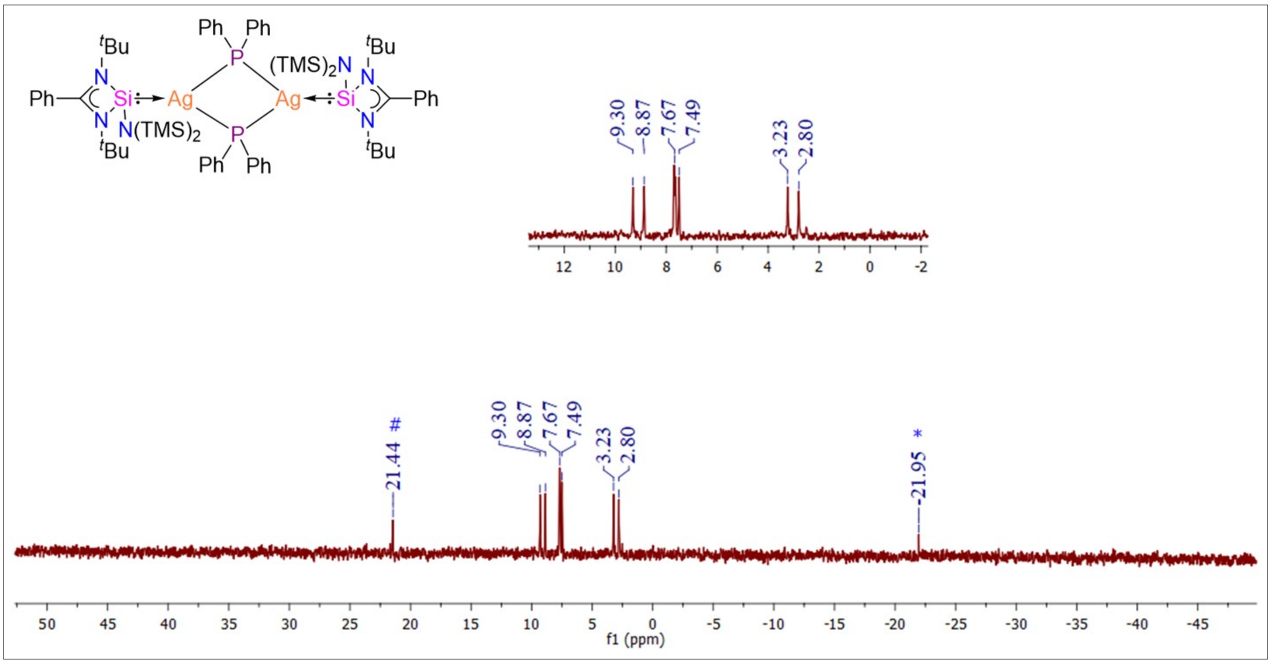

Figure S79. $^{29}Si\{^{1}H\}$ NMR spectrum of complex **24** (* = silicon grease; # = unknown).

S2.2. NMR Spectra of C–C Coupled Products

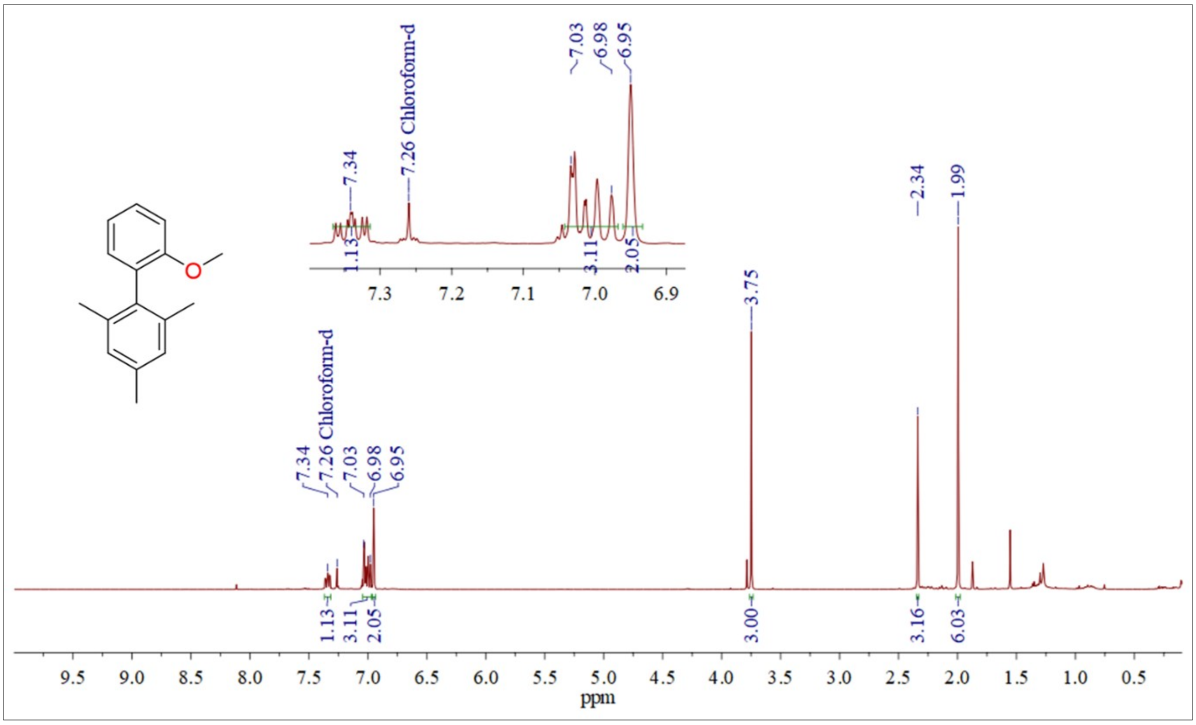

Figure S80. ^{1}H NMR spectrum of complex **I**.

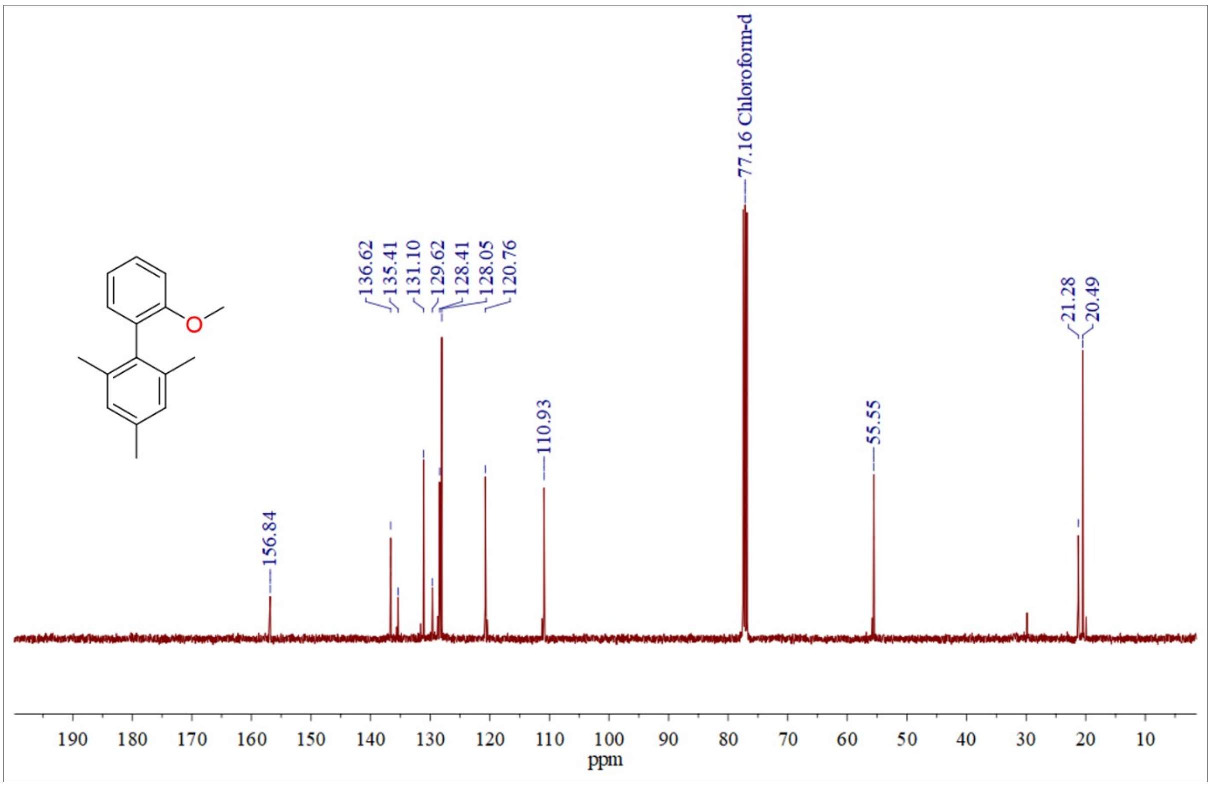

Figure S81. $^{13}C\{^{1}H\}$ NMR spectrum of complex **I**.

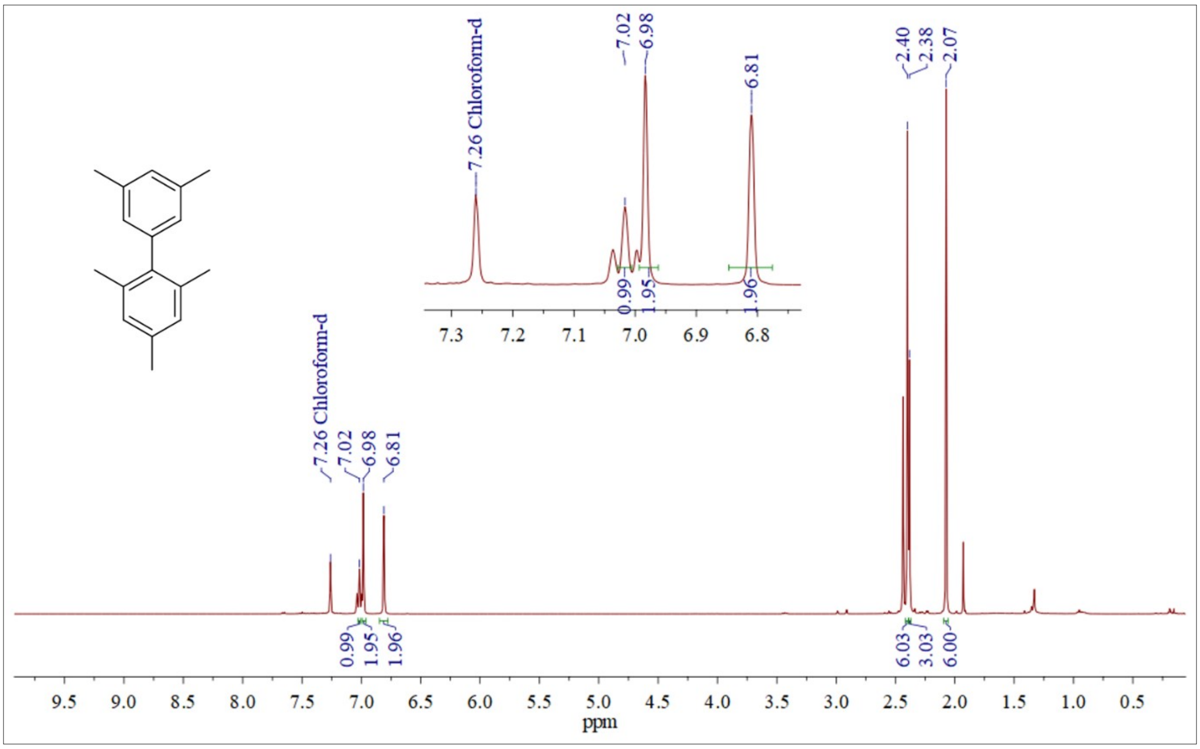

Figure S82. ^{1}H NMR spectrum of complex **II**.

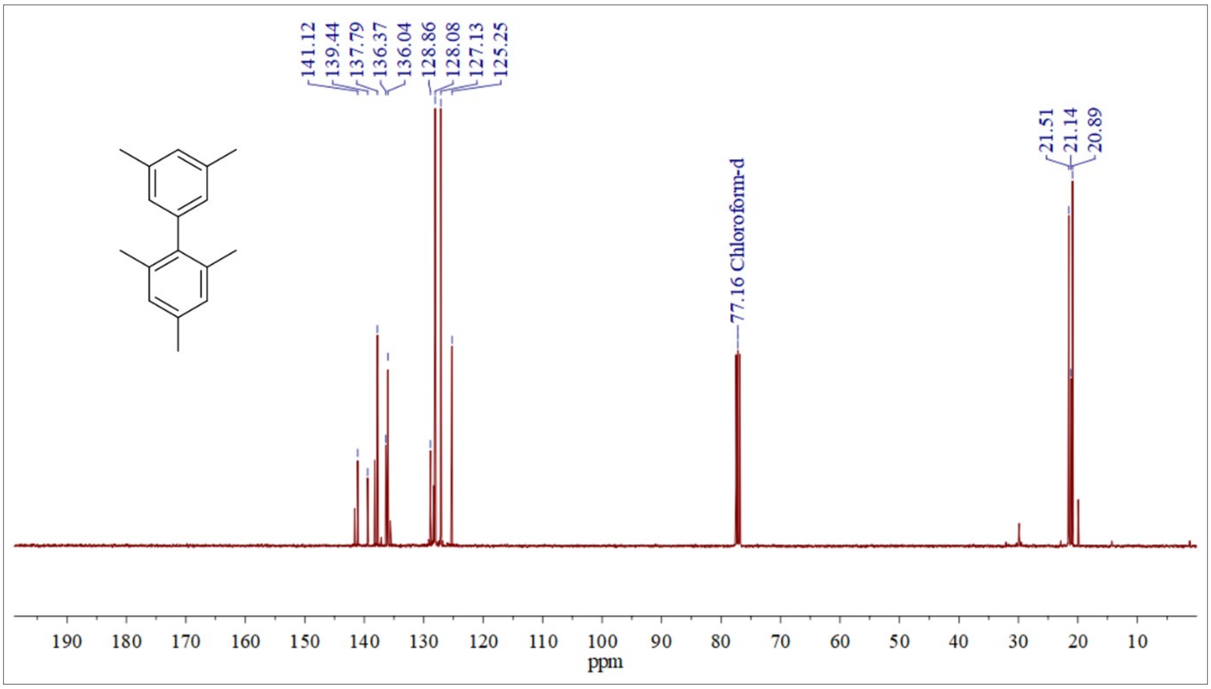

Figure S83. $^{13}C\{^{1}H\}$ NMR spectrum of complex **II**.

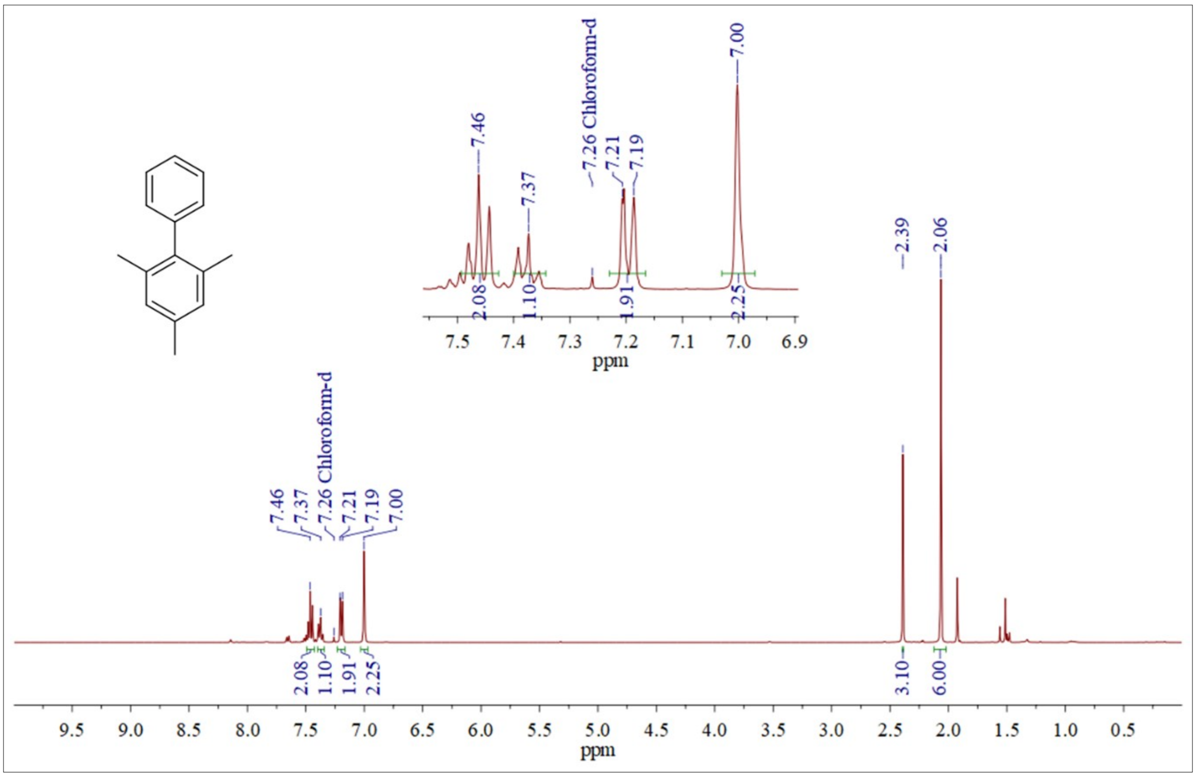

Figure S84. ^{1}H NMR spectrum of complex **III**.

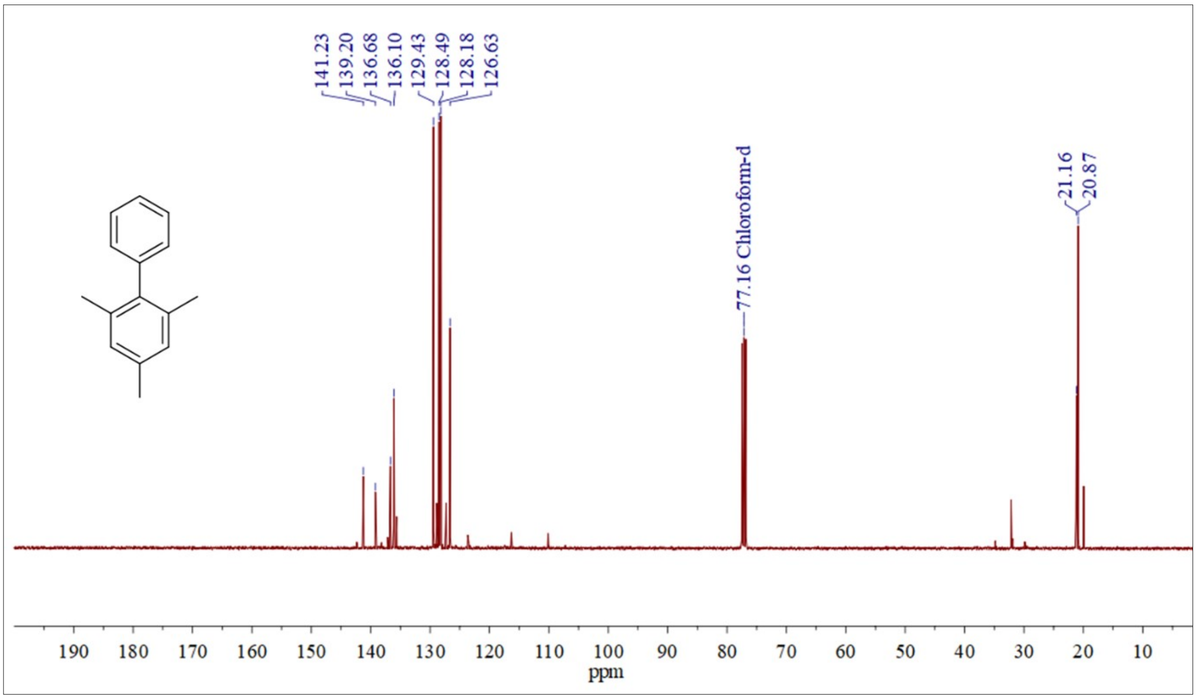

Figure S85. $^{13}C\{^{1}H\}$ NMR spectrum of complex **III**.

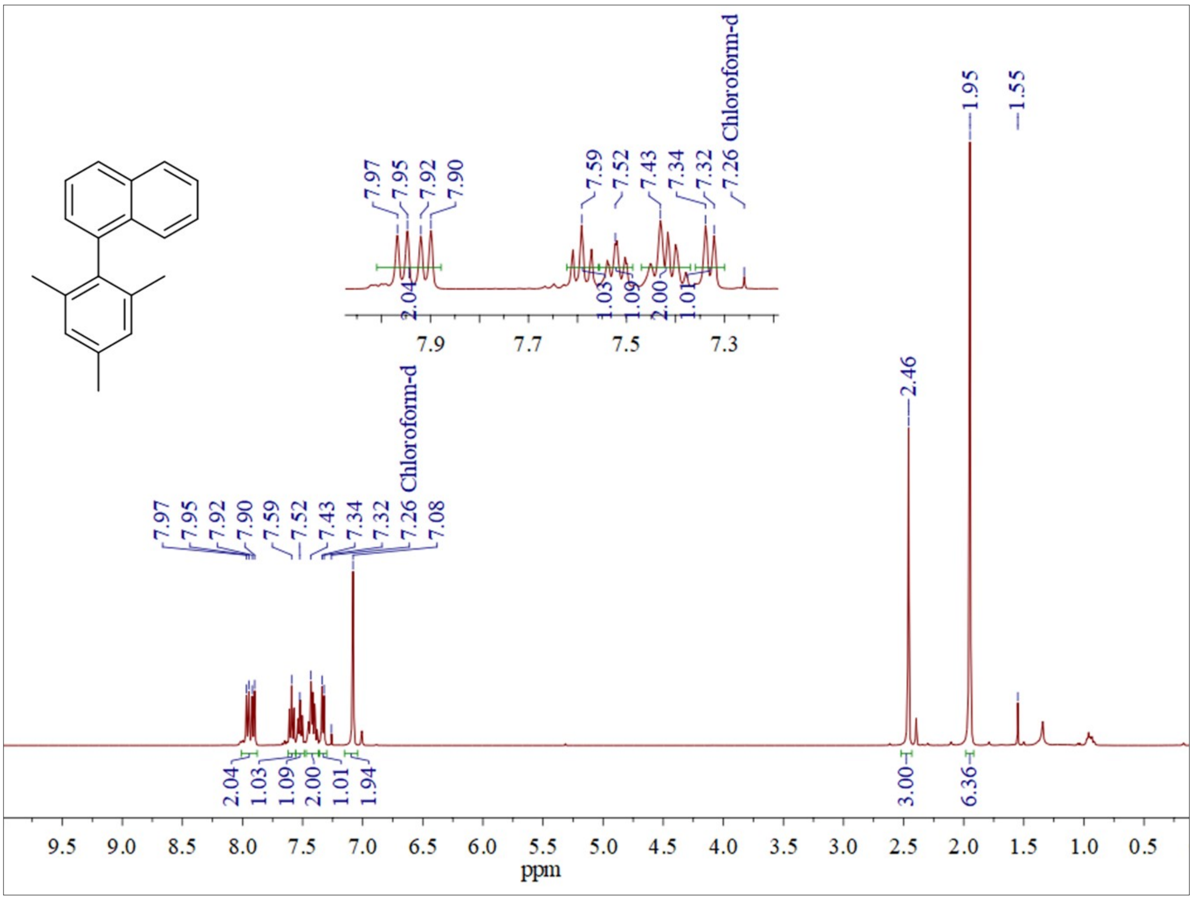

Figure S86. ^{1}H NMR spectrum of complex **IV**.

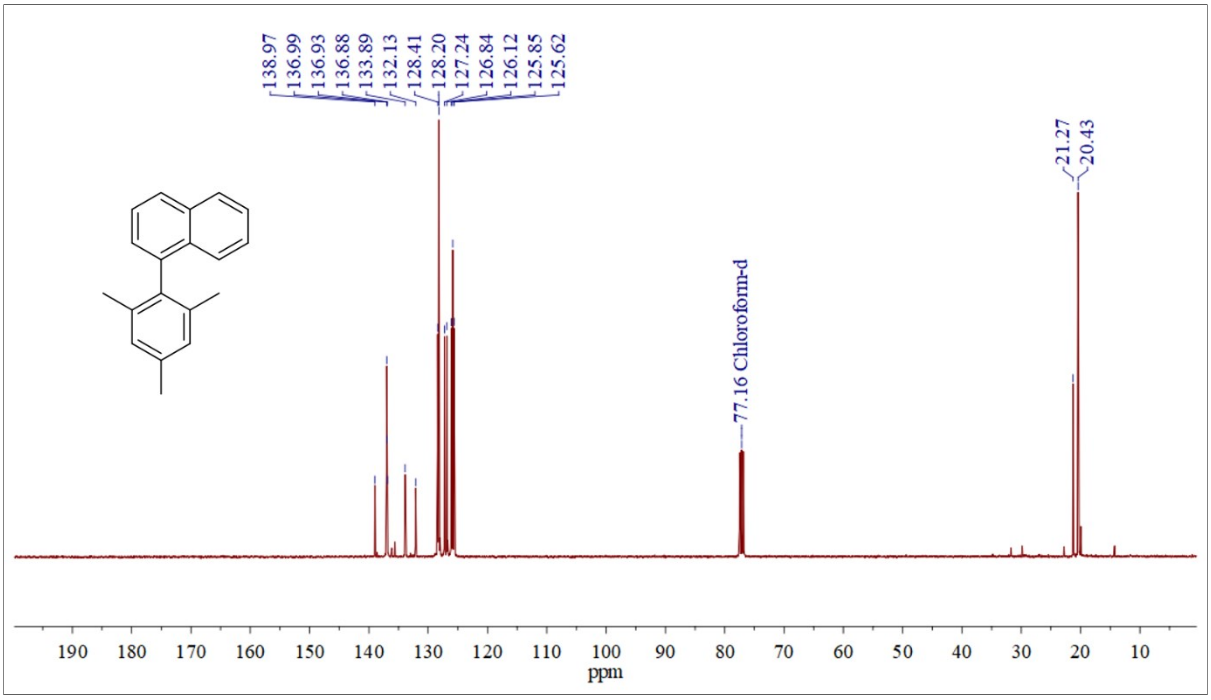

Figure S87. $^{13}C\{^1H\}$ NMR spectrum of complex **IV**.

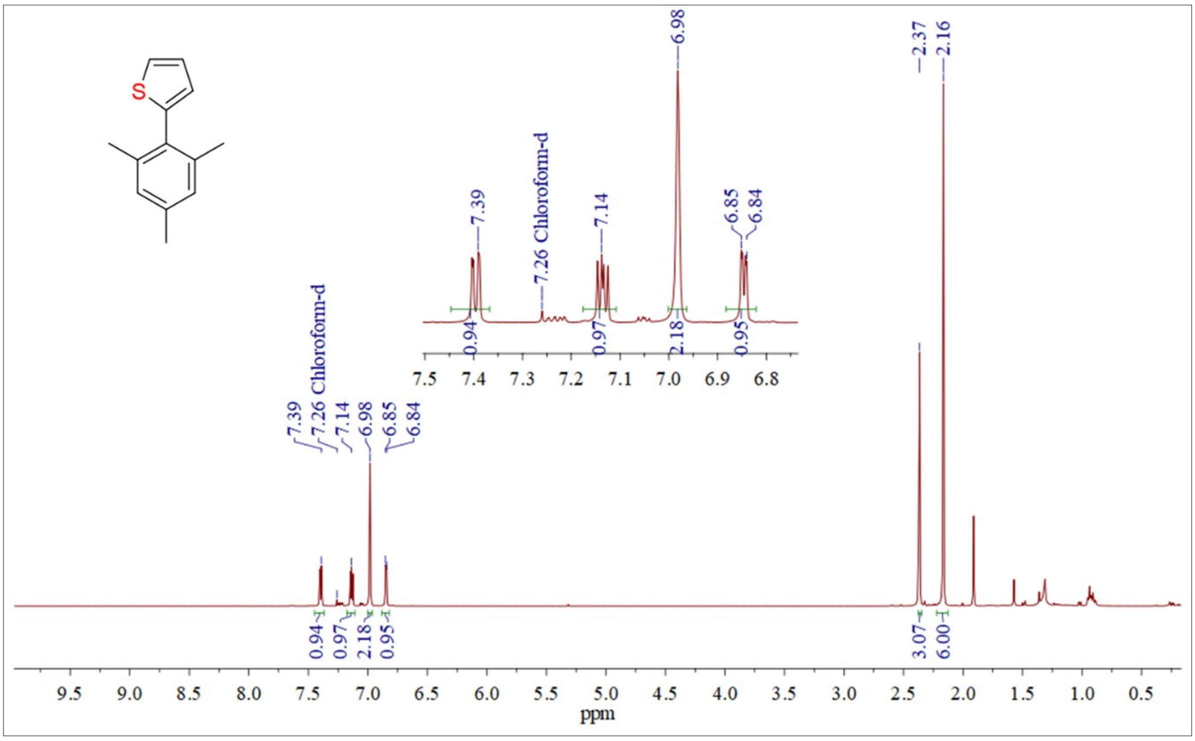

Figure S88. ^{1}H NMR spectrum of complex **V**.

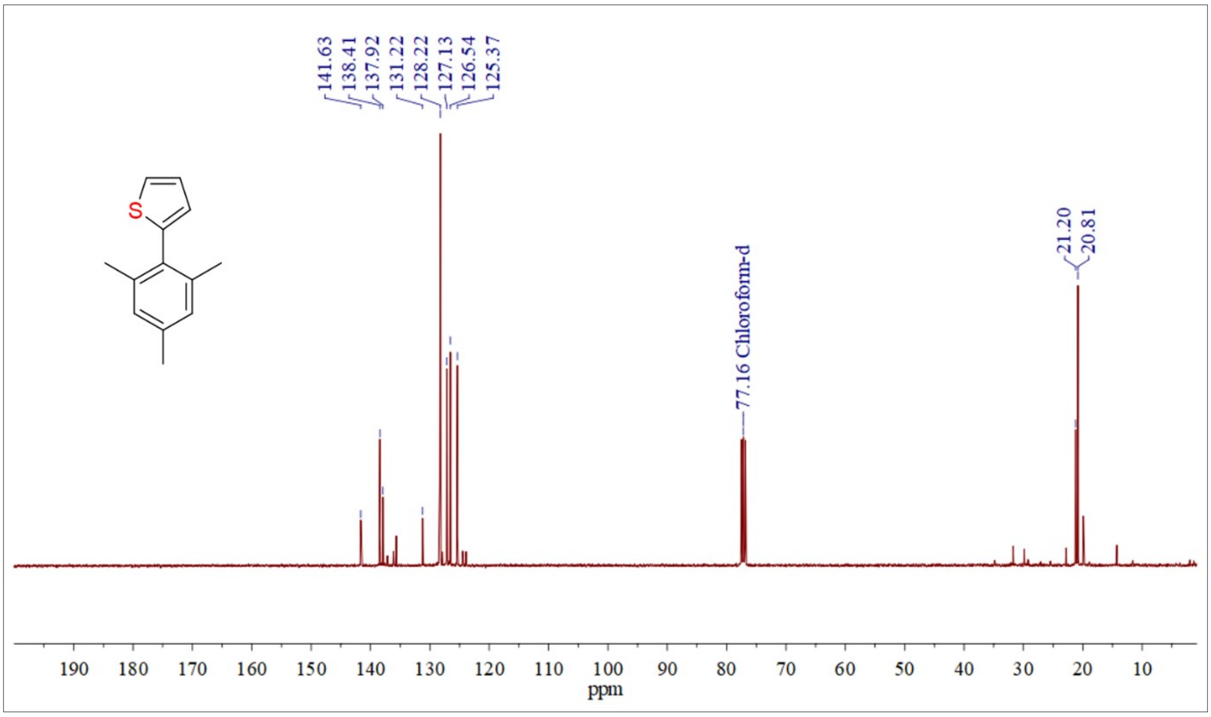

Figure S89. $^{13}C\{^{1}H\}$ NMR spectrum of complex **V**.

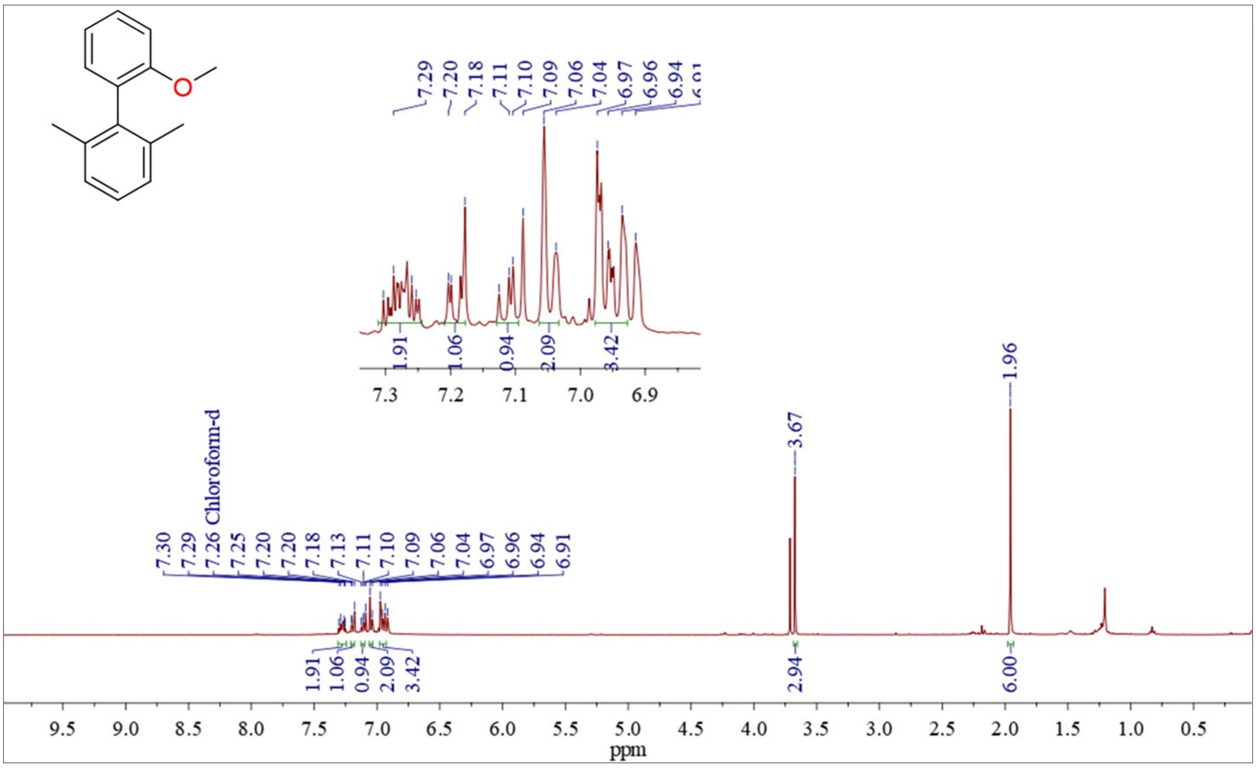

Figure S90. ^{1}H NMR spectrum of complex **VI**.

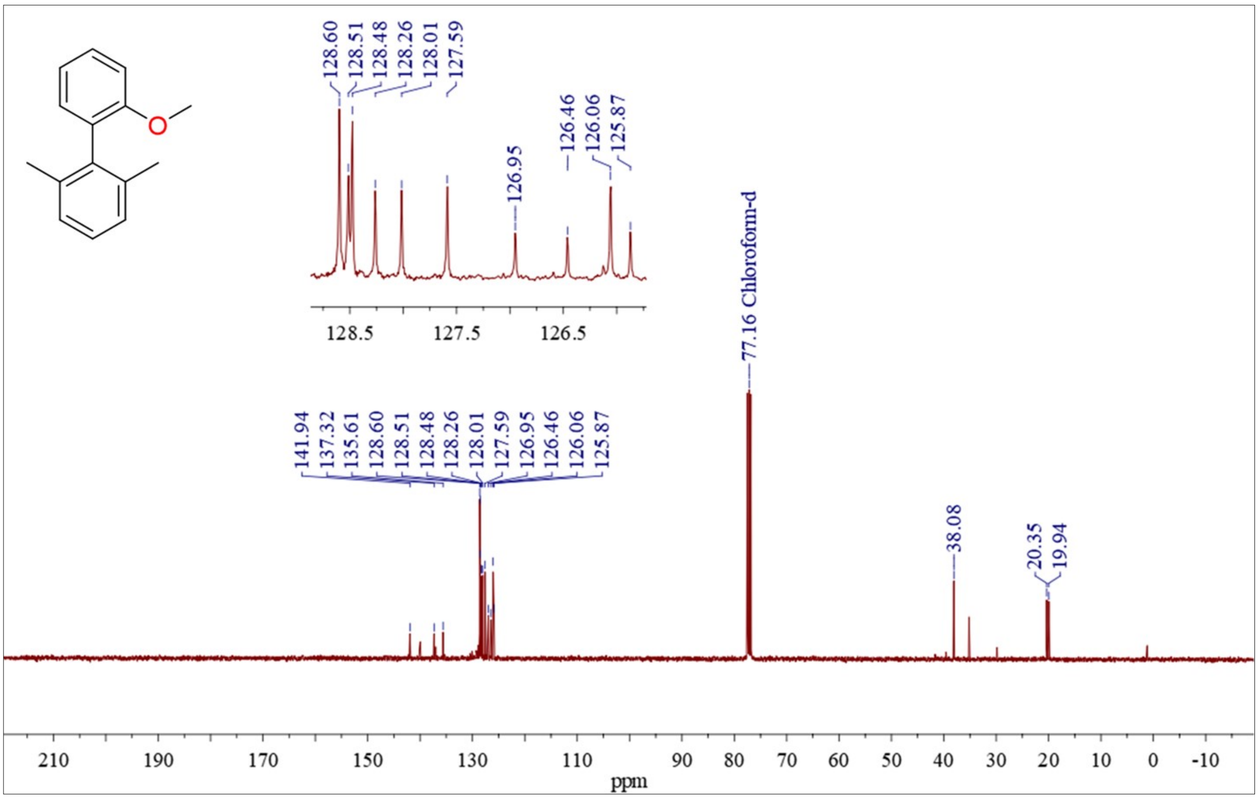

Figure S91. 13C{^{1}H} NMR spectrum of complex **VI**.

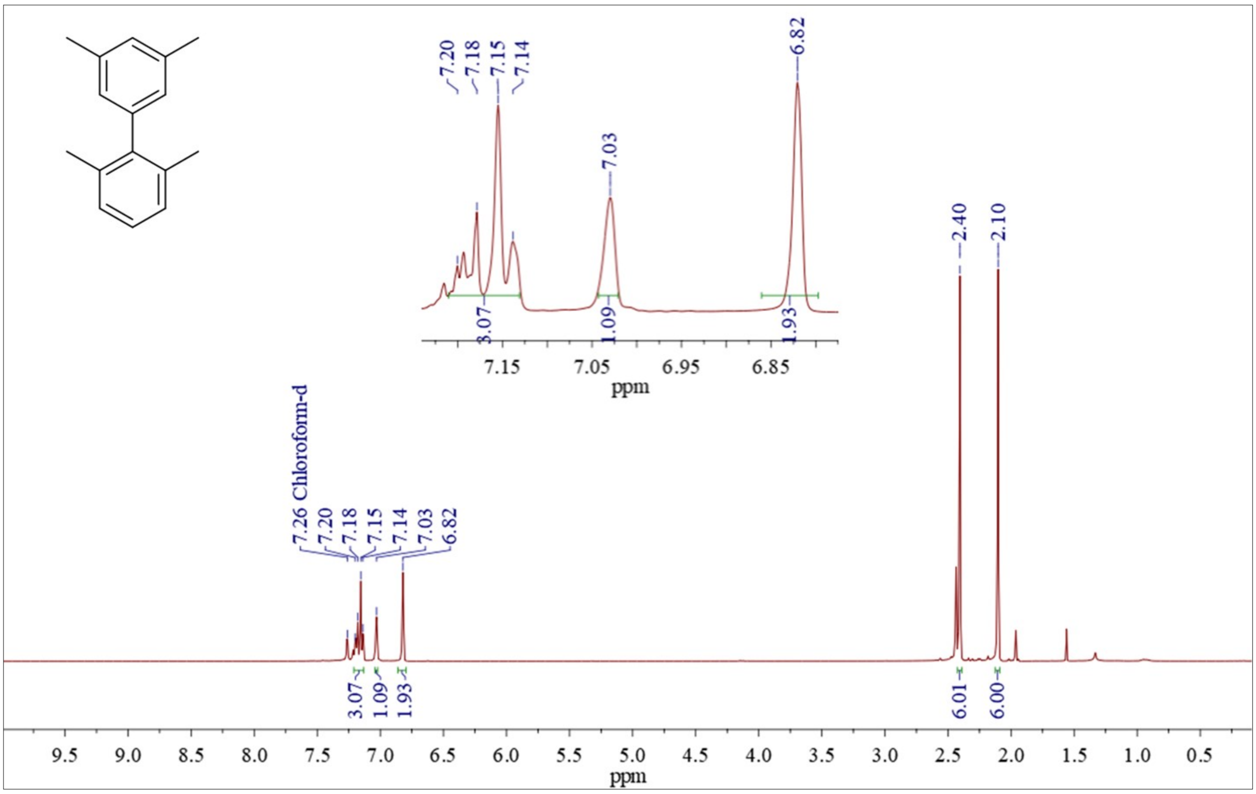

Figure S92. ^{1}H NMR spectrum of complex **VII**.

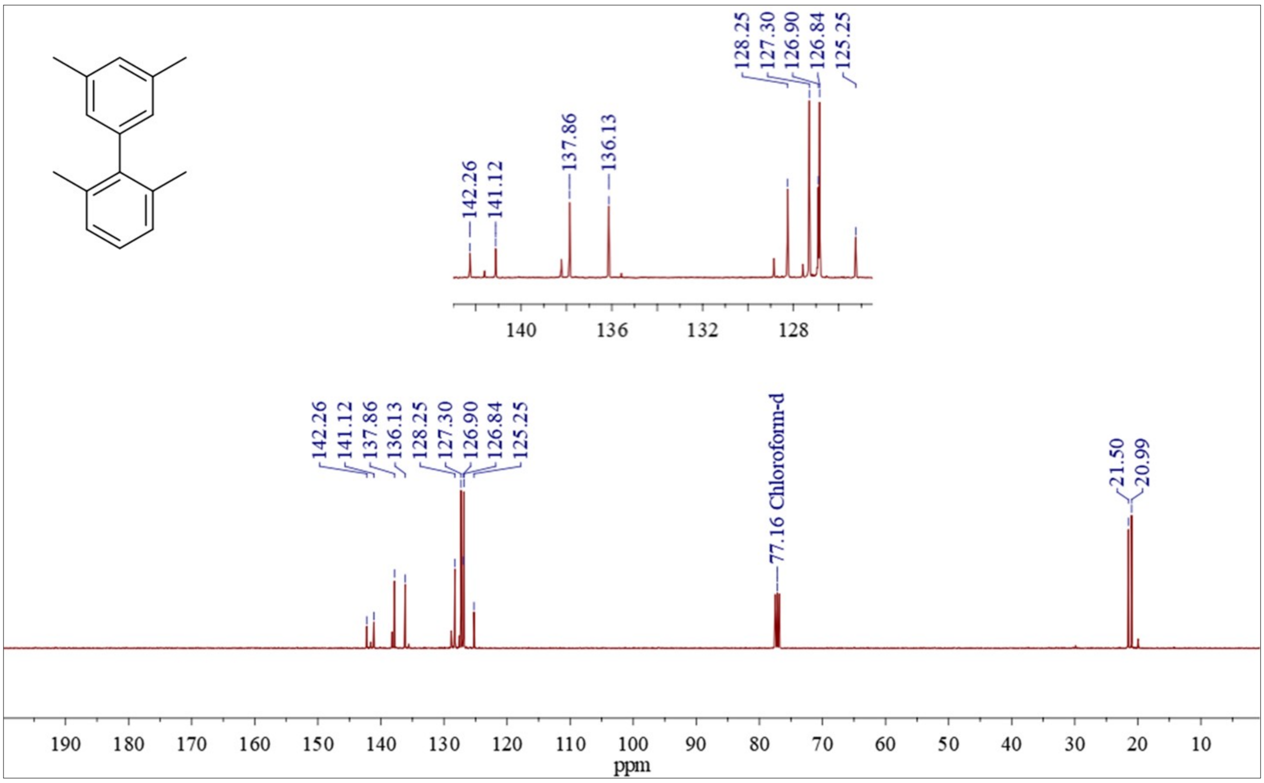

Figure S93. 13C{^{1}H} NMR spectrum of complex **VII**.

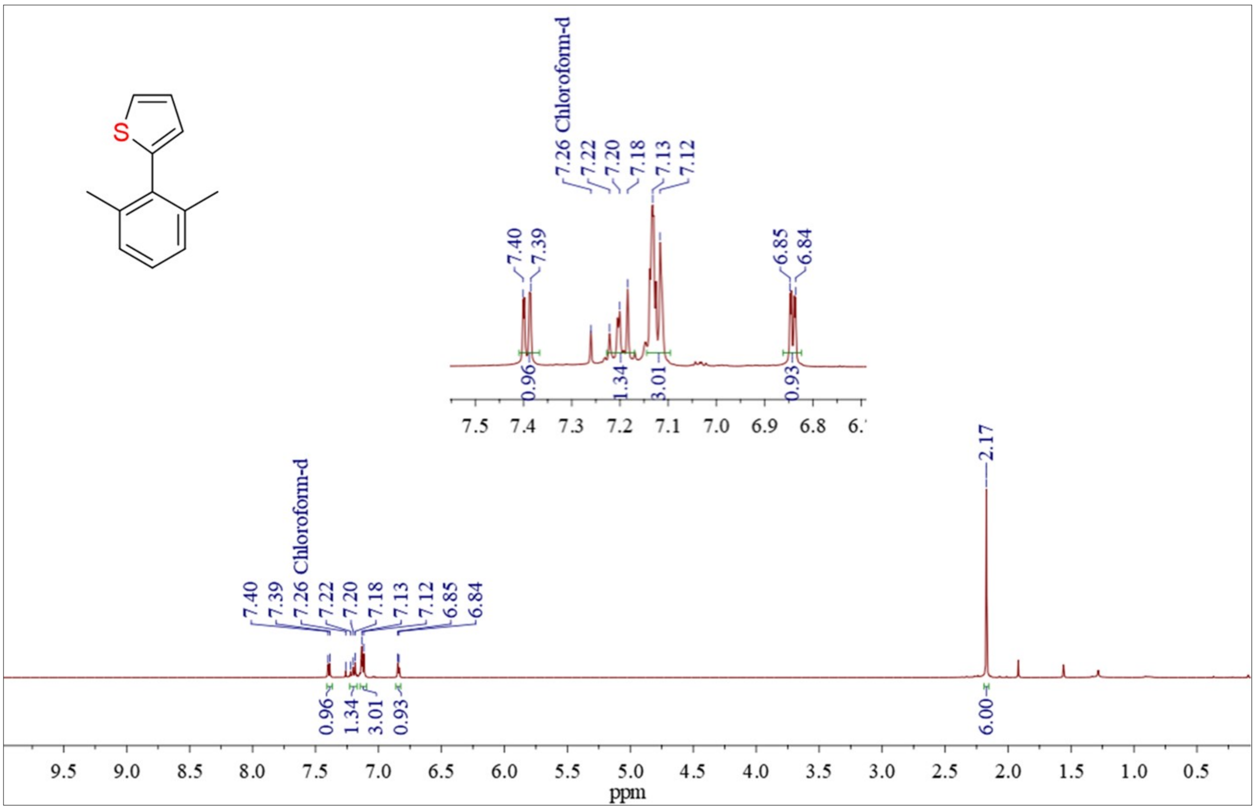

Figure S94. ^{1}H NMR spectrum of complex **VIII**.

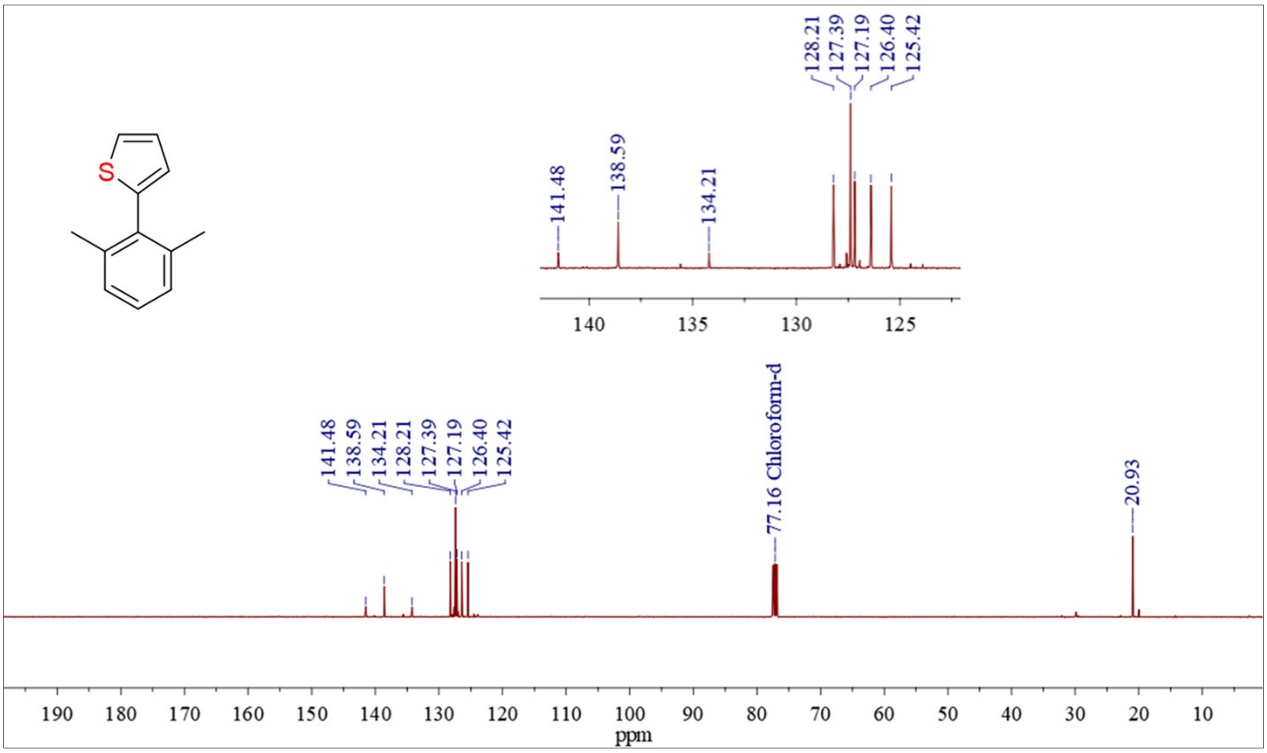

Figure S95. 13C{^{1}H} NMR spectrum of complex **VIII**.

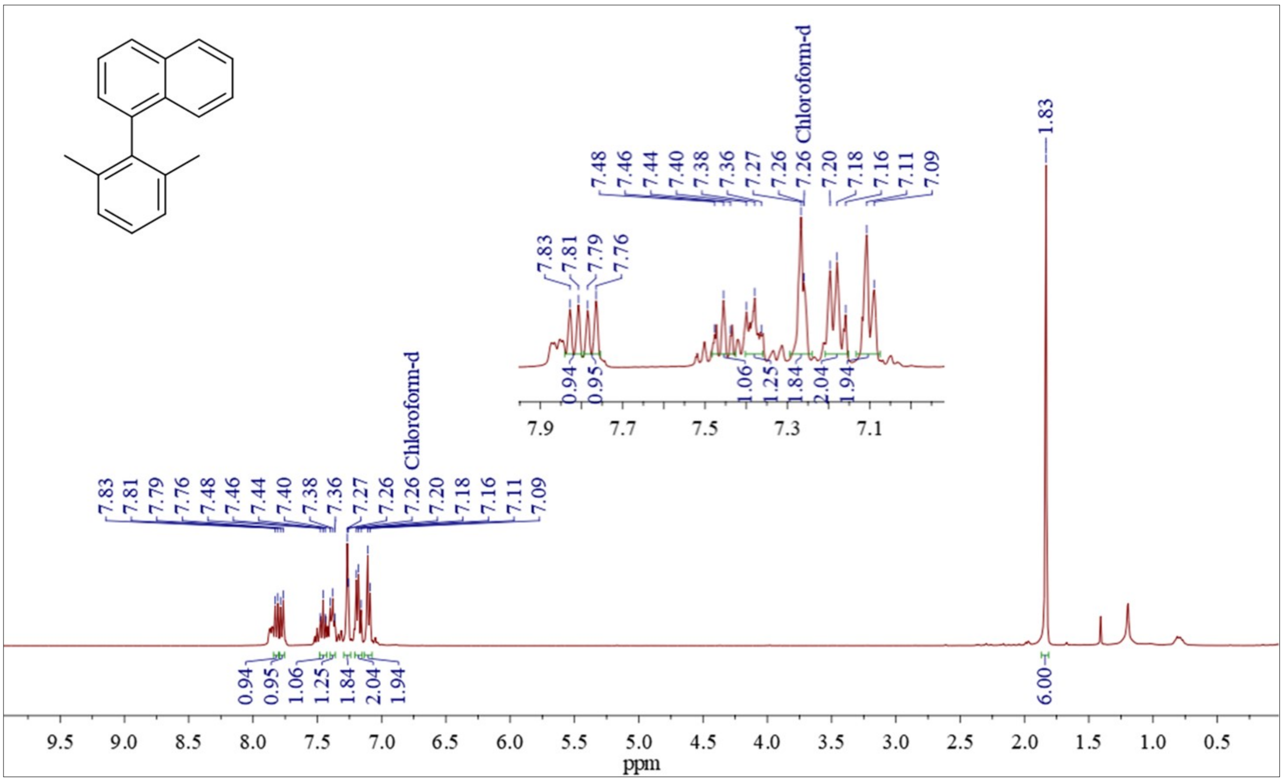

Figure S96. ^{1}H NMR spectrum of complex **IX**.

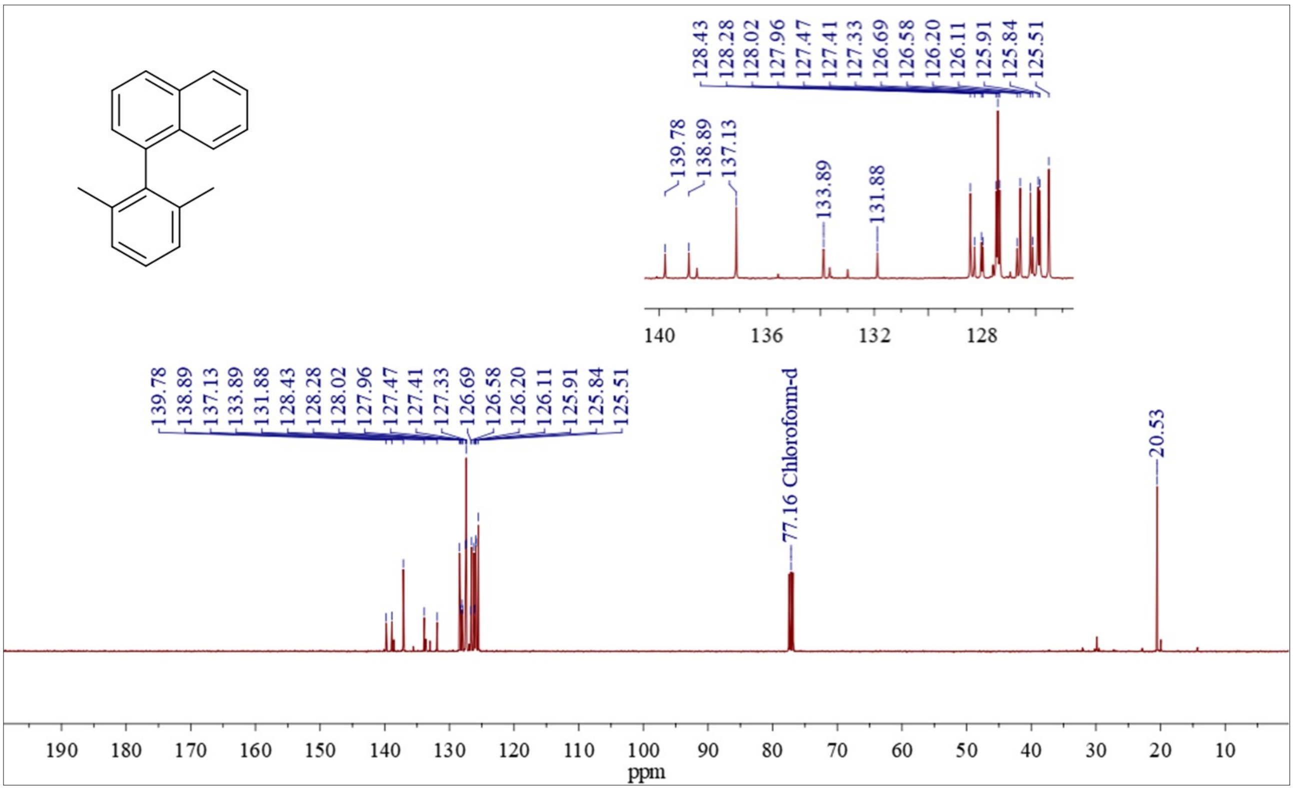

Figure S97. 13C{^{1}H} NMR spectrum of complex **IX**.

S2.3 NMR Spectra of α-aminonitriles

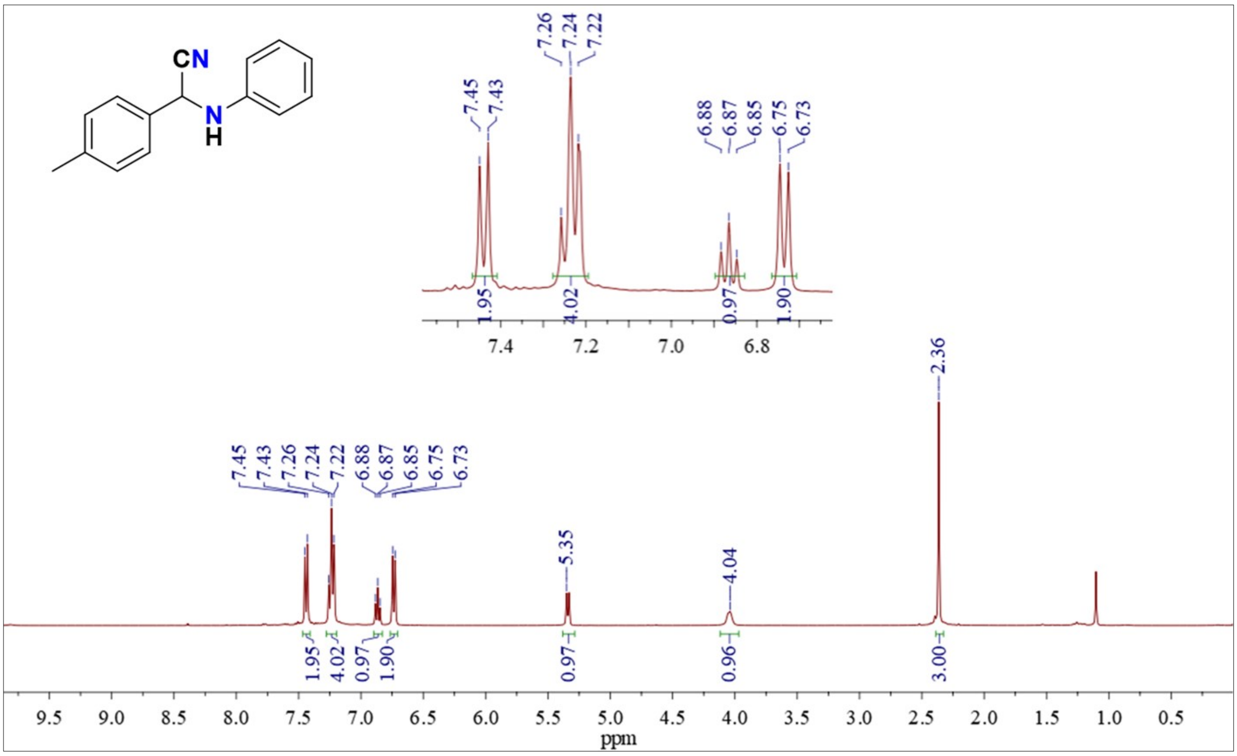

Figure S98. ^{1}H NMR spectrum of complex **X**.

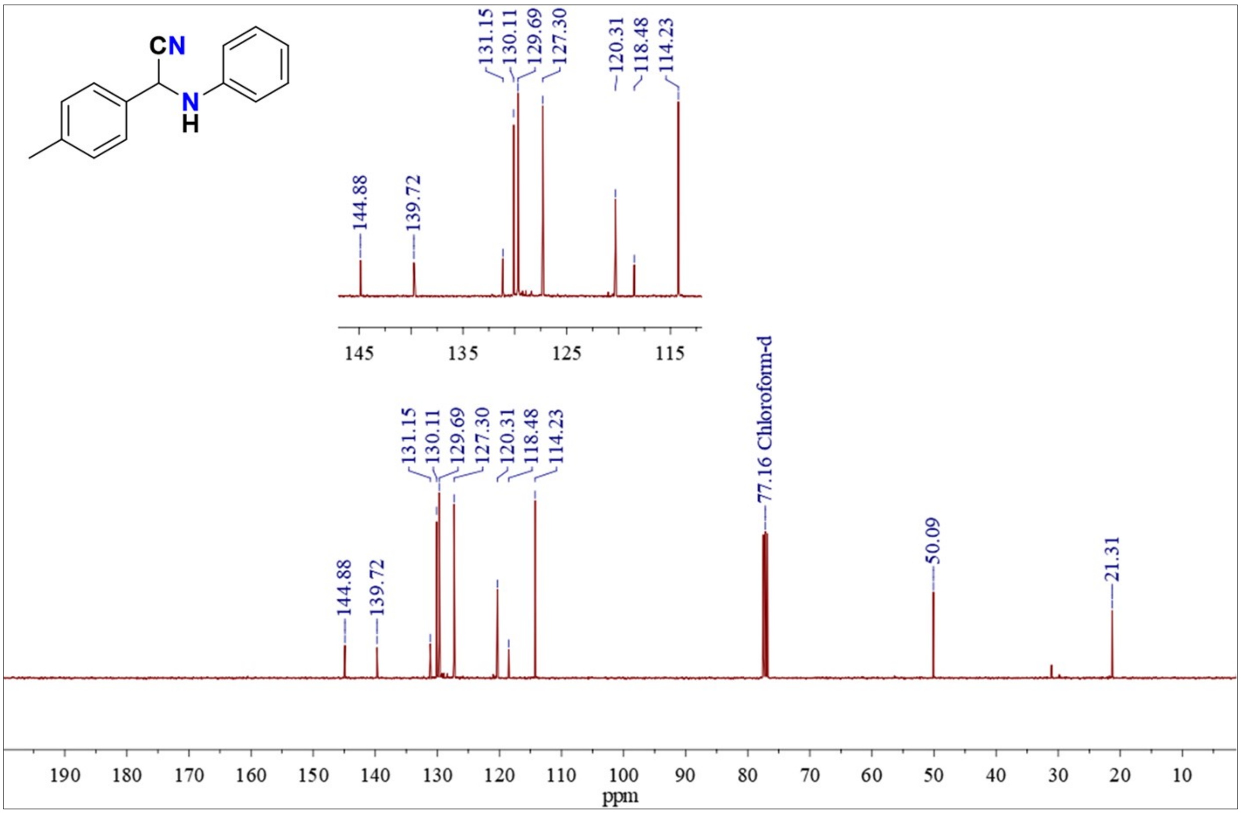

Figure S99. ^{13}C{^{1}H} NMR spectrum of complex **X**.

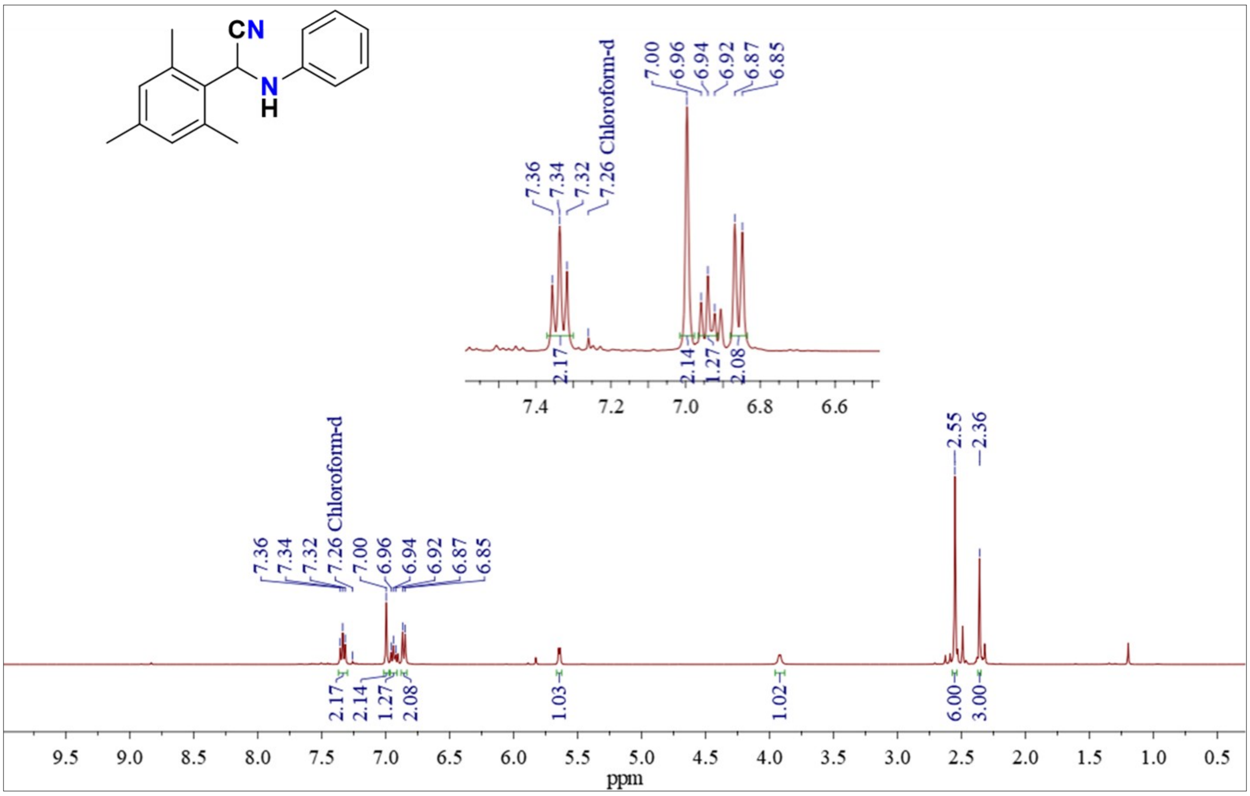

Figure S100. ^{1}H NMR spectrum of complex **XI**.

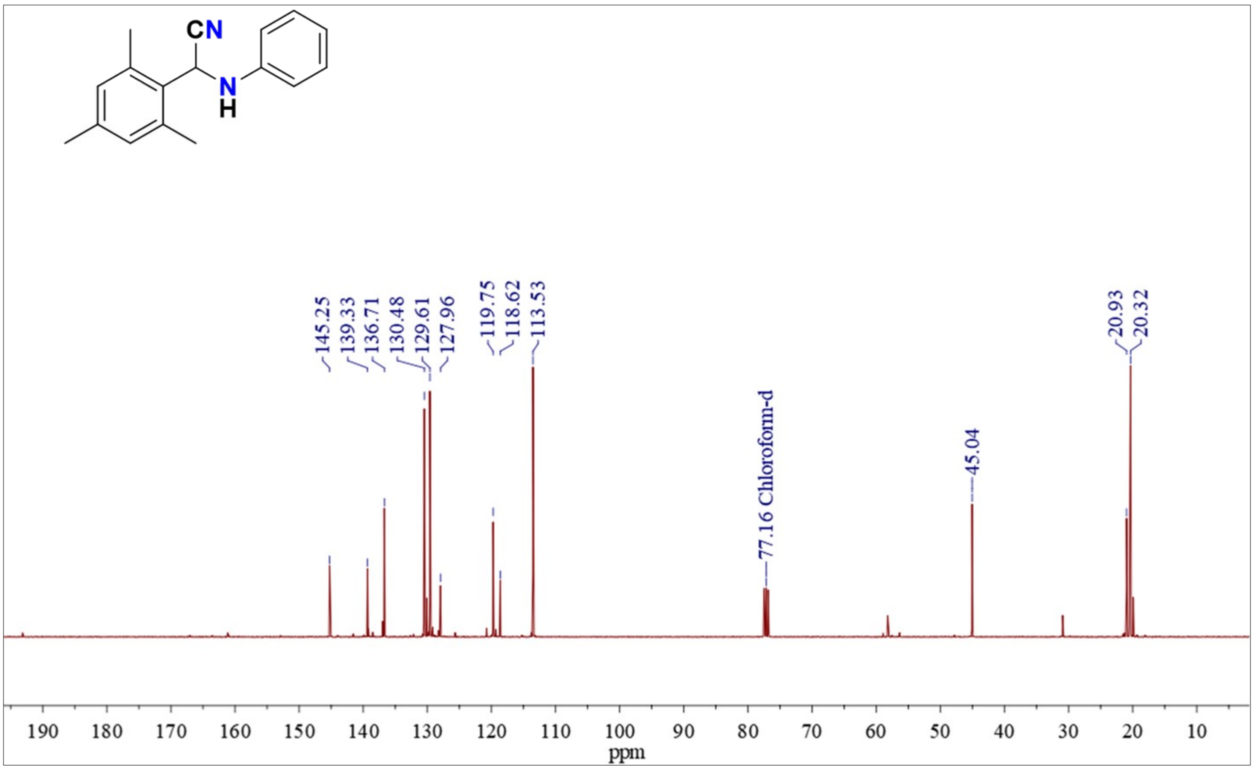

Figure S101. $^{13}C\{^{1}H\}$ NMR spectrum of complex **XI**.

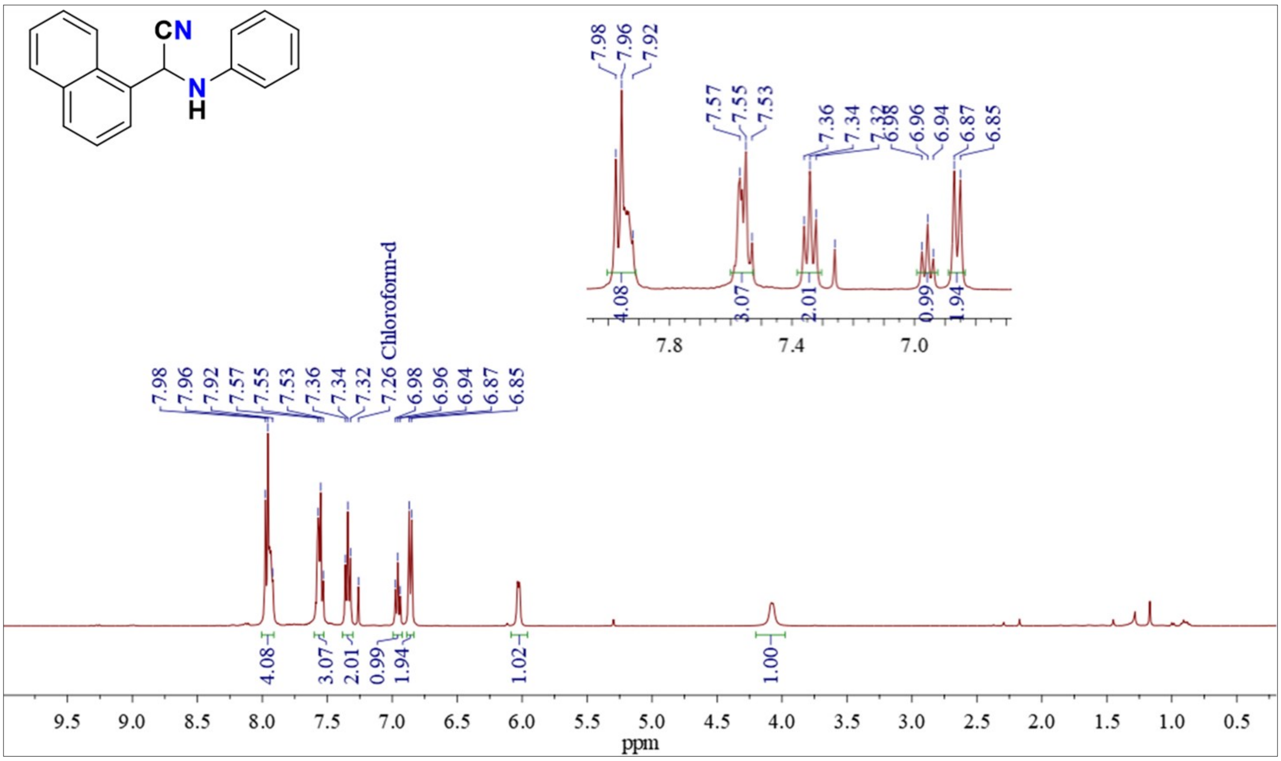

Figure S102. ^{1}H NMR spectrum of complex **XII**.

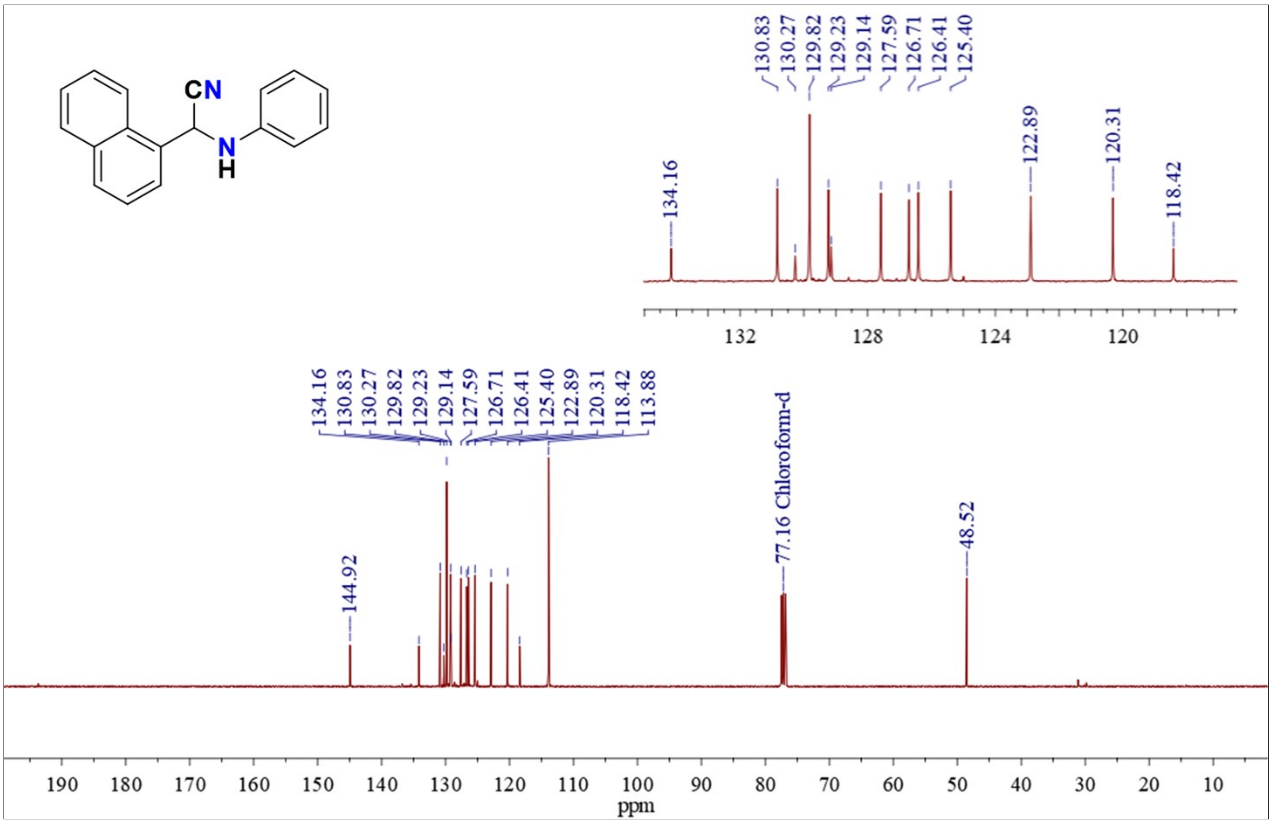

Figure S103. ^{13}C{^{1}H} NMR spectrum of complex **XII**.

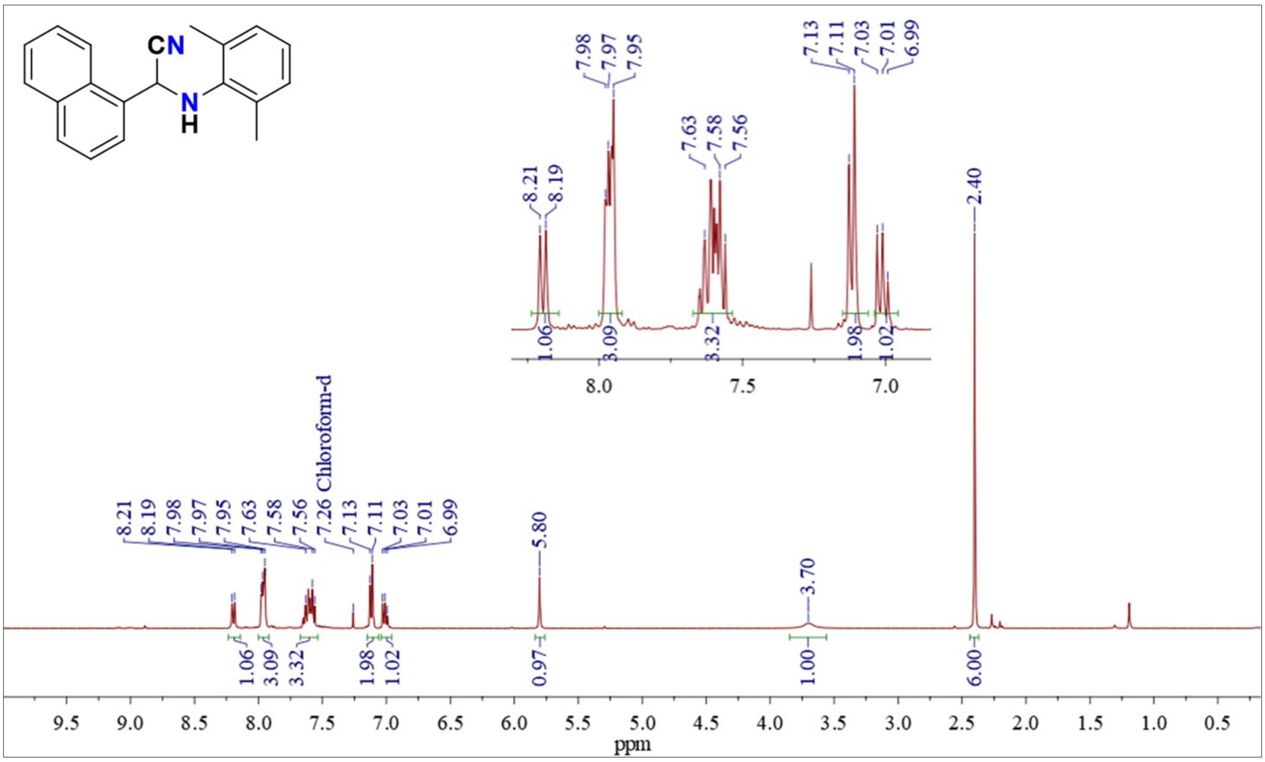

Figure S104. ^{1}H NMR spectrum of complex **XIII**.

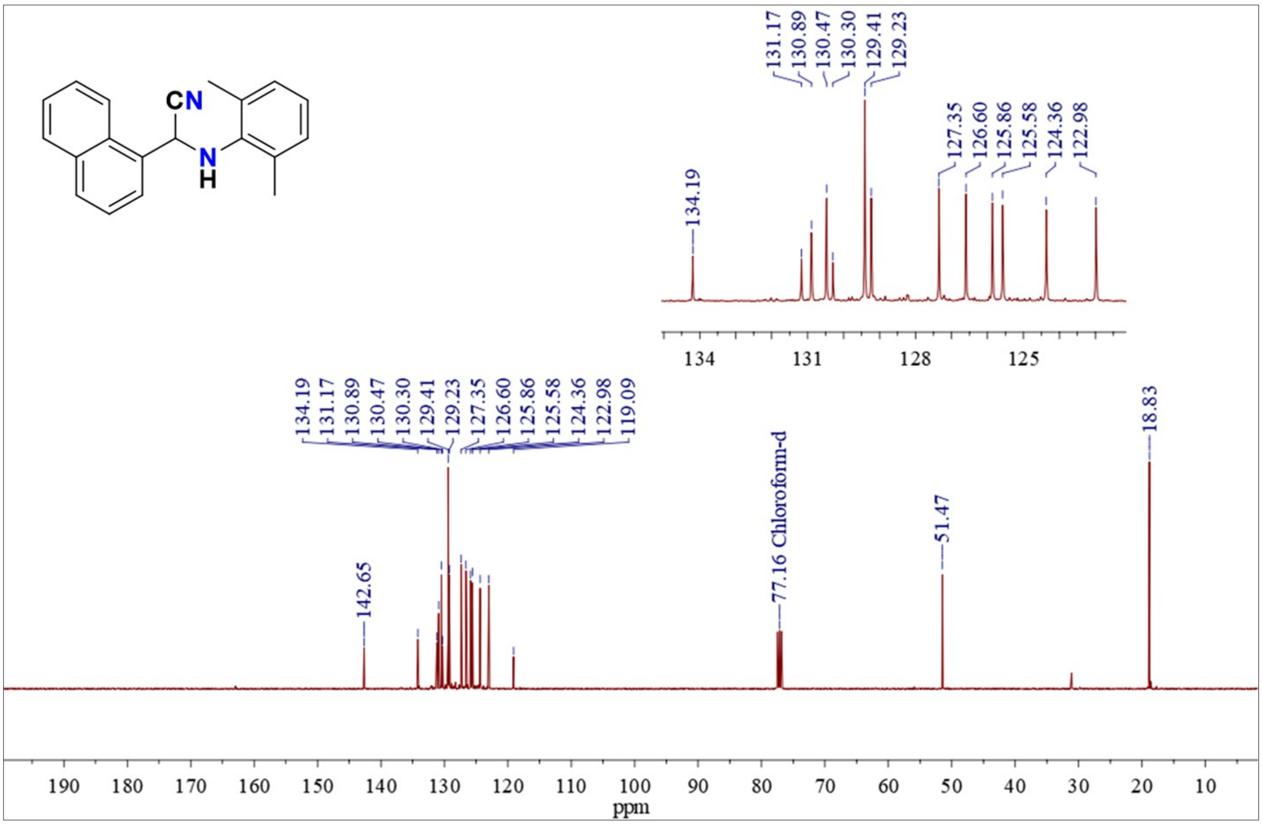

Figure S105. $^{13}C\{^{1}H\}$ NMR spectrum of complex **XIII**.

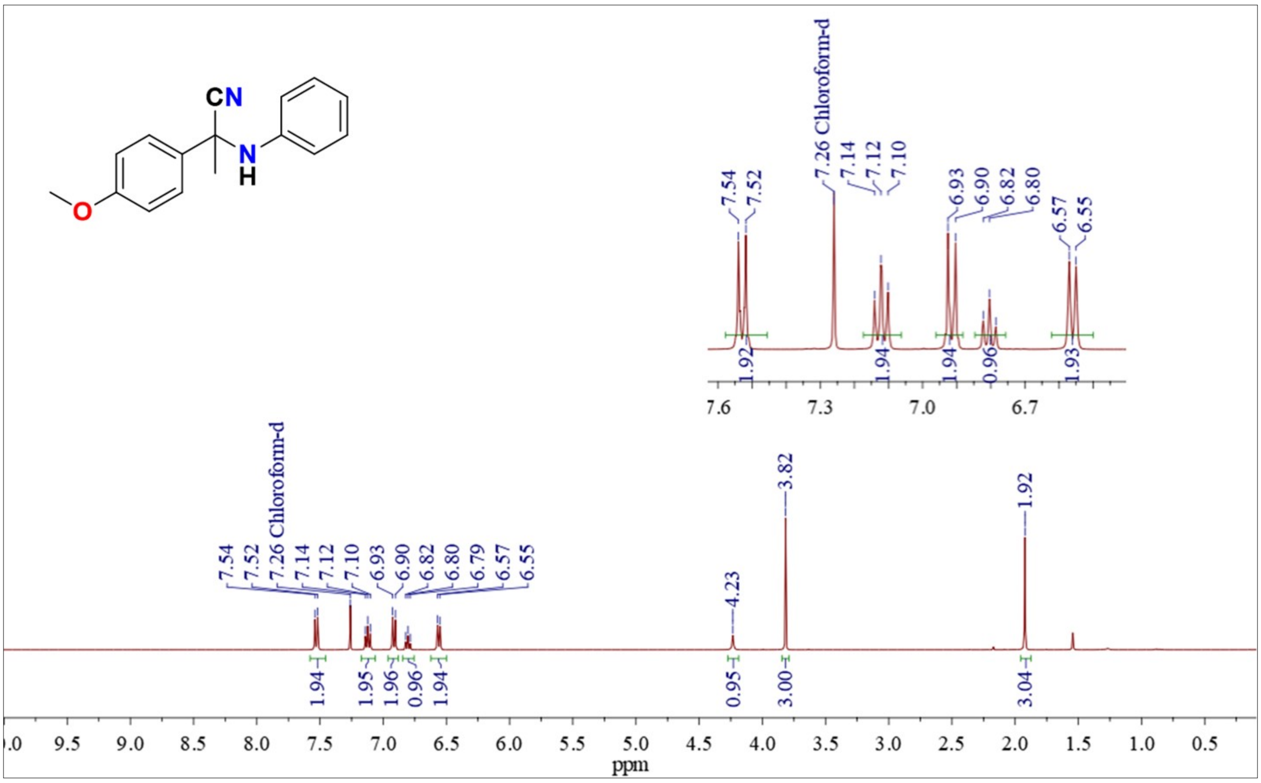

Figure S106. ^{1}H NMR spectrum of complex **XIV**.

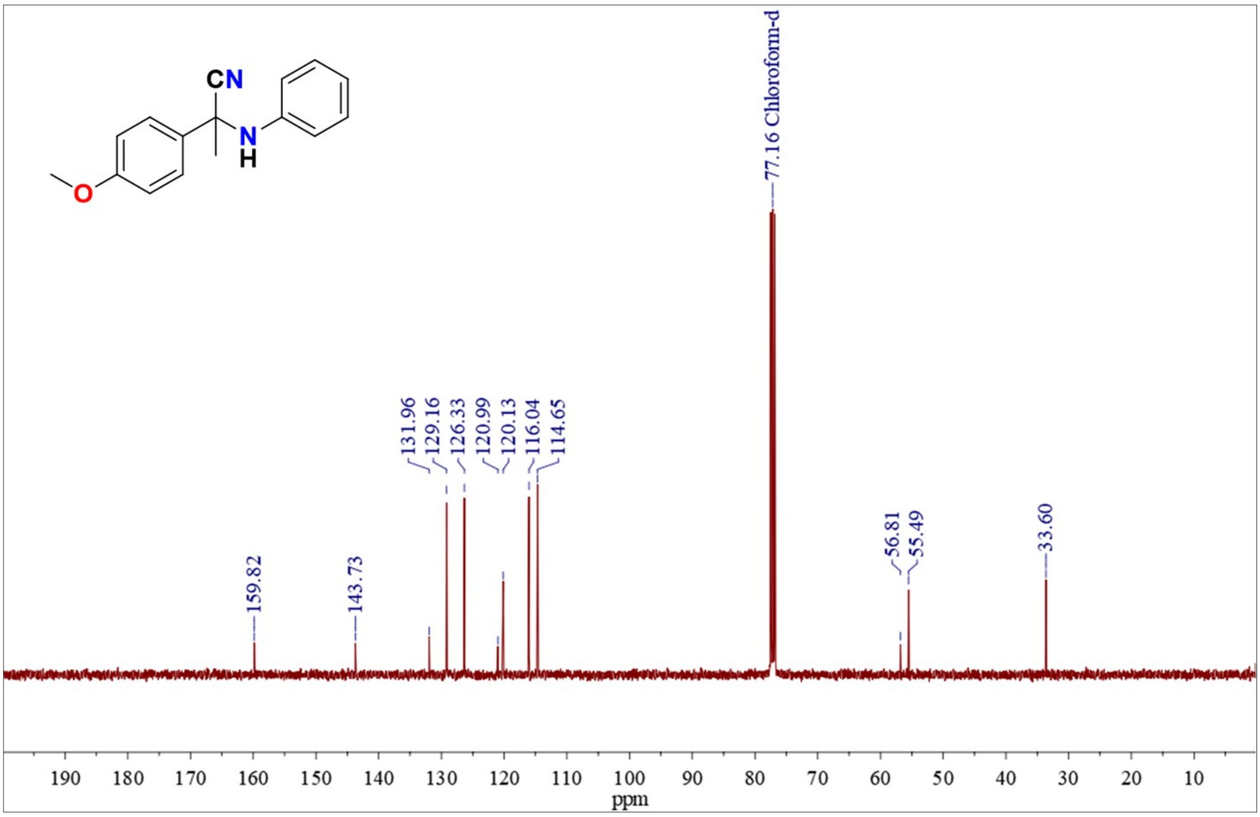

Figure S107. ^{1}H NMR spectrum of complex **XIV**.

❖ DOSY Measurement of Complex 7

Molecules are differentiated based on their diffusion coefficient (D), which correlates with their hydrodynamic radius (R_h).[18] Hereby, the diffusion coefficient D is dependent on the concentration of the sample. Calculations of the hydrodynamic radii were carried out by applying the Stokes-Einstein equation. Rh was calculated using the Stokes–Einstein equation, applying the dynamic viscosity (η) of the pure solvent (benzene or toluene), while minor changes in viscosity caused by the dissolved sample were disregarded. It is important to note that the Stokes–Einstein equation assumes spherical molecules significantly larger than solvent molecules.[19] Moreover, diffusion coefficients (and the corresponding radii) obtained from DOSY measurements are not absolute values and should be interpreted comparatively, only between measurements conducted under identical solvent and concentration conditions. A sample concentration of 0.04 µM has been used.

In Toluene-d_8

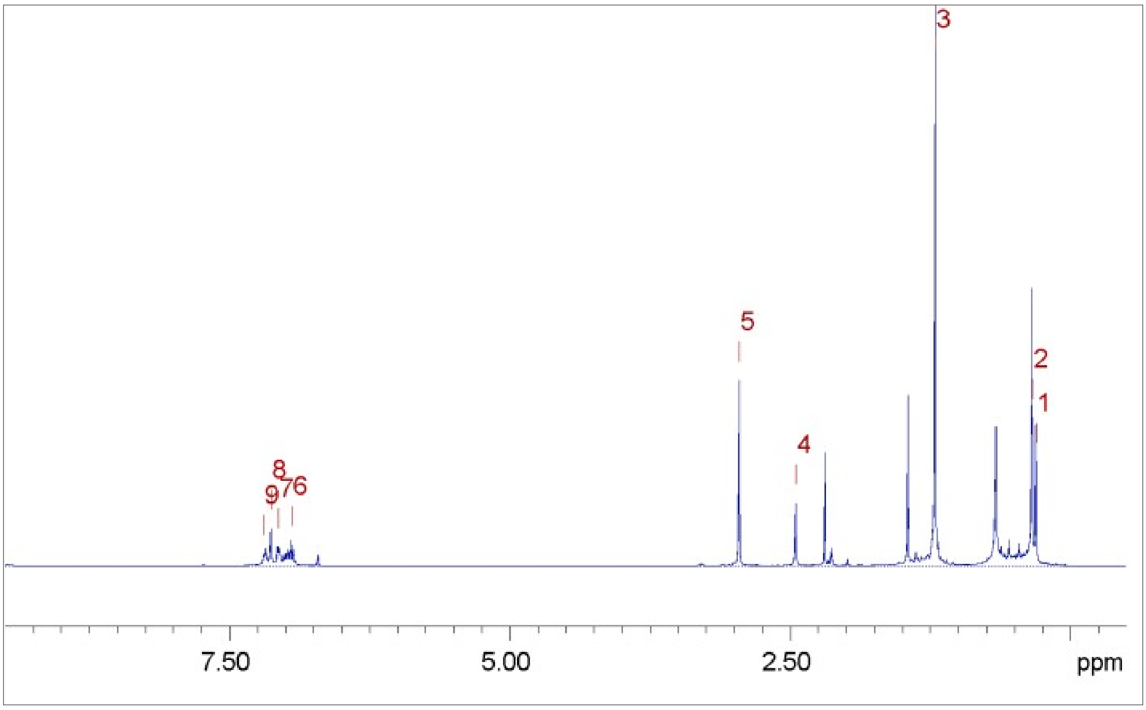

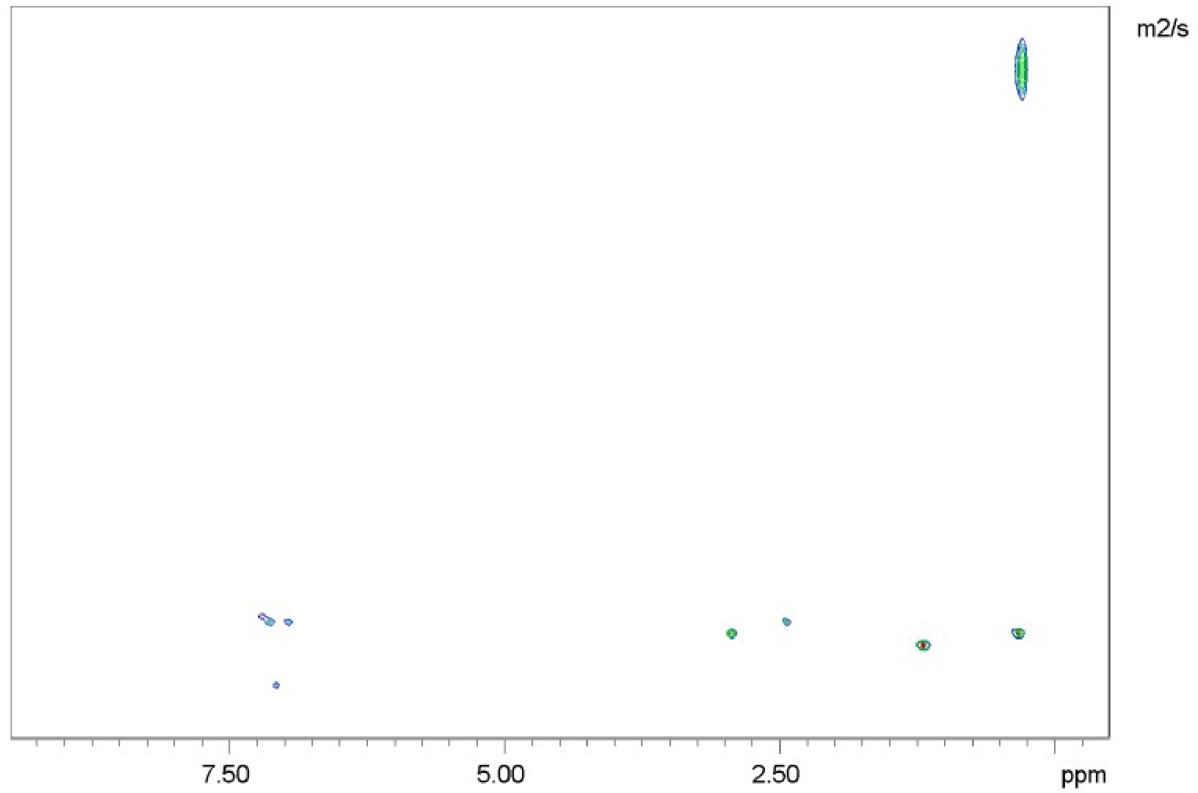

Peak name	F2 [ppm]	D [m2/s]	error	fitInfo
1	0.306	1.07e-10	6.421e-12	Done
2	0.340	6.56e-10	4.004e-12	Done
3	1.202	6.76e-10	3.828e-12	Done
4	2.447	6.34e-10	3.602e-12	Done
5	2.950	6.59e-10	3.786e-12	Done
6	6.944	6.32e-10	3.551e-12	Done
7	7.061	7.75e-10	8.896e-12	Done
8	7.125	6.36e-10	4.286e-12	Done
9	7.190	6.16e-10	5.281e-12	Done

Figure S108. DOSY report of complex **7**, measured in Toluene-d_8 (peak 4) at 298 K.

The hydrodynamic radii, r_H, were determined using the Stokes-Einstein equation,

$$r_H = \frac{k_B \cdot T}{6\pi.\eta_{sol}.D} \text{.................. Equation 1}$$

$$\frac{1 \cdot 380649 * 10^{-23}\frac{Nm}{K} \times 298\,K}{6\pi \times 0.000552\frac{N}{m^2} \times D}$$

$$\frac{1 \cdot 380649 * 10^{-23} \frac{Nm}{K} \times 298\,K}{6\pi \times 0.000552 \frac{N}{m^2} s \times 6.34 * 10^{-10} \frac{m^2}{s}}$$

r_H =6.24 *10^{-10} m = 6.24 Å (approximate molar mass 622 g mol^{-1})

This results in an average 4 % error in molecular weight with the monomer (647 g mol^{-1}) and hence indicates its monomeric presence in solution.

S3. Crystallographic Details of Complexes 2-24.

Crystal data for **2-20** were collected on a Bruker Smart Apex Duo diffractometer at 100 K using Mo Kα radiation (λ = 0.71073 Å). The absorption correction was done using the multi-scan method (SADABS). The structures were solved by direct methods and refined by full-matrix least-squares methods against F2 (SHELXL-2014/6) and Olex[20] with the ShelXT[21] structure solution program. Attempts to refine residual electron density peaks as disordered or partial occupancy solvent toluene carbon atoms were unsuccessful. The data were corrected for disordered electron density using the SQUEEZE procedure implemented in PLATON.[22] Crystallographic data files for the **2-24** have been deposited with the Cambridge Crystallographic Data Centre. CCDC No.: 2304275 (**2**), 2304278 (**3**), 2419144 (**4**), 2450951(**5**) 2304292 (**6**), 2419145 (**7**), 2419147 (**8**), 2304280 (**9**), 2450954 (**10**), 2450956 [PhC{N(^{t}Bu)}$_2$Si{N(Dipp)SiMe$_3$}], 2450959 (**11**) 2450960 (**12**) 2304289 (**13**), 2304286 (**14**), 2325641 (**15**), 2419148 (**16**), 2450961 (**17**) 2304281 (**18**), 2304284 (**19**), 2304285 (**20**), 2304283 (**21**), 2419162 (**22**), 2419149 (**23**), 2419163 (**24**).

Crystal Data and Structure Refinement for Complex 2

Identification code	**2**
Empirical formula	$C_{30}H_{52}CuN_3Si_3$
Formula weight	602.55
Temperature/K	150
Crystal system	monoclinic
Space group	$P2_1/c$
a/Å	8.7311(17)
b/Å	16.101(3)
c/Å	24.398(4)
α/°	90
β/°	96.891(6)

γ/°	90
Volume/Å^3	3405.0(11)
Z	4
ρ_{calc}g/cm^3	1.175
μ/mm^{-1}	0.769
F(000)	1296.0
Crystal size/mm^3	0.25 × 0.13 × 0.05
Radiation	MoKα (λ = 0.71073)
2Θ range for data collection/°	4.208 to 56.704
Index ranges	$-11 \leq h \leq 11$, $-21 \leq k \leq 21$, $-32 \leq l \leq 32$
Reflections collected	139004
Independent reflections	8516 [R_{int} = 0.0965, R_{sigma} = 0.0398]
Data/restraints/parameters	8516/0/350
Goodness-of-fit on F^2	1.056
Final R indexes [I>=2σ (I)]	R_1 = 0.0424, wR_2 = 0.0811
Final R indexes [all data]	R_1 = 0.0623, wR_2 = 0.0885
Largest diff. peak/hole / e Å^{-3}	0.51/-0.34

Crystal Data and Structure Refinement for Complex 3

Identification code	**3**
Empirical formula	$C_{40}H_{64}CuN_3Si_3$
Formula weight	734.75
Temperature/K	150
Crystal system	monoclinic
Space group	$P2_1/c$
a/Å	21.7756(10)
b/Å	9.2187(5)
c/Å	22.1281(12)
α/°	90
β/°	102.979(2)
γ/°	90
Volume/Å^3	4328.6(4)
Z	4
ρ_{calc}g/cm^3	1.127
μ/mm^{-1}	0.616
F(000)	1584.0
Crystal size/mm^3	0.16 × 0.14 × 0.11
Radiation	MoKα (λ = 0.71073)
2Θ range for data collection/°	3.778 to 56.872
Index ranges	$-29 \leq h \leq 28$, $-12 \leq k \leq 12$, $-29 \leq l \leq 29$
Reflections collected	155660
Independent reflections	10866 [R_{int} = 0.1030, R_{sigma} = 0.0482]
Data/restraints/parameters	10866/0/453

Goodness-of-fit on F^2	1.150
Final R indexes [I>=2σ (I)]	R_1 = 0.0537, wR_2 = 0.1138
Final R indexes [all data]	R_1 = 0.0887, wR_2 = 0.1332
Largest diff. peak/hole / e $Å^{-3}$	0.77/-0.49

<u>Crystal Data and Structure Refinement for Complex 4</u>

Identification code	**4**
Empirical formula	$C_{33}H_{58}CuN_3Si_3$
Formula weight	644.63
Temperature/K	100
Crystal system	triclinic
Space group	P-1
a/Å	12.089(7)
b/Å	12.897(8)
c/Å	13.780(8)
α/°	85.671(14)
β/°	71.467(15)
γ/°	66.640(15)
Volume/$Å^3$	1866.9(19)
Z	2
ρ_{calc}g/cm^3	1.147
μ/mm^{-1}	0.705
F(000)	696.0
Crystal size/mm^3	0.15 × 0.09 × 0.06
Radiation	MoKα (λ = 0.71073)
2Θ range for data collection/°	3.122 to 56.65
Index ranges	$-16 \le h \le 14$, $-17 \le k \le 17$, $-17 \le l \le 18$
Reflections collected	18822
Independent reflections	9183 [R_{int} = 0.0690, R_{sigma} = 0.1327]
Data/restraints/parameters	9183/0/377
Goodness-of-fit on F^2	0.983
Final R indexes [I>=2σ (I)]	R_1 = 0.0564, wR_2 = 0.1163
Final R indexes [all data]	R_1 = 0.1225, wR_2 = 0.1515
Largest diff. peak/hole / e $Å^{-3}$	0.53/-0.63

<u>Crystal Data and Structure Refinement for Complex 5</u>

Identification code	**5**
Empirical formula	$C_{65}H_{100}Cu_2N_6Si_6$
Formula weight	1261.12
Temperature/K	150
Crystal system	orthorhombic

Space group	Iba2
a/Å	25.182(8)
b/Å	13.756(4)
c/Å	20.876(7)
α/°	90
β/°	90
γ/°	90
Volume/Å^3	7232(4)
Z	4
ρ_{calc}g/cm^3	1.158
μ/mm^{-1}	0.727
F(000)	2696.0
Crystal size/mm^3	0.12 × 0.12 × 0.1
Radiation	MoKα (λ = 0.71073)
2Θ range for data collection/°	4.8 to 56.752
Index ranges	$-33 \leq h \leq 33$, $-18 \leq k \leq 18$, $-27 \leq l \leq 27$
Reflections collected	134864
Independent reflections	9041 [R_{int} = 0.0813, R_{sigma} = 0.0337]
Data/restraints/parameters	9041/1/380
Goodness-of-fit on F^2	1.046
Final R indexes [I>=2σ (I)]	R_1 = 0.0379, wR_2 = 0.0995
Final R indexes [all data]	R_1 = 0.0418, wR_2 = 0.1046
Largest diff. peak/hole / e Å^{-3}	0.68/-1.60
Flack parameter	0.172(5)

Crystal Data and Structure Refinement for Complex 6

Identification code	**6**
Empirical formula	$C_{39}H_{63}Ag_2N_3Si_3$
Formula weight	873.93
Temperature/K	296
Crystal system	triclinic
Space group	P-1
a/Å	10.0668(17)
b/Å	12.265(2)
c/Å	19.889(4)
α/°	78.550(5)
β/°	77.056(5)
γ/°	65.830(4)
Volume/Å^3	2167.6(7)
Z	2
ρ_{calc}g/cm^3	1.339
μ/mm^{-1}	1.015
F(000)	908.0

Crystal size/mm^3	0.16 × 0.13 × 0.08
Radiation	MoKα (λ = 0.71073)
2Θ range for data collection/°	4.008 to 56.622
Index ranges	$-13 \leq h \leq 13$, $-16 \leq k \leq 16$, $-26 \leq l \leq 26$
Reflections collected	110264
Independent reflections	10769 [R_{int} = 0.0590, R_{sigma} = 0.0299]
Data/restraints/parameters	10769/0/443
Goodness-of-fit on F^2	1.152
Final R indexes [I>=2σ (I)]	R_1 = 0.0329, wR_2 = 0.0738
Final R indexes [all data]	R_1 = 0.0457, wR_2 = 0.0847
Largest diff. peak/hole / e $Å^{-3}$	0.70/-0.57

Crystal Data and Structure Refinement for Complex 7

Identification code	**7**
Empirical formula	$C_{36}H_{64}AgN_3Si_3$
Formula weight	731.04
Temperature/K	100
Crystal system	monoclinic
Space group	$P2_1/c$
a/Å	9.365(5)
b/Å	20.278(11)
c/Å	21.736(12)
α/°	90
β/°	97.745(7)
γ/°	90
Volume/$Å^3$	4090(4)
Z	4
ρ_{calc}g/cm^3	1.187
μ/mm^{-1}	0.607
F(000)	1560.0
Crystal size/mm^3	0.14 × 0.09 × 0.05
Radiation	MoKα (λ = 0.71073)
2Θ range for data collection/°	3.782 to 57.442
Index ranges	$-12 \leq h \leq 12$, $-27 \leq k \leq 27$, $-29 \leq l \leq 29$
Reflections collected	105327
Independent reflections	10482 [R_{int} = 0.0905, R_{sigma} = 0.0449]
Data/restraints/parameters	10482/0/428
Goodness-of-fit on F^2	1.066
Final R indexes [I>=2σ (I)]	R_1 = 0.0390, wR_2 = 0.0834
Final R indexes [all data]	R_1 = 0.0547, wR_2 = 0.0899
Largest diff. peak/hole / e $Å^{-3}$	0.46/-0.77

Crystal Data and Structure Refinement for Complex 8

Identification code	**8**
Empirical formula	$C_{33}H_{58}AgN_3Si_3$
Formula weight	688.96
Temperature/K	100
Crystal system	triclinic
Space group	P-1
a/Å	12.0478(7)
b/Å	13.1539(8)
c/Å	13.8184(9)
α/°	86.515(2)
β/°	71.449(2)
γ/°	67.362(2)
Volume/Å^3	1911.4(2)
Z	2
ρ_{calc}g/cm^3	1.197
μ/mm^{-1}	0.645
F(000)	732.0
Crystal size/mm^3	0.14 × 0.11 × 0.04
Radiation	MoKα (λ = 0.71073)
2Θ range for data collection/°	4.422 to 56.76
Index ranges	-16 ≤ h ≤ 13, -17 ≤ k ≤ 17, -18 ≤ l ≤ 18
Reflections collected	62384
Independent reflections	9528 [R_{int} = 0.0534, R_{sigma} = 0.0319]
Data/restraints/parameters	9528/0/377
Goodness-of-fit on F^2	1.156
Final R indexes [I>=2σ (I)]	R_1 = 0.0335, wR_2 = 0.0860
Final R indexes [all data]	R_1 = 0.0378, wR_2 = 0.0932
Largest diff. peak/hole / e Å^{-3}	0.50/-1.49

Crystal Data and Structure Refinement for Complex 9

Identification code	**9**
Empirical formula	$C_{48}H_{68}Cl_2Cu_2N_4Si_2$
Formula weight	955.22
Temperature/K	296
Crystal system	monoclinic
Space group	P2$_1$/n
a/Å	10.1287(9)
b/Å	13.8838(14)
c/Å	17.7827(18)

α/°	90
β/°	94.649(3)
γ/°	90
Volume/Å^3	2492.5(4)
Z	2
ρ_{calc}g/cm^3	1.273
μ/mm^{-1}	1.044
F(000)	1008.0
Crystal size/mm^3	0.34 × 0.12 × 0.06
Radiation	MoKα (λ = 0.71073)
2Θ range for data collection/°	4.596 to 49.996
Index ranges	-12 ≤ h ≤ 12, -16 ≤ k ≤ 16, -21 ≤ l ≤ 21
Reflections collected	122744
Independent reflections	4387 [R_{int} = 0.0786, R_{sigma} = 0.0250]
Data/restraints/parameters	4387/0/311
Goodness-of-fit on F^2	1.041
Final R indexes [I>=2σ (I)]	R_1 = 0.0572, wR_2 = 0.1545
Final R indexes [all data]	R_1 = 0.0746, wR_2 = 0.1691
Largest diff. peak/hole / e Å^{-3}	1.47/-0.62

Crystal Data and Structure Refinement for Complex 10

Identification code	**10**
Empirical formula	$C_{56}H_{86}Cu_2N_4O_2Si_2$
Formula weight	1030.54
Temperature/K	150
Crystal system	triclinic
Space group	P-1
a/Å	10.836(3)
b/Å	11.503(3)
c/Å	11.911(3)
α/°	100.995(7)
β/°	101.007(8)
γ/°	99.443(7)
Volume/Å^3	1399.4(6)
Z	1
ρ_{calc}g/cm^3	1.223
μ/mm^{-1}	0.845
F(000)	552.0
Crystal size/mm^3	0.15 × 0.14 × 0.12
Radiation	MoKα (λ = 0.71073)
2Θ range for data collection/°	3.688 to 56.79
Index ranges	-14 ≤ h ≤ 14, -12 ≤ k ≤ 15, -15 ≤ l ≤ 15
Reflections collected	31981

Independent reflections	6927 [R_{int} = 0.1251, R_{sigma} = 0.1045]
Data/restraints/parameters	6927/0/310
Goodness-of-fit on F^2	1.030
Final R indexes [I>=2σ (I)]	R_1 = 0.0868, wR_2 = 0.2066
Final R indexes [all data]	R_1 = 0.1287, wR_2 = 0.2342
Largest diff. peak/hole / e $Å^{-3}$	1.59/-1.59

Crystal Data and Structure Refinement for Complex [PhC{N(^{t}Bu)}$_2$Si{N(Dipp)SiMe$_3$}]

Identification code	[PhC{N(^{t}Bu)}$_2$Si{N(Dipp)SiMe$_3$}]
Empirical formula	$C_{127}H_{196}N_{12}Si_8$
Formula weight	2115.67
Temperature/K	273.15
Crystal system	monoclinic
Space group	$P2_1/n$
a/Å	10.070(4)
b/Å	39.582(15)
c/Å	16.882(6)
α/°	90
β/°	102.573(7)
γ/°	90
Volume/Å^3	6568(4)
Z	2
ρ_{calc}g/cm^3	1.070
μ/mm^{-1}	0.131
F(000)	2308.0
Radiation	MoKα (λ = 0.71073)
2Θ range for data collection/°	2.058 to 58.072
Index ranges	$-13 \le h \le 13$, $-53 \le k \le 53$, $-23 \le l \le 22$
Reflections collected	135403
Independent reflections	17298 [R_{int} = 0.0627, R_{sigma} = 0.0382]
Data/restraints/parameters	17298/178/720
Goodness-of-fit on F^2	1.127
Final R indexes [I>=2σ (I)]	R_1 = 0.0608, wR_2 = 0.1349
Final R indexes [all data]	R_1 = 0.0773, wR_2 = 0.1438
Largest diff. peak/hole / e $Å^{-3}$	0.81/-0.41

Crystal Data and Structure Refinement for Complex 11

Identification code	**11**
Empirical formula	$C_{39}H_{60}CuN_3Si_2$
Formula weight	690.62
Temperature/K	296.15

Crystal system	monoclinic
Space group	$P2_1/c$
a/Å	9.4686(17)
b/Å	21.957(5)
c/Å	19.381(4)
α/°	90
β/°	99.170(5)
γ/°	90
Volume/Å^3	3978.0(14)
Z	4
ρ_{calc}g/cm^3	1.153
μ/mm^{-1}	0.638
F(000)	1488.0
Radiation	MoKα (λ = 0.71073)
2Θ range for data collection/°	3.71 to 56.93
Index ranges	$-10 \leq h \leq 12$, $-28 \leq k \leq 29$, $-25 \leq l \leq 25$
Reflections collected	88128
Independent reflections	10000 [R_{int} = 0.1272, R_{sigma} = 0.0846]
Data/restraints/parameters	10000/0/422
Goodness-of-fit on F^2	1.061
Final R indexes [I>=2σ (I)]	R_1 = 0.0607, wR_2 = 0.0948
Final R indexes [all data]	R_1 = 0.1151, wR_2 = 0.1110
Largest diff. peak/hole / e Å^{-3}	0.44/-0.45

Crystal Data and Structure Refinement for Complex 13

Identification code	**13**
Empirical formula	$C_{15}H_{15}BO_2$
Formula weight	238.08
Temperature/K	150
Crystal system	orthorhombic
Space group	Pnma
a/Å	23.369(4)
b/Å	11.3535(19)
c/Å	4.5612(7)
α/°	90
β/°	90
γ/°	90
Volume/Å^3	1210.2(3)
Z	4
ρ_{calc}g/cm^3	1.307
μ/mm^{-1}	0.084
F(000)	504.0
Crystal size/mm^3	0.14 × 0.11 × 0.08

Radiation	MoKα (λ = 0.71073)
2Θ range for data collection/°	6.974 to 56.672
Index ranges	-31 ≤ h ≤ 31, -15 ≤ k ≤ 15, -6 ≤ l ≤ 5
Reflections collected	29948
Independent reflections	1570 [R_{int} = 0.0575, R_{sigma} = 0.0225]
Data/restraints/parameters	1570/0/90
Goodness-of-fit on F^2	1.101
Final R indexes [I>=2σ (I)]	R_1 = 0.0439, wR_2 = 0.0975
Final R indexes [all data]	R_1 = 0.0560, wR_2 = 0.1028
Largest diff. peak/hole / e Å^{-3}	0.26/-0.27

Crystal Data and Structure Refinement for Complex 14

Identification code	**14**
Empirical formula	$C_{31}H_{36}CuN_3SeSi_3$
Formula weight	677.40
Temperature/K	100
Crystal system	monoclinic
Space group	C2/c
a/Å	17.820(3)
b/Å	11.6656(17)
c/Å	31.588(5)
α/°	90
β/°	97.787(4)
γ/°	90
Volume/Å^3	6506.2(16)
Z	8
ρ_{calc}g/cm^3	1.383
μ/mm^{-1}	1.925
F(000)	2784.0
Crystal size/mm^3	0.22 × 0.12 × 0.1
Radiation	MoKα (λ = 0.71073)
2Θ range for data collection/°	4.184 to 57.502
Index ranges	-24 ≤ h ≤ 23, -15 ≤ k ≤ 15, -42 ≤ l ≤ 42
Reflections collected	139356
Independent reflections	8370 [R_{int} = 0.1098, R_{sigma} = 0.0594]
Data/restraints/parameters	8370/0/364
Goodness-of-fit on F^2	1.030
Final R indexes [I>=2σ (I)]	R_1 = 0.1030, wR_2 = 0.2301
Final R indexes [all data]	R_1 = 0.1720, wR_2 = 0.2767
Largest diff. peak/hole / e Å^{-3}	1.43/-2.01

Crystal Data and Structure Refinement for Complex 15

Identification code	**15**
Empirical formula	$C_{60}H_{104}Cu_2N_6Se_2Si_6$
Formula weight	1363.03
Temperature/K	150
Crystal system	monoclinic
Space group	$P2_1/n$
a/Å	16.060(3)
b/Å	9.6579(15)
c/Å	22.092(4)
α/°	90
β/°	91.670(5)
γ/°	90
Volume/Å^3	3425.1(10)
Z	2
ρ_{calc}g/cm^3	1.322
μ/mm^{-1}	1.828
F(000)	1432.0
Crystal size/mm^3	0.16 × 0.11 × 0.05
Radiation	MoKα (λ = 0.71073)
2Θ range for data collection/°	3.092 to 57.732
Index ranges	$-21 \leq h \leq 21$, $-12 \leq k \leq 12$, $-29 \leq l \leq 29$
Reflections collected	156003
Independent reflections	8819 [R_{int} = 0.0801, R_{sigma} = 0.0320]
Data/restraints/parameters	8819/0/551
Goodness-of-fit on F^2	1.068
Final R indexes [I>=2σ (I)]	R_1 = 0.0285, wR_2 = 0.0624
Final R indexes [all data]	R_1 = 0.0386, wR_2 = 0.0671
Largest diff. peak/hole / e Å^{-3}	0.55/-0.47

Crystal Data and Structure Refinement for Complex 16

Identification code	**16**
Empirical formula	$C_{60}H_{104}Ag_2N_6Se_2Si_6$
Formula weight	1451.69
Temperature/K	100
Crystal system	monoclinic
Space group	$P2_1/n$
a/Å	9.8038(8)
b/Å	25.5410(17)
c/Å	14.7234(11)
α/°	90
β/°	98.888(2)
γ/°	90
Volume/Å^3	3642.5(5)

Z	2
ρ_{calc}g/cm^3	1.324
μ/mm^{-1}	1.673
F(000)	1504.0
Crystal size/mm^3	0.11 × 0.06 × 0.05
Radiation	MoKα (λ = 0.71073)
2Θ range for data collection/°	4.244 to 56.688
Index ranges	-13 ≤ h ≤ 13, -27 ≤ k ≤ 33, -19 ≤ l ≤ 19
Reflections collected	56264
Independent reflections	9039 [R_{int} = 0.1179, R_{sigma} = 0.1102]
Data/restraints/parameters	9039/0/358
Goodness-of-fit on F^2	1.043
Final R indexes [I>=2σ (I)]	R_1 = 0.0630, wR_2 = 0.1182
Final R indexes [all data]	R_1 = 0.1493, wR_2 = 0.1451
Largest diff. peak/hole / e Å^{-3}	0.85/-0.56

Crystal Data and Structure Refinement for Complex 17

Identification code	**17**
Empirical formula	$C_{28.5}H_{49}CuN_4Si_3$
Formula weight	595.536
Temperature/K	150.00
Crystal system	monoclinic
Space group	Cc
a/Å	8.9008(19)
b/Å	40.360(8)
c/Å	19.289(4)
α/°	90
β/°	101.101(5)
γ/°	90
Volume/Å^3	6799(2)
Z	8
ρ_{calc}g/cm^3	1.164
μ/mm^{-1}	0.770
F(000)	2557.9
Crystal size/mm^3	0.14 × 0.12 × 0.1
Radiation	Mo Kα (λ = 0.71073)
2Θ range for data collection/°	4.04 to 50.5
Index ranges	-10 ≤ h ≤ 10, -45 ≤ k ≤ 48, -23 ≤ l ≤ 20
Reflections collected	53846
Independent reflections	11808 [R_{int} = 0.1412, R_{sigma} = 0.1440]
Data/restraints/parameters	11808/129/636
Goodness-of-fit on F^2	1.068
Final R indexes [I>=2σ (I)]	R_1 = 0.1018, wR_2 = 0.1835

Final R indexes [all data]	$R_1 = 0.1529$, $wR_2 = 0.2047$
Largest diff. peak/hole / e Å^{-3}	1.09/-1.21

<u>Crystal Data and Structure Refinement for Complex 18</u>

Identification code	**18**
Empirical formula	$C_{34}H_{55}CuN_4Si_3$
Formula weight	667.63
Temperature/K	100
Crystal system	triclinic
Space group	P-1
a/Å	12.8772(11)
b/Å	13.0612(11)
c/Å	14.7699(13)
α/°	70.646(3)
β/°	75.242(3)
γ/°	60.717(2)
Volume/Å^3	2031.3(3)
Z	2
ρ_{calc}g/cm^3	1.092
μ/mm^{-1}	0.651
F(000)	716.0
Crystal size/mm^3	0.14 × 0.13 × 0.08
Radiation	MoKα (λ = 0.71073)
2Θ range for data collection/°	3.87 to 56.782
Index ranges	$-17 \leq h \leq 17$, $-17 \leq k \leq 17$, $-19 \leq l \leq 19$
Reflections collected	87633
Independent reflections	10170 [$R_{int} = 0.0652$, $R_{sigma} = 0.0368$]
Data/restraints/parameters	10170/0/394
Goodness-of-fit on F^2	1.025
Final R indexes [I>=2σ (I)]	$R_1 = 0.0376$, $wR_2 = 0.0868$
Final R indexes [all data]	$R_1 = 0.0554$, $wR_2 = 0.0945$
Largest diff. peak/hole / e Å^{-3}	0.52/-0.43

<u>Crystal Data and Structure Refinement for Complex 19</u>

Identification code	**19**
Empirical formula	$C_{35}H_{56}CuN_5Si_3$
Formula weight	694.65
Temperature/K	296
Crystal system	monoclinic
Space group	$P2_1/c$
a/Å	11.5755(12)
b/Å	14.9774(16)
c/Å	22.191(2)

α/°	90
β/°	90.984(3)
γ/°	90
Volume/Å^3	3846.7(7)
Z	4
ρ_{calc}g/cm^3	1.199
μ/mm^{-1}	0.691
F(000)	1488.0
Crystal size/mm^3	0.22 × 0.14 × 0.09
Radiation	MoKα (λ = 0.71073)
2Θ range for data collection/°	4.448 to 56.682
Index ranges	-15 ≤ h ≤ 15, -19 ≤ k ≤ 19, -26 ≤ l ≤ 29
Reflections collected	44646
Independent reflections	9290 [R_{int} = 0.0915, R_{sigma} = 0.0776]
Data/restraints/parameters	9290/0/413
Goodness-of-fit on F^2	1.067
Final R indexes [I>=2σ (I)]	R_1 = 0.0584, wR_2 = 0.1055
Final R indexes [all data]	R_1 = 0.0966, wR_2 = 0.1177
Largest diff. peak/hole / e Å^{-3}	0.51/-0.43

Crystal Data and Structure Refinement for Complex 20

Identification code	**20**
Empirical formula	$C_{46}H_{76}CuN_5Si_3$
Formula weight	846.92
Temperature/K	296
Crystal system	monoclinic
Space group	Cc
a/Å	17.1666(19)
b/Å	13.5100(15)
c/Å	22.268(3)
α/°	90
β/°	102.071(3)
γ/°	90
Volume/Å^3	5050.3(10)
Z	4
ρ_{calc}g/cm^3	1.114
μ/mm^{-1}	0.537
F(000)	1832.0
Crystal size/mm^3	0.15 × 0.1 × 0.06
Radiation	MoKα (λ = 0.71073)
2Θ range for data collection/°	3.74 to 56.834
Index ranges	-22 ≤ h ≤ 22, -18 ≤ k ≤ 18, -29 ≤ l ≤ 29
Reflections collected	116056

Independent reflections	12644 [R_{int} = 0.1001, R_{sigma} = 0.0578]
Data/restraints/parameters	12644/2/517
Goodness-of-fit on F^2	1.030
Final R indexes [I>=2σ (I)]	R_1 = 0.0408, wR_2 = 0.0846
Final R indexes [all data]	R_1 = 0.0598, wR_2 = 0.0917
Largest diff. peak/hole / e $Å^{-3}$	0.64/-0.51
Flack parameter	0.073(10)

Crystal Data and Structure Refinement for Complex 21

Identification code	**21**
Empirical formula	$C_{33}H_{41}CuF_{10}N_4Si_3$
Formula weight	831.51
Temperature/K	150
Crystal system	monoclinic
Space group	$P2_1/n$
a/Å	14.938(3)
b/Å	13.376(2)
c/Å	19.384(4)
α/°	90
β/°	105.935(5)
γ/°	90
Volume/$Å^3$	3724.3(12)
Z	4
ρ_{calc}g/cm^3	1.483
μ/mm^{-1}	0.763
F(000)	1712.0
Crystal size/mm^3	0.16 × 0.08 × 0.06
Radiation	MoKα (λ = 0.71073)
2Θ range for data collection/°	4.028 to 49.998
Index ranges	$-17 \leq h \leq 17$, $-15 \leq k \leq 15$, $-23 \leq l \leq 23$
Reflections collected	108936
Independent reflections	6551 [R_{int} = 0.2102, R_{sigma} = 0.0856]
Data/restraints/parameters	6551/0/472
Goodness-of-fit on F^2	1.043
Final R indexes [I>=2σ (I)]	R_1 = 0.0606, wR_2 = 0.0933
Final R indexes [all data]	R_1 = 0.1140, wR_2 = 0.1091
Largest diff. peak/hole / e $Å^{-3}$	0.60/-0.70

Crystal Data and Structure Refinement for Complex 22

Identification code	**22**
Empirical formula	$C_{33}H_{41}AgF_{10}N_4Si_3$

Formula weight	875.84
Temperature/K	100
Crystal system	monoclinic
Space group	$P2_1/n$
a/Å	15.1058(11)
b/Å	13.7633(8)
c/Å	19.4686(15)
α/°	90
β/°	106.283(2)
γ/°	90
Volume/Å^3	3885.3(5)
Z	4
ρ_{calc}g/cm^3	1.497
μ/mm^{-1}	0.689
F(000)	1784.0
Crystal size/mm^3	0.15 × 0.11 × 0.05
Radiation	MoKα (λ = 0.71073)
2Θ range for data collection/°	4.08 to 56.688
Index ranges	-20 ≤ h ≤ 20, -15 ≤ k ≤ 18, -25 ≤ l ≤ 25
Reflections collected	47256
Independent reflections	9614 [R_{int} = 0.0679, R_{sigma} = 0.0634]
Data/restraints/parameters	9614/0/472
Goodness-of-fit on F^2	1.019
Final R indexes [I>=2σ (I)]	R_1 = 0.0454, wR_2 = 0.0942
Final R indexes [all data]	R_1 = 0.0888, wR_2 = 0.1120
Largest diff. peak/hole / e Å^{-3}	0.55/-0.47

Crystal Data and Structure Refinement for Complex 23

Identification code	**23**
Empirical formula	$C_{33}H_{51}CuN_3PSi_3$
Formula weight	668.54
Temperature/K	100
Crystal system	monoclinic
Space group	$P2_1/c$
a/Å	10.4856(16)
b/Å	26.848(5)
c/Å	13.129(2)
α/°	90
β/°	102.797(5)
γ/°	90
Volume/Å^3	3604.1(11)
Z	4
ρ_{calc}g/cm^3	1.232

μ/mm^{-1}	0.776
F(000)	1424.0
Crystal size/mm^3	0.13 × 0.07 × 0.04
Radiation	MoKα (λ = 0.71073)
2Θ range for data collection/°	4.262 to 56.758
Index ranges	-14 ≤ h ≤ 11, -35 ≤ k ≤ 35, -17 ≤ l ≤ 17
Reflections collected	85099
Independent reflections	8989 [R_{int} = 0.0522, R_{sigma} = 0.0280]
Data/restraints/parameters	8989/0/382
Goodness-of-fit on F^2	1.178
Final R indexes [I>=2σ (I)]	R_1 = 0.0483, wR_2 = 0.1130
Final R indexes [all data]	R_1 = 0.0611, wR_2 = 0.1213
Largest diff. peak/hole / e Å^{-3}	0.65/-0.73

Crystal Data and Structure Refinement for Complex 24

Identification code	**24**
Empirical formula	$C_{66}H_{102}Ag_2N_6P_2Si_6$
Formula weight	1425.75
Temperature/K	100
Crystal system	monoclinic
Space group	$P2_1/c$
a/Å	10.4355(10)
b/Å	27.531(3)
c/Å	13.1929(11)
α/°	90
β/°	103.102(2)
γ/°	90
Volume/Å^3	3691.6(6)
Z	2
ρ_{calc}g/cm^3	1.283
μ/mm^{-1}	0.712
F(000)	1496.0
Crystal size/mm^3	0.16 × 0.12 × 0.05
Radiation	MoKα (λ = 0.71073)
2Θ range for data collection/°	4.272 to 56.682
Index ranges	-13 ≤ h ≤ 13, -36 ≤ k ≤ 36, -17 ≤ l ≤ 14
Reflections collected	78548
Independent reflections	9068 [R_{int} = 0.0383, R_{sigma} = 0.0215]
Data/restraints/parameters	9068/0/382
Goodness-of-fit on F^2	1.452
Final R indexes [I>=2σ (I)]	R_1 = 0.0616, wR_2 = 0.1714
Final R indexes [all data]	R_1 = 0.0676, wR_2 = 0.1752
Largest diff. peak/hole / e Å^{-3}	1.29/-1.32

S4. Molecular Structure of $[PhC\{N(^tBu)\}_2Si\{N(Dipp)SiMe_3\}]$

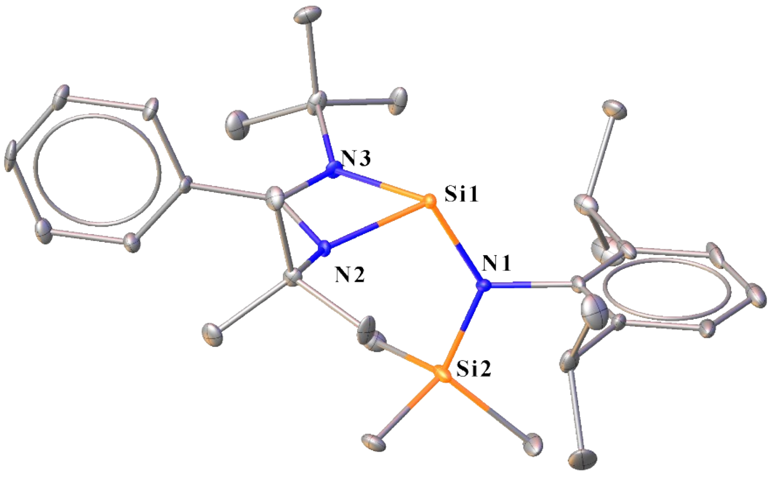

Figure S109. Molecular structure of $[PhC\{N(^tBu)\}_2Si\{N(Dipp)SiMe_3\}]$. Anisotropic displacement parameters are depicted at the 50% probability level. H atoms are omitted for clarity. Selected bond distances (Å) and angles (deg): Si1-N1 1.785(2), Si1-N3 1.918(2), Si1-N2 1.947(2), N1-Si2 1.763(1), N1-C46 1.465(2) and N3-Si1-N1 105.20(7), N3-Si1-N2 67.77(7), N2-Si1-N1 106.92(7).

- **Packing of complex 21 through various non-covalent interactions**

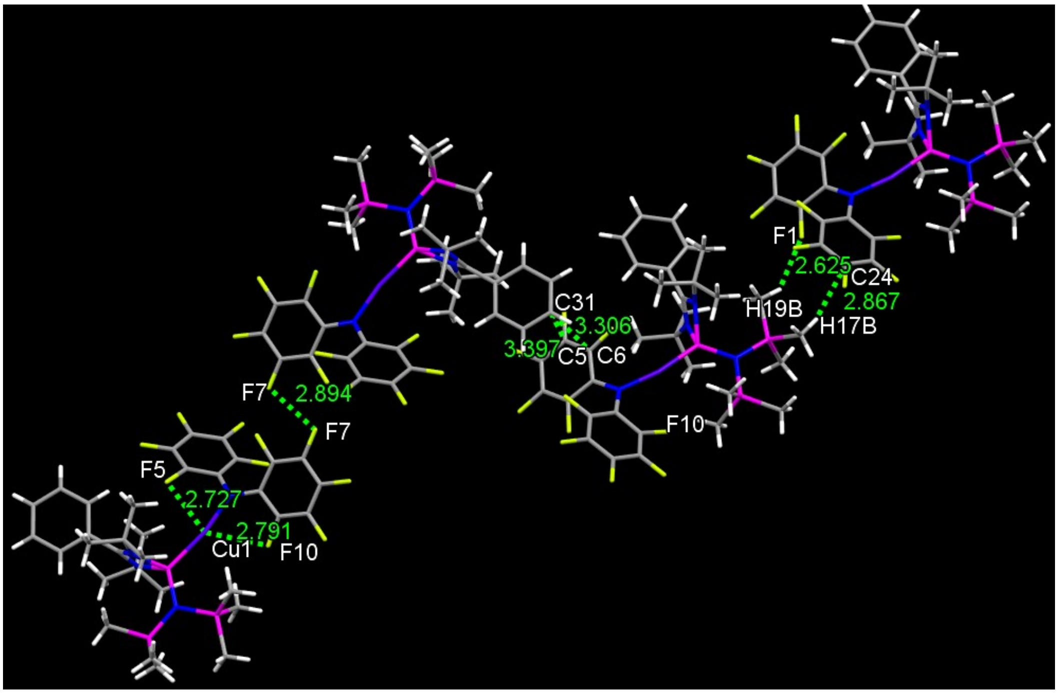

Figure S110. Cu···F interactions in complex **21**.

S5. Computational Section.

The bonding nature of complexes **2-4**, **6-8**, **12**, and **14-24** has been obtained by performing density functional theory (DFT) calculations using the Gaussian 09 program package.[23] Ground state geometries were optimised at the B3LYP-D3/def2-SVP level by taking the crystal structures as the input coordinates. These calculations were performed using an ultrafine numerical integration grid with tight convergence criteria. These optimised geometries were further optimised at the B3LYP-D3/def2-TZVPP level, and the theoretical data provided in the main text and SI are obtained at this DFT level. Harmonic frequencies were calculated to ensure the real local minima of these geometries. Frontier molecular orbital (FMO) analysis, such as relevant highest occupied molecular orbitals (HOMOs) and lowest unoccupied molecular orbitals (LUMOs) was performed to elucidate the bonding nature of various complexes. It puts forward a visual understanding of the key orbitals contributing to the stabilisation of each molecular complex. It provides information about the nature of bonding and the distribution of electron density present in a complex, such as the localisation of a particular orbital on a metal or ligand. Natural bond orbital (NBO) analysis was performed using the NBO7.0 program to determine second-order perturbative energies [$E^{(2)}$, kcal/mol] for various non-covalent interactions in these complexes.[24] The $E^{(2)}$ values are important to quantify the donor-acceptor interactions and to determine the strength of the non-covalent interactions present in various complexes. Non-covalent interactions, such as metallophilic interactions, hydrogen bonding, and other weak interactions discussed in this work, contribute to the stabilisation of these complexes either in the crystalline phase or the intermediate and transition states. Complex **14** was optimised using the integral equation formalism variant polarisable continuum model (IEFPCM) available in the Gaussian 09 program package, using toluene as a solvent to obtain information about its stability in solution.[25] Binding energy of this complex **14** was obtained by subtracting the ZPE corrected electronic energy of the monomers from the ZPE and basis set superposition error (BSSE) corrected electronic energy of the complex **14**.[26] Thermal correction to the binding energy of complex **14** was performed by calculating the relative change in the Gibbs free energy of complex **14** in the temperature range of 0-400 K.

Table S3. Relevant geometrical parameters of various complexes

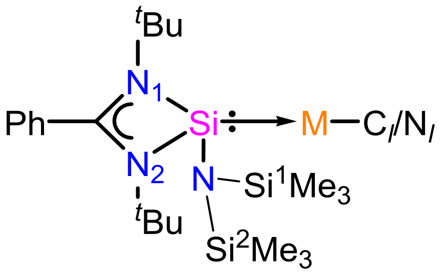

	2	3	4	6	7	8
**Si–M**	2.2583	2.2513	2.2522	2.8338	2.3892	2.3896
**M–C_{ipso}**	1.9578	1.9608	1.9629	2.2220	2.1406	2.1425
**Si–M–C_{ipso}**	171.39	172.00	172.16	178.13	169.71	169.72
**Si–N1**	1.8581	1.8597	1.8575	1.8435	1.8575	1.8572
**Si–N2**	1.8610	1.8575	1.8596	1.8469	1.8597	1.8597
**N1–Si–N2**	70.25	70.29	70.29	70.85	70.30	70.31
**N1–Si–M**	111.03	109.08	110.30	109.58	109.25	109.22
**N2–Si–M**	108.56	110.27	109.15	107.39	107.43	107.47
**Si–N_{TMS}**	1.7377	1.7366	1.7365	1.7311	1.7363	1.7361
**N_{TMS}–Si–N1**	112.24	113.38	112.70	113.77	112.74	112.73
**N_{TMS}–Si–N2**	113.03	112.73	113.36	114.16	113.02	113.04
**N_{TMS}–Si–M**	127.46	127.07	127.05	127.19	128.96	128.94
**N_{TMS}–Si1**	1.7808	1.7815	1.7602	1.7666	1.7625	1.7626
**N_{TMS}–Si2**	1.7598	1.7601	1.7815	1.7814	1.7827	1.7827
**M–M**	-	-	-	2.8195	-	-

	14		15		16	
	**unit 1**	**unit 2**	**unit 1**	**unit 2**	**unit 1**	**unit 2**
**Si–M**	2.2184	2.2233	2.2697	2.2697	2.3869	2.3869
**M–Se1**	2.4129	2.2692	2.5766	2.5765	2.8282	2.8282
**M–Se2**	2.3018	2.5804	2.4977	2.4977	2.6661	2.6661
**Si–M–Se1**	126.99	154.09	122.89	122.90	120.58	120.58
**Si–M–Se2**	131.65	108.78	144.44	144.44	146.28	146.28
**Si–N1**	1.8537	1.8575	1.8585	1.8586	1.8526	1.8526

**Si–N2**	1.8605	1.8571	1.8671	1.8671	1.8590	1.8590
**N1–Si–N2**	70.26	70.22	70.12	70.12	70.41	70.41
**N1–Si–M**	117.38	116.03	113.64	113.64	110.82	110.81
**N2–Si–M**	111.49	112.14	109.68	109.68	109.40	109.41
**M–M**	2.8874		3.5067		3.7875	

	**17**	**18**	**19**	**20**	**21**	**22**
**Si–M**	2.2196	2.2217	2.2073	2.2014	2.2237	2.3427
**M–N$_{amide}$**	1.8820	1.8951	1.9187	1.9031	1.9442	2.1704
**Si–M–N$_{amide}$**	171.04	168.95	168.12	174.18	165.23	165.38
**Si–N1**	1.8530	1.8477	1.8589	1.8521	1.8483	1.8492
**Si–N2**	1.8559	1.8564	1.8527	1.8533	1.8520	1.8464
**N1–Si–N2**	70.50	70.62	70.44	70.59	70.68	70.78
**N1–Si–M**	111.53	115.77	106.27	109.45	112.17	108.52
**N2–Si–M**	108.42	106.13	114.29	110.05	108.13	110.24
**Si–N$_{TMS}$**	1.7310	1.7323	1.7323	1.7279	1.7278	1.7252
**N$_{TMS}$–Si–N1**	112.99	112.93	113.57	114.64	113.08	114.06
**N$_{TMS}$–Si–N2**	113.92	114.03	112.87	114.11	113.92	114.03
**N$_{TMS}$–Si–M**	126.08	124.40	125.75	125.18	125.71	125.98
**N$_{TMS}$–Si1**	1.7840	1.7827	1.7851	1.7895	1.7858	1.7891
**N$_{TMS}$–Si2**	1.7630	1.7636	1.7606	1.7617	1.7646	1.7680
**F–M**	-	-	-	-	2.6907	2.8437
**F–M**	-	-	-	-	3.0693	2.9837
**N$_{Py}$–M**	-	-	2.6706	-	-	-
**N$_{formamidine}$–M**	-	-	-	2.7123	-	-

	**23**		**24**	
	**unit 1**	**unit 2**	**unit 1**	**unit 2**
**Si–M**	2.2708	2.2702	2.3892	2.3894
**M–P1**	2.4012	2.4013	2.5618	2.5746
**M–P2**	2.3974	2.3973	2.6096	2.5928

**Si–M–P1**	129.80	129.86	135.38	133.43
**Si–M–P2**	138.16	138.11	133.47	135.79
**Si–N1**	1.8618	1.8617	1.8566	1.8562
**Si–N2**	1.8649	1.8650	1.8626	1.8609
**N1–Si–N2**	70.08	70.08	70.29	70.32
**N1–Si–M**	115.11	115.15	113.77	113.37
**N2–Si–M**	105.63	105.51	105.20	105.53
**M–M**	3.3768		3.6995	

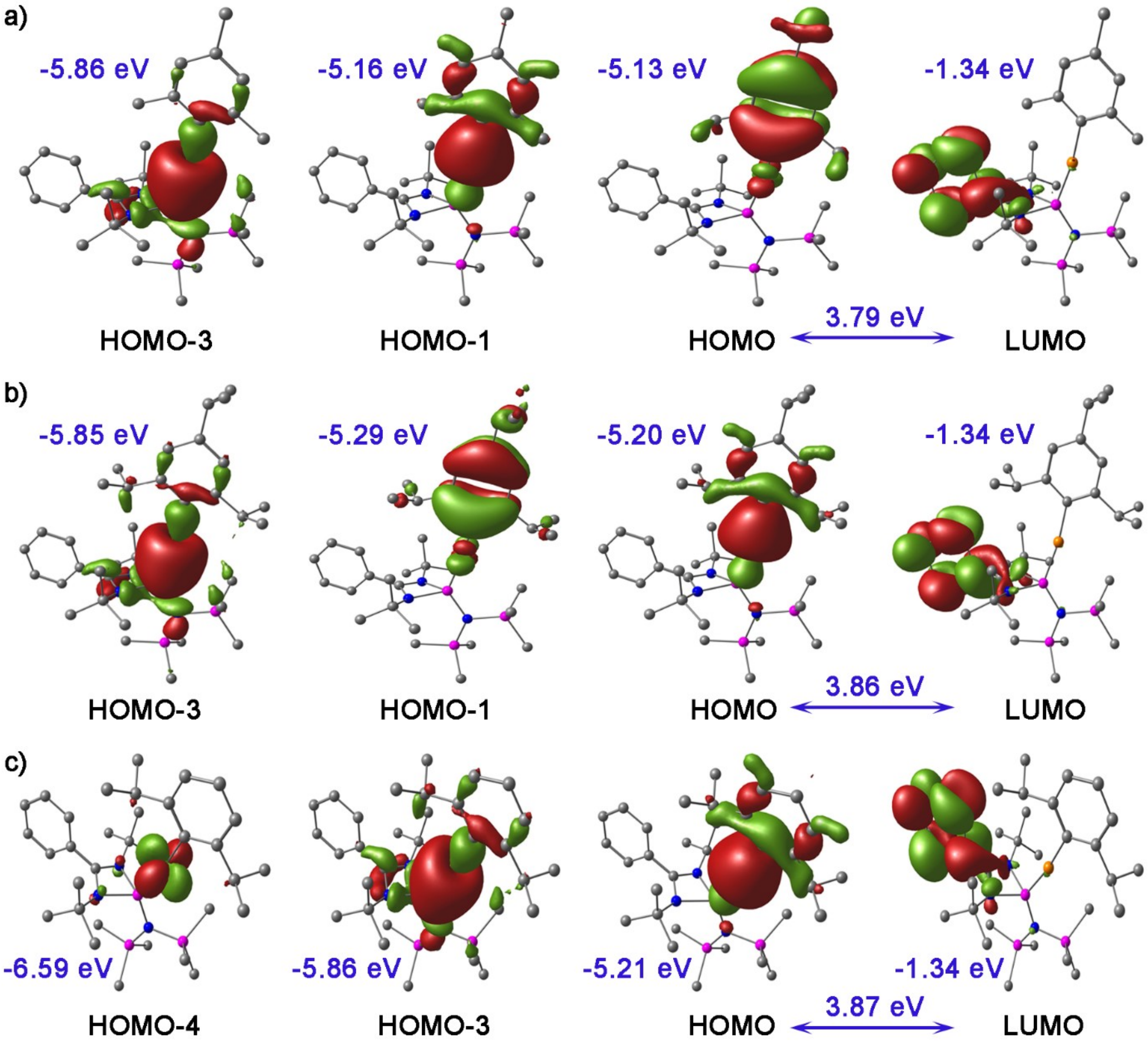

Figure S111. Molecular orbitals of complexes a) **2**, b) **3**, and c) **4** calculated at the B3LYP-D3/def2-TZVPP level.

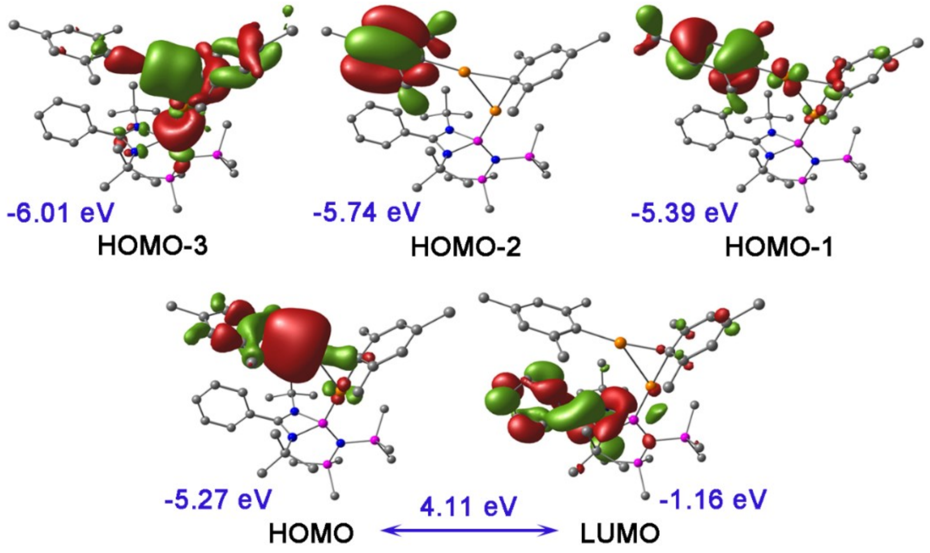

Figure S112. Molecular orbitals of complex **6**.

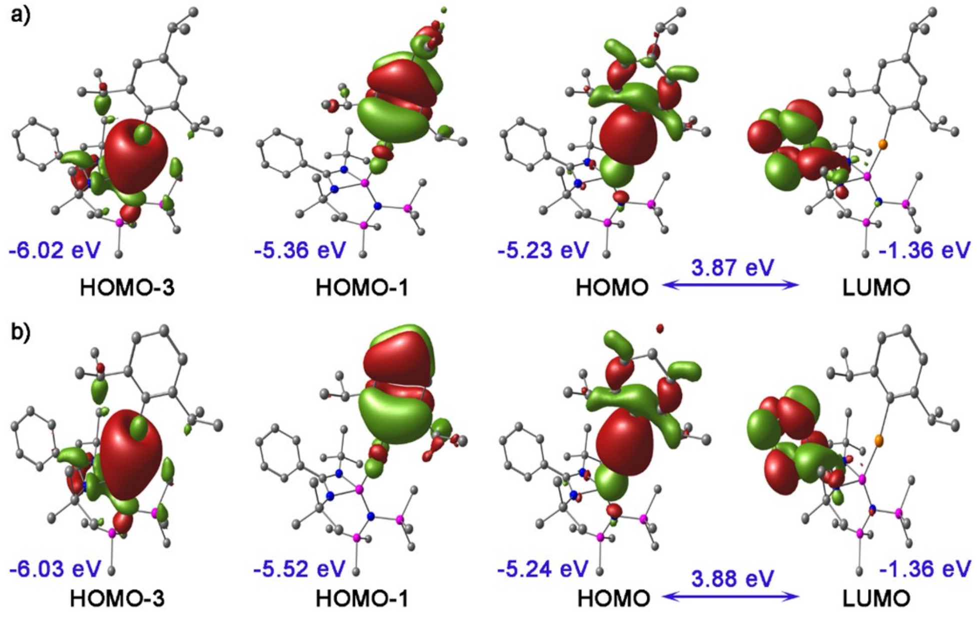

Figure S113. Molecular orbitals of complexes a) **7** and b) **8**.

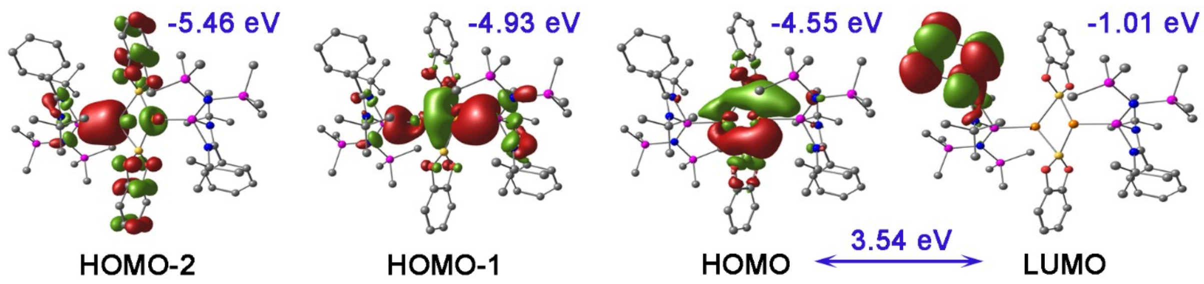

Figure S114. Molecular orbitals of complex **12**.

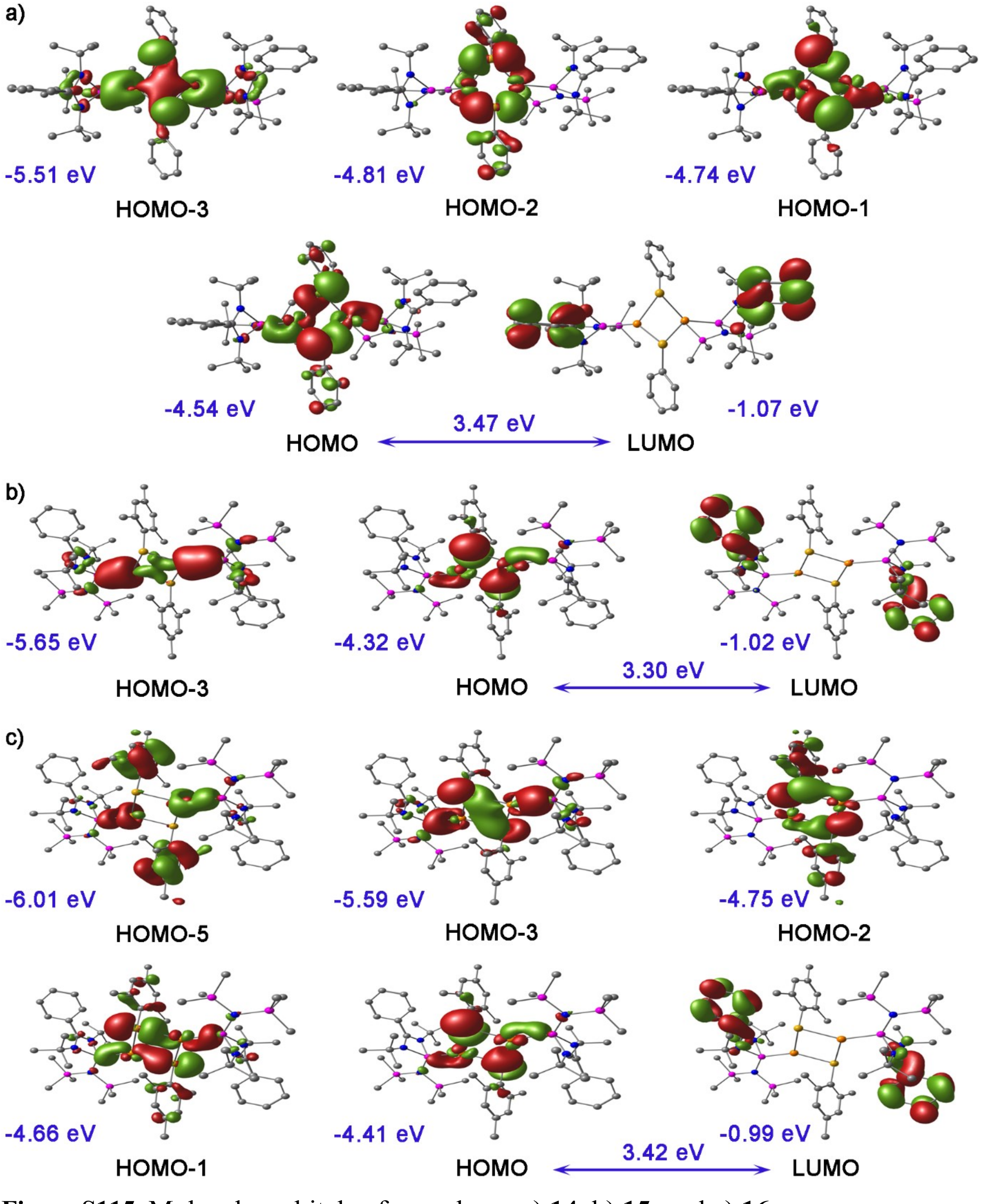

Figure S115. Molecular orbitals of complexes a) **14**, b) **15**, and c) **16**.

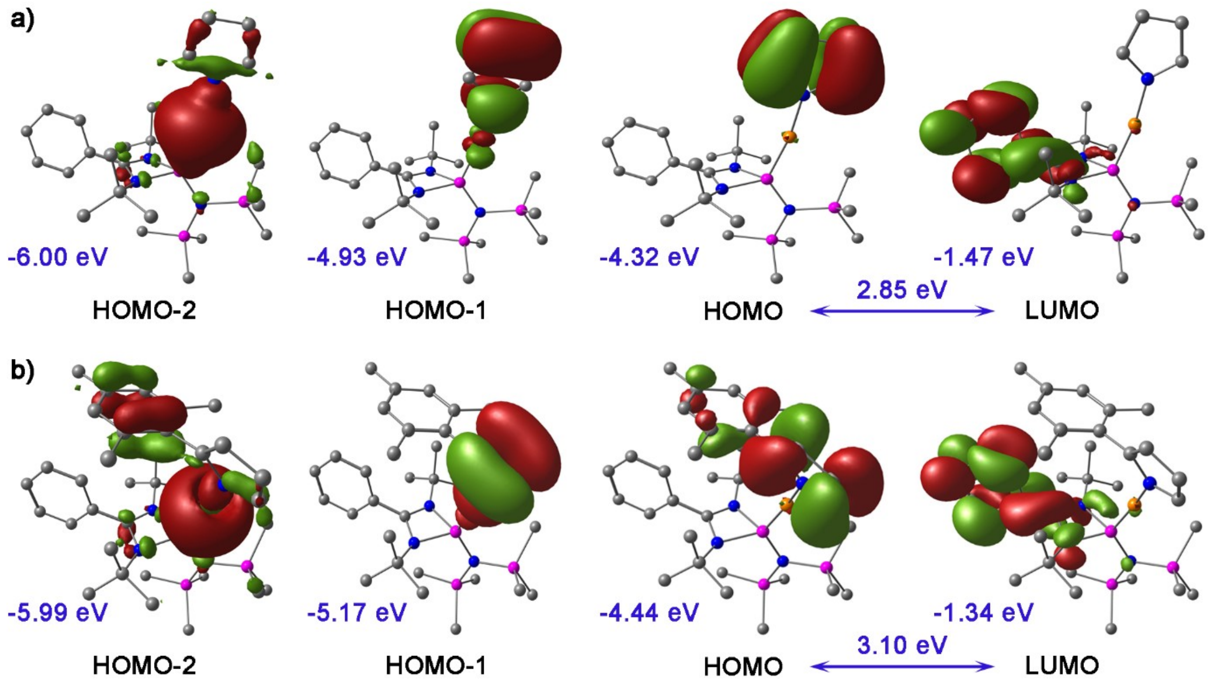

Figure S116. Molecular orbitals of complexes a) **17** and b) **18**.

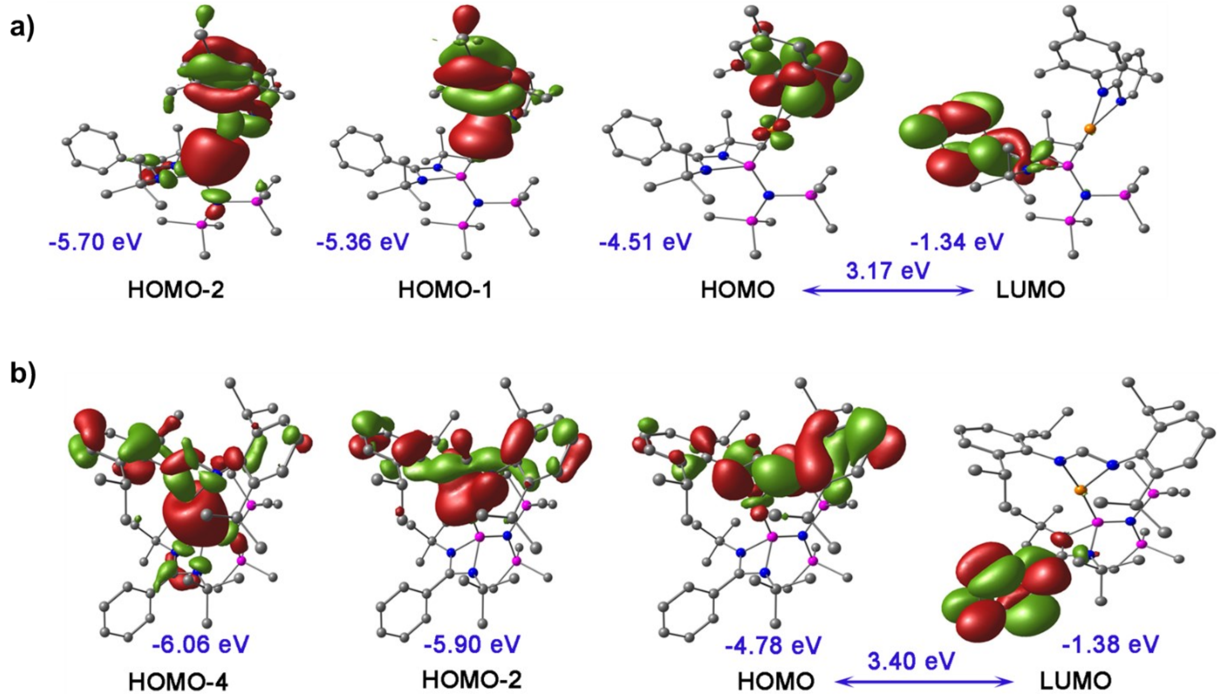

Figure S117. Molecular orbitals of complexes a) **19** and b) **20**.

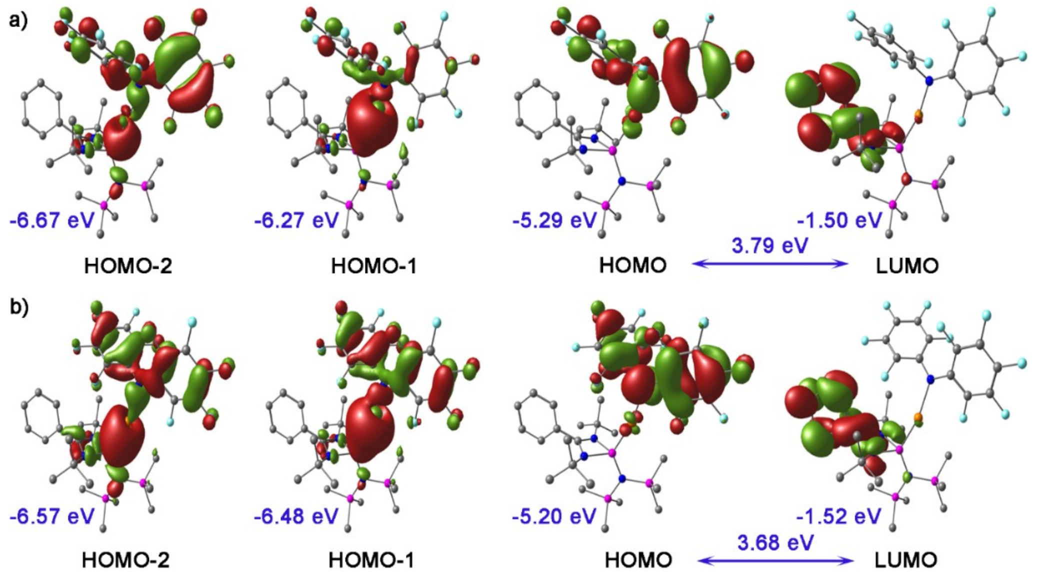

Figure S118. Molecular orbitals of complexes a) **21** and b) **22**.

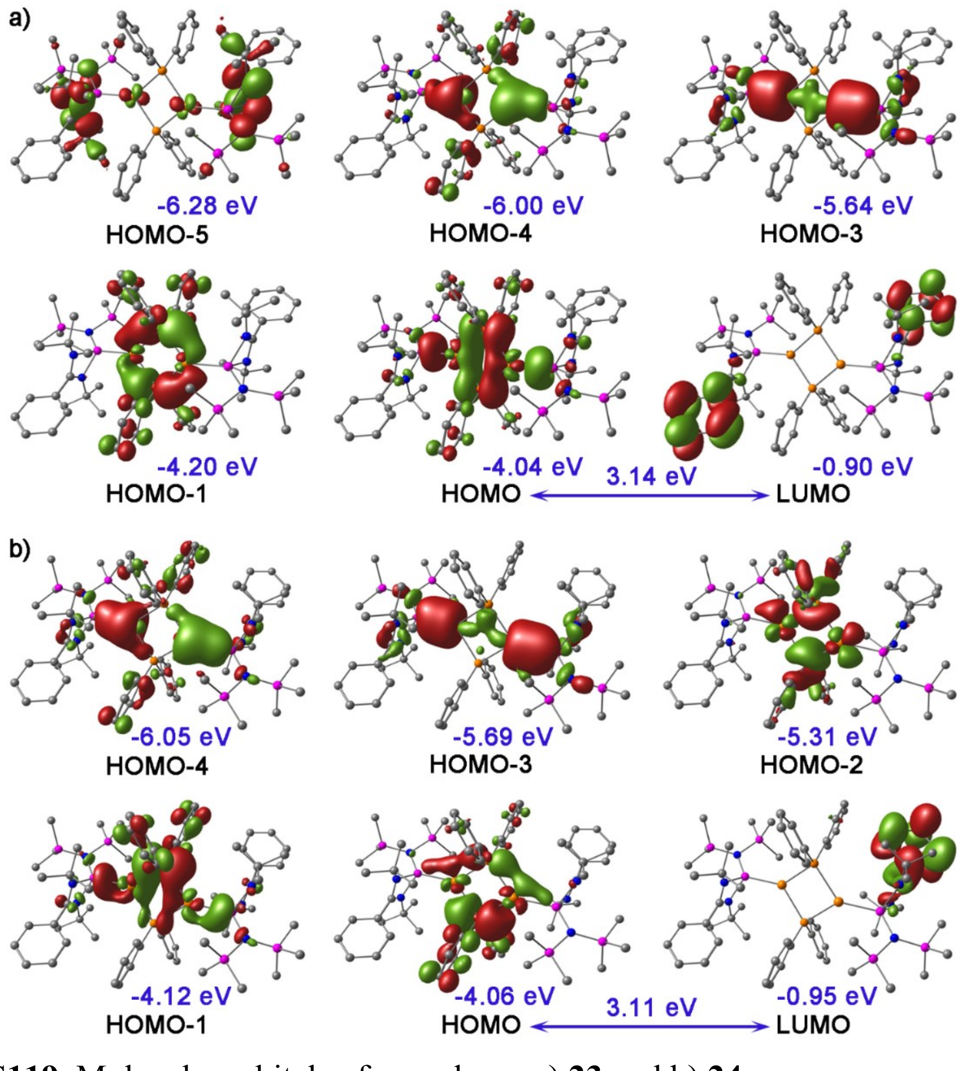

Figure S119. Molecular orbitals of complexes a) **23** and b) **24**.

- **NBO Analyses of complex 6, 14, 19-22**

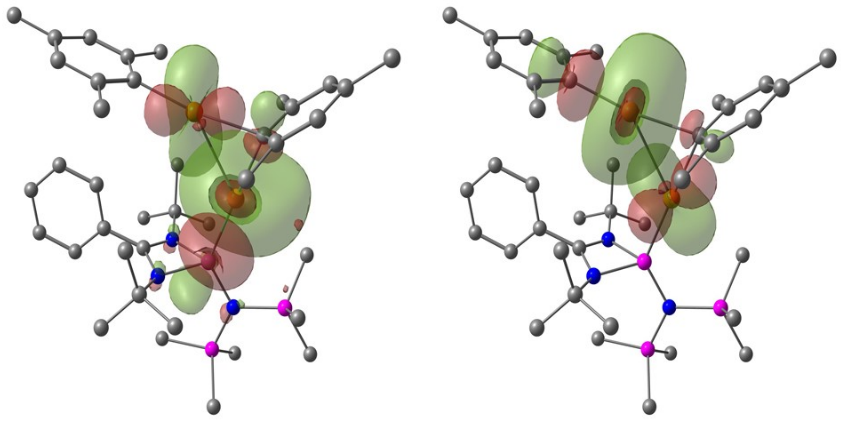

Figure S120. NBO images of **6**.

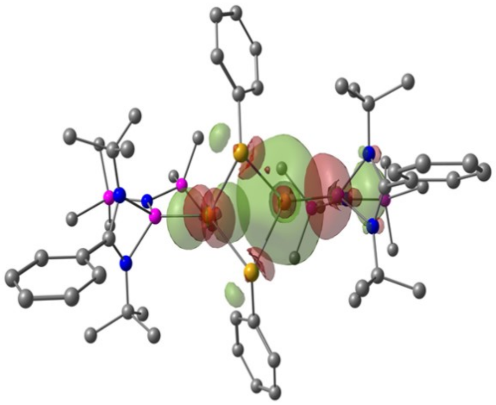

Figure S121. NBO orbital overlap with E$^{(2)}$ values for cuprophilic interaction in complex **14**.

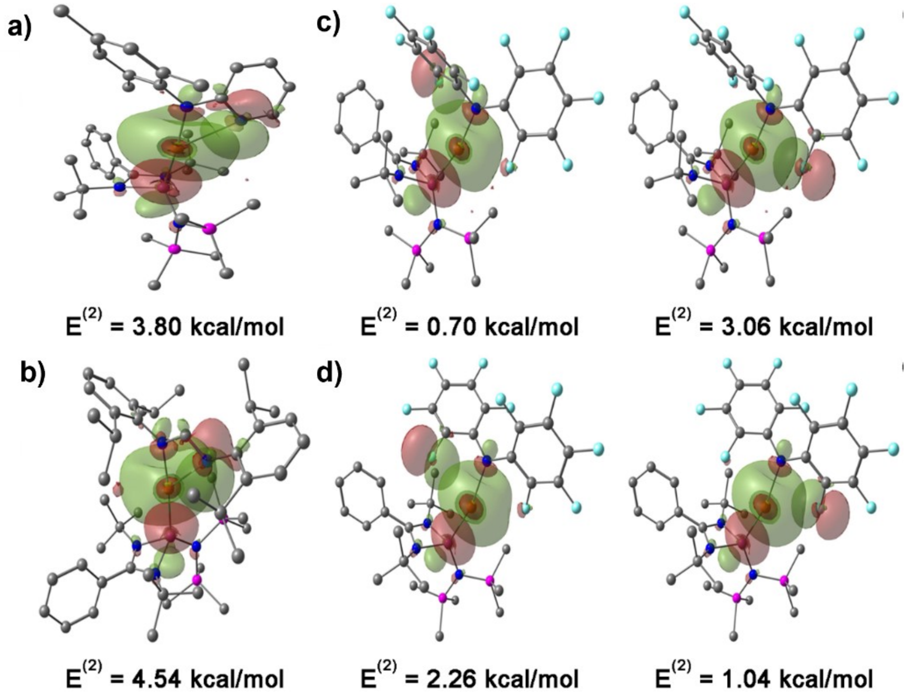

Figure S122. NBO images of a) and b) showing N→Cu interactions for complexes **19** and **20**; c) and d) Cu···F interaction in complexes **21** and **22**, respectively.

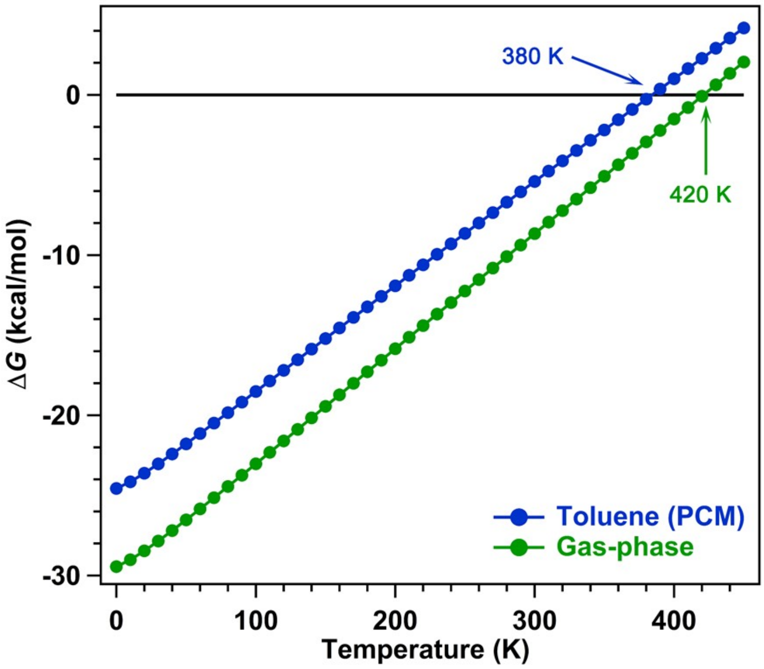

Figure S123. Plot of change in Gibbs free energy (Δ*G*, kcal/mol) of complex **14** in gas and solution-phase as a function of temperature (0-450 K).

❖ DFT studies on the mechanistic pathway

Ground state geometries of reactant and products (**P**), intermediates (**Int**), and transition states (**TS**) were optimised at the B3LYP-D3/def2-SVP level using IEFPCM for solvent correction with toluene as a solvent. Initial structures of transition states (first-order saddle points) involved in these chemical reactions were guessed with the help of chemical intuition. The single imaginary frequency along the bond-formation and bond-breaking reaction coordinate confirmed these as first-order saddle points. Furthermore, intrinsic reaction coordinate (IRC) calculations validate these transition states, showing connectivity between the reactants and products on either side of the potential energy surface.[27] The relative energies (kcal/mol) of the various species provided in the reaction profiles were corrected by zero-point energy (ZPE) corrections. The binding energies of the dimerised complexes were further corrected by the basis set superposition error (BSSE). The relative energies in the reaction profiles are calculated with respect to the sum of the energies of the two infinitely separated reactant molecules. The formation of the products in these chemical reactions passes through **Int** and **TS**, which are stabilised by the various non-covalent interactions. The analysis of these **Int** and **TS** was carried out with the help of relevant geometrical parameters obtained from the optimised geometries and NBO orbital overlaps with their second-order perturbative energies for various non-covalent interactions present therein, which provides a better understanding of their stability and favourable path for following these reaction profiles. **P*** is the association of two products before they get separated infinitely. The side products in **P*** are Mes–BCat, Mes–SePh, mesitylene, and Mes–$SiMe_3$, which are in close proximity to the complexes **12**, **14**, **17**, and **23**, respectively. The energy of the product (P) is calculated when these products and side products are infinitely separated from each other. Some of these products dimerise for further stabilisation to form μ-bridged dinuclear complexes, whose binding energies are calculated by subtracting twice the energy of the monomeric complex and further corrected by BSSE.

Complex 12: The two infinitely separated reactants come closer to form an intermediate (**Int**), stabilised by 12.23 kcal/mol energy. Several non-covalent interactions between the mesityl ring and the boron atom of B_2cat_2 stabilise this **Int**. The π orbital electron density of the mesityl ring delocalised into the vacant p_z orbital of boron atoms with $E^{(2)}$ values of 4.06 and 2.75 kcal/mol (NBO orbital overlaps are provided). This **Int** favours the twisting of B_2cat_2 in TS along the dihedral angle C–O–B–B (from -177.33° to -144.69°).

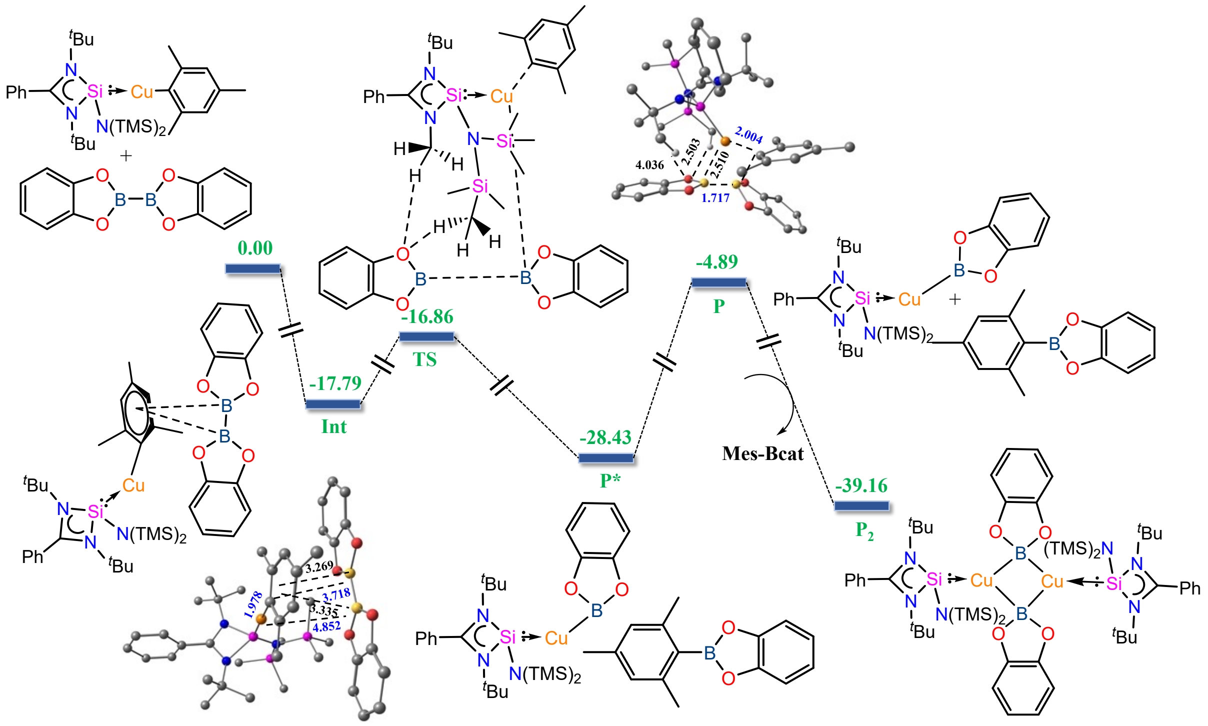

Figure S124. Relative energy (kcal/mol) reaction profile for the formation of complex **12** from complex **2** with BCat–BCat. Non-essential hydrogens are not shown for brevity.

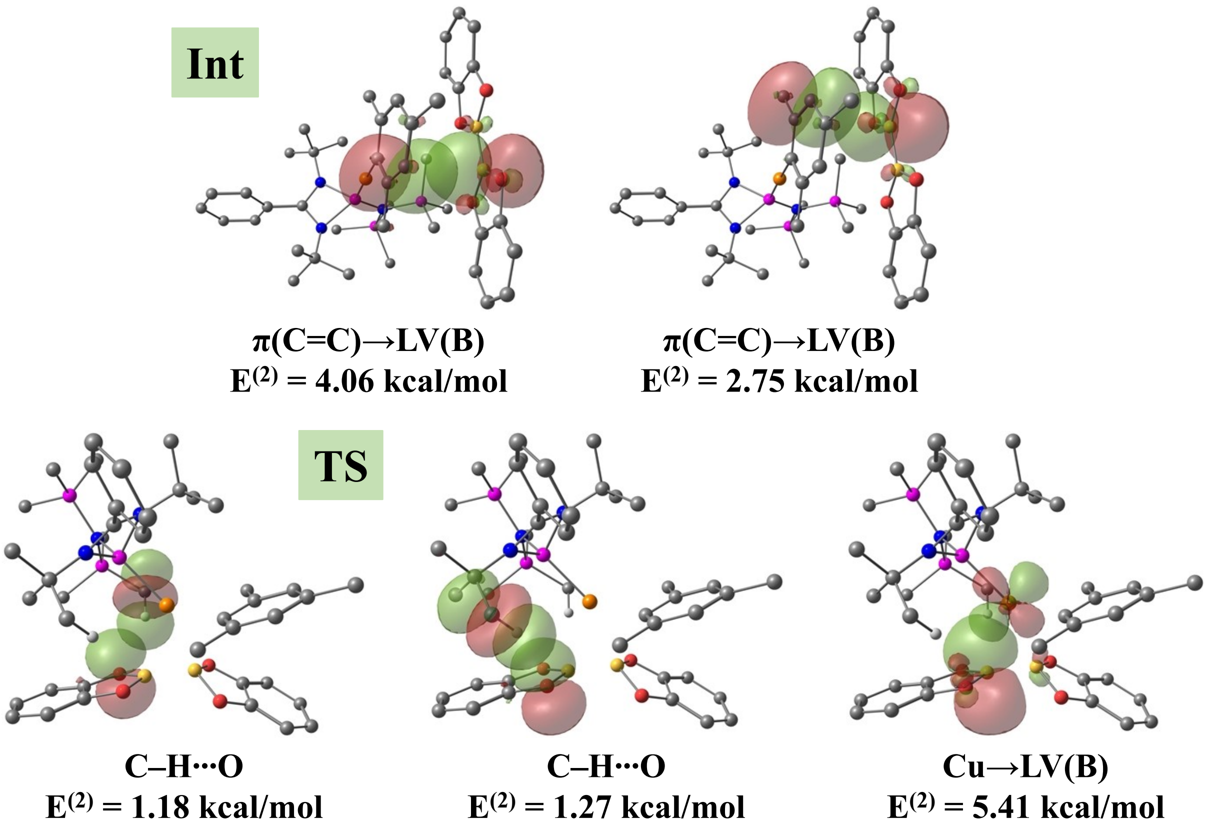

Figure S125. NBO orbital overlaps with $E^{(2)}$ values of various non-covalent interactions in **Int** and **TS** of the reaction between complex **2** with BCat–BCat.

Twisting of B_2cat_2 in **TS** shortens the Cu···B (from 4.852 to 2.510 Å) and elongates the Cu···C_{ipso} (1.978 to 2.004 Å) of the mesityl group. The imaginary frequency of 107 cm^{-1} along the reaction coordinate of B–C_{ipso} and Cu–B confirms the **TS** depicting the bond-breaking and bond-formation phenomenon. This **TS** is stabilised by the C–H···O hydrogen bond (2.45 kcal/mol) and the interaction between the copper and boron atoms. The electron density of the copper atom (d-orbital) is delocalised into the vacant p_z orbital of boron with an $E^{(2)}$ value of 5.41 kcal/mol. The IRC calculation provides the **P*** molecular complexes on the other side of the potential energy surface, followed by the infinitely separated monomeric product (**P**). The monomeric product further dimerises to give $\mathbf{P_2}$ with an additional stabilisation energy of 34.27 kcal/mol.[28]

<u>Complex 14</u>: Various non-covalent interactions, such as C–H···Se, C–H···π, tetrel bonding, and others, stabilise the **Int** observed in the reaction between complex **2** and PhSe–SePh. The lone-pair electron density of the Se atom delocalises into the vacant s orbital of the Cu atom with $E^{(2)}$ values of 32.98 kcal/mol, which increases further in the **TS** to 37.54 kcal/mol. Similarly, the delocalisation of Cu d orbital electron density into the σ* orbital of the Se–Se bond slightly increases from the **Int** to **TS** by 3.79 to 3.98 kcal/mol. A similar observation was also made by Sen and co-workers on the reactivity of T-shaped Ni radical towards Ph_2S_2.[29]

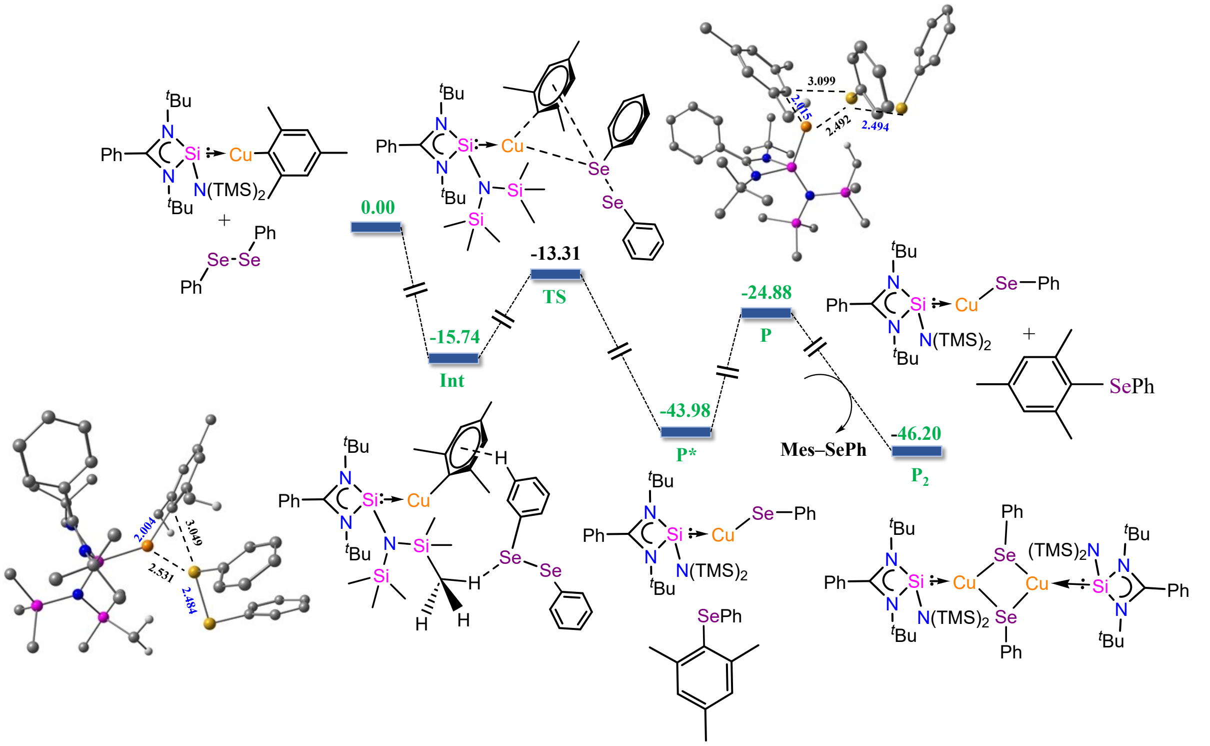

Figure S126. Relative energy (kcal/mol) reaction profile for the formation of complex **14** from complex **2** with PhSe–SePh. Non-essential hydrogens are not shown for brevity.

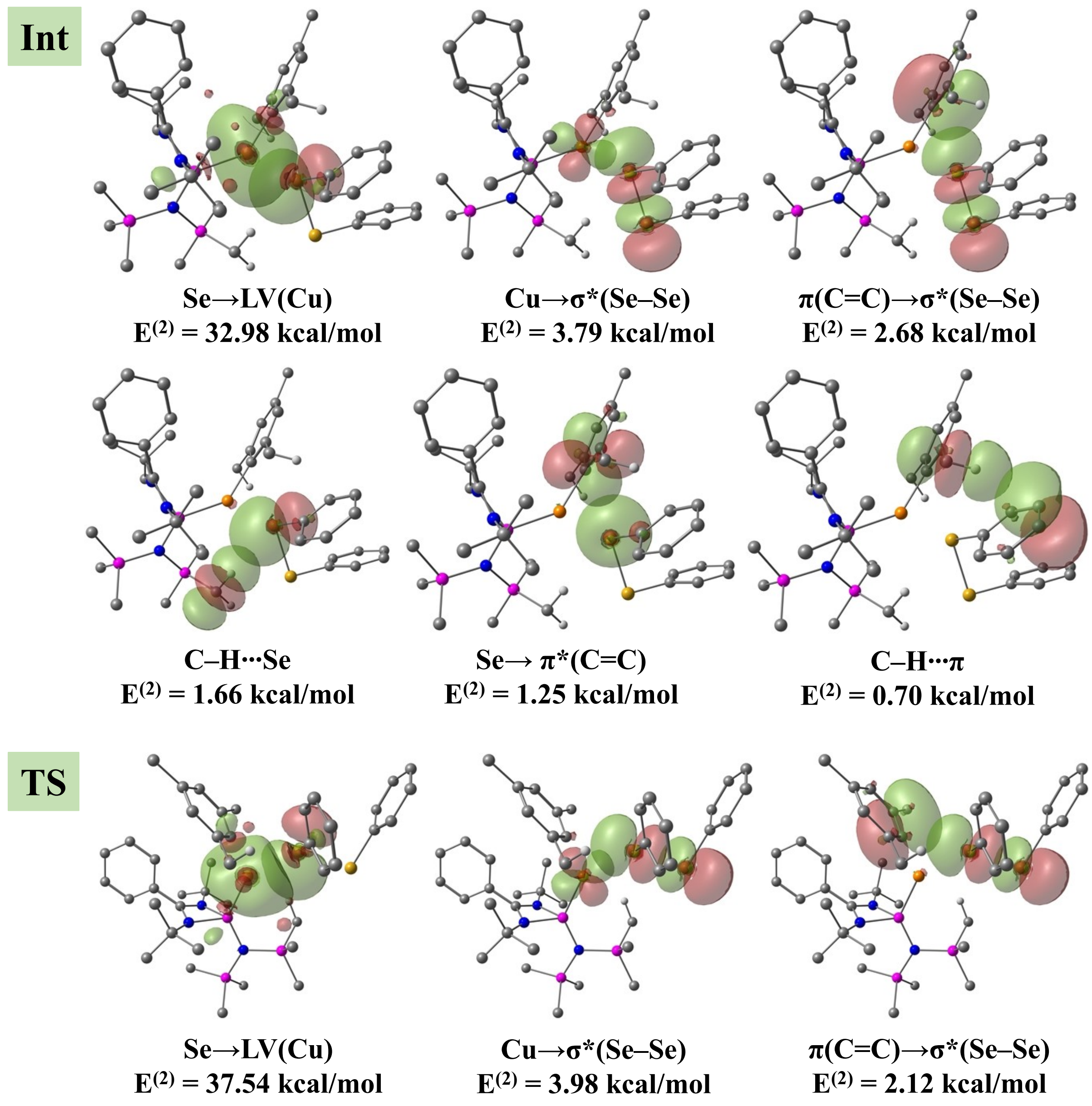

Figure S127. NBO orbital overlaps with $E^{(2)}$ values of various non-covalent interactions in Int and TS of the reaction between complex **2** with PhSe–SePh.

Interestingly, the delocalisation of π orbital electron density of the mesityl ring into the σ* orbital of the Se–Se bond slightly decreases from the **Int** to **TS** by 2.68 to 2.12 kcal/mol. This suggests the bond formation between Cu···Se in the **TS**. Moreover, the other non-covalent interactions, such as C–H···Se, C–H···π, and tetrel bonding, were also observed in Int with $E^{(2)}$ values of 1.66, 0.70, and 1.25 kcal/mol, respectively. The relevant geometrical parameters, such as slight elongation of the Cu–C_{ipso} bond (2.004 to 2.015 Å) of the mesityl group, shortening of Cu···Se distance (2.531 to 2.492 Å), and slight elongation of Se···Se bond distance (2.484 to 2.494 Å), confirm the bond-formation and bond-breaking phenomenon in **TS**. The

imaginary frequency of 8 cm^{-1} along the reaction coordinate of Se–C$_{ipso}$ and Cu–Se confirms the **TS** leading to the product formation. **P*** molecular complex having complex **14** and PhSe–Mes in close proximity was obtained on the potential energy surface *via* IRC calculation. Complex **14** undergoes dimerisation to form P$_2$ with a 21.32 kcal/mol stabilisation.

__Complex 17__: The reaction profile of a reaction between complex **2** and pyrrole passes *via* an **Int** stabilised by an N–H···π hydrogen bond of strength 3.38 kcal/mol with distance 2.239 Å from hydrogen to the C$_{ipso}$ of the mesityl group. This closure proximity of N–H group towards the mesityl ring favours the formation of the product *via* **TS**, where the hydrogen atom transfers from N–H to the C$_{ipso}$ of the mesityl group. The elongation of N–H bond (from 1.022 to 1.316 Å) in TS and shortening of the distance between the C$_{ipso}$ and the hydrogen atom (from 2.239 to 1.456 Å) suggest the hydrogen transfer in the **TS**. The elongation of Cu–C$_{ipso}$ and shortening of Cu–N distances from 1.978 to 2.157 Å and 3.206 to 2.434 Å, respectively, from intermediate to **TS**, suggests the favourable bond breaking and bond formation in **TS**.

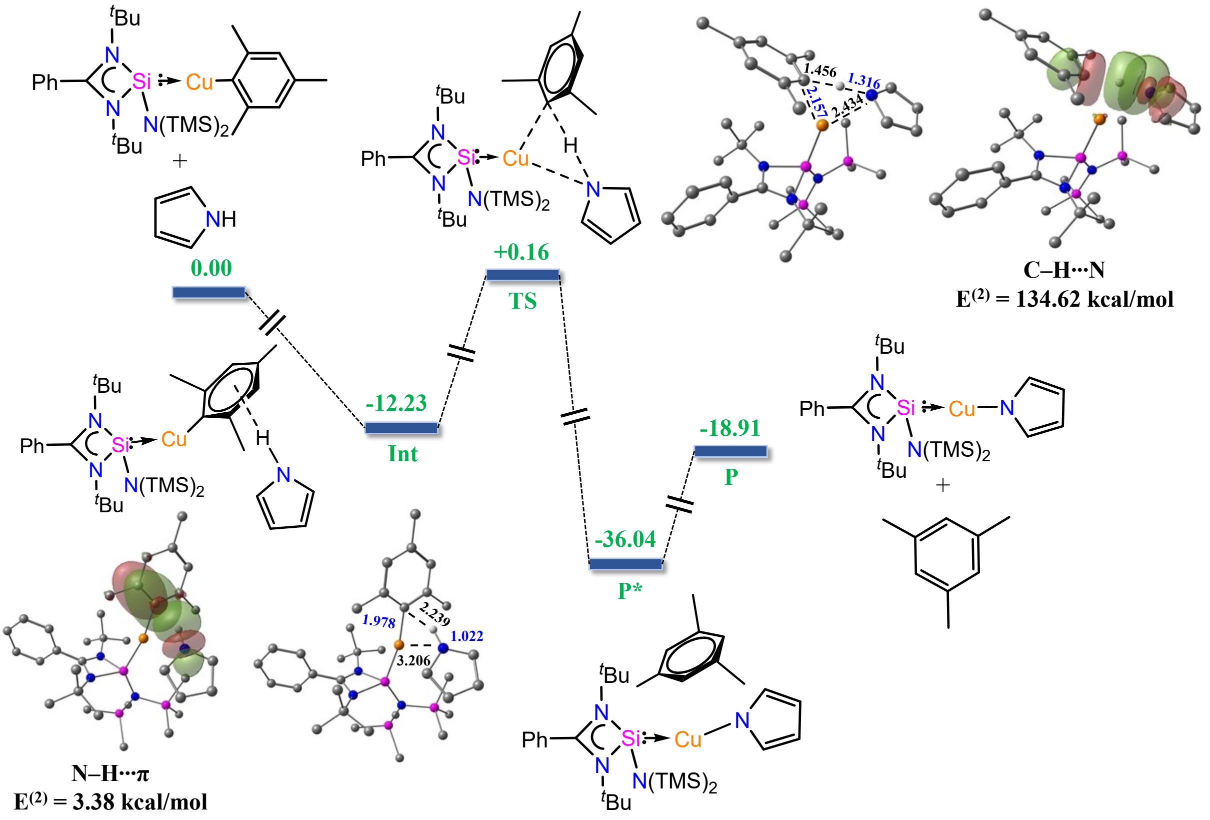

Figure S128. Relative energy (kcal/mol) reaction profile for the formation of complex **17** from complex **2** with pyrrole, with the NBO orbital overlap and E$^{(2)}$ energy for various non-covalent interactions in **Int** and TS. Non-essential hydrogens are not shown for brevity.

Imaginary frequency of 1373 cm^{-1} along the reaction coordinate of N–H–C$_{ipso}$ confirms the **TS,** showing bond-breaking and bond-formation phenomenon. Moreover, the enormously high E$^{(2)}$ energy of 134.62 kcal/mol for the C–H···N hydrogen bond in **TS** suggests the covalent nature of N–H and H–C$_{ipso}$ bond. This **TS** provides the combination of product molecules (**P***) where the side product mesitylene group is in close proximity to the complex **17**, which is further stabilised by -36.04 kcal mol^{-1} before proceeding to infinitely separated products.

<u>**Complex 23**:</u> Similar to the complex **17** reaction mechanism, π hydrogen bonding stabilises the Int observed in the reaction between complex **2** and SiMe$_3$–PPh$_2$.

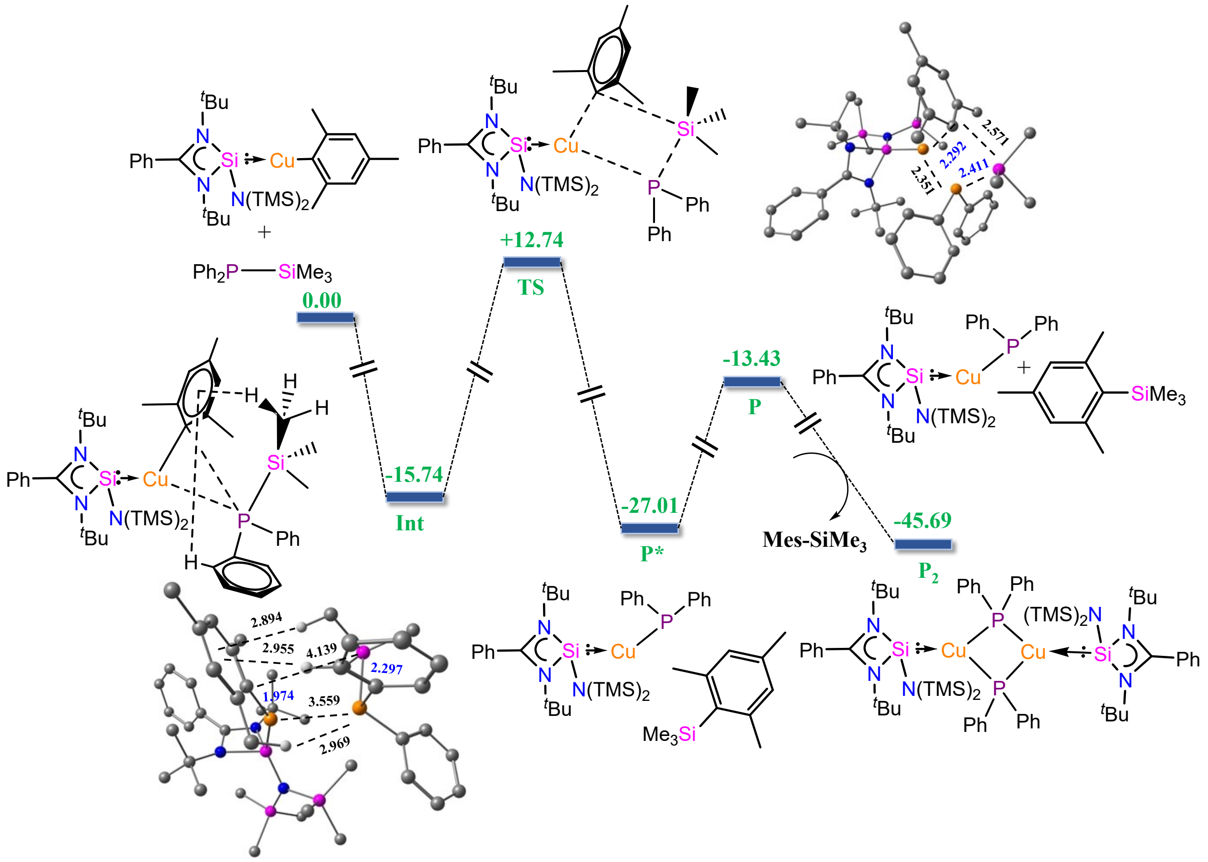

Figure S129. Relative energy (kcal/mol) reaction profile for the formation of complex **23** from complex **2** with SiMe$_3$–PPh$_2$. Non-essential hydrogens are not shown for brevity.

The π orbital electron density of the mesityl ring delocalised into the σ* orbital of C–H bond of the methyl group of SiMe$_3$ and the phenyl ring of the PPh$_2$ fragments with E$^{(2)}$ values of 2.16 and 1.76 kcal/mol (NBO orbital overlaps are provided), respectively. The lone-pair electron density of the phosphorus atom delocalises into the σ* orbital of C–H bond of the methyl group of mesitylene and the vacant s orbital of the Cu atom with E$^{(2)}$ values of 1.71 and 4.44 kcal/mol (NBO orbital overlaps are provided), respectively.

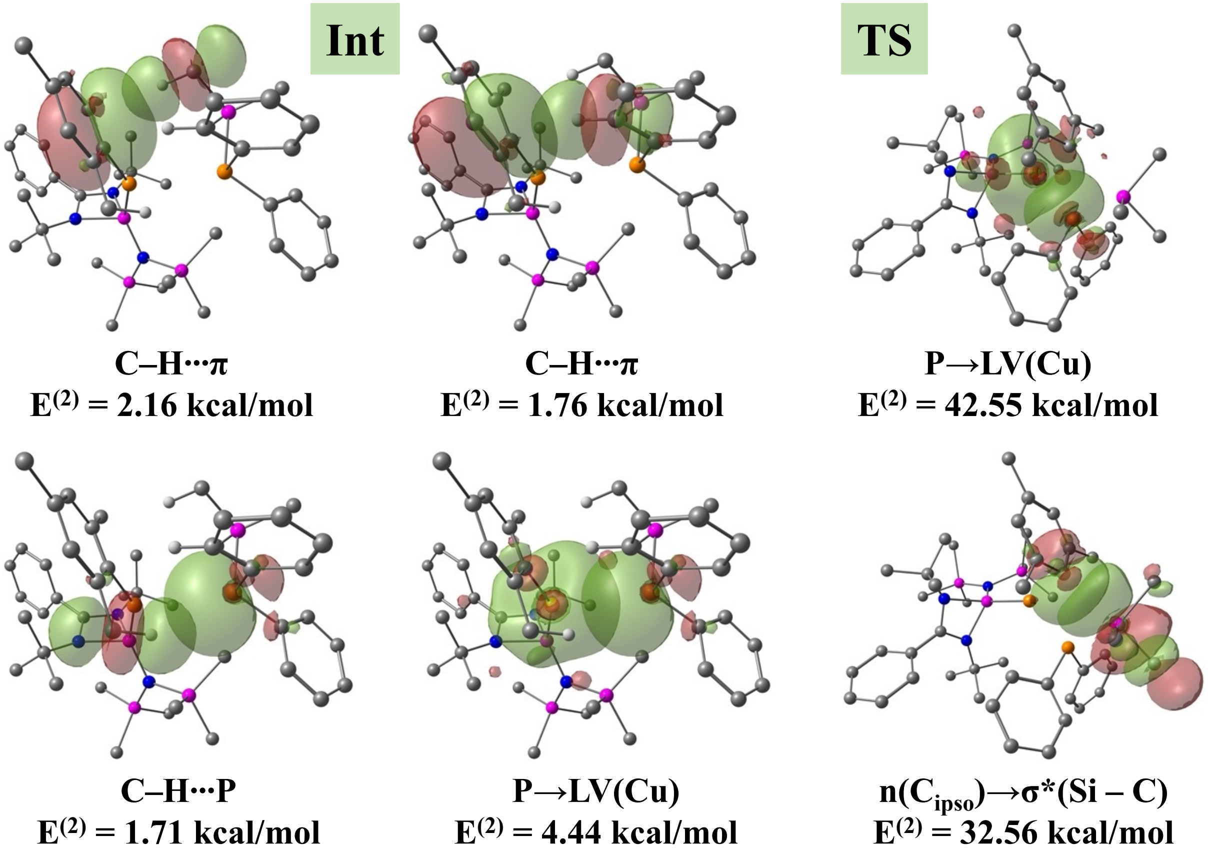

Figure S130. NBO orbital overlaps with $E^{(2)}$ values of various non-covalent interactions in **Int** and **TS** of the reaction between complex **2** with $SiMe_3$–PPh_2.

These bonding interactions bring the PPh_2 fragment closer to the Cu atom and favor the formation of TS. Interestingly, the interactions between P and Cu atoms increase to 42.55 kcal/mol in TS, which suggests the bond-formation of P···Cu. The non-bonding electron density of the C_{ipso} delocalises into the σ* orbital of the Si–C bond of the $SiMe_3$ group with $E^{(2)}$ values of 32.56 kcal/mol, suggesting that the mesitylene leaving group from complex **2** attacks the $SiMe_3$ group and forms a Si···C_{ipso} bond. The relevant geometrical parameters, such as elongation of the Cu–C_{ipso} bond (1.974 to 2.292 Å) of the mesityl group, elongation of P–Si bond (2.297 to 2.411 Å), shortening of Cu···P distance (3.559 to 2.351 Å), and shortening of Si···C_{ipso} distance (4.139 to 2.571 Å) confirms the bond-formation and bond-breaking phenomenon in **TS**. The imaginary frequency of 116 cm^{-1} along the reaction coordinate of Si–C_{ipso} and Cu–P confirms the **TS** leading to the product formation. **P*** molecular complex having complex **23** and $SiMe_3$–Mes in close proximity was obtained on the potential energy surface *via* IRC calculation. Complex **23** also undergoes dimerisation to form $\mathbf{P_2}$, similar to complex **14** (complex **12**) with a 32.26 kcal/mol stabilisation.

❖ **AIM analyses of the transition states:** Quantum theory of atoms in molecules (QTAIM) was performed at the B3LYP-D3/def2-SVP level for the transition state for various reaction mechanisms using Multiwfn3.8.[30] Bond critical points (BCPs) were observed for the new bond formation along with the bond breaking at these BCPs. Various parameters, such as ρ(r) (e $bohr^{-3}$), determining the electron density at the BCPs, and the second eigen value of Hessian matrix of electron density (λ_2) which determines the strength of the interaction and negative sign shows the attractive interaction whereas positive sign shows the repulsive nature of interaction. These parameters for the bond breaking BCPs were further compared with the Complex **2** (Cu–C_{ipso}), BCat–BCat (B–B), pyrrole (N–H), PhSe–SePh (Se–Se), and PPh_2–$SiMe_3$ (Si–P) to show the weakening of these bonds in the **TS**. AIM molecular graphs with the various electronic parameters at the BCPs in **TS** for the formation of Complexes **12**, **14**, **17**, and **23** are shown in Figures S131 and S132. In the transition states (**TS**) leading to the formation of complexes **12**, **14**, **17**, and **23**, BCPs were observed for the various bonds, specially for the Cu–C_{ipso}, B–B, and B–C_{ipso} in complex **12**; Cu–C_{ipso}, Se–Se, and Cu–Se in complex **14**; Cu–C_{ipso}, N–H, and H–C_{ipso} in complex **17**; Cu–C_{ipso}, P–Si, Si–C_{ipso}, and Cu–P in complex **23**. As shown in Figures S131 and S132, the weakening of the Cu–C_{ipso} was observed in all of these complexes in comparison to complex **2**. Moreover, the B–B bond in complex **12**, the Se–Se bond in complex **14**, the N–H bond in complex **17**, the P–Si bonds in complex **23** also shows noticeable weakening as compared to the other reagents, such as BCat–BCat, PhSe–SePh, pyrrole, and PPh_2–$SiMe_3$ reacting with the complex **2**. This weakening (bond-breaking) of these bonds is confirmed by the decrease in AIM parameters at the BCPs, such as electron density values and λ_2. AIM analysis also suggests the weak bond formation of B–C_{ipso} in complex **12**, the Cu–Se in complex **14**, the H–C_{ipso} in complex **17**, the Cu–P and Si–C_{ipso} in complex **23**.

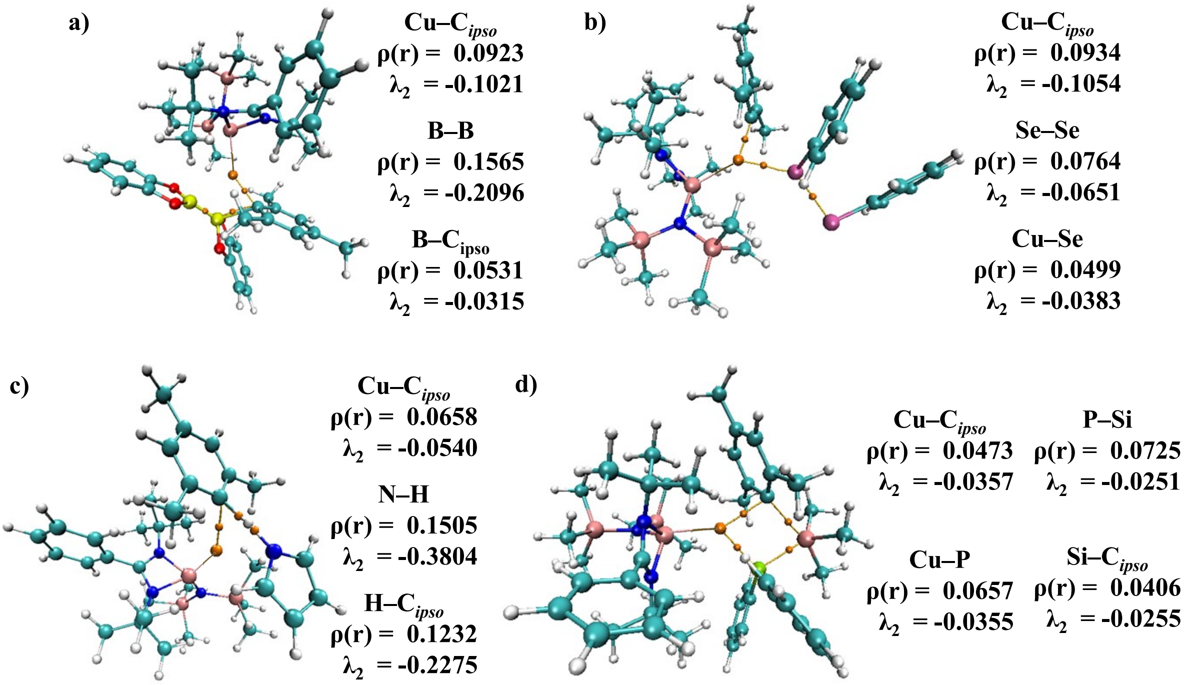

Figure S131. AIM molecular graphs with electronic parameters at various relevant BCPs in the **TS** leading to the formation of complexes a) **12**, b) **14**, c) **17**, and d) **23**.

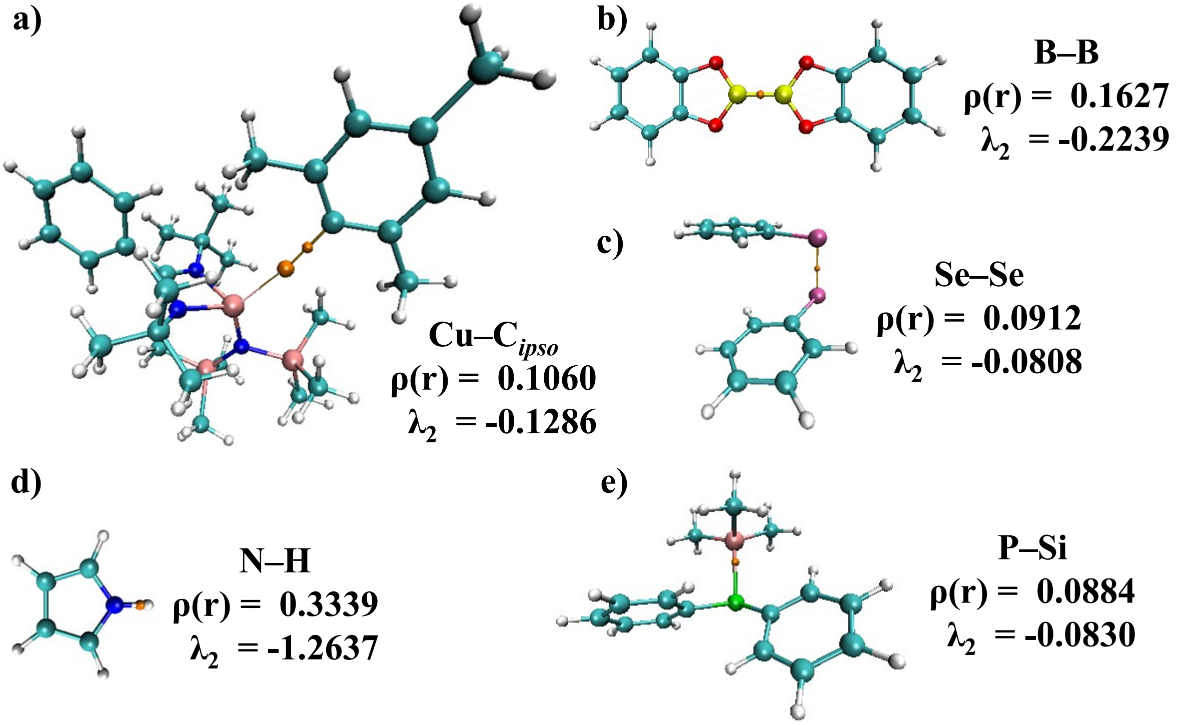

Figure S132. AIM molecular graphs with electronic parameters at various relevant BCPs in a) complex **2**, b) BCat–BCat, c) PhSe–SePh, d) pyrrole, and e) PPh_2–$SiMe_3$.

- Bond dissociation energies are calculated by subtracting the energy of a molecule from the sum of the energies of its infinitely separated fragmented radical products.

Table S4. Calculation of bond dissociation energy values

	E(A–B) in hr	E(A°) in hr	E(B°) in hr	BDE (kJ/mol)= [E(B°)+ E(A°)- E(A–B)]*2625.5
BCat–BCat (B–B)	-813.026671	-406.425188	-406.425188	462.86
PhSe–SePh (Se–Se)	-5266.503068	-2633.205928	-2633.205928	239.48
Pyrrole (N–H)	-210.177443	-209.527734	-0.5021542	387.41
PPh_2–$SiMe_3$ (P–Si)	-1213.951726	-804.660496	-409.190837	263.58

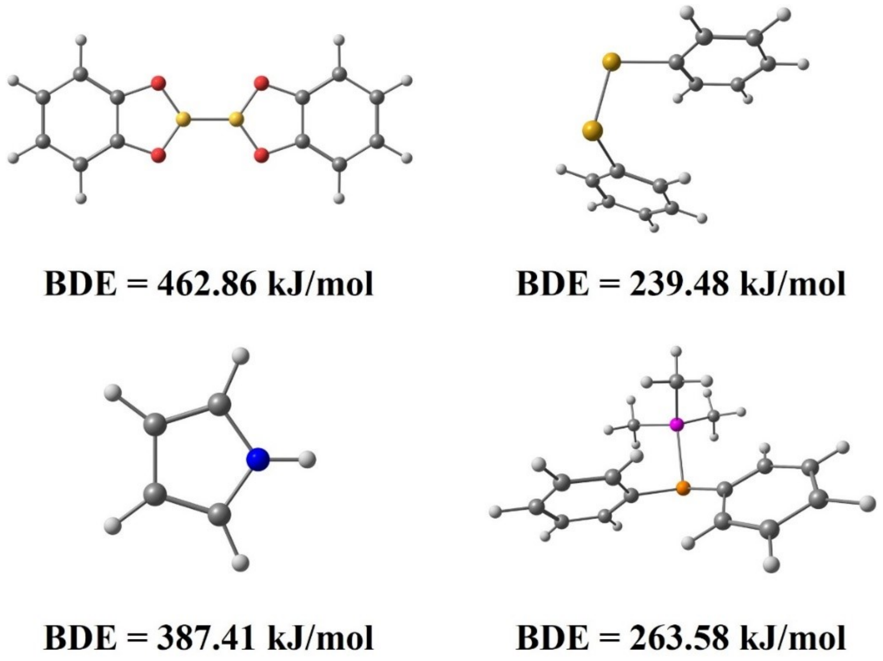

Figure S133. The bond dissociation energies of the B–B (B_2cat_2), Se–Se (Ph_2Se_2), N–H (Pyrrole), and P–Si (PPh_2–$SiMe_3$) bonds calculated at the B3LYP-D3/def2-TZVPP level.

S6. Metrical Parameters of Isolated Complexes

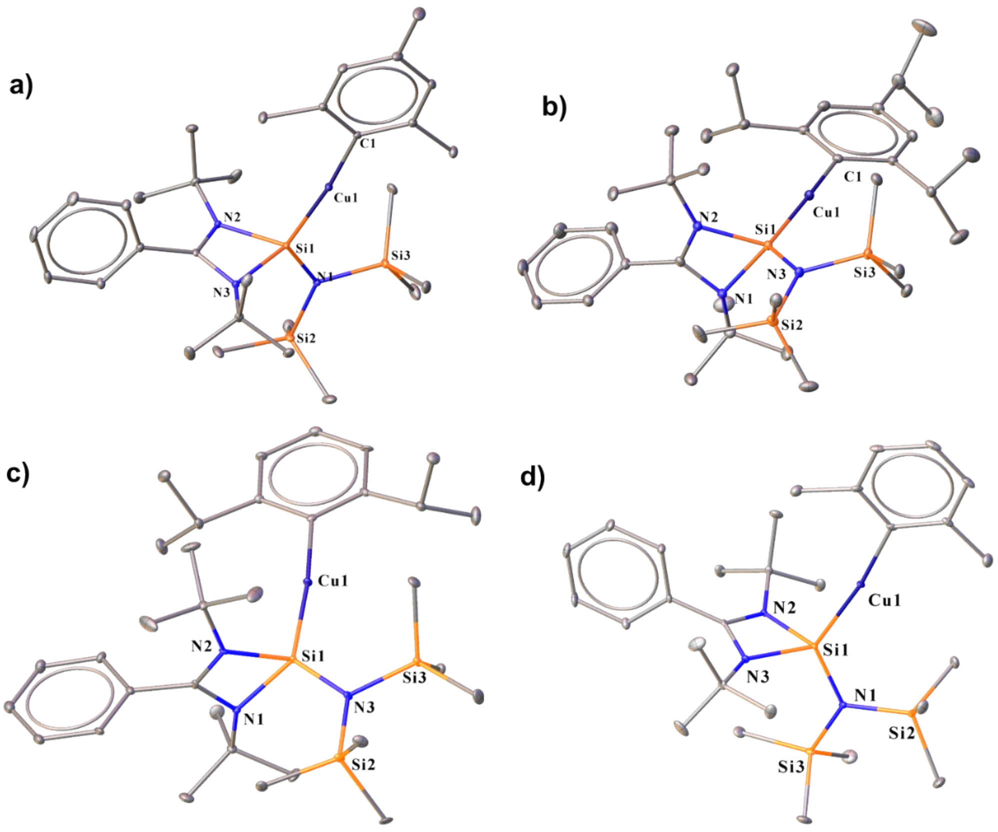

Figure S134. Molecular structure of complexes a) **2,** b) **3**, c) **4**, d) **5**. Anisotropic displacement parameters are depicted at the 50% probability level. Solvent moiety and H atoms are omitted for clarity. Selected bond distances (Å) and angles (deg): for **a)** Si1–Cu1 2.2509(8), Cu1–C1 1.946(2), Si1–N1 1.735(2), N1–Si2 1.758(2), N1–Si3 1.768(2), Si1–N2 1.845(2), Si1–N3 1.852(2) and Si1–Cu1–C1 173.28(6), N1–Si1–Cu1 124.91(6), N1–Si1–N2 110.71(8), N2–Si1–N3 70.49(7), N3–Si1–Cu1 109.99(5), for **b)** Si1–Cu1 2.2407(8), Cu1–C1 1.937(2), N1–Si1 1.861(2), N2–Si1 1.847(2), N3–Si1 1.736(2), N3–Si3 1.768(2), N3–Si2 1.753(2) and Si1–Cu1–C1 170.72(7), N3–Si1–N1 113.42(9), N3–Si1–Cu1 124.77(7), N1–Si1–Cu1 108.56(7), for **c)** Si1-Cu1 2.246(2), Cu1-C1 1.943(4), Si1-N2 1.851(4), Si1-N3 1.849(3), Si1-N1 1.735(3) and Si1-Cu1-C1 172.7(1), Cu1-Si1-N1 125.1(1), Cu1-Si1-N2 111.9(1), N3-Si1-N2 70.6(1), N3-Si1-N1 113.2(1); for **d)** Si1-Cu1 2.243(1), Cu1-C8 1.946(3), Si1-N1 1.737(3), N1-Si2 1.759(3), N1-Si3 1.758(3), Si1-N2 1.847(2), Si1-N3 1.844(2) and Si1-Cu1-C8 172.5(1), Cu1-Si1-N1 124.22(9), Cu1-Si1-N3 113.27(8), N2-Si1-N3 70.8(1), N2-Si1-N1 113.8(1).

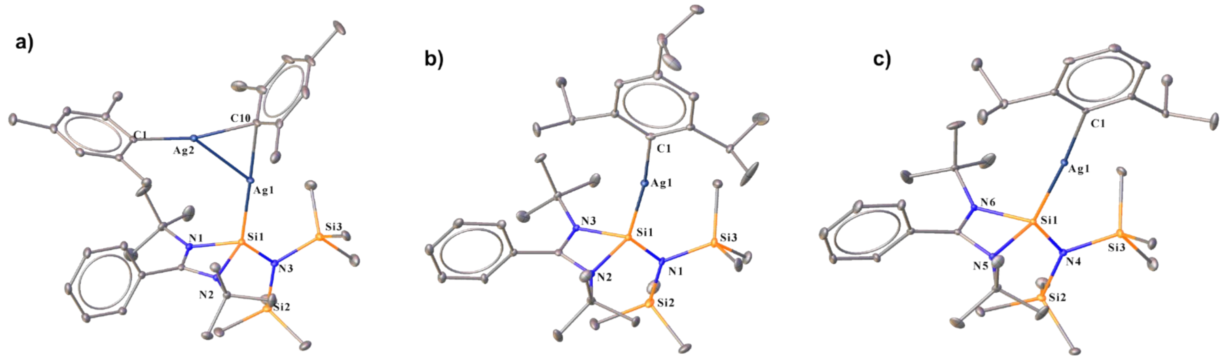

Figure S135. Molecular structure of complexes a) **6,** b) **7**, c) **8**. Anisotropic displacement parameters are depicted at the 50% probability level. Solvent moiety and H atoms are omitted for clarity. Selected bond distances (Å) and angles (deg): for (a) Si1-Cu1 2.246(2), Cu1-C1 1.943(4), Si1-N2 1.851(4), Si1-N3 1.849(3), Si1-N1 1.735(3) and Si1-Cu1-C1 172.7(1), Cu1-Si1-N1 125.1(1), Cu1-Si1-N2 111.9(1), N3-Si1-N2 70.6(1), N3-Si1-N1 113.2(1); Si1–Ag1 2.3900(9), Ag1–Ag2 2.7453(5), Ag1–C10 2.215(3), C1–Ag2 2.106(3), C10–Ag2 2.211(3), N1–Si1 1.843(2), N2–Si1 1.844(2), Si1–N3 1.719(2), N3–Si2 1.770(2), N3–Si3 1.768(2) and Si1–Ag1-C10 178.85(7), Si1–Ag1–Ag2 127.28(2), Ag2–Ag1–C10 51.60(7), C10–Ag2–Ag1 51.74(7), N2–Si1–N3 113.9(1), N1–Si1–N3 113.9(1), Ag1–Si1–N2 109.64(7), N3–Si1–Ag1 126.42(8), N1–Si1–N2 70.53(9). for **b)** Si1-Ag1 2.400(1), Ag1-C1 2.139(2), Si1-N3 1.841(2), Si1-N2 1.854(2), Si1-N1 1.733(2) and Si1-Ag1-C1 172.21(6), N3-Si1-N2 70.67(8), Ag1-Si1-N3 110.59(6), Ag1-Si1-N1 128.26(7), N2-Si1-N1 113.83(9), for **c)** Si1-Ag1 2.3873(6), Ag1-C1 2.132(2), Si1-N2 1.842(1), Si1-N3 1.849(2), Si1-N1 1.734(1) and Si1-Ag1-C1 173.44(5), N2-Si1-N3 70.82(7), Ag1-Si1-N2 110.50(5), Ag1-Si1-N1 125.59(6), N1-Si1-N3 112.78(7).

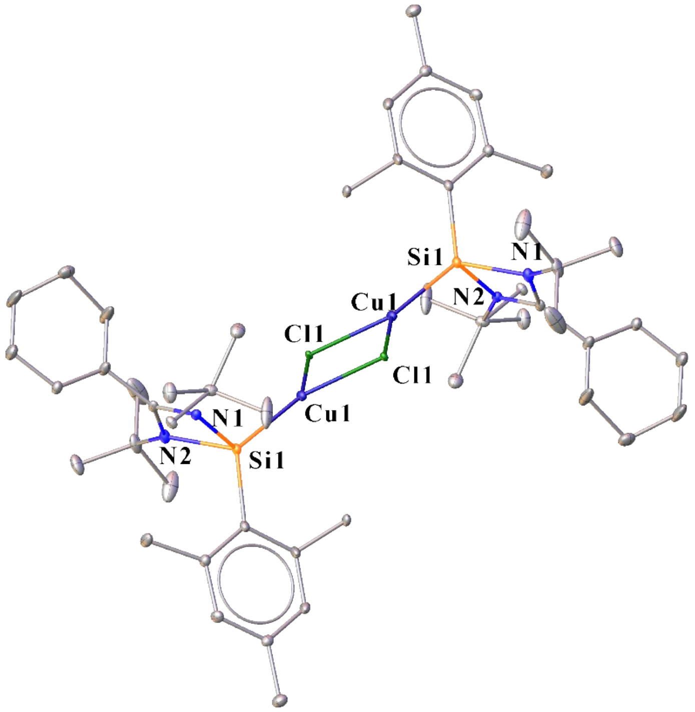

Figure S136. Molecular structure of **9**. Anisotropic displacement parameters are depicted at the 50% probability level. Disordered C atoms of *tert*-butyl, mesityl, phenyl moieties, and H atoms are omitted for clarity, and only the asymmetric unit is shown. Selected bond distances (Å) and angles (deg): Si1–Cu1 2.213(1), Cu1–Cl1 2.345(1), Si1–C16 1.899(5), Si1–N2 1.835(4), Si1–N1 1.842(4) and Si1–Cu1–Cl1 132.14(5), N2–Si1–N1 70.6(2), N2–Si1–Cu1 115.4(1), N1–Si1–C16 110.1(2), C16–Si1–Cu1 125.0(2).

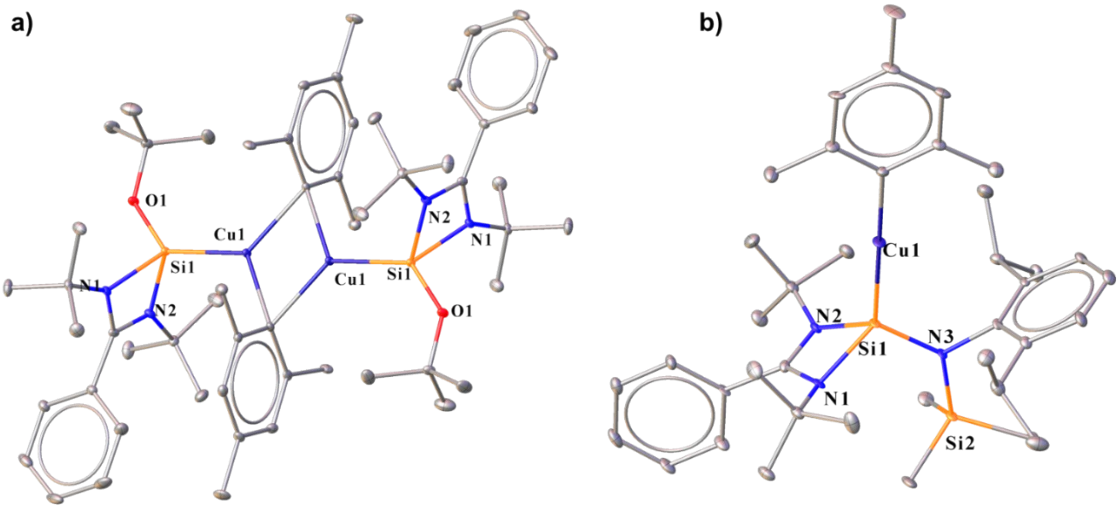

Figure S137. Molecular structure of complexes a) **10** and b) **11**. Anisotropic displacement parameters are depicted at the 50% probability level. Solvent moiety and H atoms are omitted for clarity. Selected bond distances (Å) and angles (deg): for (a) Si1-Cu1 2.249(1), Si1-Cu1 2.249(1), Cu1-Cu1 2.3356(7), Cu1-C1 2.170(4), C1-Cu1

2.132(4), Cu1-C1 2.132(4), Si1-O1 1.645(3), N1-Si1 1.858(4), Si1-N2 1.861(3) and Si1-Cu1-C1 119.8(1), C1-Cu1-C1 114.2(1), C1-Cu1-Si1 126.0(1), Cu1-C1-Cu1 65.8(1), Cu1-Si1-O1 131.7(1), Cu1-Si1-N1 117.1(1), N1-Si1-N2 70.0(2), O1-Si1-N2 102.7(2) and b) Si1-Cu1 2.2272(9), Cu1-C16 1.934(3), N2-Si1 1.888(2), Si1-N3 1.882(2), Si1-N1 1.747(2), N1-Si2 1.767(2) and Si1-Cu1-C16 179.68(9), Cu1-Si1-N2 118.23(7), Cu1-Si1-N1 118.11(7), N3-Si1-N1 109.8(1), N2-Si1-N3 69.80(9).

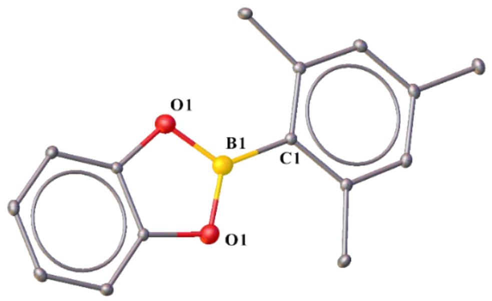

Figure S138. Molecular structure of **13**. Anisotropic displacement parameters are depicted at the 50% probability level. H atoms are omitted for clarity. Selected bond distances (Å) and angles (deg): O1-B1 1.398(1), B1-C1 1.553(3), and C1-B1-O1 125.0(1), O1-B1-O1 110.0(2).

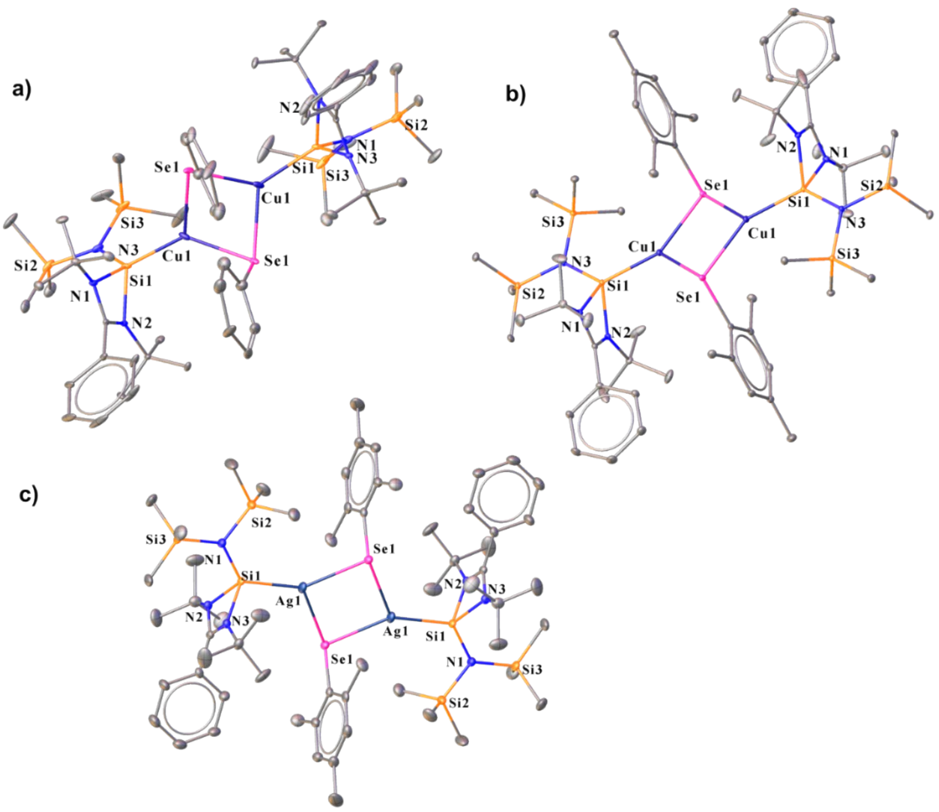

Figure S139. Molecular structure of a) **14**, b) **15**, and c) **16**. Anisotropic displacement parameters are depicted at the 50% probability level. Disordered aromatic rings and H atoms are omitted for clarity. Selected bond distances (Å) and angles (deg): for **a)** Si1–Cu1 2.248(3), Cu1–Se1 2.456(1), Si1–N3 1.743(7), N3–Si3 1.770(8), N3–Si2 1.76(1), Si1–N2 1.848(6), Si1–N1 1.862(6) and Si1–Cu1–Se1 133.89(8), N3–Si1–Cu1 125.8(3), N3–Si1–N1 112.6(3), N2–Si1–Cu1 117.0(2), N1–Si1–N2 70.4(3), for **b)** Si1–Cu1 2.2631(6), Cu1–Se1 2.4579(6), Cu1–Se1 2.5076(6), Si1–N3 1.743(1), Si1–N2 1.848(1), Si1–N1 1.853(1), Cu1–Cu1 3.4398(6) and Si1–Cu1–Se1 143.51(2), Si1–Cu1–Se1 124.12(2), Se1–Cu1–Se1 92.31(1), N3–Si1–Cu1 128.42(5), N3–Si1–N1 109.30(7), N2–Si1–Cu1 113.93(5), N1–Si1–N2 70.45(6) and for **c)** Si1-Ag1 2.401(1), Ag1-Se1 2.6245(7), Si1-N2 1.845(4), Si1-N3 1.840(4), Si1-N1 1.728(5), Ag1-Ag1 3.8110(7) Å and Si1-Ag1-Se1 146.34(4), Si1-Ag1-Se1 123.17(4), Se1-Ag1-Se1 90.49(2), N2-Si1-N3 70.6(2), Ag1-Si1-N3 110.1(1), Ag1-Si1-N1 126.8(2), N1-Si1-N2 111.2(2).

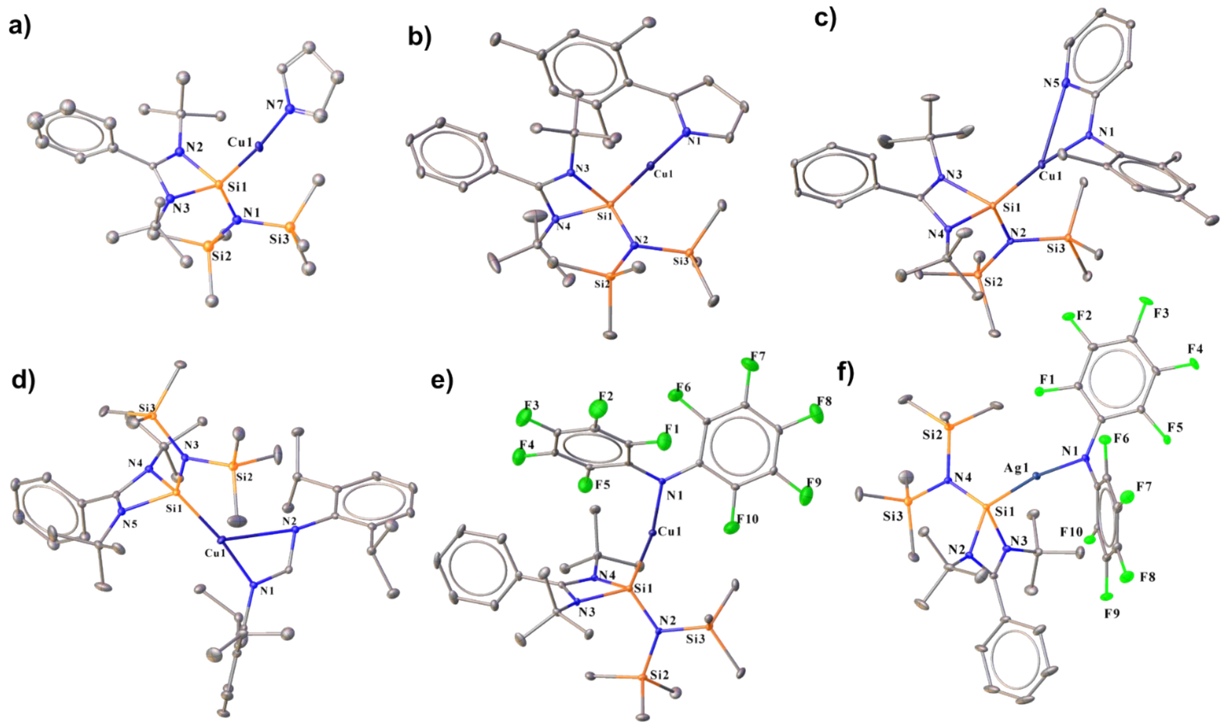

Figure S140. Molecular structure of a) **17**, b) **18**, c)**19**, d) **20**, e) **21**, and f) **22.** Anisotropic displacement parameters are depicted at the 50% probability level. H atoms are omitted for clarity. Selected bond distances (Å) and angles (deg): for **a)** Si1-Cu1 2.231(3), Cu1-N7 1.878(7), Si1-N1 1.748(8), Si1-N2 1.844(7), Si1-N3 1.846(8), N1-Si3 1.781(7), N1-Si2 1.756(7) and Si1-Cu1-N7 172.0(2), Cu1-Si1-N1 123.1(3), Cu1-Si1-N2 108.3(3), N1-Si1-N3 112.7(4), N2-Si1-N3 70.7(3); **b)** Si1–Cu1 2.203(1), Cu1–

N1 1.874(3), Si1–N2 1.730(5), N2–Si2 1.761(4), N2–Si3 1.770(3), Si1–N3 1.842(3), Si1–N4 1.837(4) and Si1–Cu1–N1 177.5(1), N2–Si1–Cu1 118.8(1), N2–Si1–N4 112.5(2), N3–Si1–Cu1 113.4(1), N3–Si1–N4 71.2(2); **c)** Si1–Cu1 2.1964(8), Cu1–N1 1.906(2), Cu1–N5 2.654(2), Si1–N4 1.842(2), Si1–N3 1.838(2), Si1–N2 1.740(2), N2–Si3 1.770(2), N2–Si2 1.754(2) and Si1–Cu1–N1 172.44(7), Si1–Cu1–N5 128.28(6), N5–Cu1–N1 57.09(8), N3–Si1–Cu1 114.98(8), N3–Si1–N2 112.6(1), N4–Si1–N2 112.6(1), Cu1–Si1–N2 118.48(8); and **d)** Si1–Cu1 2.194(1), Cu1–N1 1.903(3), Cu1–N2 2.712(3), N2–C13 1.294(4), C13–N1 1.337(4), Si1–N5 1.837(3), Si1–N4 1.845(3), Si1–N3 1.719(3), N3–Si3 1.761(3), N3–Si2 1.768(3) and Si1–Cu1–N1 171.24(9), Si1–Cu1–N2 129.22(6), N1–Cu1–N2 56.1(1), N1–C13–N2 120.6(3), N3–Si1–N5 112.4(1), N3–Si1–N4 114.2(1), Cu1–Si1–N5 112.4(1), N3–Si1–Cu1 124.8(1), N4–Si1–N5 70.7(1). **e)** Si1–Cu1 2.220(1), Cu1–N1 1.929(4), N1–C1 1.387(5), N1–C7 1.383(5), Si1–N2 1.724(3), N2–Si2 1.754(4), N2–Si3 1.774(3), Si1–N3 1.836(4), Si1–N4 1.846(3) and Si1–Cu1–N1 169.3(1), N4–Si1–N3 70.7(2), N4–Si1–N2 112.4(2), N2–Si1–Cu1 123.5(1), N4–Si1–Cu1 113.3(1); **f)** Si1-Ag1 2.3543(8), Ag1-N1 2.167(3), Si1-N2 1.843(2), Si1-N3 1.842(3), Si1-N4 1.719(2) and Si1-Ag1-N1 169.86(7), Ag1-Si1-N2 111.86(8), Ag1-Si1-N4 123.21(9), N4-Si1-N3 114.0(1), N2-Si1-N3 71.1(1);

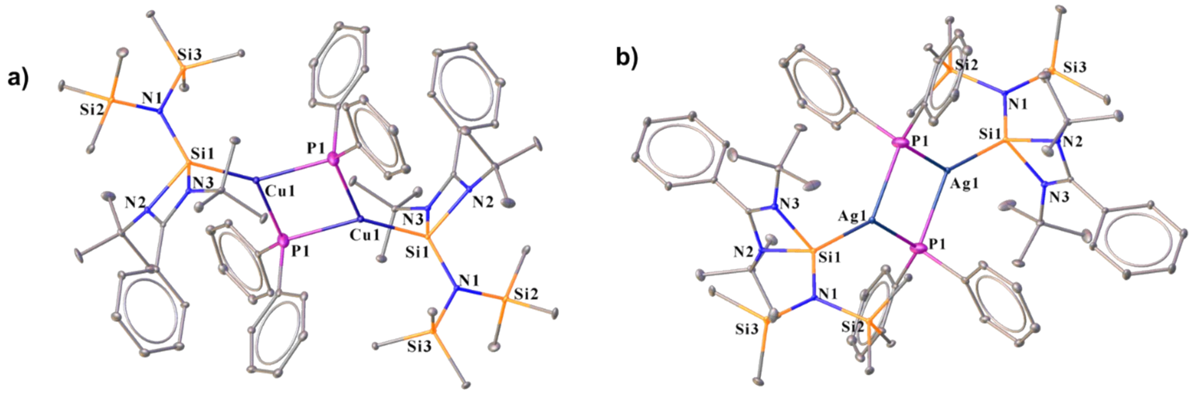

Figure S141. Molecular structure of a) **23** and b)**24**. Anisotropic displacement parameters are depicted at the 50% probability level. H atoms are omitted for clarity. Selected bond distances (Å) and angles (deg): for **a)** Si1-Cu1 2.2758(8), Cu1-P1 2.3799(8), Cu1-P1 2.3530(8), Si1-N1 1.747(2), Si1-N3 1.854(2), Si1-N2 1.865(2), Cu1-Cu1 3.4570(8) and Si1-Cu1-P1 135.37(3), P1-Cu1-P1 86.16(3), Si1-Cu1-P1 136.92(3), Cu1-Si1-N1 130.64(7), Cu1-Si1-N2 113.70(7), N2-Si1-N3 70.24(9), N3-Si1-N1 110.6(1) °; for **b)** Si1-Ag1 2.401(1), Ag1-P1 2.537(1), Ag1-P1 2.584(1), Ag1-Ag1 3.7907(7), Si1-N2 1.857(3), Si1-N1 1.743(4), Si1-N3 1.850(4) and Si1-Ag1-P1

137.45(4), Si1-Ag1-P1 135.84(4), P1-Ag1-P1 84.50(4), Ag1-Si1-N3 112.0(1), Ag1-Si1-N1 130.2(1), N3-Si1-N2 70.5(2), N2-Si1-N1 111.7(2).

S7. Reactivity of complex 2 with B_2pin_2

To probe complex **2** for the homolytic diboron bond cleavage, we performed a stoichiometric reaction of complex **2** with bis(pinacolato)diboron (B_2pin_2) in toluene at room temperature. However, we obtained the single crystals of an unprecedented cocrystallised compound **S2** instead of the B–B bond cleavage. The molecular structure of **S2** reveals that the two units are stabilised by several non-covalent interactions, as seen from the Hirshfeld analysis at 0.5 iso-surface. The Hirshfeld analysis shows that the two units are stabilised *via* several C–H···B and O···H interactions—two distinct, independent molecules are crystallised in the asymmetric unit (Figure S148). The Si1–Cu1 bond length in compound **S2** is found to be 2.246(2) Å, which is shorter than the Si–Cu bond in complex **2**. The Cu1–C1 bond [1.939(6)] Å of **S2** is marginally shorter than the Cu–C bond [1.946(2)] in the complex **2**. Several non-covalent interactions stabilise complex **S2**, which features C–H···B interaction, with a distance ranging from 3.0-3.1 Å, which is marginally shorter than the sum of their van der Waal radii (3.12 Å). Cremer and co-workers recently established B–H···π interaction (**Chart S1**).[31] We observed charge transfer from the σ-orbital of the C–H group to the vacant p-orbital of the boron centre in its gas-phase optimised structure, along with strong C–H···O interaction. Conversely, when mesityl copper was treated directly with Bpin–Bpin in the presence of PPh_3, Mes–Bpin was obtained.[32] Observing the reaction pattern, we rationalised that the enhanced steric bulk of B_2pin_2 might have prevented the desired product formation. We also tried the reaction at elevated temperature to access the NHSi–Cu–Bpin complex, but could not isolate the desired complex.

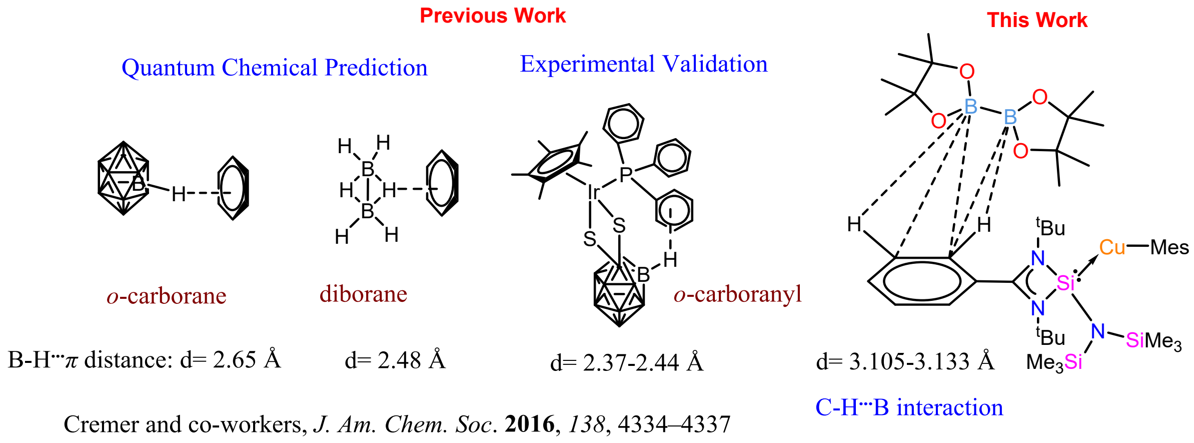

Chart S1. Reported examples of B-H···π interactions and this work, representing C-H···B interactions.

<u>Preparation of **S2**:</u> A Schlenk flask charged with compound **2** (210.42 mg, 0.5 mmol) and mesityl copper (91.37 mg, 0.5 mmol) followed by the addition of toluene (20 ml) and kept in stirring for 4 h. Bpin-Bpin (127mg, 0.5 mmol) was added to the solution and kept in stirring overnight under an inert atmosphere of Argon (Ar). The dark red-coloured solution was filtered off and concentrated to 2 ml to obtain single crystals at room temperature. Yield: 171 mg (40 %). ^{1}H NMR (400 MHz, C_6D_6, 298 K): δ 0.45 (s, 9H, Si-C*H*$_3$), 0.65 (s, 9H, Si-C*H*$_3$), 1.15 (s, 18H, {N-C(C*H*$_3$)$_3$}), 1.24 (s, 24H, -C*H*$_3$) 2.15 (s, 3H, *p*-C*H*$_3$), 2.55 (s, 6H, *o*-C*H*$_3$), 6.94-6.97 (m, 5H, arom. H), 7.28 (d, 2H, arom. H) ppm; ^{13}C{^{1}H} NMR (100.613 MHz, C_6D_6, 298 K): δ 4.05 (Si-*C*H$_3$), 4.56 (Si-*C*H$_3$), 21.63 (mesityl *C*H$_3$), 23.83 (mesityl *C*H$_3$), 23.97 (-*C*H$_3$ of pinB-pinB) 30.96 {N-C(*C*H$_3$)$_3$}, 52.55 {N-*C*(CH$_3$)$_3$}, 82.01 {-*C*(CH$_3$)$_2$ of pinB-pinB} 123.97, 128.11, 128.60, 133.64, 137.85, 141.83, 145.45, 155.70, (arom. C) ppm ^{29}Si{^{1}H} NMR (79.49 MHz, C_6D_6, 298 K): δ 3.7 [-N(*Si*Me$_3$)$_2$], 2.8 [-N(*Si*Me$_3$)$_2$], -8.1 (*Si*-Cu); ^{11}B {^{1}H} NMR (C_6D_6, 298 K, 128 MHz): δ 32.8 (pin*B*-*B*pin) ppm. HRMS (ESI, CH_3CN) (*m/z*) for [$C_{42}H_{76}B_2CuN_3O_4Si_3$] calc. 855.4625; found 855.4675 [M]$^+$.

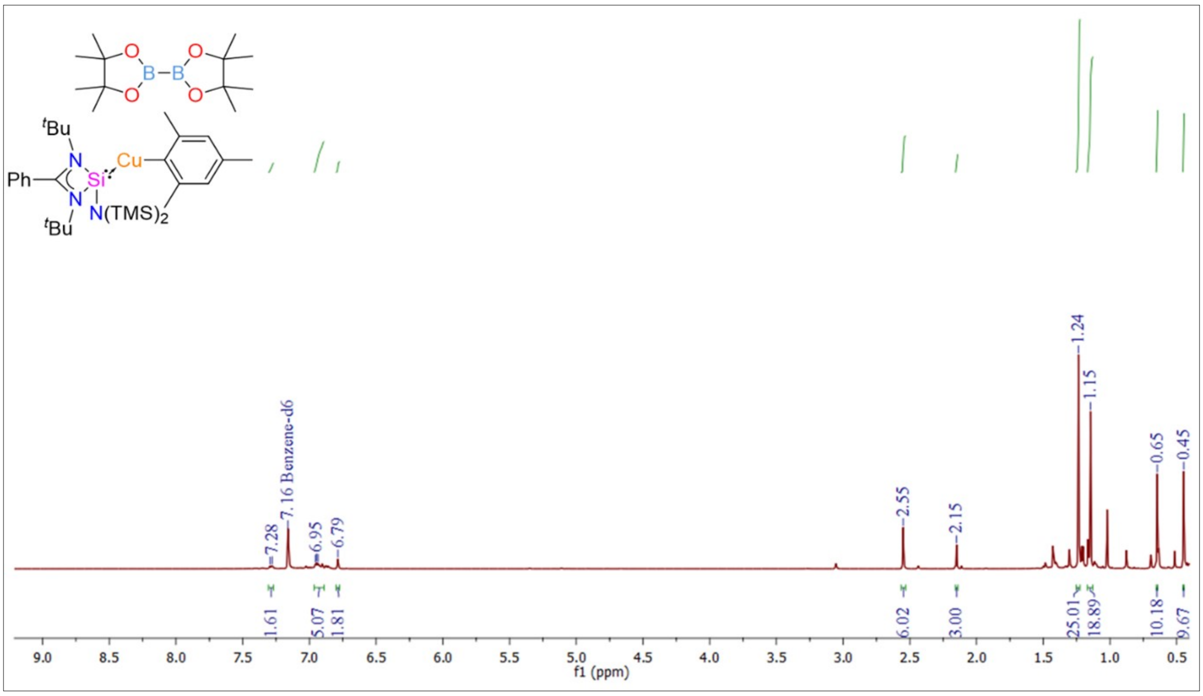

Figure S142. ^{1}H NMR spectrum of complex **S2**.

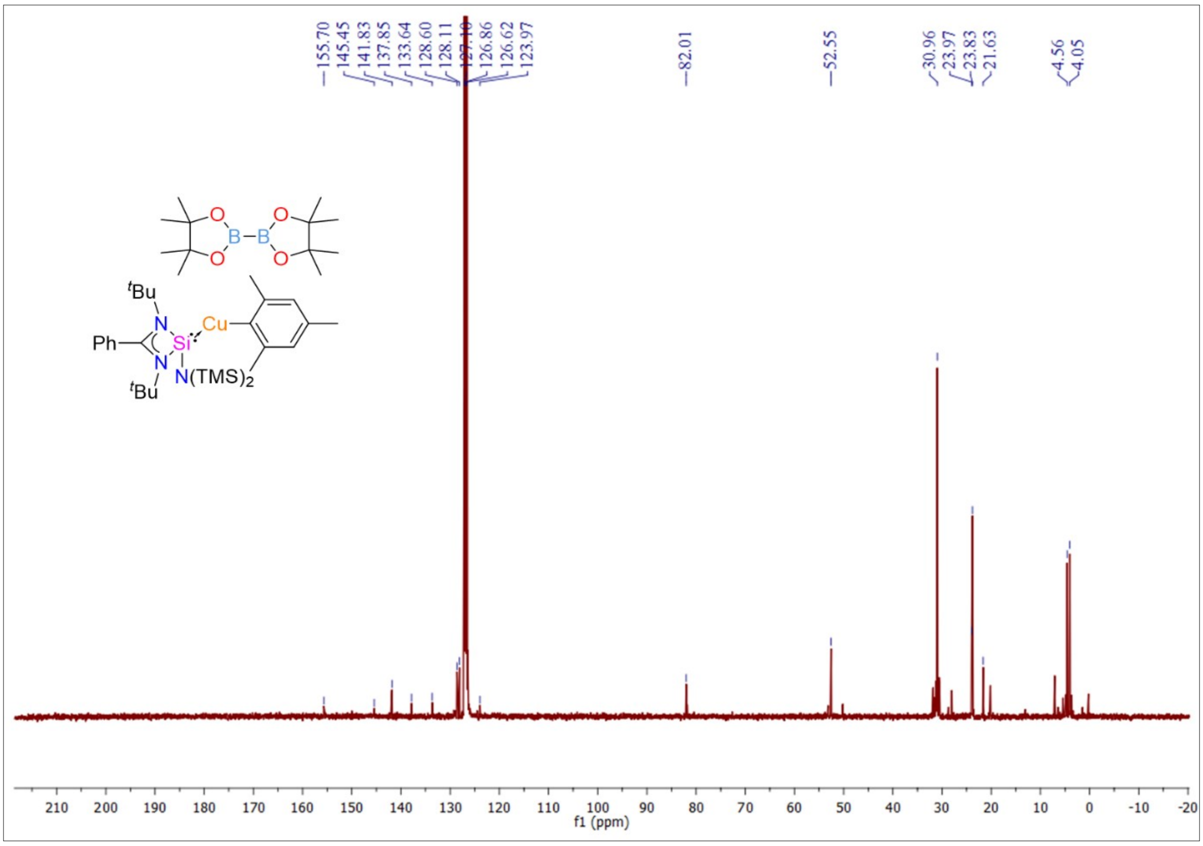

Figure S143. $^{13}C\{^1H\}$ NMR spectrum of complex **S2**.

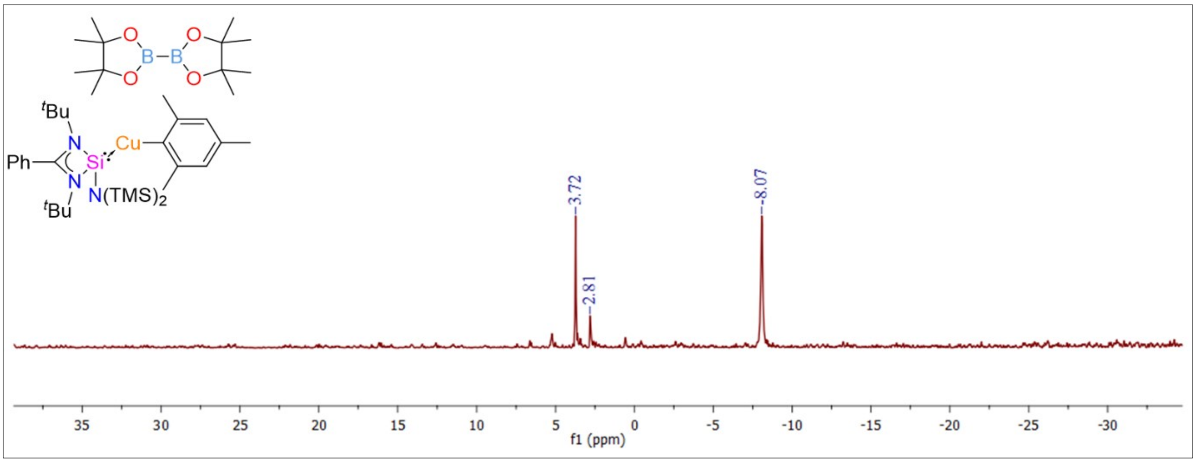

Figure S144. $^{29}Si\{^1H\}$ NMR spectrum of complex **S2**.

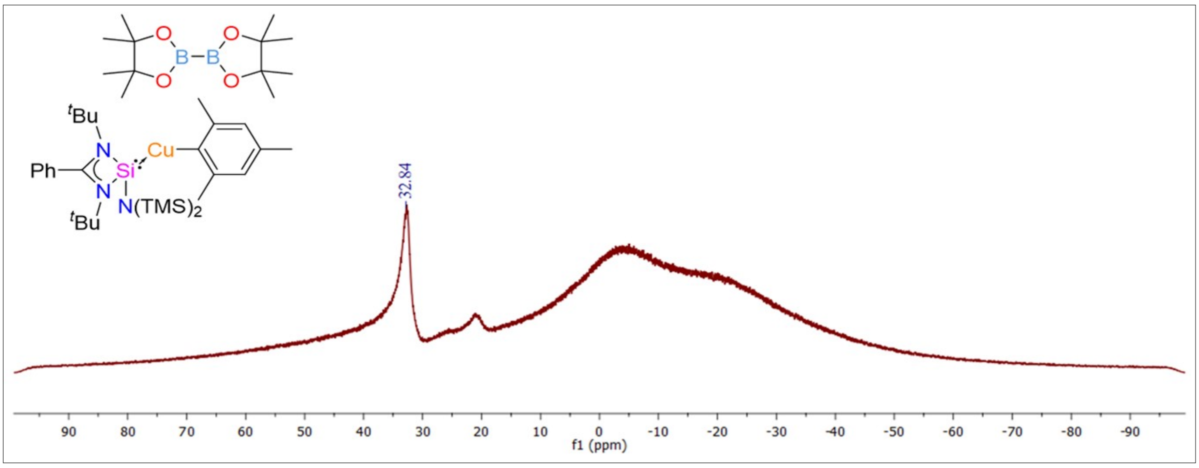

Figure S145. $^{11}B\{^1H\}$ NMR spectrum of complex **S2**.

❖ Molecular Structure and Packing of Complexes S2

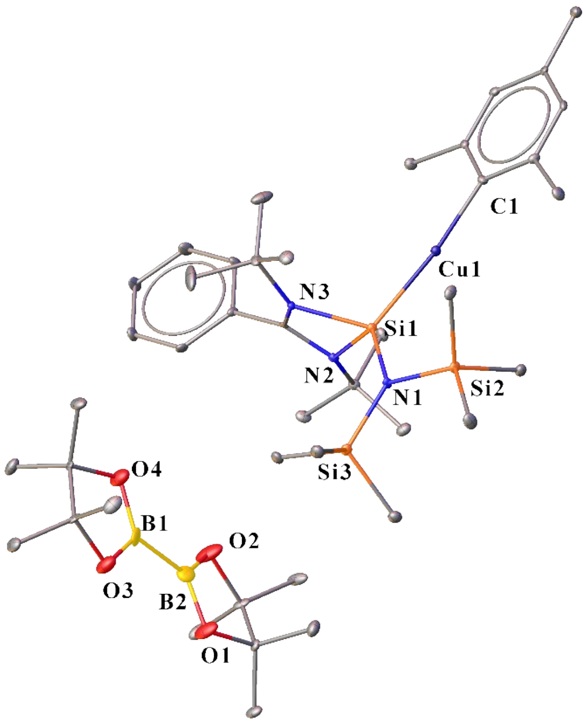

Figure S146. Molecular structure of **S2**. Anisotropic displacement parameters are depicted at the 50% probability level. Solvent molecule and H atoms are omitted for clarity. Selected bond distances (Å) and angles (deg): Si1-Cu1 2.246(2), Cu1-C1 1.939(6), Si1-N1 1.732(4), N1-Si3 1.755(5), N1-Si2 1.767(5), Si1-N3 1.848(4), Si1-N2 1.842(4), B2-B1 1.688(6), B2-O2 1.354(7), B2-O1 1.365(6), B1-O3 1.368(6), B1-O4 1.360(6) and Si1-Cu1-C1 172.8(2), N3-Si1-Cu1 113.9(1), N3-Si1-N2 70.3(2), N2-Si1-N1 113.0(2), Cu1-Si1-N1 122.2(2), O2-B2-O1

112.9(5), B1-B2-O2 125.5(5), O1-B2-B1 121.6(5), O3-B1-O4 112.6(4), O3-B1-B2 121.7(4), B2-B1-O4 125.6(4).

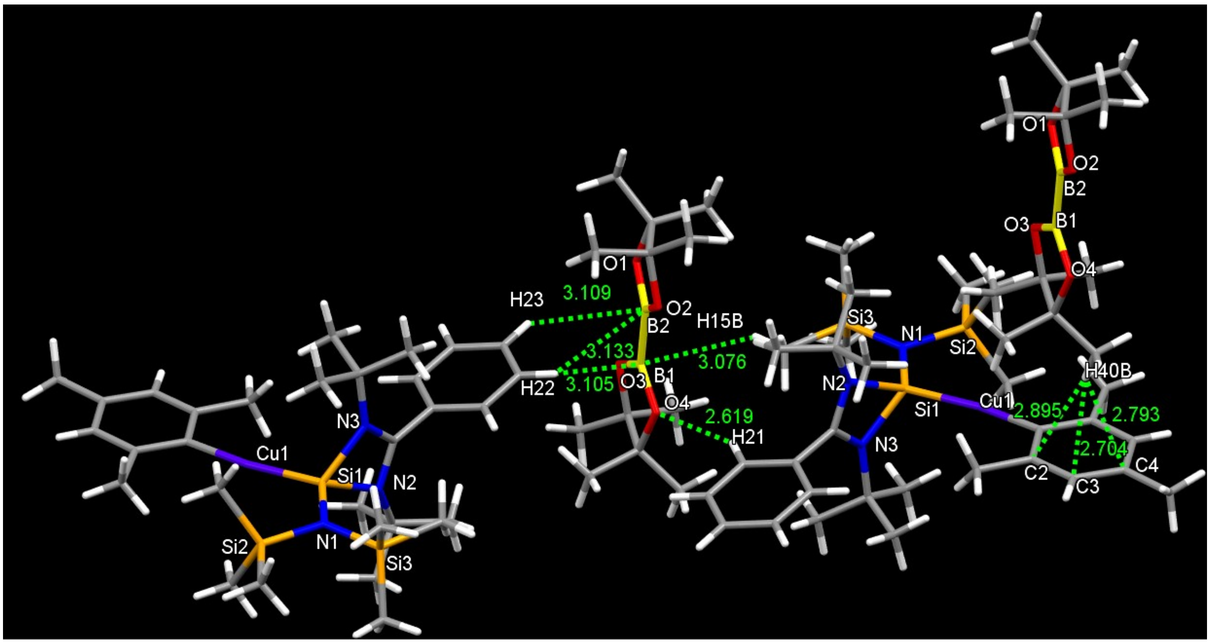

Figure S147. Various weak interactions (C–H···B and C–H···O) stabilising complex **S2s**.

❖ Hirshfield Analysis of Complex S2

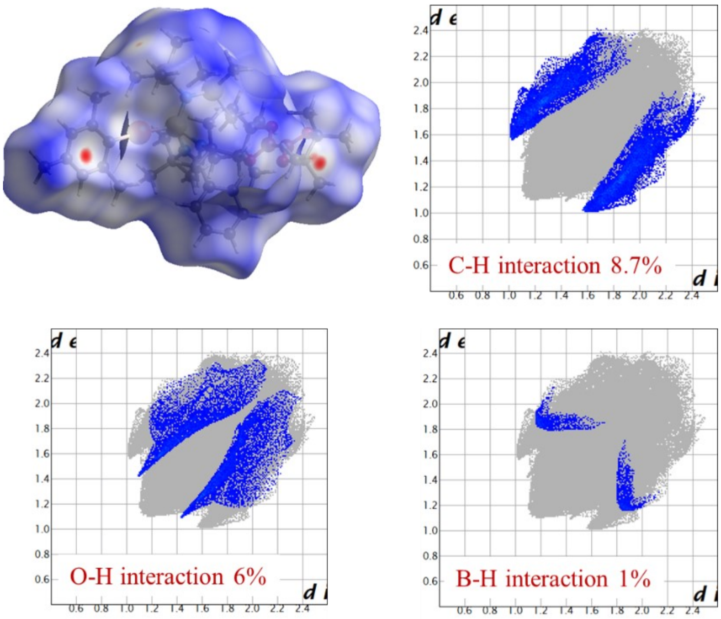

Figure S148. Hirshfield Analysis of Complex **S2**.

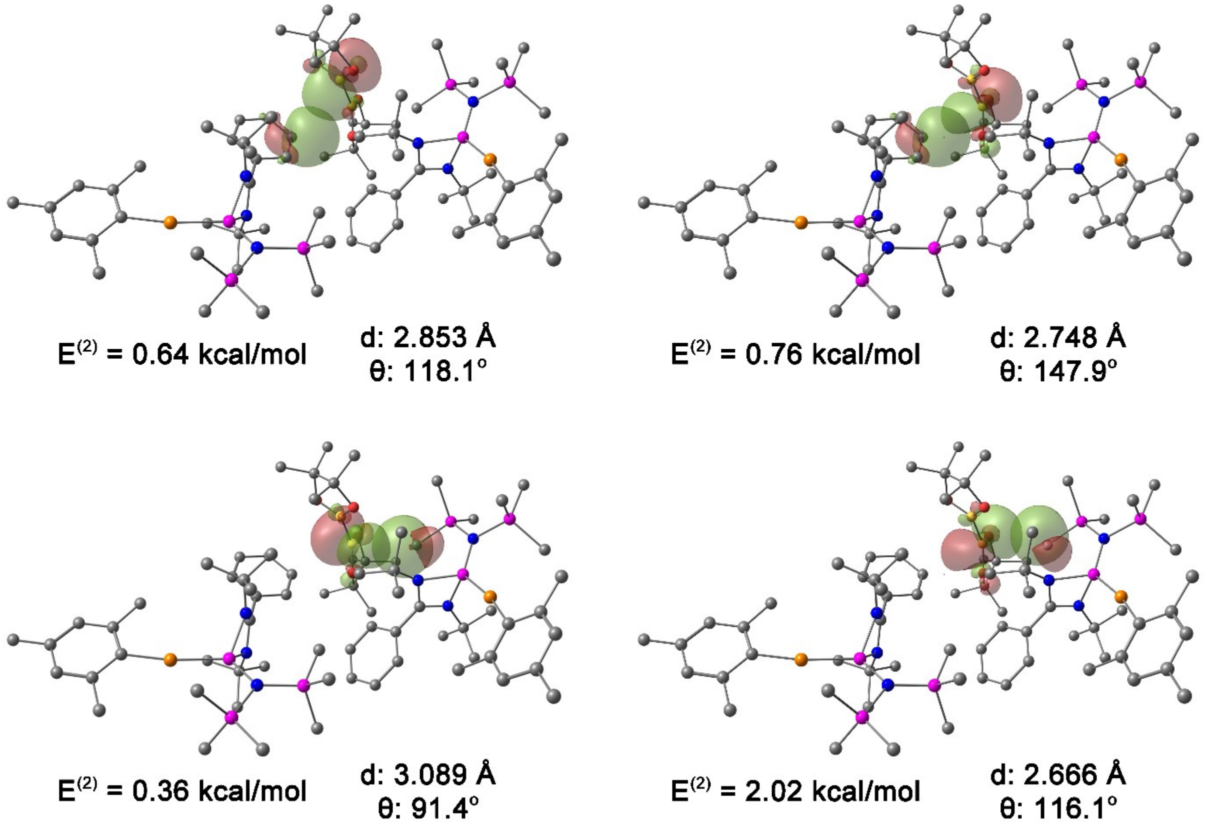

Figure S149. NBO images of complex **S2** with second-order perturbative energies for charge transfer from the σ-orbital of C–H to the vacant p-orbital of the boron centre and relevant geometrical parameters such as distance (d) and angle (θ).

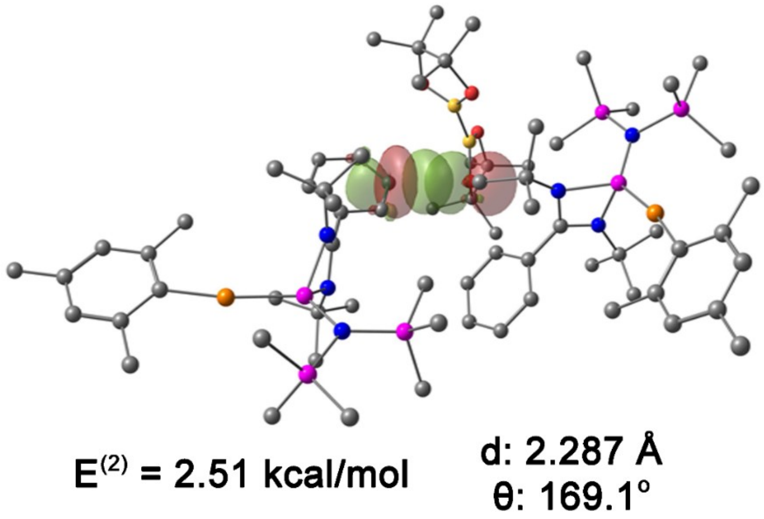

Figure S150. NBO images of complex **S2** with second-order perturbative energies for C–H···O interaction and relevant geometrical parameters such as distance (d) and angle (θ).

References

1. a) R. Azhakar, R. S. Ghadwal, H. W. Roesky, H. Wolf and D. Stalke, *Organometallics*, 2012, ***31***, 4588-4592; b) S. S. Sen, H. W. Roesky, D. Stern, J. Henn and D. Stalke, *J. Am. Chem. Soc.*, 2010, ***132***, 1123-1126.
2. a) H. Bhattacharjee, J. Zhu and J. Müller, *Angew. Chem. Int. Ed.*, 2019, ***58***, 16575-16582; b) T. Tsuda, T. Yazawa, K. Watanabe, T. Fujii and T. Saegusa, *J. Org. Chem.*, 1981, ***46***, 192-194; c) E. M. Meyer, S. Gambarotta, C. Floriani, A. Chiesi-Villa and C. Guastini, *Organometallics*, 1989, ***8***, 1067-1079.
3. P. Ramirez-Lopez, A. Ros, B. Estepa, R. Fernandez, B. Fiser, E. Gomez-Bengoa and J. M. Lassaletta, *ACS Catal.*, 2016, ***6***, 3955-3964.
4. D. K. Kennepohl, S. Brooker, G. M. Sheldrick and H. W. Roesky, *Chem. Ber.*, 1991, ***124***, 2223-2225.
5. T. Umemoto and K. Adachi, *J. Org. Chem.*, 1994, ***59***, 5692-5699.
6. R. D. Rieth, N. P. Mankad, E. Calimano and J. P. Sadighi, *Org. Lett.*, 2004, ***6***, 3981-3983.
7. S. Manna, P. O. Serebrennikova, I. A. Utepova, A. P. Antonchick and O. N. Chupakhin, *Org. Lett.*, 2015, ***17***, 4588-4591.
8. K. M. Kuhn and R. H. Grubbs, *Org. Lett.*, 2008, ***10***, 2075-2077.
9. H. Yin, A. J. Lewis, U. J. Williams, P. J. Carroll and E. J. Schelter, *Chem. Sci.*, 2013, ***4***, 798-805.
10. A. Sumida, K. Ogawa, H. Imoto and K. Naka, *Dalton Trans.*, 2023, ***52***, 2838-2844.
11. J. Yang, Q.-Y. Han, C.-L. Zhao, T. Dong, Z.-Y. Hou, H.-L. Qin and C.-P. Zhang, *Org. Biomol. Chem.*, 2016, ***14***, 7654-7658.
12. a) S. M. Wang, X. Y. Wang, H. L. Qin and C. P. Zhang, *Chem. Eur. J.*, 2016, ***22***, 6542-6546; b) T. Hatakeyama, S. Hashimoto, K. Ishizuka and M. Nakamura, *J. Am. Chem. Soc.*, 2009, ***131***, 11949-11963; c) A. Biswas, A. Bhunia and S. K. Mandal, *Chem. Sci.*, 2023, ***14***, 2606-2615.
13. W. Su, S. Urgaonkar, P. A. McLaughlin and J. G. Verkade, *J. Am. Chem. Soc.*, 2004, ***126***, 16433-16439.
14. a) D.-D. Lu, X.-X. He and F.-S. Liu, *J. Org. Chem.*, 2017, ***82***, 10898-10911; b) M. G. Organ, M. Abdel-Hadi, S. Avola, N. Hadei, J. Nasielski, C. J. O'brien and C. Valente, *Chem. Eur. J.*, 2007, ***13***, 150-157.
15. V. K. Pandey, C. S. Tiwari and A. Rit, *Chem. Asian J.*, 2022, ***17***, e202200703.

16. a) J. Wang, Y. Masui and M. Onaka, Synthesis of α-Amino Nitriles from Carbonyl Compounds, Amines, and Trimethylsilyl Cyanide: Comparison between Catalyst-Free Conditions and the Presence of Tin Ion-Exchanged Montmorillonite.*Journal*, 2010; b) M. A. Kumar, M. S. Babu, K. Srinivasulu, Y. Kiran and C. S. Reddy, *J. Mol. Catal. A Chem.*, 2007, ***265***, 268-271.
17. a) Y. Tao, S. She, X. Wang, F. Wang, X. Ji, B. Yan, C. Chu and S. Wang, *J. Organomet. Chem.*, 2022, ***970***, 122357; b) S. K. Sachan and G. Anantharaman, *Inorg. Chem.*, 2021, ***60***, 9238-9242.
18. R. Neufeld and D. Stalke, *Chem. Sci.*, 2015, ***6***, 3354-3364.
19. D. Li, I. Keresztes, R. Hopson and P. G. Williard, *Acc. Chem. Res.*, 2009, ***42***, 270-280.
20. O. V. Dolomanov, L. J. Bourhis, R. J. Gildea, J. A. K. Howard and H. Puschmann, *J. Appl. Crystallogr.*, 2009, ***42***, 339-341.
21. G. M. Sheldrick, *Acta Crystallogr. C Struct. Chem.*, 2015, ***71***, 3-8.
22. A. L. Spek, *J. Appl. Crystallogr.*, 2003, ***36***, 7-13.
23. M. J. Frisch, G. W. Trucks, H. B. Schlegel, G. E. Scuseria, M. A. Robb, J. R. Cheeseman, G. Scalmani, V. Barone, B. Mennucci, G. A. Petersson, H. Nakatsuji, M. Caricato, X. Li, H. P. Hratchian, A. F. Izmaylov, J. Bloino, G. Zheng, J. L. Sonnenberg, M. Hada, M. Ehara, K. Toyota, R. Fukuda, J. Hasegawa, M. Ishida, T. Nakajima, Y. Honda, O. Kitao, H. Nakai, T. Vreven, J. Montgomery, J. A., J. E. Peralta, F. Ogliaro, M. Bearpark, J. J. Heyd, E. Brothers, K. N. Kudin, V. N. Staroverov, R. Kobayashi, J. Normand, K. Raghavachari, A. Rendell, J. C. Burant, S. S. Iyengar, J. Tomasi, M. Cossi, N. Rega, N. J. Millam, M. Klene, J. E. Knox, J. B. Cross, V. Bakken, C. Adamo, J. Jaramillo, R. Gomperts, R. E. Stratmann, O. Yazyev, A. J. Austin, R. Cammi, C. Pomelli, J. W. Ochterski, R. L. Martin, K. Morokuma, V. G. Zakrzewski, G. A. Voth, P. Salvador, J. J. Dannenberg, S. Dapprich, A. D. Daniels, Ö. Farkas, J. B. Foresman, J. V. Ortiz, J. Cioslowski and D. J. Fox, *GAUSSIAN 09, Revision D.01*, Gaussian, Inc., Wallingford CT (**2009**).
24. E. D. Glendening, C. R. Landis and F. Weinhold, *J. Comput. Chem.*, 2019, ***40***, 2234-2241.
25. a) S. Miertuš, E. Scrocco and J. Tomasi, *Chem. Phys.*, 1981, ***55***, 117-129; b) J. Tomasi, B. Mennucci and R. Cammi, *Chem. Rev.*, 2005, ***105***, 2999-3094.
26. S. F. Boys and F. Bernardi, *Mol Phys*, 1970, ***19***, 553-566.

27. H. Iwamoto, K. Endo, Y. Ozawa, Y. Watanabe, K. Kubota, T. Imamoto and H. Ito, *Angew. Chem. Int. Ed.*, 2019, ***58***, 11112-11117.
28. A. L. Speelman, B. L. Tran, J. D. Erickson, M. Vasiliu, D. A. Dixon and R. M. Bullock, *Chem. Sci.*, 2021, ***12***, 11495-11505.
29. S. Pahar, V. Sharma, K. V. Raj, M. P. Sangole, C. P. George, K. Singh, K. Vanka, R. G. Gonnade and S. S. Sen, *Chem. Eur. J.*, 2024, ***30***, e202303957.
30. a) R. F. Bader, *Acc. Chem. Res.*, 1985, ***18***, 9-15; b) T. Lu and F. W. Chen, *J. Comput. Chem.*, 2012, ***33***, 580-592.
31. X. Zhang, H. Dai, H. Yan, W. Zou and D. Cremer, *J. Am. Chem. Soc.*, 2016, ***138***, 4334-4337.
32. H. Yoshida, S. Kawashima, Y. Takemoto, K. Okada, J. Ohshita and K. Takaki, *Angew. Chem. Int. Ed.*, 2012, ***51***, 235-238.
